# De novo and somatic structural variant discovery with SVision-pro

**DOI:** 10.1038/s41587-024-02190-7

**Published:** 2024-03-22

**Authors:** Songbo Wang, Jiadong Lin, Peng Jia, Tun Xu, Xiujuan Li, Yuezhuangnan Liu, Dan Xu, Stephen J. Bush, Deyu Meng, Kai Ye

**Affiliations:** 1 Department of Gynecology and Obstetrics, Center for Mathematical Medical, The First Affiliated Hospital of Xi’an Jiaotong University, Xi’an, China; 2 School of Automation Science and Engineering, Faculty of Electronic and Information Engineering, Xi’an Jiaotong University, Xi’an, China; 3 MOE Key Lab for Intelligent Networks & Networks Security, Faculty of Electronic and Information Engineering, Xi’an Jiaotong University, Xi’an, China; 4 School of Life Science and Technology, Xi’an Jiaotong University, Xi’an, China; 5 School of Mathematics and Statistics, Xi’an Jiaotong University, Xi’an, China; 6 Macau Institute of Systems Engineering, Macau University of Science and Technology, Taipa, Macau; 7 Pazhou Laboratory (Huangpu), Guangzhou, Guangdong, China; 8 Faculty of Science, Leiden University, Leiden, The Netherlands; 9 Genome Institute, The First Affiliated Hospital of Xi’an Jiaotong University, Xi’an, China

**Keywords:** Genomics, Genetic variation, Genome informatics, Software, Machine learning

## Abstract

Long-read-based de novo and somatic structural variant (SV) discovery remains challenging, necessitating genomic comparison between samples. We developed SVision-pro, a neural-network-based instance segmentation framework that represents genome-to-genome-level sequencing differences visually and discovers SV comparatively between genomes without any prerequisite for inference models. SVision-pro outperforms state-of-the-art approaches, in particular, the resolving of complex SVs is improved, with low Mendelian error rates, high sensitivity of low-frequency SVs and reduced false-positive rates compared with SV merging approaches.

## Abstract (web summary)

SVision-pro enables pairwise genome comparison and improves de novo and somatic structural variant calling.

## Main

Long-read sequencing (LRS) technologies have greatly facilitated the detection of SVs^1^, including simple SVs (SSV)^2–5^ and complex SVs (CSVs)^6^, which typically comprise several internal SSV subcomponents. Given that de novo and somatic SVs^7,8^ are responsible for Mendelian disorders^9,10^ and development of cancers^11,12^, comparative SV discovery between genomes (for example, comparing a proband genome against parent genomes to identify de novo SVs) has generally been attempted by either callset-merge or read-inference strategies. Callset-merge strategies^13–15^ (for example, Jasmine) extract genome-specific calls from merged callsets and hence inevitably incorporate the miscalls from callers, leading to many false positives. In contrast, read-inference strategies^16^ (for example, nanomonsv) directly search differential alignments between genomes and construct SV inference models. However, this is typically limited to SSVs, and CSV modeling cannot be accommodated due to the unexplored CSV types and nested internal components^17^. Although sequencing-to-image and deep-learning-based callers have improved CSV characterization^6,18^, two principal issues hinder their application to comparative SV discovery. First, existing sequencing-to-image schemas can represent SVs only of an individual genome, whereas comparative SV discovery requires additional image features that can represent SV differences between genomes. Second, comparative SV discovery demands several recognition tasks to detect and genotype SV between genomes simultaneously, while current single-task deep-learning callers classify one entire image into either a specific SV type^6,19^ or genotype^20^.

Here we propose SVision-pro, comprising two key modules: a sequence-to-image representation module encoding genomic features from two samples in a single image, from which a neural-network recognition module comparatively recognizes SVs as well as their intergenome differences. SVision-pro integrates SV detection and genotyping between genomes as a one-stop neural-network-based image instance segmentation task, facilitating the discovery of both de novo and somatic SSVs and CSVs.

**Extended Data Fig. 1:**
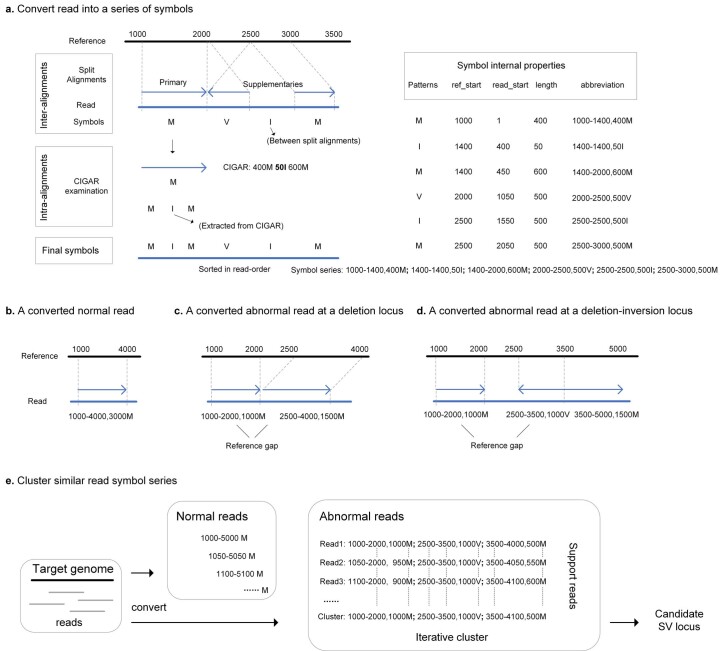
Illustration of the candidate SV locus searching step in SVision-pro. **a**, SVision-pro converts each read into a series of symbols, including ‘M’, ‘V’ and ‘I’, based on the aligner’s output. Staring with inter-alignment examination, primary alignment and supplementary alignments of the read are directly converted into ‘M’ and ‘V’ according to their mapping orientation. Unmapped sequence between split alignments are converted into ‘I’. For each alignments, SVision-pro further examine their CIGAR string (intra-alignment) to retrieve more ‘I’s. Consequently, a read is converted into a series of symbols arranged in their occurrence on read sequence. Each symbol contains several inner properties, including start position on reference sequence, start position on read sequence and its length. Each symbol can be abbreviated as ‘reference_start-reference_end, length and symbol type’ for subsequent clustering step. **b**, An example of converting a normal read into a symbol series. **c**, An example of converting an abnormal read, which spans a deletion, into a symbol series. **d**, An example of converting an abnormal read, which spans a CSV deletion-inversion, into a symbol series. **e**, For a genome locus, normal reads, which contain only one ‘M’ in their symbol series, are filtered out. The remaining abnormal reads are iteratively clustered together by comparing their symbol series to identify candidate SV loci.

The sequence-to-image representation module first takes as input aberrant genome loci identified from LRS data. In contrast to traditional LRS-based callers, which search for SV-specific alignment signatures, SVision-pro summarizes each read into a series of symbols (Extended Data Fig. 1a–d and Methods). These one-dimensional (1D)-symbol series are obtained directly from read alignment results without any SV-type-oriented preprocessing, and then clustered together iteratively as candidate aberrant loci (Extended Data Fig. 1e). This process, without matching known SV types, ensures the comprehensive capture of SV loci, especially for unexplored CSVs. The SV-type-classification task is delegated to subsequent representation and recognition modules.

**Fig. 1:**
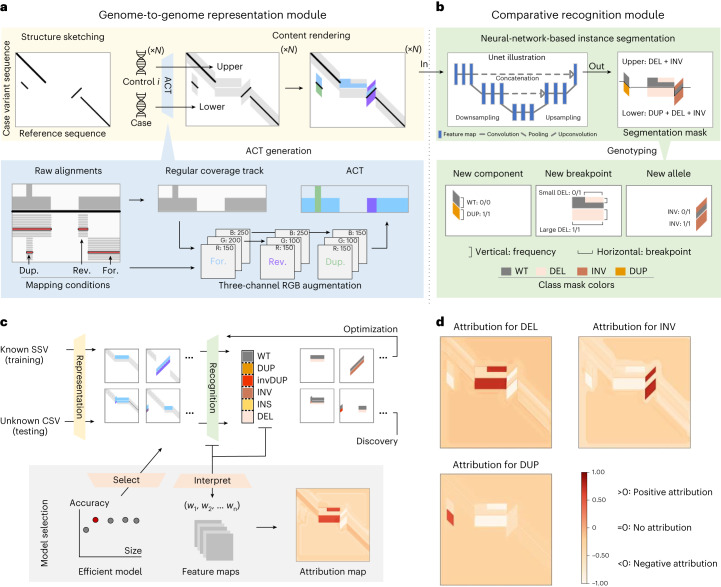
SVision-pro overview. **a**, Overview of the sequence-to-image representation module in SVision-pro. SVision-pro sketches the structures of a candidate SV locus and renders ACTs (above) into the sparse image regions. The ACT is generated from mapped alignments by the three-channel RGB augmentation (below). Dup., duplicated-matching; Rev., reversed-matching; For., forward-matching. **b**, Overview of the comparative recognition module in SVision-pro. The neural-network-based instance segmentation framework outputs a segmentation mask, providing intuitive SV types (above). By comparative genotyping analysis of the colored regions in the upper and lower panels (below), we can determine the SV differences between case and control genomes. **c**, Neural-network model training and selection strategy of SVision-pro. SVision-pro was trained with five basic SV subcomponent types along with wild type (identical to reference genome) and was able to recognize CSVs with several internal subcomponents (above). To select an efficient instance segmentation models (red solid circle), we leveraged three factors: validation accuracy, parameter size and interpretability. **d**, Attribution maps of the Lite-Unet model. Pixels relevant for a certain prediction class are highlighted. DEL, deletion; DUP, duplication; INV, inversion; INS, insertion; invDUP, inverted-duplication; WT, wild type; R, red; G, green; B, blue; w_1_, w_2_ and w_n_, parameter weights.

**Extended Data Fig. 2:**
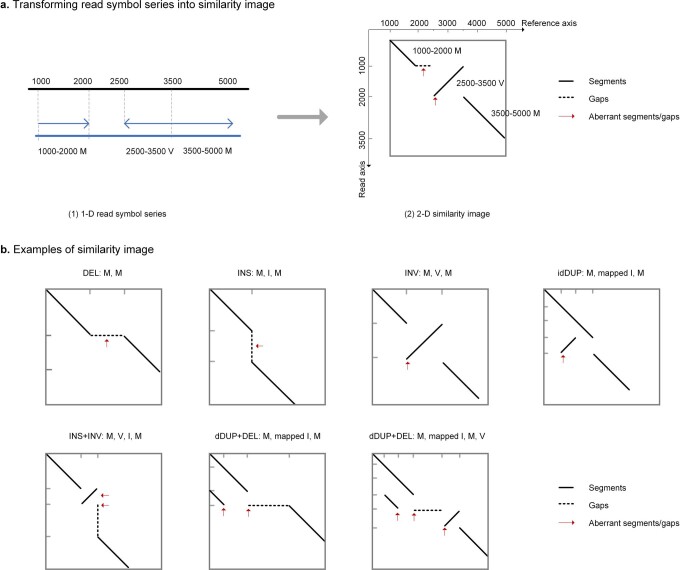
Illustration of the structure sketching step in SVision-pro. **a**, SVision-pro directly transforms the 1-dimensional symbol series into a 2-dimensional similarly image, which utilizes segments and gaps to sketch the structure of the SV. Segments, derived from symbol ‘M’ and ‘V’, are represented in solid lines while gaps, derived from symbol ‘I’, are represented in dash lines. Gaps along with segments converted from symbol ‘V’ are mark with an aberrant flag (red arrows) for subsequence process. **b**, Several examples for transforming symbol series that span SSVs or CSVs, into similarity images.

**Extended Data Fig. 3:**
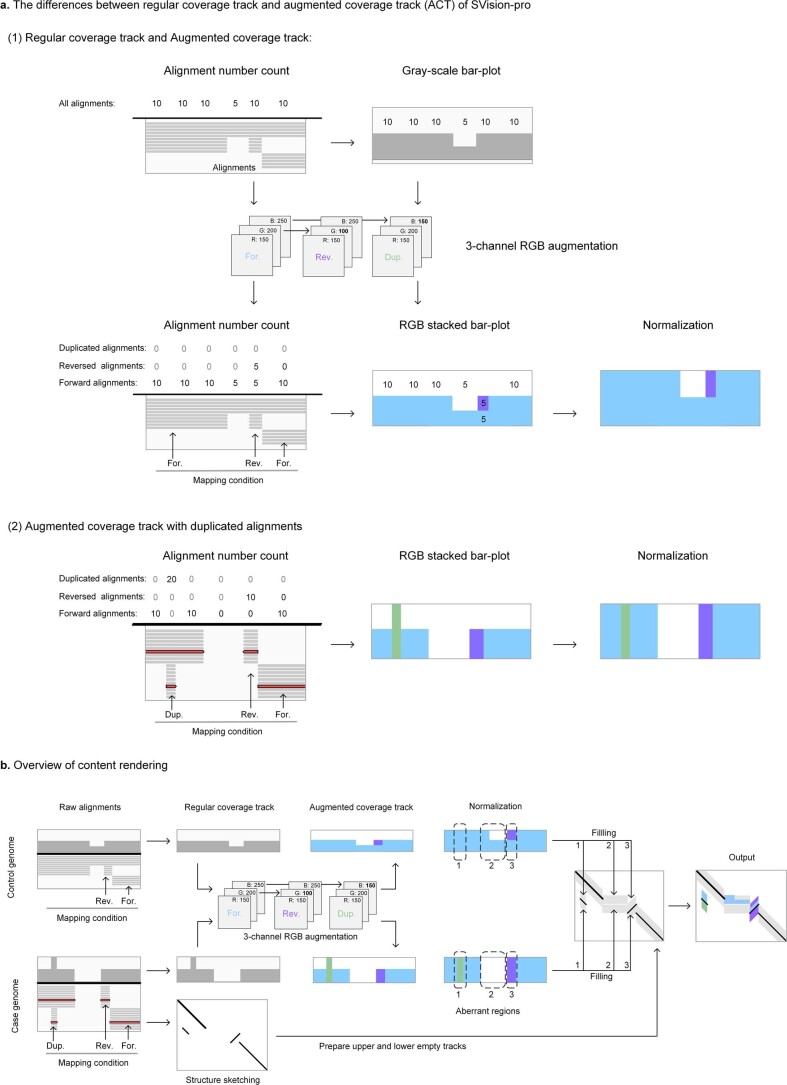
Illustration of the content rendering step in SVision-pro. **a**, Comparison of regular coverage track and the augmented coverage track (ACT) in SVision-pro. The ACTs are generated by 3-channel RGB augmentation. SVision-pro counts read alignments according to their mapping conditions and generates a RGB stacked bar-plot, where different mapping conditions are represented in their respective RGB colors. **b**, Overview of the content rendering step. For both control and case genomes, the ACTs are generated, normalized, and further filled into the upper/lower tracks around aberrant segments and gaps in the similarity. Abbreviations: ‘Dup.’ denotes duplicated mapping; ‘Rev.’ denotes reversed mapping; ‘For.’ denotes forward mapping.

The sequence-to-image representation module then compares two genomes (termed as case and control genome) in two steps (Fig. 1a): structure sketching and content rendering. For an aberrant locus in the case genome (for example, from child or tumor tissue), the structure sketching step directly transforms the 1D read symbol series into a two-dimensional (2D) similarity image (Extended Data Fig. 2a), which uses segments and gaps to measure the structural similarity of the reference sequence and the variant feature sequence from the case genome in an image (Extended Data Fig. 2b). The content rendering step (Methods) fills the sparse image regions with augmented coverage tracks (ACTs), which represent genomic differences between the case and control genome. First, we color the raw coverage track according to the forward-, inverted- and duplicated-matching conditions of alignments in three image channels (Fig.1a and Extended Data Fig. 3a). Then, we use a fixed-height track above these structures (upper track) to encode the normalized ACT from the control genome (for example, from parent samples or normal tissue) while the track below (lower track) encodes ACT from the case genome (Extended Data Fig. 3b). This representation strategy facilitates genome-to-genome comparison, simultaneously encoding both SV structures (via segments and gaps) and their intergenome differences (via contrasting ACTs in lower and upper tracks), thereby requiring a multitask neural-network framework that can perform the detection and genotyping tasks simultaneously.

**Extended Data Fig. 4:**
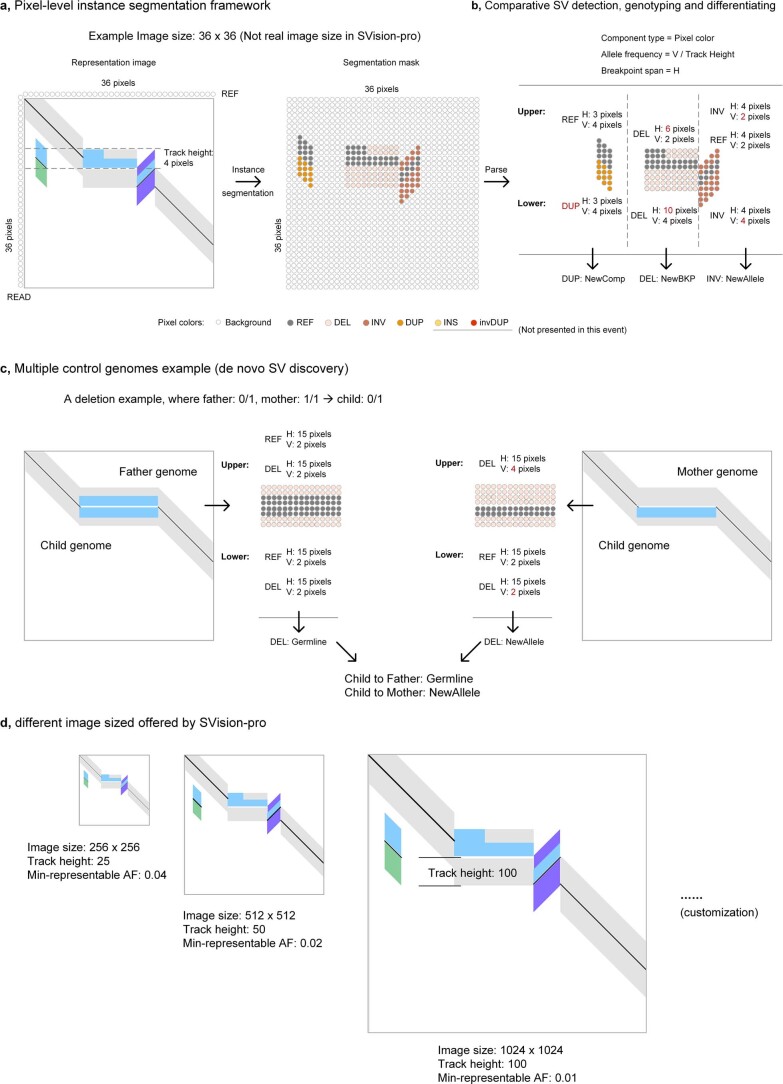
Illustration of the image instance segmentation framework in SVision-pro. **a**, At the pixel level, the segmentation process predicts each image pixel as either belonging to the background or a specific variant type in the segmentation mask image **b**, The segmentation mask provides obvious comparison in SV subcomponent type, breakpoint, and allele frequency (AF) by contrasting the lower and upper track. Mask color comparison indicates the differences in SV subcomponent type. Horizontal comparison indicates the differences in SV subcomponent breakpoint span. Vertical comparison indicated the differences in SV subcomponent AF. Consequently, SVision-pro outputs four distinct comparison types to depict the SV difference between the case genome and the control genome, including germline, new components, new breakpoints and new alleles. **c**, In the scenarios where multiple control genomes are provided (such as the parent genomes in de no SV discovery), the instance segmentation framework predicts each image and outputs the SV difference between case genome and each control genome. Abbreviation: ‘NewComp’ for new component; ‘NewBKP’ for new breakpoint; ‘NewAllele’ for new allele frequency. **d**, SVision-pro currently provides three different image sizes. Larger image sizes lead to larger track heights, and thereby lower minimum representable allele frequencies (AFs). Moreover, the properties of the representation image, such as image size, track height and colors, can be customized for user-specific applications.

We integrated those many tasks into a one-stop neural-network-based image instance segmentation framework instead of utilizing several deep-learning classification modules (Fig. 1b and Extended Data Fig. 4; Methods). Briefly, this framework takes in an encoded image and generates a pixel-level segmentation mask, classifying image areas in the upper and lower tracks into five basic SV component classes (Fig. 1b and Extended Data Fig. 4a), and one wild-type reference (REF). The other image regions, such as the flanking sequence encoding region, were classified as Background. SV types are predicted directly by joining components together in both the case and control tracks. Moreover, this instance segmentation framework enables a three-task comparison of SV component types, breakpoints and allele frequencies (AFs) between the case and control genomes (Fig. 1b). Specifically, for each SV component in the segmentation mask, the horizontal span of the masked pixels represents its breakpoint span, while the vertical span represents its AF (Extended Data Fig. 4b). Apart from the widely used genotyping tags (1/1, 0/1 and 0/0) derived from AF, SVision-pro generated four distinct categories by contrasting each SV component presented in the case genome with that of the control genome (Extended Data Fig. 4b; Methods). These categories are: (1) ‘Germline,’ indicating the presence of the SV subcomponent in the control genome with the same allele frequency as that of the case; (2) ‘New component,’ indicating the absence of the SV subcomponent in the control genome; (3) ‘New breakpoint,’ indicating the presence of the SV subcomponent in the control genome but with a different breakpoint span to the case and (4) ‘New alleles,’ indicating the presence of the SV subcomponent in the control genome but with a different AF to the case. In the scenarios for de novo SV discovery, SVision-pro will output the differences between the case genome and each control genome (Extended Data Fig. 4c). SVision-pro offers flexible image properties for different sensitivity requirements. Currently, SVision-pro enables a minimum detection AF of 0.01. Larger image sizes result in lower minimum representable and detectable AFs (Extended Data Fig. 4d; Methods).

**Extended Data Fig. 5:**
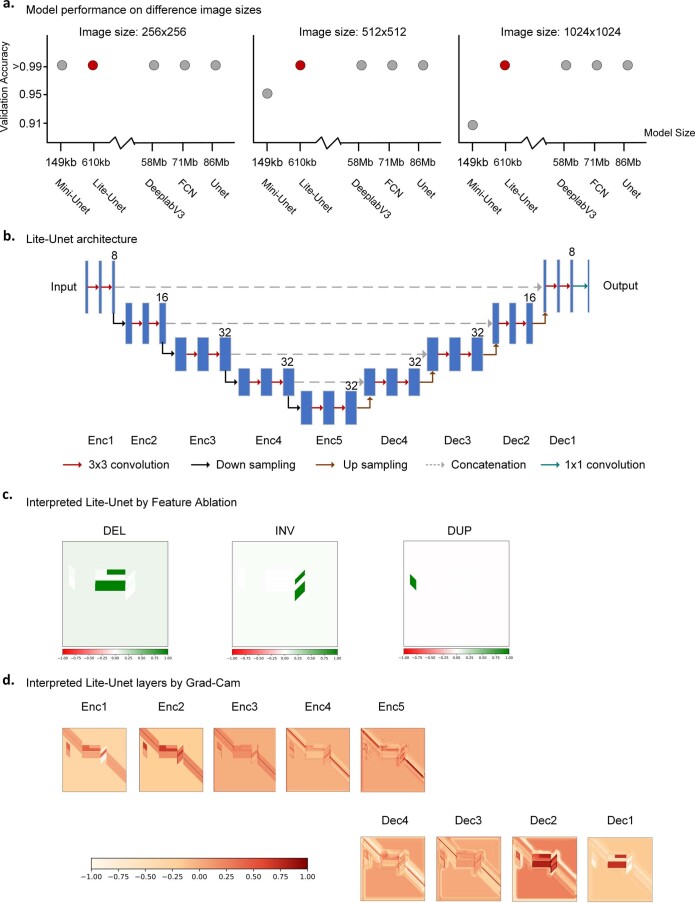
Comparison and interpretation of the neural-network-based instance segmentation frameworks. **a**, Comparison of the accuracy (y-axis) on validation dataset among the five models (x-axis). The models are arranged based on their parameter sizes. **b**, the network architecture of the default Lite-Unet model. **c**, A heatmap to illustrate the Feature Ablation interpretation of the Lite-Unet model. Positives values (in green) indicates positive attrition to the specific prediction while negative values are shown in red. **d**, Using Grad-Cam to generate attribution maps of each layer in Lite-Unet.

To identify an appropriate instance segmentation model (Fig. 1c), five well-known models of different parameter sizes, including Unet^21^, Fully-Convolutional-Network^22^, Deeplab v.3 (ref. ^23^), Lite-Unet and mini-Unet were trained and compared on simulated data (Supplementary Note 1). The default model, Lite-Unet, achieved a balance between accuracy and model size (Extended Data Fig. 5a,b) while also exhibiting strong model interpretability (Fig. 1d and Extended Data Fig. 5c,d).

We benchmarked the performance of SVision-pro and other approaches using both simulated and publicly available datasets (Supplementary Table 1), covering high-fidelity (HiFi), Oxford nanopore (ONT) and continuous long reads (CLR). The computational resource usages were assessed on both a personal computer and a cluster node (Supplementary Note 2 and Supplementary Table 14).

**Extended Data Fig. 6:**
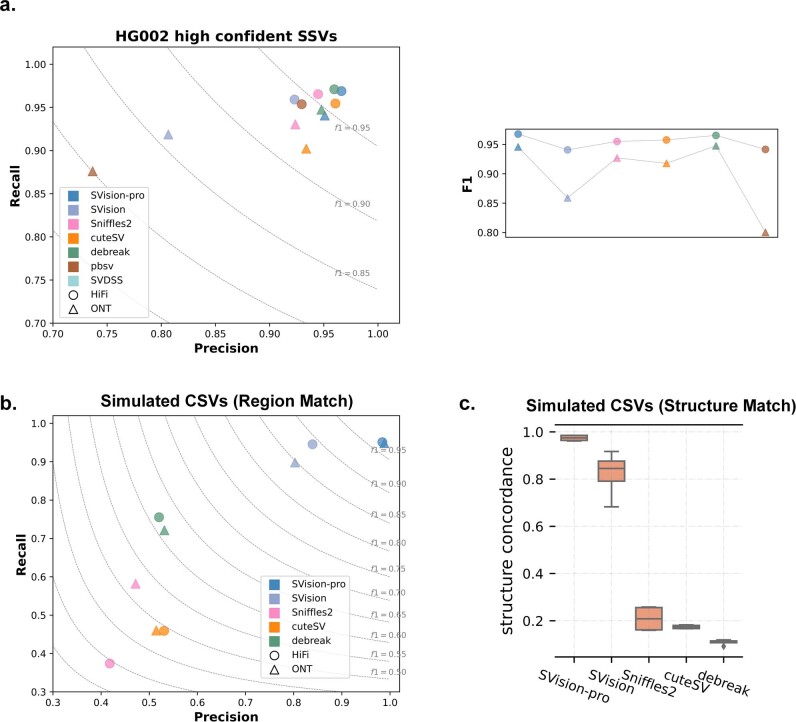
Performance evaluation of SSV and CSV calling among callers. **a**, SSV detection performance on HG002 groundtruth HiFi and ONT dataset. Recall, precision and F1-score were compared among callers. **b**, CSV detection performance on simulated 3,000 CSV HiFi and ONT dataset. Five of the highest-performing callers at SSV detection were chosen for a CSV performance comparison. Since only SVision-pro and SVision were equipped with CSV characterization ability, we utilized the region matching strategy to avoid the comparison of CSV types. **c**, CSV structure concordance evaluation among callers. Each box contains four values (Supplementary Table 3). The boxplot defines the median (Q2, 50th percentile), first quartile (Q1, 25th percentile) and third quartile (Q3, 75th percentile). The bounds of box, that is interquartile range (IQR), of the boxplot is between Q1 and Q3. The minima and maxima values are defined as Q1-1.5*IQR and Q3 + 1.5*IQR, respectively. The whiskers are values between minima and Q1 as well as between Q3 and maxima. Values falling outside the Q1 – Q3 range are plotted as outliers of the data.

SVision-pro outperformed other callers on HG002 groundtruth SSVs and simulated CSVs (Extended Data Fig. 6a,b and Supplementary Table 2; Methods). Moreover, SVision-pro achieved 96–98% accuracy in CSV subcomponent accuracy (Extended Data Fig. 6c and Supplementary Table 3; Methods), improving, on average, 15% compared with SVision—the state-of-the-art CSV caller. Further experimental validations (Supplementary Table 4, Supplementary File 1 and Supplementary Note 3) supported that SVision-pro has high sensitivity and a low false-positive rate for CSV detection.

**Fig. 2:**
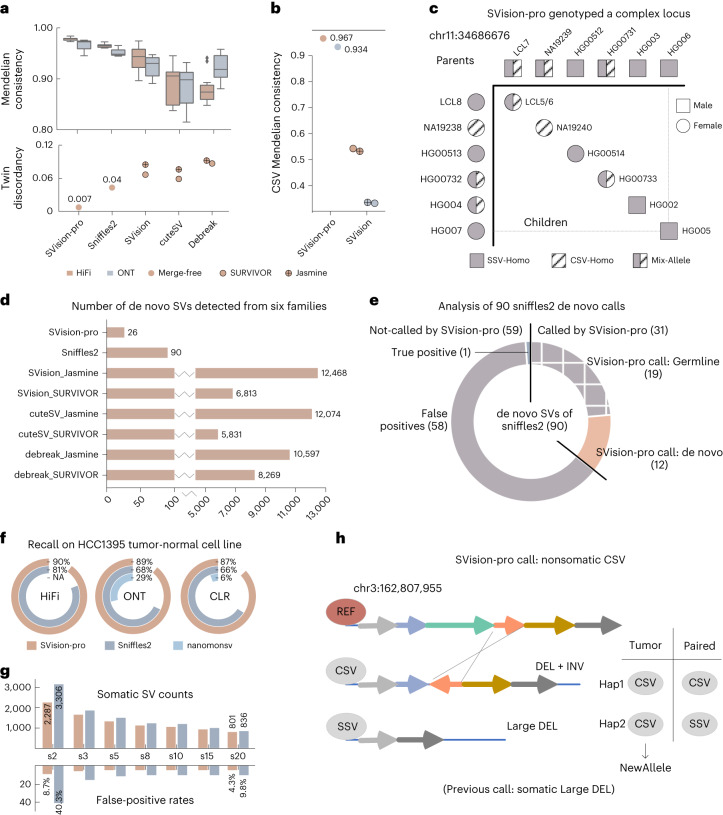
Performance comparison. **a**, Comparison of the Mendelian consistency in six family datasets (above) and the twin discordancy in the ChineseQuartet (below). SVision-pro is compared with Sniffles2 (multisample mode) and SVision, cuteSV and debreak (followed by SURVIVOR and Jasmine merging). Each box contains six and three values for HiFi and ONT, respectively (Supplementary Table 5). The boxplot defines the median (Q2, 50th percentile), first quartile (Q1, 25th percentile) and third quartile (Q3, 75th percentile). The bounds of the boxplot, representing interquartile range (IQR), are between Q1 and Q3. The minimum and maximum values are defined as Q1 − 1.5× IQR and Q3 + 1.5× IQR, respectively. The whiskers are values between minima and Q1 and between Q3 and maxima. Values falling outside the Q1–Q3 range are plotted as outliers of the data. **b**, Comparison of the CSV Mendelian genotype consistency on the simulated trio data. SVision-pro was compared with state-of-the-art CSV caller SVision (followed by SURVIVOR and Jasmine merge). **c**, In the six families, SVision-pro correctly genotyped a complex locus comprising both an SSV and a CSV. Three distinct alleles are found by SVision-pro, including homologous SSV, homologous CSV and mixed heterozygous SSV and CSV. **d**, Comparison of the number of de novo calls in the six family datasets. **e**, Overlapping of 90 de novo calls produced by Sniffles2 with all calls produced by SVision-pro. **f**, Recall values on the previously published somatic SV callset of HCC1395 tumor-normal paired cell lines. **g**, The number of somatic SVs and the false-positive rates produced by Vapor validation decrease as the supporting read number increases. **h**, SVision-pro identified a nonsomatic complex locus that had been reported as a somatic SSV. SVision-pro revealed that the paired normal genome exhibited a heterozygous SSV and CSV, whereas the tumor genome exhibited homozygous CSV.

**Extended Data Fig. 7:**
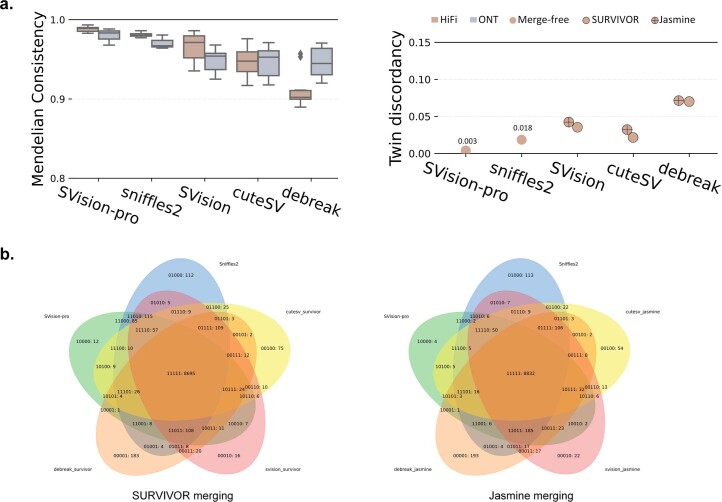
Performance evaluation of Mendelian sample calling within high-confidence regions. **a**, In high-confidence regions, comparison of the Mendelian consistency in six family datasets (left) and the twin discordancy in the ChineseQuartet (right). SVision-pro is compared to Sniffles2 (multi-sample mode) and SVision, cuteSV and debreak (followed by SURVIVOR and Jasmine merging). Each box contains six and three values for HiFi and ONT, respectively (Supplementary Table 5). The boxplot defines the median (Q2, 50th percentile), first quartile (Q1, 25th percentile) and third quartile (Q3, 75th percentile). The bounds of box, that is interquartile range (IQR), of the boxplot is between Q1 and Q3. The minima and maxima values are defined as Q1-1.5*IQR and Q3 + 1.5*IQR, respectively. The whiskers are values between minima and Q1 as well as between Q3 and maxima. Values falling outside the Q1 – Q3 range are plotted as outliers of the data. **b**, Venn diagrams show the overlapping results of high-confidence calls among approaches. We overlapped these high-confidence calls from each approach in AshkenazimTrio. there were only several unique calls (n = 12 and 4 when overlapping with SURVIVOR and Jasmine, respectively) from SVision-pro (9,348 in total), indicating that the leading consistency in Mendelian samples was attribute to the higher genotyping accuracy of SVision-pro compared to merging approaches.

We next compared SVision-pro with callset-merge strategies on six families, including a ChineseQuartet^24^ (Methods). SVision-pro achieved the highest Mendelian consistency (97.3–98.4% on HiFi reads and 94.5%-97.6% on ONT reads) and the lowest discordancy (0.7%) between monozygotic twins (Fig. 2a and Supplementary Tables 5 and 6; Methods). When restricted to high-confidence regions (Methods), SVision-pro continued to outperform other approaches: the Mendelian consistency improved to 98.4–99.3% and 96.8–98.8% for HiFi and ONT, respectively, and the twin discordancy decreased to 0.3% (Supplementary Tables 5 and 6 and Extended Data Fig. 7). On a simulated trio harboring de novo/inherited CSVs (Supplementary Note 4), SVision-pro achieved 96.6% and 93.3% Mendelian genotype accuracy on HiFi and ONT long reads, respectively, while the second-best approach, SVision (followed by Jasmine merging), achieved 53.2% and 33.5% (Fig. 2b and Supplementary Table 7).

**Extended Data Fig. 8:**
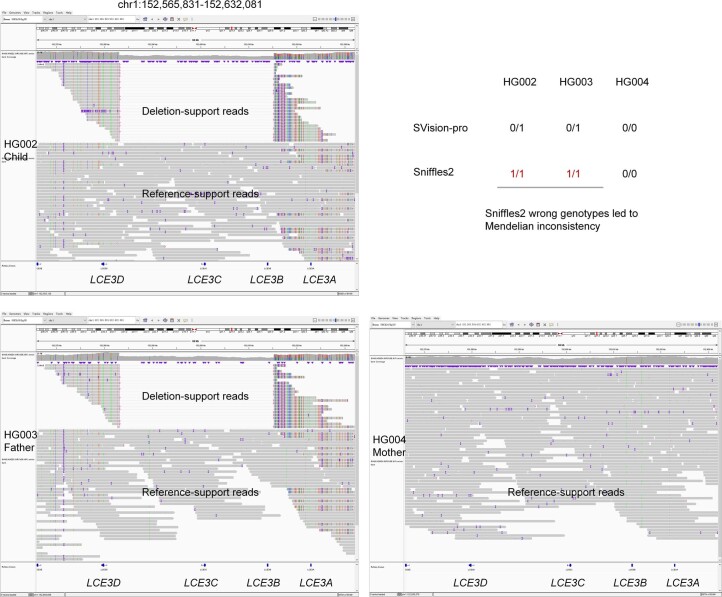
IGV screenshot of the 32,549 bp deletion in chromosome 1. The Ashkenazim Trio (HG002, HG003 and HG004) from GIAB was used to illustrate the various genotypes of this deletion. Sniffles calculated incorrect genotypes in this trio, leading to mendelian inconsistency. SVision-pro correctly genotyped this locus in the trio dataset, revealing that both the child genome (HG002) and the father genome (HG003) exhibited a heterozygous deletion, while the mother genome (HG004) contained no SV in this locus.

**Extended Data Fig. 9:**
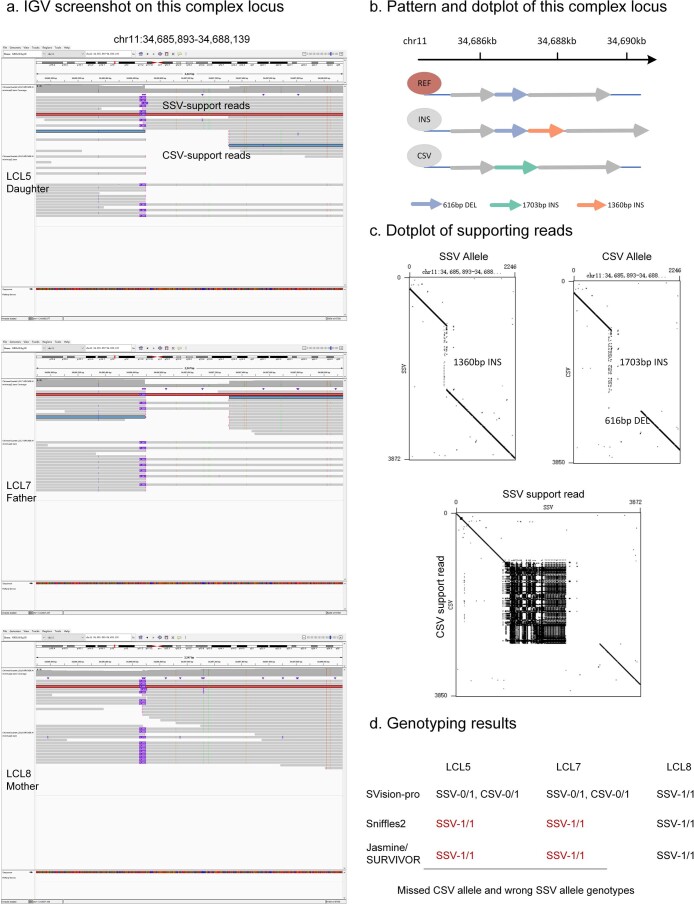
Illustration of the complex locus in chromosome 11. **a**, IGV screenshot on this complex locus in the ChineseQuartet. This complex locus comprised of two alleles, including one SSV deletion and one CSV deletion-insertion. Read that supported the SSV allele was marked in red while read that supported the CSV allele was marked in blue. **b**, The summarized pattern at this complex locus. **c**, Gepard Dotplots^36^ were used to show the differences between the SSV allele and CSV allele. **d**, SVision-pro correctly genotyped the two alleles, outputting the correct genotype of each allele. Sniffles2 and callset-merging strategies missed the CSV allele and incorrectly genotyped the SSV allele as homozygous in the child and father genome.

The high genotyping accuracy of SVision-pro led to reliable discoveries in Mendelian samples. For instance, a 32,549 bp deletion, encompassing the genes *LCE3B* and *LCL3C* and associated with increased risk of psoriasis^25,26^, was incorrectly genotyped by Sniffles2 (ref. ^15^) yet was correctly genotyped by SVision-pro in the six families (Extended Data Fig. 8 and Supplementary File 2). Another complex locus, which was mis-called by all other approaches, comprised two SV alleles: an SSV (insertion) and an CSV (insertion–deletion) (Extended Data Fig. 9a–c). SVision-pro correctly genotyped these two alleles (Fig. 2c and Extended Data Fig. 9d), consistent with visual verification on HiFi reads and published assemblies (Supplementary File 3).

In the six families, SVision-pro reported 26 de novo SVs, including 13 insertions and 13 deletions (Supplementary Table 8), all of which were validated manually (Supplementary File 4). LRS enabled the discovery of a larger proportion of de novo insertions compared with SRS, and further annotation of the reported de novo SVs revealed that 20 of them featured repeat expansions or contractions (Supplementary Table 8). By contrast, Sniffles2 reported 90 whereas Jasmine/SURVIVOR reported many more redundant calls: 5,831–12,468 de novo SVs in total (Fig. 2d). We overlapped these 90 de novo calls of Sniffles2 with SVision-pro (Fig. 2e and Supplementary Table 9): among the 59 nonoverlapping calls, only one true-positive de novo SV was confirmed by manual inspection. Of the remaining 31 overlapped calls, 19 were identified as germline by both SVision-pro and manual curation (Supplementary File 5), indicating that they are false positives. Additional experimental validations (Supplementary Note 3, Supplementary Files 6 and 7 and Supplementary Table 10) further supported that SVision-pro effectively reduced false-positive calls in Mendelian samples and reported high-quality de novo SVs.

**Extended Data Fig. 10:**
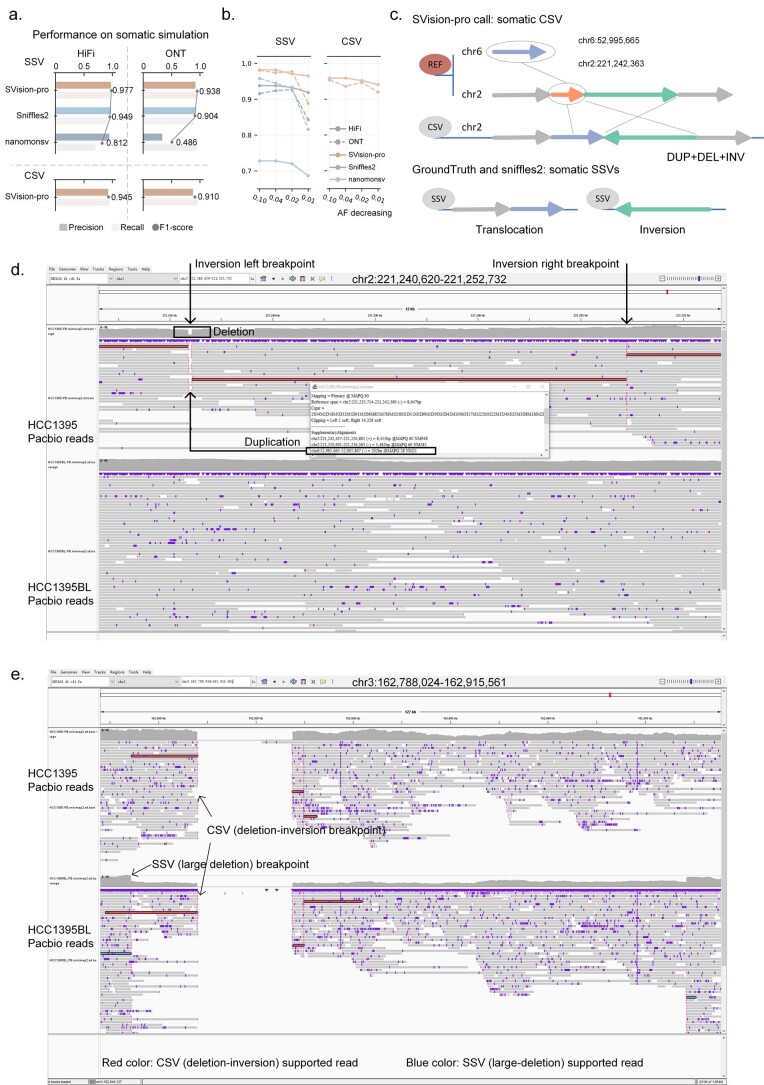
Somatic detection evaluation and discovery. **a**, The Precision, Recall, and F1-score of SVision-pro, Sniffles2, and nanomonsv on the simulated somatic SSVs and CSVs. **b**, The recall values of various low-frequency SSVs and CSVs in the simulation. **c**, A somatic CSV locus in chromosome 2 of HCC1395 cell line. SVision-pro reported this locus as somatic CSV, dispersed duplication-deletion-inversion, while in the previous published somatic SV set, the deletion component was missed and the dispersed duplication component was classified into translocation. **d**, IGV screenshot supported the CSV outputted by SVision-pro. **e**, IGV screenshot supported the homozygous CSV in the tumor genome and heterozygous SSV and CSV in the paired normal genome. The SSV large deletion breakpoint present in the paired normal genome while absent from the tumor genome.

To assess the somatic detection performance, we simulated a subclonal tumor genome, which harbored somatic SSVs and CSVs with AFs ranges from 0.01 to 0.10 (Supplementary Note 4). For SSVs, the F1-scores of SVision-pro were 0.98 (HiFi) and 0.94 (ONT), leading the other two somatic-capable callers, Sniffles2 and nanomonsv^16^, by 0.03 to 0.45 (Extended Data Fig. 10a). For CSVs, the F1-scores were 0.95 and 0.91. As expected, as the AF decreased, the detection accuracy exhibited a decreasing trend (Extended Data Fig. 10b). Nevertheless, for somatic SSVs and CSVs with AF = 0.01, SVision-pro still achieved average accuracies of 95.3% and 90.4% on HiFi and ONT reads (Supplementary Table 11). SVision-pro maintained consistent high-performance with various numbers of simulated events and coverages (Supplementary Table 12).

We next assessed SVision-pro using normal-tumor paired cell lines, HCC1395 and HCC1395BL, across three sequencing technologies, including HiFi, ONT and CLR (Methods). SVision-pro detected 87–90% of the published somatic SSV loci^27^, while Sniffles2 detected 66–81% and nanomonsv detected 6–29% (Fig. 2f). Through computational validation using Vapor^28^ on the detected somatic calls, SVision-pro demonstrated a much lower false-positive rate (4.3–8.7%; Fig. 2g, Supplementary Table 13 and Supplementary Note 5) compared with Sniffles2 (9.8–40.3%). Taken together, these results show that SVision-pro detects somatic SVs with higher sensitivity and lower false-positive rates compared with Sniffles2 and nanomonsv^16^.

Moreover, SVision-pro resolved eight CSVs that were previously reported as SSVs (Supplementary File 8; Methods), including a dispersed duplication-deletion-inversion where the deletion component was missed and the dispersed duplication component was classified as a translocation (Extended Data Fig. 10c,d). SVision-pro also identified a nonsomatic complex locus, which was previously reported as a somatic SSV (Fig. 2h). SVision-pro revealed that the paired normal genome comprised one SSV allele and one CSV allele (deletion-inversion), whereas the tumor genome lost the SSV allele and acquired a homozygous CSV (Extended Data Fig. 10e).

In summary, SVision-pro is an accurate and interpretable approach for comparative SV detection and genotyping, addressing the challenges in de novo and somatic SV discovery from long-read data. SVision-pro visually compares genomic features encoded from sequencing alignments, and so avoids the error-prone merging process intrinsic to a callset-level strategy, hence resulting in high-quality calls. The instance segmentation framework removes the requirement for prebuilding inference models for SV types, thereby providing high CSV resolution. We conducted experimental validation for the findings of SVision-pro, in which certain events were deemed inconclusive due to PCR failure, characterized by the absence of notable PCR band or the presence of noisy PCR bands. This ambiguity raises the possibility that these events could be false positives, necessitating an orthogonal technique capable of validating SVs identified by LRS. Future work would develop merging- and model-free approaches for population-scale SV characterization to further improve discovery of the human SV spectrum.

## Methods

### SVision-pro methodology

#### Overall workflow of SVision-pro

SVision-pro initiates by searching the case genome for candidate SV loci, after which a sequence-to-image module encodes genome-to-genome image to visually compare the case and control genomes. Then, the neural-network-based instance segmentation framework recognizes basic SV component types from the encoded image and determines the genomic differences between the case genome and the control genome. Note that, if several control genomes (*N* and *N* > 1) are specified, SVision-pro works in a 1-to-*N* mode and generates representation images for the case genome and each control genome. Consequently, the instance segmentation framework outputs the SV differences between the case genome and each control genome.

#### Candidate SV locus searching from case genome

SVision-pro identifies candidate SV loci by collecting and clustering abnormal read alignments in a model-free way that avoids searching for specific aberrant patterns of read alignments (Extended Data Fig. 1). Specifically, SVision-pro converts each read into a series of signature symbols, which can be extracted directly from a BAM file: M indicates directly mapping of alignment to the reference genome, V indicates reversed mapping and I indicates an additional sequence in read. Moreover, several properties are allocated to each signature symbol, including its span on the reference sequence, span on the read sequence, subsequence length and read name. Typically, symbols M and V are converted from split read alignments (primary and supplementary alignments) according to their reference span (reference start and end position) and mapping orientation. The symbol I is derived from both intraread alignments, by examining the CIGAR string, and inter-read alignments, by retrieving unmapped sequence between split alignments (Extended Data Fig. 1a). Note that for I, if the unmapped sequence is aligned to a distal location on the reference sequence, SVision-pro marks it as a mapped I by recoding the additional source reference span. Finally, each read is converted into a series of symbols arranged in their read order. For example, if a read does not span any SVs, there will be only one symbol M (Extended Data Fig. 1b). If a read spans a deletion, the read will be converted into symbol series MM, where there is a gap between the reference end position of the first M and the reference start position of the last M (Extended Data Fig. 1c). For complex events, such as a deletion associated with an inversion, the event-supporting read is converted into symbol series MVM (Extended Data Fig. 1d). By adopting this convention, we are able to cluster similar read symbol series iteratively and identify any abnormal ones (Extended Data Fig. 1e). A read with the converted symbol series M is considered a normal read, otherwise, it will be marked as an aberrant one. If the number of reads supporting the same aberrant symbol series surpasses the minimum requirement (default ten reads), the genomic region covered by the aberrant symbol series is considered a candidate SV locus.

#### Image representation at candidate SV loci

To generate representation images, SVision-pro takes two main steps: structure sketching (Extended Data Fig. 2) and content rendering (Extended Data Fig. 3).

1. Structure sketching: for a candidate SV locus, the structure sketching step directly converts the 1D read symbol series into a 2D similarity image (Extended Data Fig. 2a), which uses segments and gaps to visually measure the mapping similarity between reference sequence (*x* axis) against variant feature sequence (*y* axis). The reference axis ranges from the start reference position of the first symbol to the end reference position of the last symbol. The read axis ranges from 0 to the length of the read. Typically, segments are derived from symbols M, V and mapped I, whereas gaps are derived from the unmapped symbol I and reference gaps between M and V symbols. Segments and gaps, excluding those converted from M symbols, are marked with aberrant flags for subsequent content rendering step (Extended Data Fig. 2b). This type of similarity image makes it easy for humans and machines to visualize SV structures.
2. Content rendering: SVision-pro fills the sparse region in the similarity image with ACTs originated from both case and control genomes.

#### Generating ACTs

Inspired by the regular coverage track commonly used in Integrative Genomics Viewer (IGV)^29^, SVision-pro introduces the ACT. In brief, the regular coverage track is a 2D grayscale barplot, where the *x* axis indicates reference positions and *y* axis indicates the coverage values, which are computed by counting the number of mapped alignments at each reference position (Extended Data Fig. 3a). The ACT in SVision-pro utilizes an RGB (red, green and blue) stacked barplot to encode additional genomic information that reflects SV signatures. Before constructing the ACT (Extended Data Fig. 3a), we count the number of alignments along with their mapping conditions. The mapping conditions of alignments include forward mapping, reversed mapping, duplicated mapping and reverse-duplicated mapping. Forward and reversed mapping conditions are retrieved directly from the aligner’s outputs and duplicated mapping is determined by checking whether an alignment is encompassed by other alignments from the same read (Extended Data Fig. 3a).

Next, we convert the count table into a three-channel RGB image. We use the RGB color values (135, 206, 255) to plot the coverage value of forward-mapped alignments. For the coverage value of reversed alignments, we subtract 100 from the color value in the second channel (Supplementary Fig. 1a). Likewise, for the coverage value of duplicated alignments, we subtract 100 from the color value in the third channel (Supplementary Fig. 1b). In cases of reverse-duplicated alignments, both the second and third channels undergo a subtraction of 100 (Supplementary Fig. 1c). In brief, we use the second image channel to depict the reverse signatures and the third image channel to depict the duplication signatures. By leveraging this RGB stacked barplot in the ACT, SVision-pro provides a more comprehensive representation of the coverage information, incorporating distinct color variations to depict different types of alignments and their contribution to the SV signature.

#### Filling ACTs into similarity image

Genome-to-genome comparison requires comparative representation features to contrast the SV differences between the case genome and the control genome. Therefore, we utilize the sparse regions within the similarity image to fill the two ACTs originating from the case and control genomes (Extended Data Fig. 3b). To accomplish this, we first create two fixed-height and empty tracks along these sketched segments and gaps: one track (upper track) above and one track below (lower track). The upper track is used to fill the ACT of the control genome whereas the lower track is used to fill the ACT of the case genome. For a sketched similarity image i, we generate ACTs in both case and control genomes by fetching all read alignments from i.reference_start to i.reference_end. This ensures that the reference span of the sketched similarity image matches that of the ACTs. Next, we fill ACTs into upper/lower tracks that surround aberrant segments and gaps by aligning the reference coordinates. Contrasting ACTs in upper and lower tracks show apparent SV differences between the case and control genomes. Moreover, this kind of similarity image and ACTs maintains readability for both human and machines for further analysis.

#### Insertion-associated SV representation

Insertions and insertion-related SVs involve additional sequence present in the read sequence that is not in the reference sequence, leading to vertical gaps in the sketched similarity images (Supplementary Fig. 2a). Therefore, for insertions, we create two empty tracks located on the left (used to fill the ACT of the control genome) and right (used to fill the ACT of the case genome) sides of these insertion-induced vertical gaps (Supplementary Fig. 2b). Unlike deletions, inversion and duplications, where we count the alignment mapping conditions against the reference genome, for insertions, we count the alignments at read-level to calculate the number of reads that contain the inserted sequence (Supplementary Fig. 2c). Then, we generate vertical ACTs for both case genome and control genome and fill them into the right and left empty tracks, respectively. For insertion-associated CSVs, such as insertion-associated inversion, alignments are counted at both read-level and reference-level (Supplementary Fig. 2d).

#### One-to-*N* mode

The genome-to-genome representation module in SVision-pro allows for the comparison of one case genome with one control genome within a single image. However, in certain applications, such as de novo SV discovery, several control genomes are involved. To accommodate such scenarios, SVision-pro employs a One-to-*N* mode to generate images between case genome and each control genome. For example, de novo SV discovery in a trio comprises three genomes: child, father and mother. For a candidate SV locus, SVision-pro generates one image that compares the child genome with the father genome, and another that compares the child genome with the mother genome. This process results in two images that can be utilized by the subsequent instance segmentation framework for further analysis. By employing the One-to-*N* mode, SVision-pro enables direct comparison of the case genome with several control genomes. Moreover, SVision-pro can identify any genome-specific SVs among several genomes by taking one genome as the case genome and all others as control genomes.

#### Flexible properties of representation image

The image sizes, colors and track heights are flexible and can be customized to meet various application scenarios. Currently, SVision-pro offers three optional image sizes for different sensitivity requirements, including 256, 512 and 1,024, whose track height for rendering contents is 25, 50 and 100 pixels, respectively. Thereby, the minimum representable (1 pixel) and detectable AFs (one per track height) of the three image sizes are 0.04, 0.02 and 0.01, respectively. Note that AF 0.01 is not the lowest detection limit of SVision-pro, and that the track heights and images sizes can be customized to meet lower AF detection requirements.

#### SV detection and genotyping by instance segmentation

The encoded representation images are directly fed into a neural-network-based instance segmentation framework without any manual or knowledge-oriented preprocessing. Since CSVs typically comprise several internal subcomponents, the instance segmentation framework in SVision-pro is designed to recognize five basic subcomponent types, including insertion (INS), deletion (DEL), inversion (INV), duplication (DUP) and inverted duplication (invDUP). In cases where there is no SV present in the control genome, a recognition type reference (REF) is included to denote that the control genome is identical to the reference genome. Specifically, the instance segmentation framework recognizes these six instance types in the encoded image and generates a segmentation mask. The mask assigns each pixel in the image to either a predicted specific type or the background type, segmenting the image regions and providing quantitative information about the presence and location of various SV subcomponents (Extended Data Fig. 4a). The horizontal span of the masked regions represents the breakpoint span of the subcomponents, while the vertical span represents the allele frequency (Extended Data Fig. 4b). Finally, in respective panels, we obtain the final SV type of the candidate locus by directly jointing together these subcomponents in their read order. By contrasting the lower and upper panels in the segmentation mask image, SVision-pro can determine whether a SV subcomponent is (Extended Data Fig. 4b) Germline, indicating that the SV subcomponent is present in the control genome with same allele frequency; (2) New allele, indicating that the SV subcomponent is present in the control genome at a different allele frequency; (3) New component, indicating that the SV subcomponent is absent from the control genome or (4) New breakpoint, indicating that the SV subcomponent is present in the control genome with a different breakpoint span. If several control genomes are provided, such as the father and mother genome in the scenarios for de novo SV discovery, SVision-pro will output the differences between the case genome and each control genome (Extended Data Fig. 4c).

### Performance benchmarking methodology

#### SSV detection benchmark in HG002 groundtruth

The groundtruth SSVs (HG002_SVs_Tier1_v0.6.vcf.gz, highly confident insertions and deletions) of HG002 (Ashkenazim Trio, son), were applied to benchmark the SSV detection performance of callers. The detailed data generation steps were identical to those described in cuteSV^3^ paper. Briefly, both raw HiFi and ONT reads were aligned to human genome GRCh37 using Minimap2 (ref. ^30^) with parameter ‘-x pacbio/ont’. Seven state-of-the-art callers, including SVision-pro, SVision^6^, Sniffles2 (ref. ^15^), cuteSV^3^, debreak^4^, pbsv and SVDSS^5^, were applied to the aligned reads with the minimum SV supporting read number set to ten. Truvari^31^ was employed to calculate precision, recall and F1-score between the groundtruth and the callset. Please refer to Supplementary Note 6 for the specific versions and parameters of each caller.

#### CSV detection benchmark in simulated data

The CSV simulation set, which contains 3,000 CSVs crossing ten frequently reported types, was obtained directly from our previous SVision paper^6^. We followed the same procedure described in this paper to generate both HiFi and ONT reads and performed subsequent alignment to GRCh38 by NGMLR^2^. The five highest-performing callers on the HG002 groundtruth dataset (SVision-pro, SVision, Sniffles2, cuteSV and debreak) were employed for the subsequent Truvari region-based comparison. Type-based comparison was performed by examining the CSV subcomponent accuracy. To accomplish this (Supplementary Fig. 3a), we first extracted the matched SV record pairs between the groundtruth and callset from Truvari output files, namely TP-base.vcf and TP-call.vcf, which respectively enumerated the groundtruth record and matched callset record, respectively. Then, for each matched record pair, if any SV component from the groundtruth record was absent from the called record, this record pair was marked as inaccurate (Supplementary Fig. 3b). Note that, only SVision-pro and SVision reported SV component types. For the remaining callers, since they only reported SSVs and limited number of CSV types, we treated their output type directly as a component type.

#### Mendelian consistency analysis in six families

We collected 19 Mendelian samples from six previously published families, including the Ashkenazim Trio, Chinese Trio, YRI Trio, CHS Trio, PUR Trio and Chinese Quartet (Supplementary Table 1). All six families were sequenced using HiFi reads, with the Ashkenazim Trio, Chinese Trio and Chinese Quartet also sequenced with ONT reads. All reads were aligned to GRCh38 genome using Minimap2. We utilized five callers, including SVision-pro, SVision, Sniffles2, cuteSV and debreak, and two merging approaches, including Jasmine and SURVIVOR. For SVision-pro, we considered the child sample as the case genome and parent samples as control genomes. Sniffles2 was employed in multisample calling mode, following official instructions. For the remaining three callers that required merging approaches, we first applied them independently to generate callsets for each sample, including child(ren), father and mother. Then, we merged these callsets (for example, for ChineseQuartet, there were four callsets) together by Jasmine and SURVIVOR with the default or recommended parameters (Supplementary Note 2). To measure the Mendelian consistency within each family, we extracted the child and parent genotypes from each SV record in the VCF. If the genotypes of child, father and mother adhered to the Mendelian Law, we marked this record as a consistent one. Finally, we computed the Mendelian consistency rate by dividing the number of consistent records by the total number of records.

#### Twin discordancy analysis in Chinese Quartet

A common assumption is that the genomes of monozygotic twins are almost identical^32^. Therefore, the monozygotic twins (termed as child1 and child2) in the Chinese Quartet were used to calculate the twin discordancy. In brief, if one SV was present in the child1 genome while absent from the child2 genome, we would consider this SV as a discordant one between the twins. As such, for each SV record, we extracted the outputted genotypes of both child1 and child2 and examined whether they were identical. Finally, we computed the twin discordancy by dividing the number of discordant records by the total number of records.

#### De novo SV analysis in six families

For SVision-pro, de novo SVs were extracted by checking whether the comparison results of child-to-father and child-to-mother were both ‘New Component.’ For Sniffles2 and the merging approaches, de novo SV records were extracted by checking whether the SUPP_VEC equaled 100, indicating this SV record presented only in the child genome. Moreover, we compared the de novo SVs between SVision-pro and Sniffles2. De novo SV calls from Sniffles2 were overlapped with all SV calls from SVision-pro using the BEDtools^33^ intersect option with reciprocal overlap fraction set to 0.5. Since merging approaches resulted in many more redundant de novo SVs, we verified manually only the de novo SVs called by SVision-pro and Sniffles2 using IGV^29^ (Supplementary Files 4 and 5).

#### Somatic SV analysis in tumor-normal paired cell line HCC1395

A previous study^27^ utilized several sequence technologies and established a consensus somatic SV callset of 1,788 SVs on cell line HCC1395 and its normal pair HCC1395BL. We download the published HiFi, ONT and PacBio CLR long reads of the two cell lines and aligned them to human genome GRCh38 by Minimap2 with parameter ‘-x pacbio.’ Three callers that could detect somatic SVs were employed on this tumor-normal paired cell line, including SVision-pro, Sniffles2 and nanomonsv. SVision-pro took the tumor cell line as the case genome and normal cell line as the control genome. Sniffles2 was employed in its nongermline mode and nanomonsv was employed according to official instructions. For the three callers, the minimum number of supporting reads was set to 2 and the minimum detectable AF was set to 0.01.

#### High-confidence region filter

The raw high-confidence regions (HG002_SVs_Tier1_v0.6.bed) were hg19-based. Therefore, following the instruction of SVDSS paper^5^, we first used liftOver to convert these regions into hg38-based coordinates. Then we applied BEDtools intersect option with reciprocal overlap fraction set to 0.5 to filter out SV calls that were not located within high-confidence regions.

### Reporting summary

Further information on research design is available in the Nature Portfolio Reporting Summary linked to this article.

## Online content

Any methods, additional references, Nature Portfolio reporting summaries, source data, extended data, supplementary information, acknowledgements, peer review information; details of author contributions and competing interests; and statements of data and code availability are available at 10.1038/s41587-024-02190-7.

## Supplementary information

Supplementary Information Supplementary Notes 1–7, Figs. 1–4 and Files 1–8.

Reporting Summary

Supplementary Tables 1–14.

## Data Availability

The sources of HiFi, ONT and CLR reads of the six family datasets and HCC1395 normal-tumor paired cell are listed in Supplementary Table 1. The human reference genome GRCh37 was downloaded from http://ftp-trace.ncbi.nih.gov/1000genomes/ftp/technical/reference/phase2_reference_assembly_sequence/hs37d5.fa.gz. The human reference genome GRCh38 was downloaded from http://ftp.1000genomes.ebi.ac.uk/vol1/ftp/technical/reference/GRCh38_reference_genome/.

## Code availability

SVision-pro (v.1.6) is available at GitHub (https://github.com/songbowang125/SVision-pro.git)^34^. The scripts for model training, performance valuation and simulate data generation are available at GitHub (https://github.com/songbowang125/SVision-pro-Utils.git)^35^. Both repositories are available under a GNU General Public License v.3.0, and are free for noncommercial use by academic, government and nonprofit/not-for-profit institutions.
