# Supplementary material for: De novo and somatic structural variant discovery with SVision-pro
Source: Nat Biotechnol. 2024 Mar 22;43(2):181–5. doi: 10.1038/s41587-024-02190-7 (PMC11825360; doi:10.1038/s41587-024-02190-7)
Supplement: Supplementary file 1 — Supplementary Notes 1–7, Figs. 1–4 and Files 1–8. [file 41587_2024_2190_MOESM1_ESM.pdf]

nature biotechnology

Brief Communication

https://doi.org/10.1038/s41587-024-02190-7

De novo and somatic structural variant discovery with SVision-pro

In the format provided by the authors and unedited

Contents

Supplementary_Notes 1

Supplementary Note 1. SVision-pro model training, selection and interpretation 1

Supplementary Note 2. SVision-pro resource usage 2

Supplementary Note 3. Experimental validation for germline and de novo calling 2

Supplementary Note 4. De novo and somatic simulation 3

Supplementary Note 5. Computational validation for somatic calling 4

Supplementary Note 6. Aligner and Caller version and parameters 4

Supplementary Note 7. Merging approaches version and parameters 6

Supplementary Figures 8

Supplementary Fig.1 | Examples of different alignment mapping conditions 8

Supplementary Fig.2 | Illustration of insertion-related representation 9

Supplementary Fig.3 | CSV component and breakpoint comparison 10

Supplementary Fig.4 | SVision-pro resource consumption 11

Supplementary Files 12

Supplementary File 1. Experimental validation of CSVs in ChineseQuartet 13

Supplementary File 2. Verification of the 32,549bp deletion in chromosome 1 32

Supplementary File 3. Verification of the complex locus at chr11:34,686,676 39

Supplementary File 4. Verification of 26 de novo SVs of SVision-pro 49

Supplementary File 5. Verification of 19 false-positive de novo calls of Sniffles2 76

Supplementary File 6. Experimental validation of de novo SVs in ChineseQuartet 96

Supplementary File 7. Experimental validation of other callers' False-positive (FP) de novo SVs in ChineseQuartet 99

Supplementary File 8. Retrieved eight CSVs from HCC1395 N-T paired cell line 128

Supplementary Notes

Supplementary Note 1. SVision-pro model training, selection and interpretation

Model training data simulation. We trained the instance segmentation models with simulated data, which contains known SV types, lengths and genotypes as prediction labels during the simulation procedure. Since CSVs typically comprise multiple internal subcomponents, we train the instance segmentation framework in SVision-pro with only five SSVs, including deletion, insertion, inversion, duplication and inverted duplication. For each SSV type, VISOR[1] was used to randomly simulate 1,000 non-overlapped events. Note that, for the two duplication SSVs, dispersed events and tandem events were equally simulated (500 events for each). In particular, those 5,000 SSVs of the five types were generated by the VISOR randomregion.R module with parameters '-n 5000 –r 20:20:20:10:10:10:10 –l 500 –s 150 -v deletion,insertion,inversion, tandem duplication,inverted tandem duplication,dispersed duplication,dispersed inverted duplication'.

Model training process. SVision-pro adopted five neural-network-based image segmentation models, including mini-Unet, Lite-Unet, Unet, FCN and DeepLabV3. The mini-Unet architecture was derived from the standard Unet but with fewer encoder/decoder blocks and smaller output channels for convolutional kernels. The Lite-Unet was also derived from the standard Unet architecture, with the output channels of convolutional kernels being reduced. Briefly, the Lite-Unet consisted of five encoder blocks, four decoder blocks and one 1x1 convolutional layer for the final outputs (**Extended Data Fig. 5b**). Concatenation operations combined feature maps from a downward decoder block with another from upward encoder block, preserving high-resolution information from the input image and enabling the network to learn complex features through multiple layers of convolutional kernels. Unet, FCN and DeepLabV3 were implemented in their default network architecture. During the training procedure, the batch size was set to 2 and the learning rate was set to 0.0001. The loss function was defined as the sum of Cross Entropy Loss and Dice Loss. Adam Optimizer was utilized to guild the training process. Additionally, an early stopping strategy was implemented to determine the best trained models. This strategy assumed that the validation accuracy would remain unchanged (within a tolerance of 0.001) for a continuous period of 15 epochs, thereby signifying the end of training.

Model selection. Upon completion of the training procedure, we evaluated the performance of the five trained models on the validation dataset to determine the default model of SVision-pro. Our selection criterion sought to choose the best-performing model with as few parameters as possible (**Fig. 1c**). This consideration was essential to ensure the usability and facilitate the adoption by general scientific users. Among the five models, the smallest model, mini-Unet (model size: 149kb), demonstrated excellent performance at an image size of 256. However, when applied with larger image sizes (e.g. 1024), its validation accuracy fell to 91%. The second smallest model, Lite-Unet (610kb), achieved comparably high validation accuracy (~99%) at all three image sizes to the other three large models: Unet (86.7Mb), FCN (71.1MB) and DeepLabV3 (58.5Mb) (**Extended Data Fig. 5b**). Shrinking parameters further (mini-Unet) led to performance degradation (**Extended Data Fig. 5a**). Taking all factors into account, the default instance segmentation model in SVision-pro was Lite-Unet, which struck a balance between its parameter size and performance.

Model interpretation. Neural networks are complex models with a large number of parameters, making them challenging to comprehend. Therefore, interpreting neural network models becomes crucial in order to gain insight into their functioning and understand the factors that influence their decision-making processes. To accomplish this, we adopted two distinct approaches for model interpretation, including Grad-CAM[2] and Feature Ablation, which provide valuable insights by highlighting the pixels and regions that contribute to the classification of specific classes. These approaches were implemented using the Captum[3] library, following the official instructions provided. As a result, we investigated the interpretability of Lite-Unet with Grad-CAM[2] and Feature Ablation (Methods). Certain image pixels, which guided classification decisions, were highlighted, while those irrelevant pixels were silenced **(Fig. 1d and Extended Data Fig. 5c)**. Those highlighted pixels matched the desired areas, indicating that the adopted Lite-Unet achieved high prediction accuracy and preserved the qualitative interpretability. Moreover, the later layers of Lite-Unet produced attribution maps that were very similar to the final segmentation map, while the earlier layers produced more abstract results, focusing on areas of types rather than specific ones (**Extended Data Fig. 5d**). Collectively, we selected Lite-Unet as the default model.

Supplementary Note 2. SVision-pro resource usage

We estimated the computational resources for SVision-pro (**Supplementary Table 14**). Owing to the lightweight image segmentation model, we were able to execute SVision-pro on personal computers (CPU: Intel i9-13900K, Memory: 64GB, Hard Disk Drive). For example, with eight threads, SVision-pro finished *de novo* SV detection in the 5-40-fold HiFi AshkenazimTrio dataset within 9-47 minutes, using 0.89-2.31G of maximum memory (**Supplementary Fig. 4a**). For the ONT dataset, SVision-pro spent 24-135 minutes and consumed 1.02-3.68G of maximum memory. The computational process of SVision-pro could be accelerated by using more threads, such as in a cluster node (CPU: Intel Xeon Gold 6240R, Memory: 376GB, RAID) with 24 threads, resulting in an average of 3-18 and 15-77 minutes for HiFi and ONT sequencing, respectively (**Supplementary Fig. 4a**). We further exported the proportion of the representation module and the recognition module, and found that the representation module, including the candidate search process, took up an average of 2/3rds and 4/5ths of the total running time in HiFi and ONT sequencing, respectively (**Supplementary Fig. 4b and Supplementary Table 14**).

Supplementary Note 3. Experimental validation for germline and de novo calling

We performed experimental validation (PCR and Sanger sequencing) on the same batch DNAs (batch number: FDU_Quartet_DNA_20171028) used for sequencing the ChineseQuartet dataset. Firstly, we designed primers flanking the selected SSVs and CSVs. Target lengths were calculated by counting the base-pair numbers between upstream and downstream primers. Next, we attempted to amplify each event and conducted gel electrophoresis. PCR bands that matched the target sequence length were extracted from gels for subsequent Sanger sequencing. Inconclusive events indicated either primer designation failure (due to high GC contents or highly repetitive flanking sequence) or PCR amplification failure in three attempts with alterations of the PCR conditions and template amounts.

To reveal the false-positive rate of CSV detection of SVision-pro, we performed PCR and Sanger sequencing to experimentally validate all the CSVs (n=33) detected from the ChineseQuartet

offspring (**Supplementary Table 4**). Except for 15 inconclusive events (due to primer design or amplification failure), all the remaining 18 CSVs showed specific PCR bands that matched the targeted lengths with subsequent Sanger sequencing confirming the appropriate sequences (Methods and **Supplementary File 1**). These experimental results demonstrate that SVision-pro has a high sensitivity and low false-positive rate for CSV detection.

Given the discrepancies in *de novo* calls among approaches, we performed PCR and Sanger sequencing validations using the ChineseQuartet. We firstly validated the two *de novo* calls reported by SVision-pro in the ChineseQuartet. Despite one being a repeat extension and the other a contraction, experimental validation confirmed that the two calls were only present in the child genome and not in the genomes of either parent **(Supplementary File 6)**. Then, we randomly selected 50 *de novo* calls reported by other approaches (4,404 in total) for experimental validation. Of the 25 events which could be experimentally tested (the remainder, being technically challenging events, showed a high degree of PCR failure), all could be detected in both the child and parental genomes (**Supplementary File 7** and **Supplementary Table 10**), indicating they were actually germline events. Taken together, these results suggested that SVision-pro reduces false-positive calls in Mendelian samples and reports high-quality *de novo* SVs.

Supplementary Note 4. De novo and somatic simulation

*CSV Mendelian detection performance in simulated trio***.** Since there were few CSVs in real samples, we simulated a trio dataset to further evaluate the performance of Mendelian CSV detection. The simulation process involved implanting 3,000 ground-truth CSVs into templated genomes to create the trio dataset consisting of a child, father, and mother. We implanted the simulated 3,000 ground-truth CSVs into templated genomes to simulate a trio dataset (child, father and mother), on the basis of the following steps:

1. Generating two haplotypes for each individual genome using chromosome 1 to X of the GRCh38 genome as the template.
2. Tagging CSVs events with ‘inherited’ or ‘*de novo*’ labels and maintaining a 1:1 ratio between them. ‘*De novo*’ meant a child-specific CSV. ‘Inherited’ meant that this CSV presented in child genome as well as parents’ genomes, and that the child inherited this CSV from parents
3. For '*de novo*' events, we directly implanted them into a random haplotype of the child using the same method described in the SVision paper. For ‘inherited’ events, we first needed to simulate the genotypes of parents. The genotype symbols 1/1, 0/1 and 0/0 represented homozygous, heterozygous and non-present events. Based on the parent genotypes, the child's genotype was computed following the Mendelian Law. For example, if the parent genotypes were both 0/1, the child genotype could be 1/1 (with a 0.25 probability), 0/1 (with a 0.50 probability) or 0/0 (with a 0.25 probability). Finally, using the genotypes of this trio, the CSV was implanted into two, one, or none of the haplotypes for each individual.
4. Simulated 60X HiFi and ONT reads and alignment. We employed PBSIM2[4] to simulated HiFi and ONT reads. HiFi reads were simulated in sampling-based mode, where read parameters such as length and quality score were calculated based on published HG002 HiFi reads. ONT reads were simulated in model-based mode, determining read parameters by the given R94 ONT model. Both simulated HiFi and ONT reads were aligned to human genome GRCh38 via NGMLR[5].

Somatic detection benchmark in simulated paired dataset. A major objective of somatic detection was to detect low-AF SVs in tumor genomes. To accomplish this, we simulated a tumor genome, which harbored somatic SSVs and CSVs, by replacing reads from a simulated paired normal genome, with AFs ranges from 0.01 to 0.10. Different AF SVs in the simulated tumor genome represented subclones common in real tumor samples. First, we randomly assigned ground-truth SSVs from HG002 Tier1 and 3,000 CSVs with alternative AF values of 0.01, 0.02, 0.03, 0.05, 0.08, and 0.10. Then, we generated 100X HiFi reads and 100X ONT reads with PBSIM2[4] using the GRCh38 reference genome as the templated genome. Finally, we followed a similar procedure to BAMsurgeon[6], which primarily handled small variants rather than SVs, to implant those low-AF SVs into the simulated reads of tumor sample. Briefly, for a candidate SV, we collected all reads that spanned this SV region and implanted this SV into a specific number of candidate reads (coverage * AF). The 100X reads ensured that even the lowest AF SVs (0.01) were supported by one SV-specific read.

Supplementary Note 5. Computational validation for somatic calling

Vapor[7] is an efficient algorithm frequently used to autonomously validates SVs using long-read sequencing data[8, 9]. To reveal the false positive rate of callers' somatic calls on HCC1395 tumor-normal pared cell lines, we used Vapor to computationally validate the somatic SVs called on the HiFi data. SVision-pro reported 2,287, Sniffles2 reported 3,306 while nanomonsv failed the calling process. For each candidate somatic call, we not only validated it using the HiFi data but also the ONT and CLR data, which were different sequencing technologies and could bolster the validation results. If Vapor confirmed the existence of the SV in the tumor sample but not the normal sample in all three sequencing technologies, we would consider it as a true-positive somatic event, otherwise, a false-positive event.

To reveal the false positive rates of callers, we applied Vapor[7] validation (Methods) among three different sequencing technologies on the detected somatic calls by SVision-pro (n=2,287) and Sniffles2 (n=3,306). Note that nanomonsv failed producing any outputs using the HiFi sequencing data. As a result, 8.7% of SVision calls while 40.3% of Sniffles2 calls were validated as non-somatic in all three sequencing technology datasets, indicating that they were potential false-positives **(Fig. 2g and Supplementary Table 13)**. As increasing the supporting read number threshold from 2 to 10, the false-positive rate of SVision-pro and Sniffles2 decreased from 8.7 to 4.3% and 40.3 to 9.8%, respectively.

Supplementary Note 6. Aligner and Caller version and parameters

For aligners, including Minimap2 (v2.20-r1061) and NGMLR (v0.2.7), only sequence technology relevant parameters are set while other parameters are default. For minimap2, '-x map-pb/map-ont' is used and for NGMLR, '-x pacbio/ont' is used. For callers, default or recommended parameters were set, except three filter-relevant parameters: *minimun_support_read_number*, *minimum_detectable_AF* and *minimum_map_quality*.

(1) For HG002 ground-truth SSVs, simulated 3000 CSVs, six family datasets and simulated trio dataset:

    minimun_support_read_number: 10

minimum_detectable_AF: default

minimum_map_quality: 20

(2) For simulated somatic SSVs and CSVs:

minimun_support_read_number: 1

minimum_detectable_AF: 0.01

minimum_map_quality: 20

(3) For HCC1395 normal-tumor paired cell lines:

minimun_support_read_number: 2

minimum_detectable_AF: 0.01

minimum_map_quality: 0

Command lines for each callers are listed as follows.

SVision (v1.3.9)

SVision -o /path/to/output -b /path/to/sample.bam -m /path/to/model.ckpt -g /path/to/referece.fasta -n sample_name -s minimun_support_read_number --min_mapq minimum_map_quality --min_sv_size 50

Sniffles2 (v2.0.7)

Single sample mode:

sniffles --input /path/to/sample.bam --vcf /path/to/output.vcf --minsupport minimun_support_read_number --mapq minimum_map_quality --minsvlen 50

Multiple samples mode:

sniffles --input /path/to/sample1.bam --snf /path/to/sample1.snf --minsupport minimun_support_read_number --mapq minimum_map_quality --minsvlen 50

sniffles --input /path/to/sample2.bam --snf /path/to/sample1.snf --minsupport minimun_support_read_number --mapq minimum_map_quality --minsvlen 50

sniffles --input /path/to/sample1.snf /path/to/sample2.snf --vcf /path/to/output.vcf --minsupport minimun_support_read_number --mapq minimum_map_quality --minsvlen 50

Somatic (Non-germline) mode

sniffles --input /path/to/sample.bam --vcf /path/to/output.vcf --minsupport minimun_support_read_number --mapq minimum_map_quality --minsupport-auto-mult minimum_detectable_AF --minsvlen 50 --non-germline

cuteSV (v2.0.2)

For HiFi long reads:

cuteSV /path/to/sample.bam /path/to/referece.fasta /path/to/output -s minimun_support_read_number -q minimum_map_quality --min_size 50 --genotype --max_cluster_bias_INS 1000 --diff_ratio_merging_INS 0.9 --max_cluster_bias_DEL 1000 --diff_ratio_merging_DEL 0.5

For ONT long reads

cuteSV /path/to/sample.bam /path/to/referece.fasta /path/to/output -s minimun_support_read_number -q minimum_map_quality --min_size 50 --genotype --max_cluster_bias_INS 100 --diff_ratio_merging_INS 0.3 --max_cluster_bias_DEL 100 --diff_ratio_merging_DEL 0.3

pbsv (v2.9.0)

pbsv discover /path/to/sample.bam /path/to/sample.svsig.gz

pbsv call --ccs -A minimun_support_read_number -O minimun_support_read_number -S 0 -P 1 -m 50 /path/to/referece.fasta /path/to/sample.svsig.gz /path/to/output.vcf

debreak (v1.0.2)

debreak –bam /path/to/sample.bam -o /path/to/output --ref /path/to/referece.fasta --min_size 50 --min_support minimun_support_read_number --rescue_large_ins --rescue_dup --poa

SVDSS (v1.0.5)

SVDSS smooth --reference /path/to/referece.fasta --bam /path/to/sample.bam --workdir /path/to/output

samtools sort -T sample_name.sort-tmp /path/to/output/smoothed.selective.bam > /path/to/output/smoothed.selective.sorted.bam

samtools index /path/to/output/smoothed.selective.sorted.bam

SVDSS search --index /path/to/referece.fasta.fmd --bam /path/to/output/smoothed.selective.sorted.bam --workdir /path/to/output –assemble

n=$(ls /path/to/output/solution_batch_*.assembled.sfs | wc -l)

SVDSS call --reference /path/to/referece.fasta --bam /path/to/output/smoothed.selective.sorted.bam --workdir /path/to/output –batches n –min-sv-length 50 --min-cluster-weight minimun_support_read_number

bcftools sort /path/to/output/svs_poa.vcf > /path/to/output.vcf

nanomonsv (0.5.0)

nanomonsv parse /path/to/tumor.bam /path/to/tumor.prefix

nanomonsv parse /path/to/normal.bam /path/to/normal.prefix

nanomonsv get -median_mapQ_thres minimum_map_quality --min_tumor_variant_read_num minimun_support_read_number --min_tumor_VAF minimum_detectable_AF --control_prefix /path/to/normal.prefix --control_bam /path/to/normal.bam /path/to/tumor.prefix /path/to/tumor.bam /path/to/reference.fasta

Supplementary Note 7. Merging approaches version and parameters

The two popular callset-merging approaches, Jasmine and SURVIVOR, were applied to generate consensus callset from multiple.

For Jasmine (v1.1.5), we set '--output_genotypes' parameter to output genotypes of every merged sample:

jasmine --output_genotypes file_list= sample_list_file out_file=/path/to/consensus.vcf genome_file=/path/to/reference.fasta

For SURVOVOR (v1.0.7), we used the recommended parameters:

SURVIVOR merge sample_list_file 1000 1 1 1 0 50 /path/to/consensus.vcf

Reference

1. Bolognini, D. et al. VISOR: a versatile haplotype-aware structural variant simulator for short- and long-read sequencing. *Bioinformatics* **36**, 1267-1269 (2020).
2. Selvaraju, R.R. et al. arXiv:1610.02391 (2016).

3. Kokhlikyan, N. et al. arXiv:2009.07896 (2020).
4. Ono, Y., Asai, K. & Hamada, M. PBSIM2: a simulator for long-read sequencers with a novel generative model of quality scores. *Bioinformatics* **37**, 589-595 (2021).
5. Sedlazeck, F.J. et al. Accurate detection of complex structural variations using single-molecule sequencing. *Nat Methods* **15**, 461-468 (2018).
6. Ewing, A.D. et al. Combining tumor genome simulation with crowdsourcing to benchmark somatic single-nucleotide-variant detection. *Nat Methods* **12**, 623-630 (2015).
7. Zhao, X.F., Weber, A.M. & Mills, R.E. A recurrence based approach for validating structural variation using long-read sequencing technology. *Gigascience* **6** (2017).
8. Collins, R.L. et al. A structural variation reference for medical and population genetics. *Nature* **581**, 444-451 (2020).
9. Vialle, R.A., de Paiva Lopes, K., Bennett, D.A., Crary, J.F. & Raj, T. Integrating whole-genome sequencing with multi-omic data reveals the impact of structural variants on gene regulation in the human brain. *Nat Neurosci* **25**, 504-514 (2022).

Supplementary Figures

Supplementary Fig.1

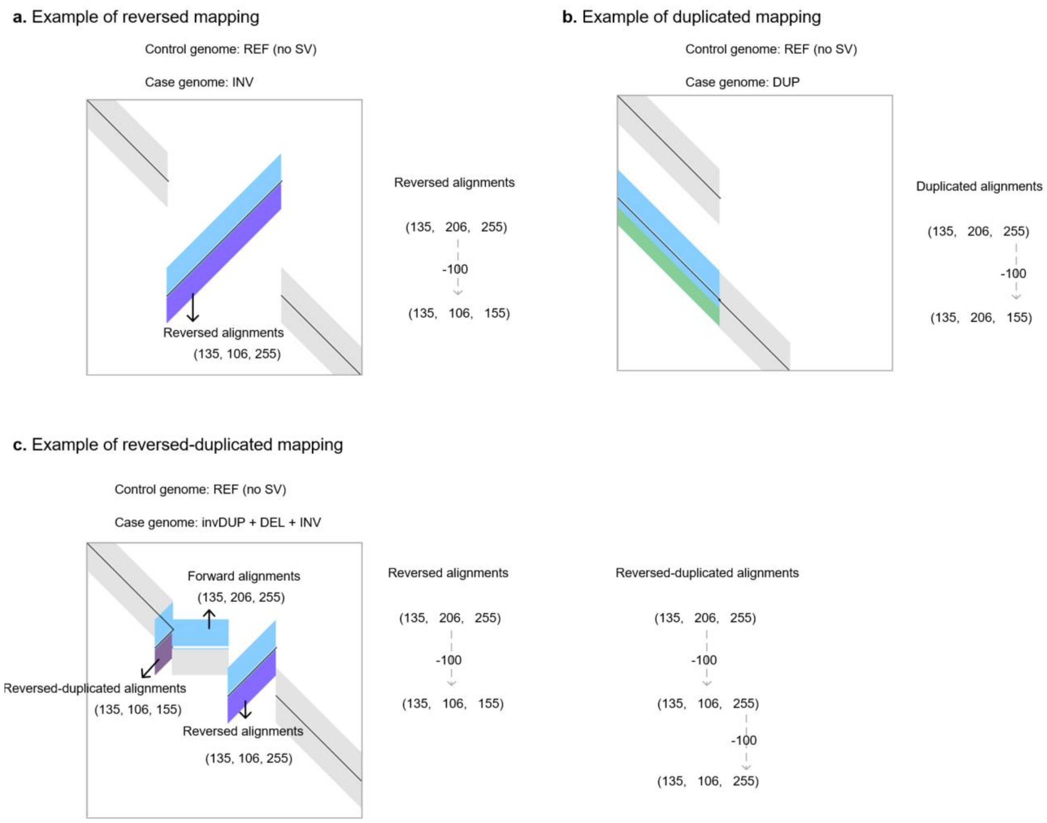

Supplementary Fig.1 | Examples of different alignment mapping conditions. a, Reversed mapping requires subtracting 100 from the second channel. **b,** Duplicating mapping requires subtracting 100 from the third channel. **c,** Reversed-duplicated mapping requires subtracting 100 from both the second and third channels.

Supplementary Fig.2

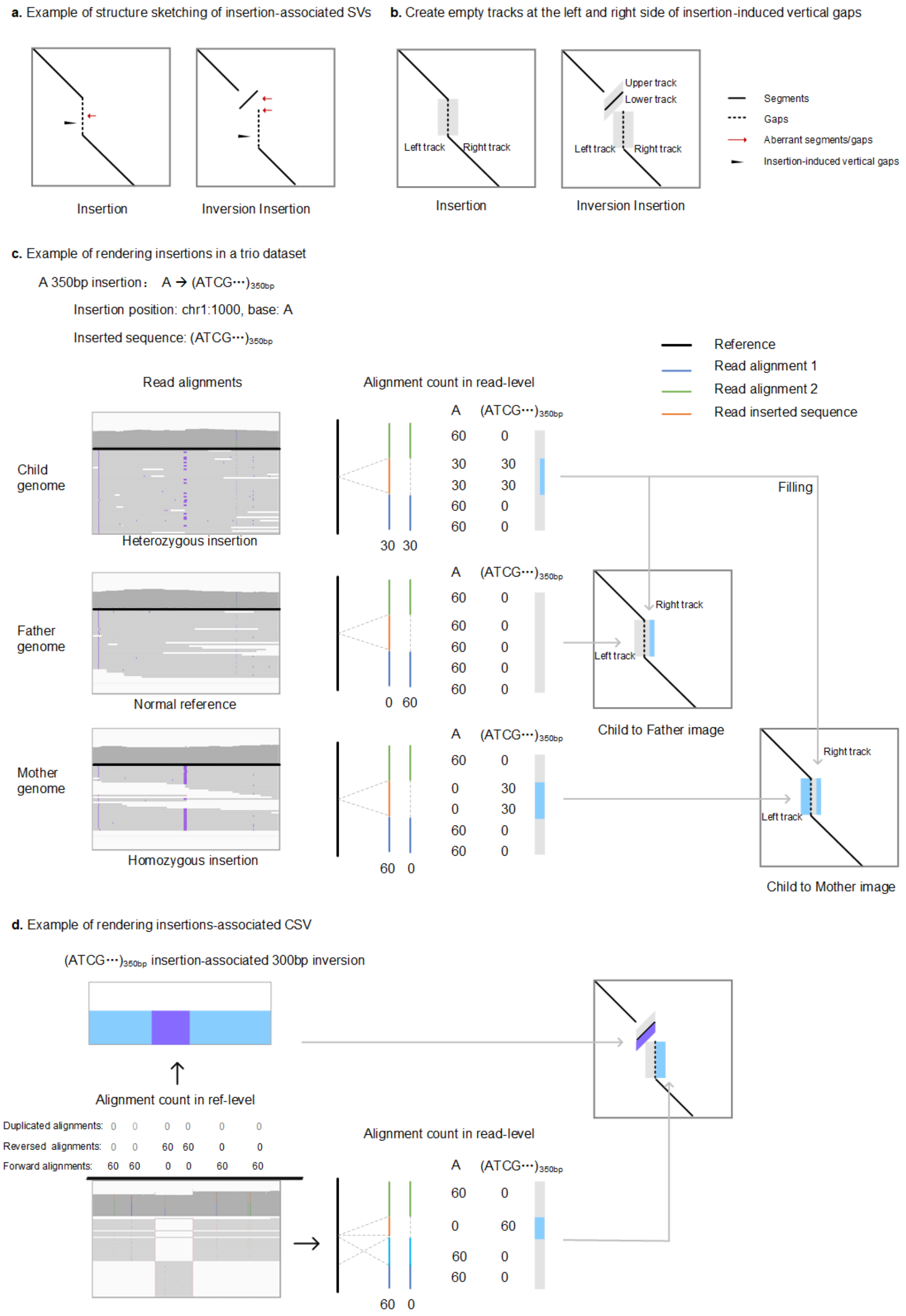

Supplementary Fig.2 | Illustration of insertion-related representation. a, Sketching the insertion-related SVs**. b,** Create empty tracks at the left and right side of insertion-induced vertical gaps. **c,** Rendering insertions in a trio dataset. **d,** Rendering insertions-associated CSV.

Supplementary Fig.3

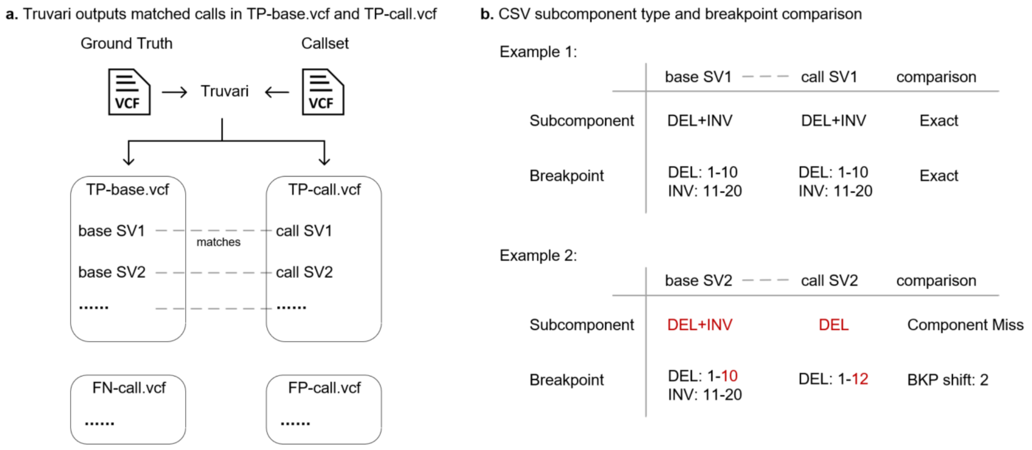

Supplementary Fig.3 | CSV component and breakpoint comparison. a, we extracted the matched SV record pairs between the ground truth and callset from Truvari output files. **b,** for each matched record pair, if any SV component from the ground-truth record was absent from the called record, this record pair was marked as inaccurate (Example 2).

Supplementary Fig.4

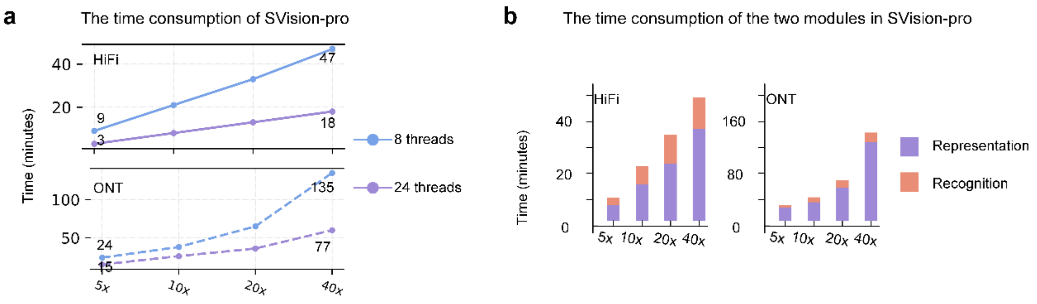

Supplementary Fig.4 | SVision-pro resource consumption. a, Runtime of SVision-pro given HiFi and ONT data at various sequencing coverages and using either 8 or 24 threads (to simulate use on a PC and cluster, respectively). **b,** Proportion of runtime used by the representation and recognition modules.

Supplementary Files

Supplementary File 1. Experimental validation of CSVs in ChineseQuartet

(See Supplementary Table 4 for details)

CSV information

ID	SV	PCR lane ID
CSV-1	chr1-14109813-14112445-INS+DEL	1
CSV-2	chr1-43593642-43594284-INV+INS+DEL	2
CSV-3	chr1-187495697-187497596-DEL+INV	3
CSV-4	chr1-248510749-248519266-INS+DEL	4
CSV-5	chr2-122719024-122724930-INV+DEL	5
CSV-6	chr2-152603406-152604918-DEL+INV	6
CSV-7	chr2-231578973-231579346-INS+DEL	7
CSV-8	chr2-242127550-242129857-DEL+INV+DEL	8
CSV-9	chr3-9584393-9585301-INS+DEL	9
CSV-10	chr4-145693877-145694353-INS+INV	10
CSV-11	chr5-148173477-148175216-DEL+INV+DEL	11
CSV-12	chr6-94031358-94031695-INV+INS	12
CSV-13	chr6-108293166-108293570-INS+DEL	13
CSV-14	chr7-566767-567341-INS+DEL	14
CSV-15	chr7-56369054-56375181-INS+tDUP	15
CSV-16	chr7-65258046-65259112-INS+DEL	16
CSV-17	chr8-12609996-12611663-INS+DEL	17
CSV-18	chr8-72111387-72111630-INS+INV	18
CSV-19	chr8-102423362-102423790-INV+INS+DEL	19
CSV-20	chr8-133312972-133313542-INS+DEL	20
CSV-21	chr8-144021102-144022109-INS+DEL	21
CSV-22	chr9-74283224-74283474-INS+INV+INS	22
CSV-23	chr10-57497185-57498224-INS+DEL	23
CSV-24	chr14-37300397-37302023-INS+DEL	24
CSV-25	chr14-65375820-65376575-DEL+INV+DEL	25
CSV-26	chr16-15115699-15116376-DEL+INV	26
CSV-27	chr16-29226811-29227350-INS+INV+INS+DEL	27
CSV-28	chr16-48616973-48617404-INV+INS	28
CSV-29	chr16-82130592-82130903-INS+DEL	29
CSV-30	chr16-85155340-85156196-INV+INS+DEL	30
CSV-31	chr19-15707370-15708594-INS+DEL	31
CSV-32	chr21-26648369-26649407-INV+DEL	32
CSV-33	chrX-104069779-104082053-INS+DEL	33

PCR results

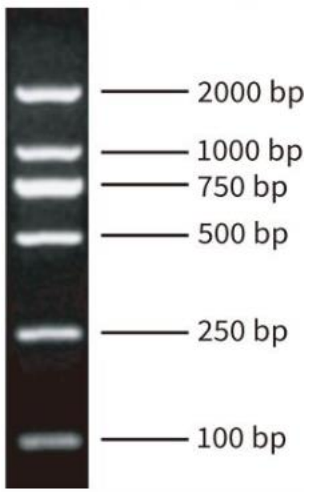
2000 bp
1000 bp
750 bp
500 bp
250 bp
100 bp

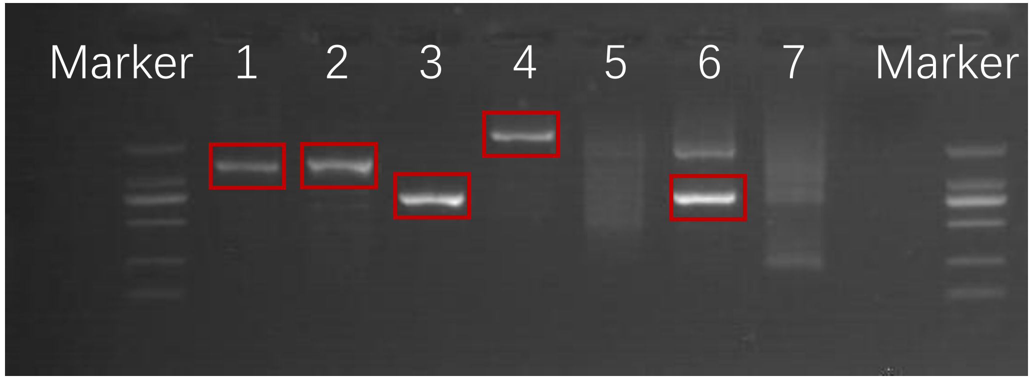
Marker 1 2 3 4 5 6 7 Marker

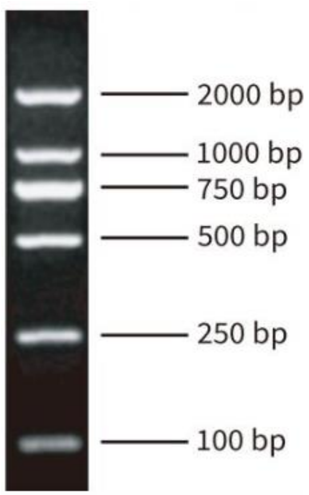
2000 bp
1000 bp
750 bp
500 bp
250 bp
100 bp

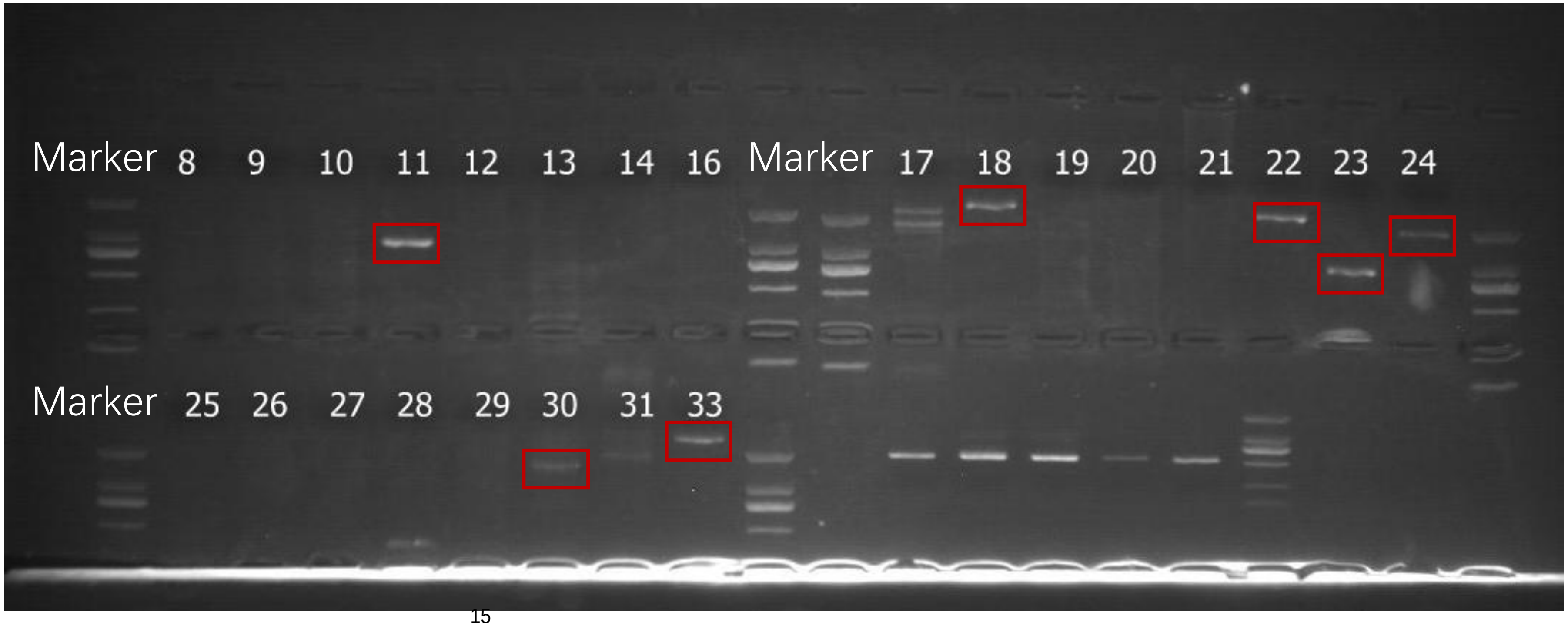
Marker 8 9 10 11 12 13 14 16 Marker 17 18 19 20 21 22 23 24
Marker 25 26 27 28 29 30 31 33

Nested PCR results

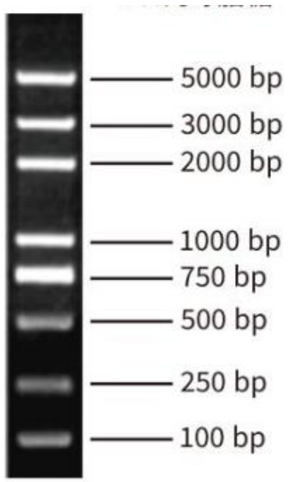

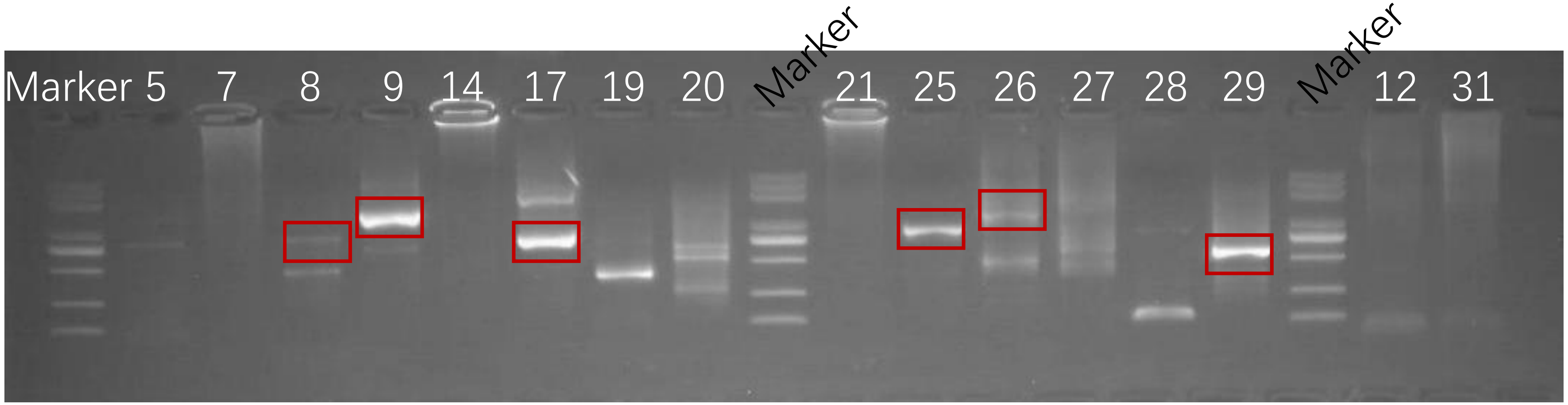

Sanger sequencing results for ID CSV-1 chr1-14109813-14112445-INS+DEL

■ Sanger trace alignment evidences:

5' Upstream (15bp) Downstream (15bp) 3'

Reference: ATTCCTTATCAAAAC | GATTTCAGCGTCAAGTAGGAG……CCCACTCCTCTTTGGCCACTGTGGGTTGTCAACA | GTCTTGATTCCAAG

CSV sequence: ATTCCTTATCAAAAC | TTTTTTTTTTTTGAGATGGAGTCTCGCT……TTGCATATCTATCTATTTCTCTTAGGAG | GTCTTGATTCCAAG

INS breakpoint DEL breakpoint

(CSV sequence length: 813bp)

Child 5' Sanger trace: ATTCCTTATCAAAACTTTTTTTTTTTGAGATGGAGTCTCGCTCTGT…. GCATTTGACCTGTGGATACA (703bp)

(688bp) CCGGCTAATTTTTGTATTTTAGTAGAGACGGGCTTTCA……CTATTTCTCTTAGGAGGTCTTGATTCCAAG Child 3' Sanger trace

■ Sanger trace raw evidences:

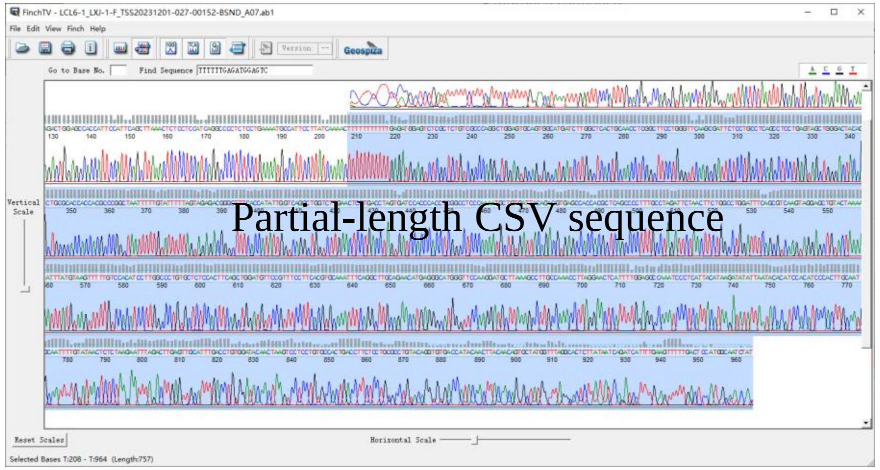

Child 5' primer Sanger trace

The CSV sequence in Sanger trace is marked in Blue

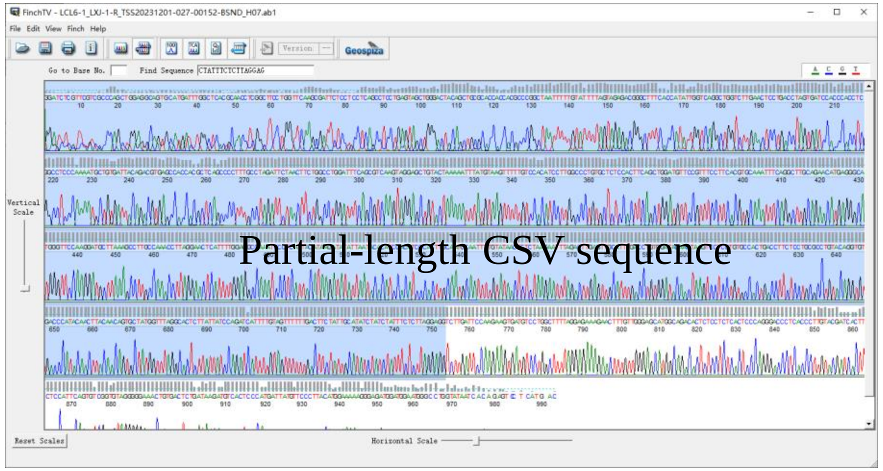

Child 3' primer sanger trace (reverse complement sequence)

■ Conclusion:

Sanger trace confirmed all the subcomponents' breakpoints, therefore, it is a true-positive CSV

Sanger sequencing results for ID CSV-2 chr1-43593642-43594284-INV+INS+DEL

- Sanger trace alignment evidences:

5’ Upstream (15bp) (667bp) Downstream (15bp) 3’

Reference: TCCTTATGAAGTGGG | TTATGGTCACAAGGTAAGCCACCGT… CTTCCTTCAGGCCTTGGGAAAGTGTCACCTC | CCCAAGGCCTGAAGG

CSV sequence: TCCTTATGAAGTGGG | ACCATGTGCATGTACCAGTGATGTCA……GAGGGTTGCCTTGGGGAGGTGACACTTT | CCCAAGGCCTGAAGG

INV breakpoint INS breakpoint (to 5’ start: 589bp) DEL breakpoint

(CSV sequence length: 974bp)

Child 5’ Sanger trace: TCCTTATGAAGTGGGACCATGTGCATG……ACCTTGGCCTCCCC (490bp)

(114bp) AAAAAAAA…CACTTTCCCAAGGCCTGAAGG Child 3’ Sanger trace

- Sanger trace raw evidences:

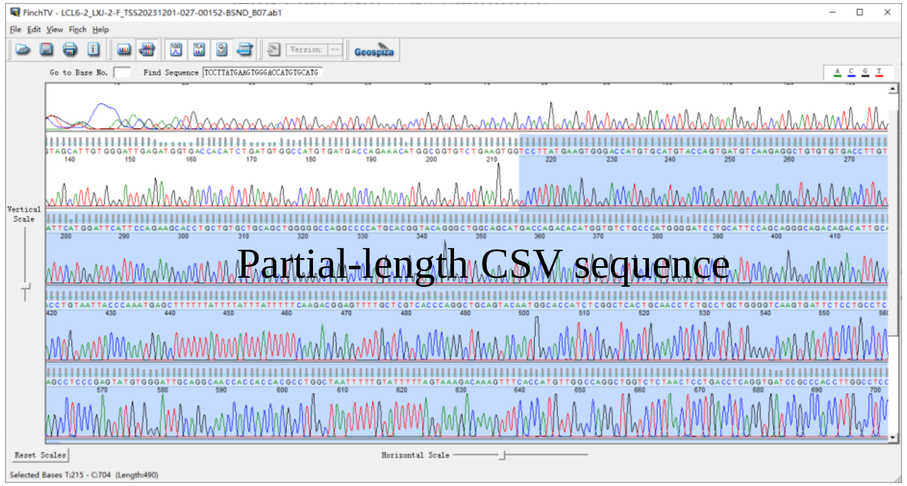

Child 5’ primer Sanger trace

The CSV sequence in Sanger trace is marked in Blue

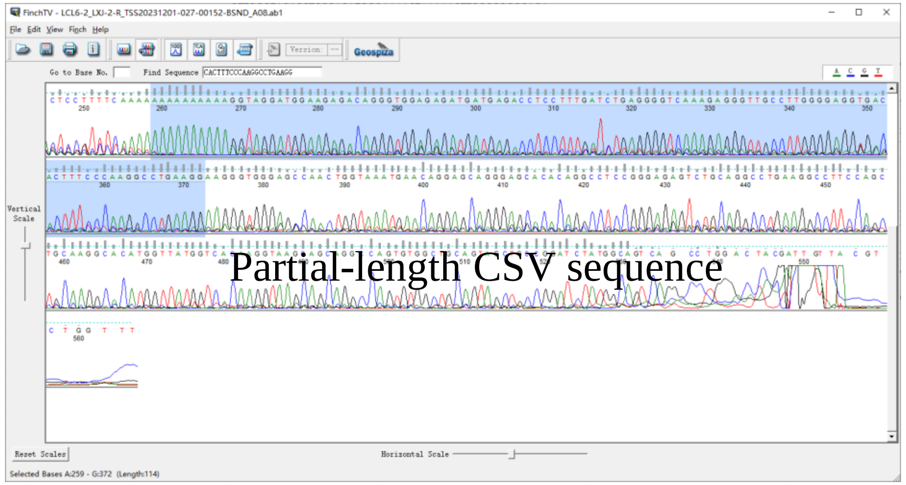

Child 3’ primer sanger trace (reverse complement sequence)

- Conclusion:

Sanger trace confirmed 2 of the 3 subcomponents’ breakpoints (INV and DEL), therefore, it is a true-positive CSV

Sanger sequencing results for ID CSV-3 chr1-187495697-187497596-DEL+INV

■ Sanger trace alignment evidences:

5’ Upstream (15bp) (1945bp) Downstream (15bp) 3’

Reference: ATTGAGTATTACATT | CCAAGAATACAGTGTATTTCTATAAA… AAAGCATATTTACCTTGTGAGAGAGATAG | TCTTAATATTACTAA

CSV sequence: ATTGAGTATTACATT | AATATGCTTTCTGTTTTTGGGTGTCA………CTCCTGGATTTCCTTGTGAGAGAGATAG | TCTTAATATTACTAA

DEL breakpoint ... INV breakpoint

(CSV sequence length: 296bp)

Child 5’ Sanger trace: ATTGAGTATTACATTAATATGCTTTCTGTTTTTGGGTGTCA……AATGGTCTCCTGGATTTCCTTGTGAGAGAGATAGTCTTAATATTACTAA (296bp)

■ Sanger trace raw evidences:

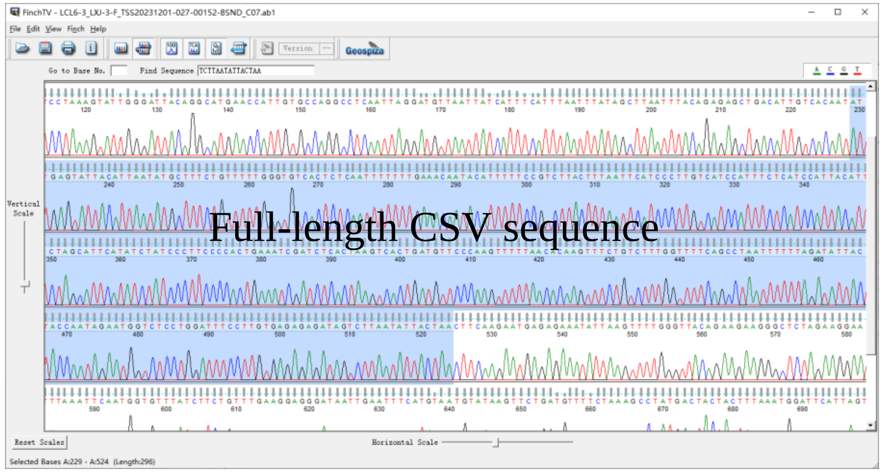

Child 5’ primer Sanger trace

The CSV sequence in Sanger trace is marked in Blue

■ Conclusion:

Sanger trace confirmed all the subcomponents’ breakpoints, therefore, it is a true-positive CSV

Sanger sequencing results for ID CSV-4 chr1-248510749-248519266-INS+DEL

■ Sanger trace alignment evidences:

5' Upstream (15bp) (8638bp) Downstream (15bp) 3'

Reference: GAAAAAGACTCAAGG | TAAAGGGAACCATCATCTTTTTCAG… GATGATGGTGATGTACAGAAGGGTTTTTGG | TTTTCTCCCATTCTA

CSV sequence: GAAAAAGACTCAAGG | CAGAGGGAACAGTCTTCTTTTTCCTTTA……CTTTGTCAGACAGACAGATTGCAAAAT | TTTTCTCCCATTCTA

INS breakpoint DEL breakpoint

(CSV sequence length: 2512bp)

Child 5' Sanger trace: GAAAAAGACTCAAGGCAGA…. ATCTCTGTCTCATG (469bp)

(670bp) AAGGAAAAGAAA…TGCAAAATTTTTCTCCCATTCTA Child 3' Sanger trace

■ Sanger trace raw evidences:

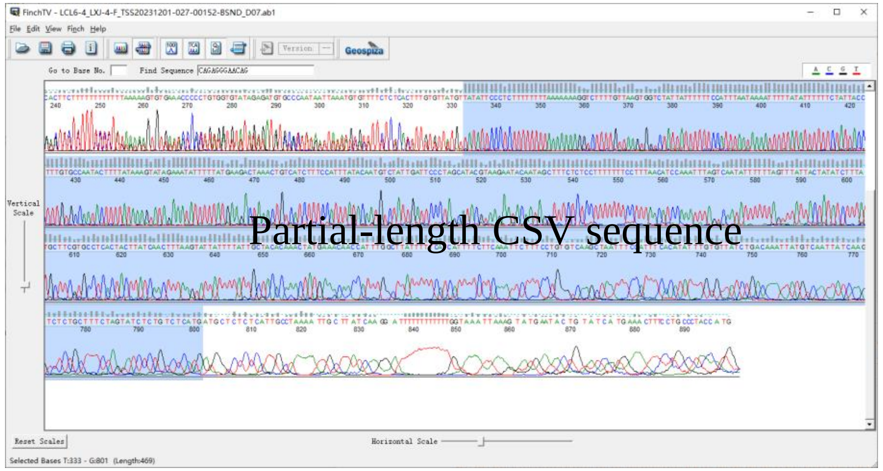

Child 5' primer Sanger trace

The CSV sequence in Sanger trace is marked in Blue

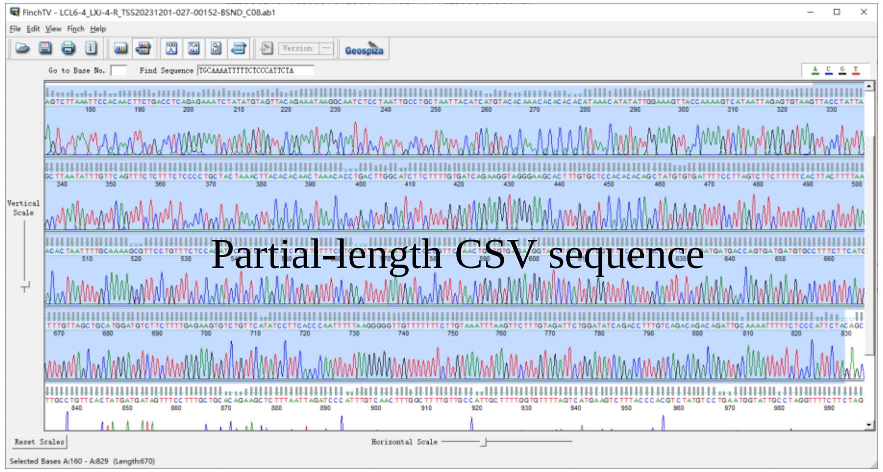

Child 3' primer sanger trace (reverse complement sequence)

■ Conclusion:

Sanger trace confirmed all the subcomponents' breakpoints, therefore, it is a true-positive CSV

Sanger sequencing results for ID CSV-6 chr2-152603406-152604918-DEL+INV

- Sanger trace alignment evidences:

5’ Upstream (15bp) (1549bp) Downstream (15bp) 3’

Reference: TTAAGTCTGTTTTTT TTTTTTTTTTTAAGATTAGAGGTATT…TTTCGTATAAAACCTCAGGGTTTTTTACCCA GTTCTGACCTTTCGT

CSV sequence: TTAAGTCTGTTTTTT CTTTAGCAGAAACCCCAATACAGGCT……AAAAAAAACAGTGTACTTTGGTCTTGTT GTTCTGACCTTTCGT

DEL breakpoint INV breakpoint

(CSV sequence length: 364bp) Downstream (15bp)

Child 5’ Sanger trace: TTAAGTCTGTTTTTTCTTTAGCAGAAACCCCAATACAGGC……TCTCAAAAAAAAAACAGTGTACTTTGGTCTTGTTGTTCTGACCTTTCGT (364bp)

- Sanger trace raw evidences:

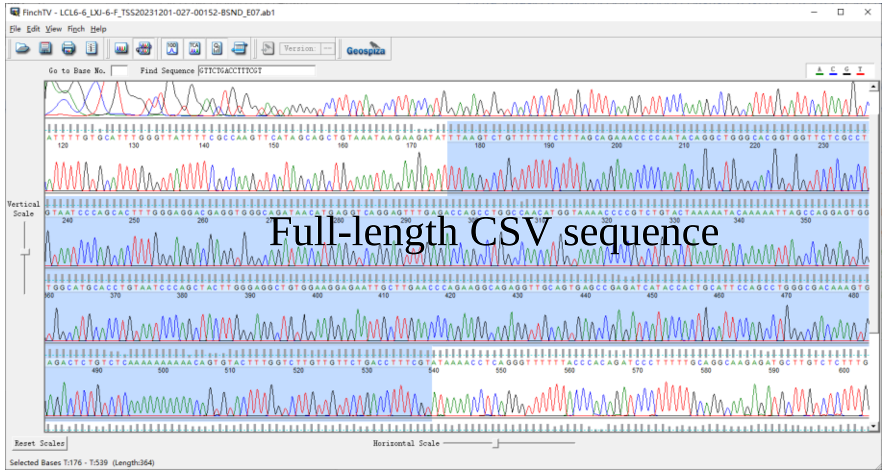

Child 5’ primer Sanger trace

The CSV sequence in Sanger trace is marked in Blue

- Conclusion:

Sanger trace confirmed all the subcomponents’ breakpoints, therefore, it is a true-positive CSV

Sanger sequencing results for ID CSV-8 chr2-242127550-242129857-DEL+INV+DEL

■ Sanger trace alignment evidences:

5' Upstream (15bp) (2340bp) Downstream (15bp) 3'

Reference: TATAAAAAACTGAAG AAAGGTCTAATGTGGCTTAAATACAGAAG…AGTTACTATATTTTATTAAAGGAAATT AGCCTTATCTCTTAT

CSV sequence: TATAAAAAACTGAAG TATATTACATACATATATATATGTTT….GTGGGCATGTAATGAATATTTGTTAAGTGA AGCCTTATCTCTTAT

DEL breakpoint INV breakpoint DEL breakpoint

(CSV sequence length: 473bp)

Child 5' Sanger trace TATAAAAAACTGAAGTATATTACATACATATATATATGTT….AAACAGTGGGCATGTAATGAATATTTGTTAAGTGAAGCCTTATCTCTTAT (473bp)

■ Sanger trace raw evidences:

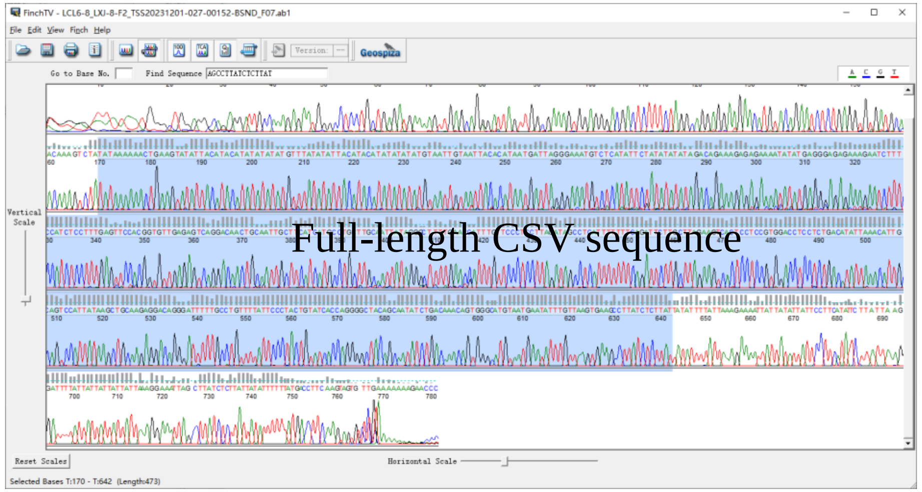

Child 5' primer Sanger trace

The CSV sequence in Sanger trace is marked in Blue

■ Conclusion:

Sanger trace confirmed all the subcomponents' breakpoints, therefore, it is a true-positive CSV

Sanger sequencing results for ID CSV-11 chr5-148173477-148175216-DEL+INV+DEL

■ Sanger trace alignment evidences:

5’ Upstream (15bp) (1764bp) Downstream (15bp) 3’

Reference: CTTTGAAATAGGAAA | TGAAAAAAAAGTGATAAATATTTGAAATCA…AATCCAATAATTTTGTCTTTTGTCAT | TAGTCAATTCCCTTT

CSV sequence: CTTTGAAATAGGAAA | GTTAATTCCCTCACACTTTGGCTGAAT……. ACTACAATTTTTCTTAAAATAAAAAAA | TAGTCAATTCCCTTT

DEL breakpoint INV breakpoint DEL breakpoint

(CSV sequence length: 407bp)

Child 5’ Sanger trace: CTTTGAAATAGGAAAGTTAATTCCCTCACACTTTGGCTGA…. AAGCAAAACTACAATTTTTCTTAAAATAAAAAAATAGTCAATTCCCTTT (407bp)

■ Sanger trace raw evidences:

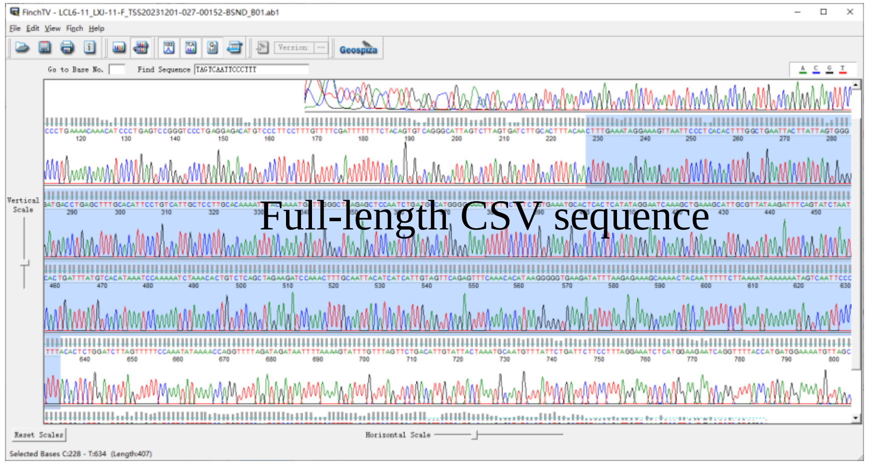

Child 5’ primer Sanger trace

The CSV sequence in Sanger trace is marked in Blue

■ Conclusion:

Sanger trace confirmed all the subcomponents’ breakpoints, therefore, it is a true-positive CSV

Sanger sequencing results for ID CSV-17 chr8-12609996-12611663-INS+DEL

■ Sanger trace alignment evidences:

5’ Upstream (15bp) (920bp) Downstream (15bp) 3’

Reference: TTTAAAAATGTAAAC N AGGAGTTCGAGAGCA

CSV sequence: TTTAAAAATGTAAAC ACCTCCTGGGGCTAGTGAGAGTCCTCC……CCTTGTGATCTGCCTGCCTCGGCCTCCC AGGAGTTCGAGAGCA

INS breakpoint DEL breakpoint

(CSV sequence length: 1086bp)

Child 5’ Sanger trace: TTTAAAAATGTAAAC…TCCTCCAAACAG (48bp)

Child 3’ Sanger trace: NA

■ Sanger trace raw evidences:

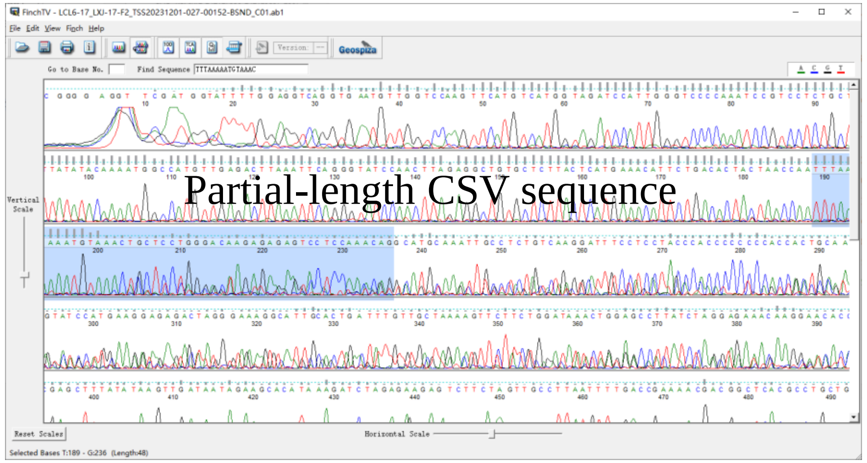

The CSV sequence in Sanger trace is marked in Blue

Child 5’ primer Sanger trace

NA

Child 3’ primer sanger trace (reverse complement sequence)

■ Conclusion:

Sanger trace confirmed 1 of the 2 subcomponents’ breakpoints, therefore, it is a true-positive CSV

Sanger sequencing results for ID CSV-18 chr8-72111387-72111630-INS+INV

■ Sanger trace alignment evidences:

5’ Upstream (15bp) (245p) Downstream (15bp) 3’

Reference: GAAAGCTTTGCCGGT | GTGAATCATTCTACTTTCTACCTCC… GATGGATACAGGTTGATTTCATATCTTAAAT | ATGGTGTAAGGGGGA

CSV sequence: GAAAGCTTTGCCGGT | CTCTCTTTTCTCCAGCAAAATTC……AAAGTAGAATGATTCACACCAGAGGCTGGGA | ATGGTGTAAGGGGGA

INS breakpoint INV breakpoint

(CSV sequence length: 2180bp)

Child 5’ Sanger trace: GAAAGCTTTGCCGGTCTCTCTTTTCT……CAAAAAAAAAAA (759bp)

(712bp) AAGGAACTCAA…AGGCTGGGAATGGTGTAAGGGGGA Child 3’ Sanger trace

■ Sanger trace raw evidences:

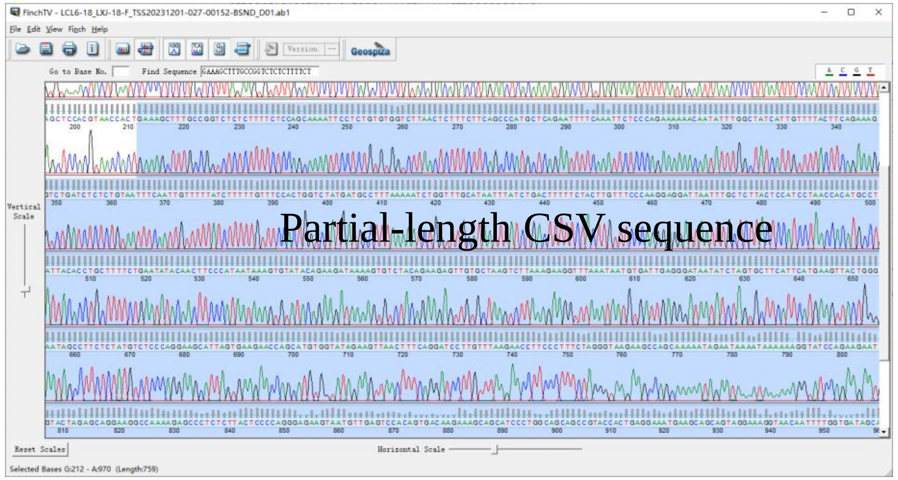

Child 5’ primer Sanger trace

The CSV sequence in Sanger trace is marked in Blue

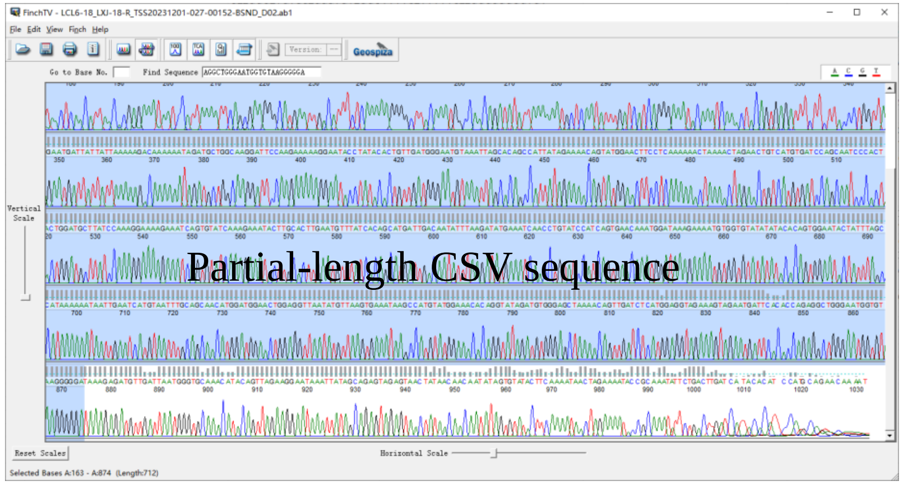

Child 3’ primer sanger trace (reverse complement sequence)

■ Conclusion:

Sanger trace confirmed all the subcomponents’ breakpoints, therefore, it is a true-positive CSV

Sanger sequencing results for ID CSV-22 chr9-74283224-74283474-INS+INV+INS

■ Sanger trace alignment evidences:

5' Upstream (15bp) (252p) Downstream (15bp) 3'

Reference: TCACCTATTAACTAG AAAGCCTGGCGTCACACCCTGACTTG… CACCCCTCTCTCACCCTCCTGCCCACTGTA GCCTAAGTGACATCA

CSV sequence: TCACCTATTAACTAG AGTCACAGTCAACCAAGTTCAGCACTA……..CTGGCCAATTGCAGGCCTTTGAAGGT GCCTAAGTGACATCA

INS breakpoint INV breakpoint (to 3': 325bp) INS breakpoint

(CSV sequence length: 1836bp)

Child 5' Sanger trace: TCACCTATTAACTAGAGTCACAGTCAACC……TTTTGTTGGGGA (762bp)

(728bp) TCAAGTGAAGTTCT…CTTTGAAGGTGCCTAAGTGACATCAT Child 3' Sanger trace

■ Sanger trace raw evidences:

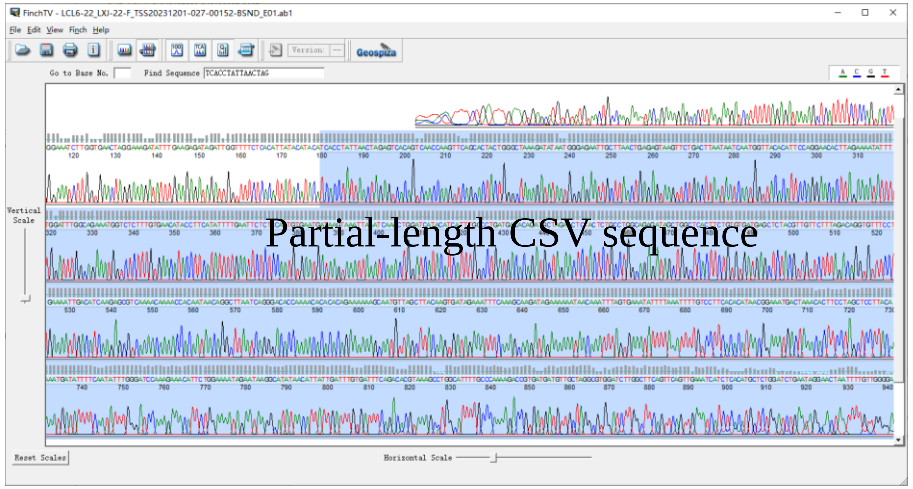

Child 5' primer Sanger trace

The CSV sequence in Sanger trace is marked in Blue

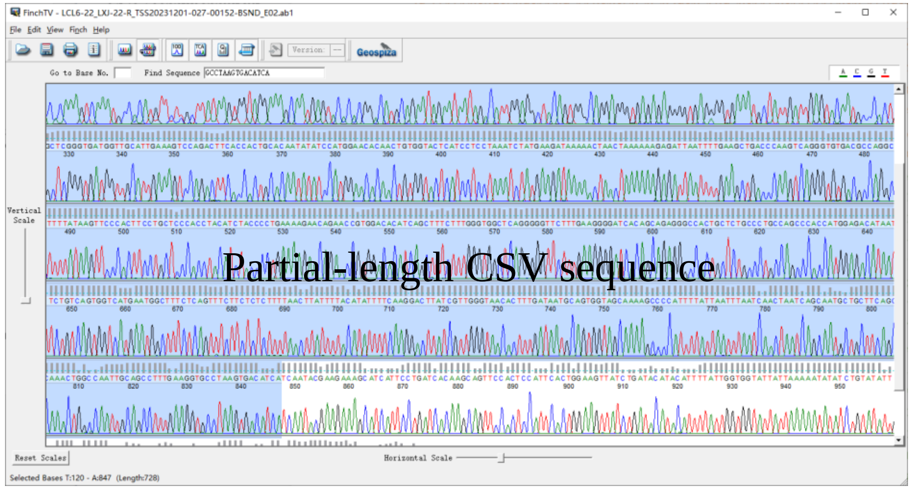

Child 3' primer sanger trace (reverse complement sequence)

■ Conclusion:

Sanger trace confirmed all the subcomponents' breakpoints, therefore, it is a true-positive CSV

Sanger sequencing results for ID CSV-23 chr10-57497185-57498224-INS+DEL

■ Sanger trace alignment evidences:

5’ Upstream (15bp) (1053bp) Downstream (15bp) 3’

Reference: CCAAACACCACATAT | TCTCACTCATAAGTGGGAGTTGAACAATAA… TTCAACTGTAAAACTAGGATAGCAA | AATCTATGTCATATT

CSV sequence: CCAAACACCACATAT | ATAGATCACAAATTTTGTGTAGTTTGTCA….AAAAAAATGATAAGAAAAAAGAAATG | AATCTATGTCATATT

INS breakpoint DEL breakpoint

(CSV sequence length: 301bp)

Child 5’ Sanger trace CCAAACACCACATATATAGATCACAAATTTTGTGTAGTTTGTCAATAC….AAAAAAATGATAAGAAAAAAGAAATGAATCTATGTCATATT (301bp)

■ Sanger trace raw evidences:

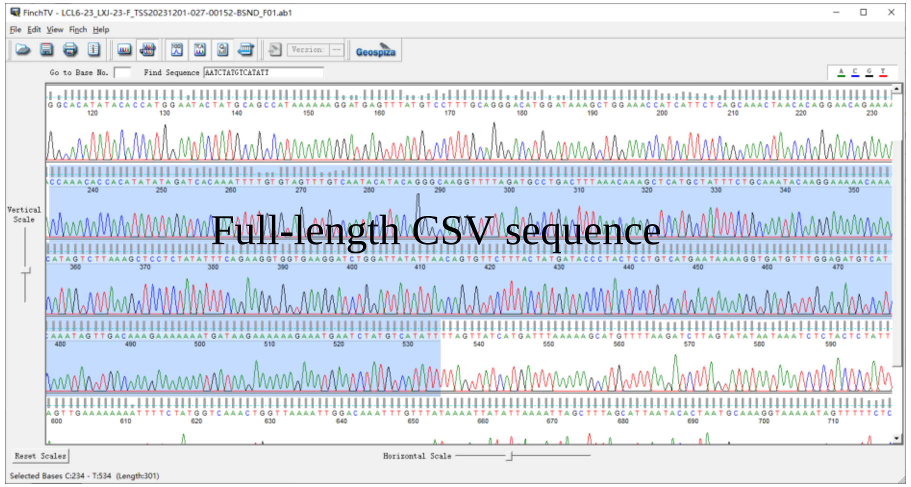

The CSV sequence in Sanger trace is marked in Blue

Child 5’ primer Sanger trace

■ Conclusion:

Sanger trace confirmed all the subcomponents’ breakpoints, therefore, it is a true-positive CSV

Sanger sequencing results for ID CSV-24 chr14-37300397-37302023-INS+DEL

- Sanger trace alignment evidences:

5’ Upstream (15bp) (1648p) Downstream (15bp) 3’

Reference: TTGGATTATAATCTA | ATACTATGTTATTTGTGTTGTTGCTTG…CATTCCTTTGTATAAAGGATATCTTGGTTA | CTTCTATGTTTGGTG

CSV sequence: TTGGATTATAATCTA | TTTTTTTTTTCTACATTTAGTCTTAACTTT……CTAGGGTTTACTCATCTGACAAAGGG | CTTCTATGTTTGGTG

INS breakpoint DEL breakpoint

(CSV sequence length: 1302bp)

Child 5’ Sanger trace: TTGGATTATAATCTATTTTTTTTTT (25bp)

Child 3’ Sanger trace: (811bp) GGCTGCATAGTATTCCATGG…TCTAGGGTTTACTCATCTGACAAAGGGCTTCTATGTTTGGTG

- Sanger trace raw evidences:

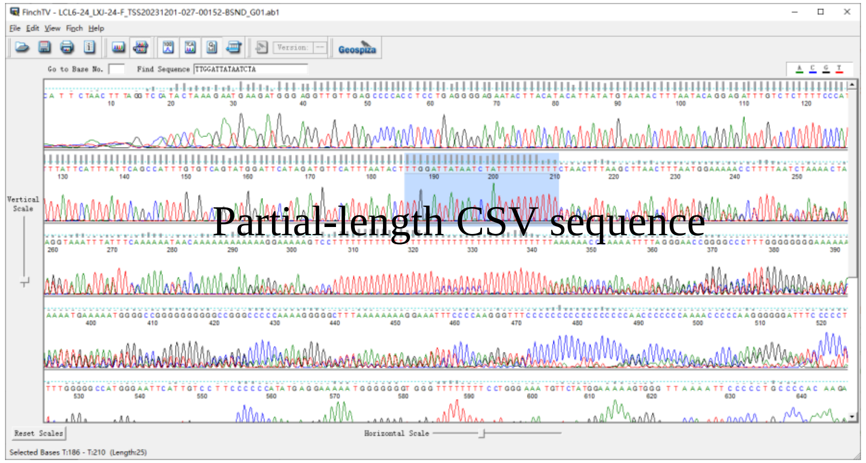

Child 5’ primer Sanger trace

The CSV sequence in Sanger trace is marked in Blue

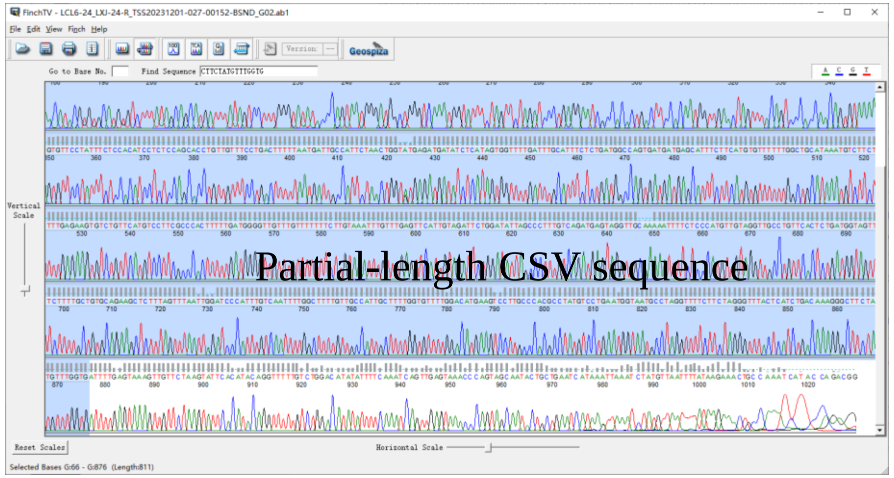

Child 3’ primer sanger trace (reverse complement sequence)

- Conclusion:

Sanger trace confirmed all the subcomponents’ breakpoints, therefore, it is a true-positive CSV

Sanger sequencing results for ID CSV-25 chr14-65375820-65376575-DEL+INV+DEL

- Sanger trace alignment evidences:

5’ Upstream (15bp) (766bp) Downstream (15bp) 3’

Reference: CTGTGGCTGGGTGCA GTGGCTCATGCCTGTAATCCCAGCACTT…ATTATAGGCGCCCACCACCATGCCTGGC TAGTTTTTGTGTTTT

CSV sequence: CTGTGGCTGGGTGCA AAAACAATCTGTGGATGAAACGAGAGATTTT……AGGCATGCGCCACCATGCCCAGT TAGTTTTTGTGTTTT

DEL breakpoint INV breakpoint DEL breakpoint

(CSV sequence length: 501bp)

Child 5’ Sanger trace CTGTGGCTGGGTGCAAAAACAATCTGTGGATGAAACGAGAGATTTTC….ATTACAGGCATGCGCCACCATGCCCAGTTAGTTTTTGTGTTTT (501bp)

- Sanger trace raw evidences:

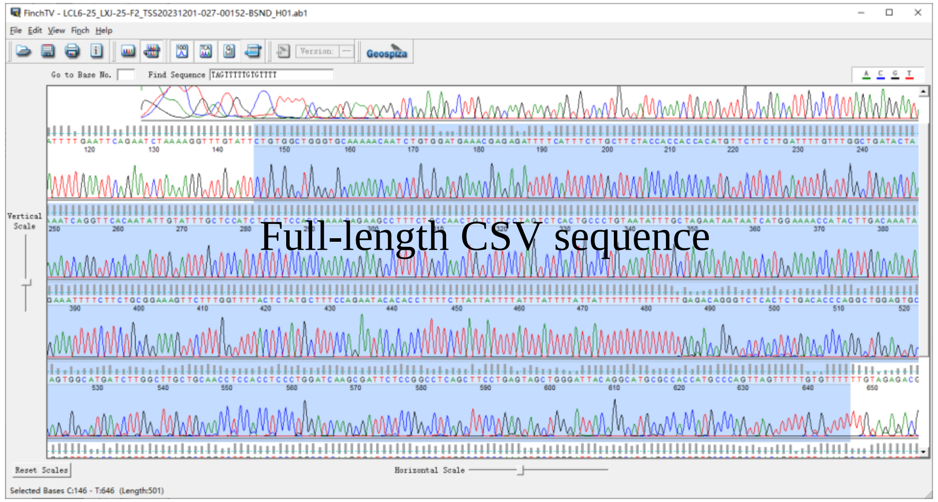

Child 5’ primer Sanger trace

The CSV sequence in Sanger trace is marked in Blue

- Conclusion:

Sanger trace confirmed all the subcomponents’ breakpoints, therefore, it is a true-positive CSV

Sanger sequencing results for ID CSV-29 chr16-82130592-82130903-INS+DEL

■ Sanger trace alignment evidences:

5’ Upstream (15bp) (1053bp) Downstream (15bp) 3’

Reference: GCGTGTGTATATATA | TCTCACTCATAAGTGGGAGTTGAACAATAA… TTCAACTGTAAAACTAGGATAGCAA | TATATATGTGTGCGT

CSV sequence: GCGTGTGTATATATA | CACACATGTATATATATGTATATATGT….ATACACACGTGTGGATATACACGTGTGTA | TATATATGTGTGCGT

INS breakpoint DEL breakpoint

(CSV sequence length: 92bp)

Child 5’ Sanger trace: GCGTGTGTATATATACACACATGTATATATATGTATATATGTGTATATATACACACGTGTGGATATACACGTGTGTATATATATGTGTGCGT (92bp)

■ Sanger trace raw evidences:

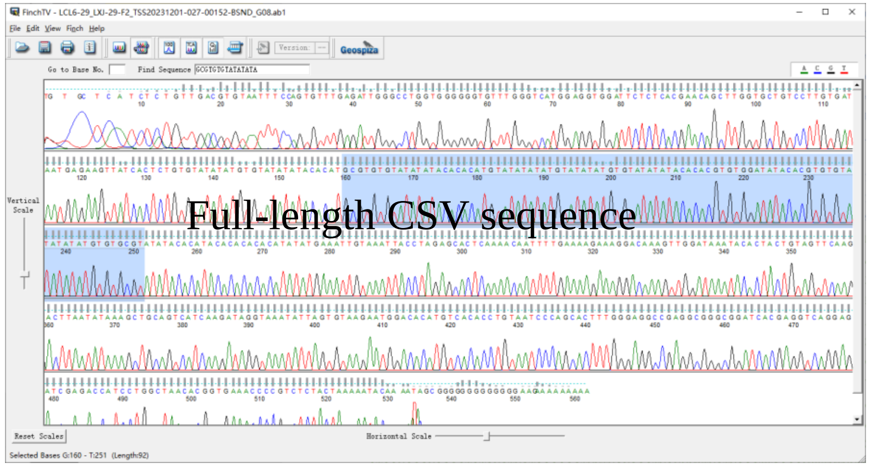

The CSV sequence in Sanger trace is marked in Blue

Child 5’ primer Sanger trace

■ Conclusion:

Sanger trace confirmed all the subcomponents’ breakpoints, therefore, it is a true-positive CSV

Sanger sequencing results for ID CSV-33 chrX-104069779-104082053-INS+DEL

■ Sanger trace alignment evidences:

5’ Upstream (15bp) (1648p) Downstream (15bp) 3’

Reference: AATGAGACTTTTTTT ATACTATGTTATTTGTGTTGTTGCTTG…CATTCCTTTGTATAAAGGATATCTTGGTTA GAGAGTTCTGTAGAT

CSV sequence: AATGAGACTTTTTTT TAAATTTTTTTTTATTATACTTTAAGTTTT……GATTCGTTTTGCCAGTATTTTGGAGT GAGAGTTCTGTAGAT

INS breakpoint DEL breakpoint

(CSV alternative sequence: 2440bp)

Child 5’ Sanger trace: AATGAGACTTTTTTTTAAATTTTTTT…..CCATTCTAACTGG (715bp)

(628bp) TGTTGTTGGTGTATAAGAA…TATTTTGGAGTGGAGAGTTCTGTAGAT Child 3’ Sanger trace

■ Sanger trace raw evidences:

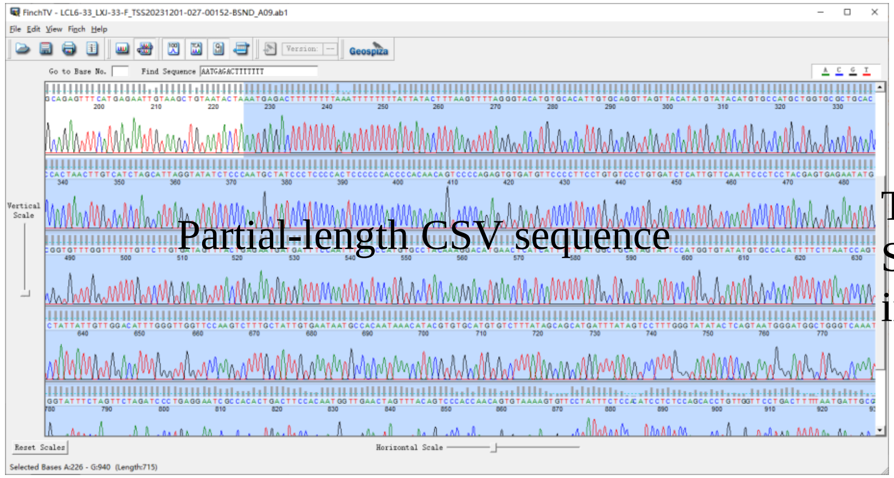

Child 5’ primer Sanger trace

The CSV sequence in Sanger trace is marked in Blue

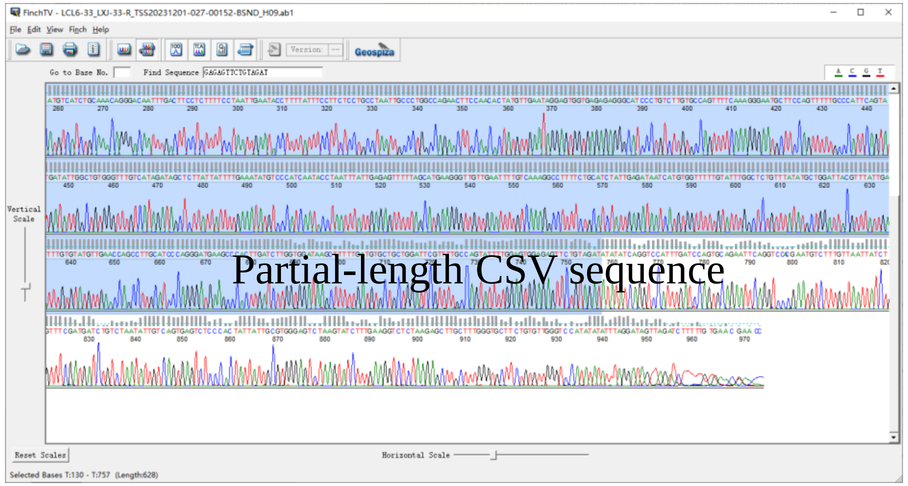

Child 3’ primer sanger trace (reverse complement sequence)

■ Conclusion:

Sanger trace confirmed all the subcomponents’ breakpoints, therefore, it is a true-positive CSV

Supplementary File 2. Verification of the 32,549bp deletion in chromosome 1

HiFi long reads

chr1:152,567,581-152,633,831

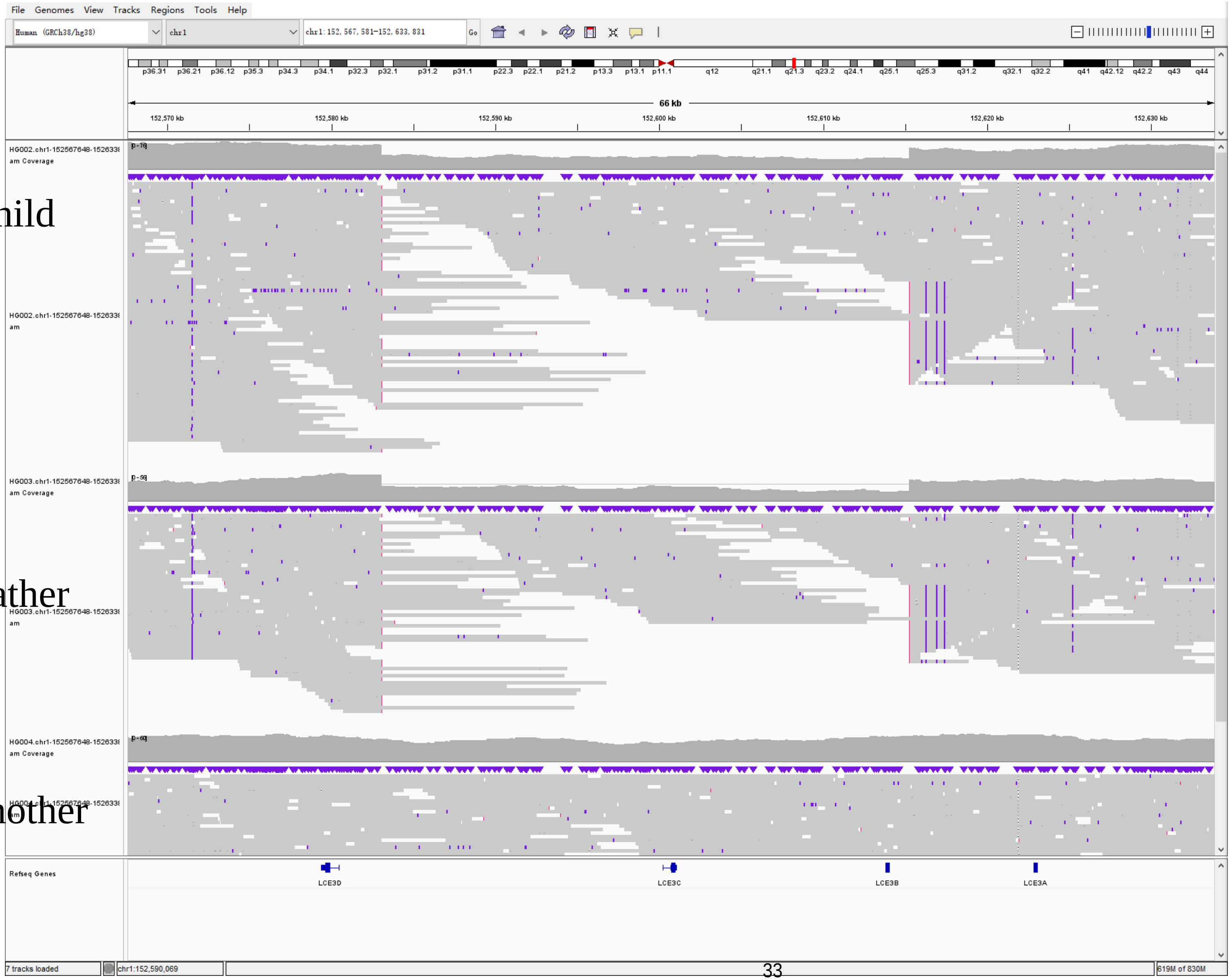

HG002, child

DEL: 0/1

HG003: father

DEL: 0/1

HG004, mother

DEL: 0/0

	SVision-pro	Sniffles2
HG002, child	0/1	1/1
HG003: father	0/1	1/1
HG004, mother	0/0	0/0

HiFi long reads

chr1:152,567,581-152,633,831

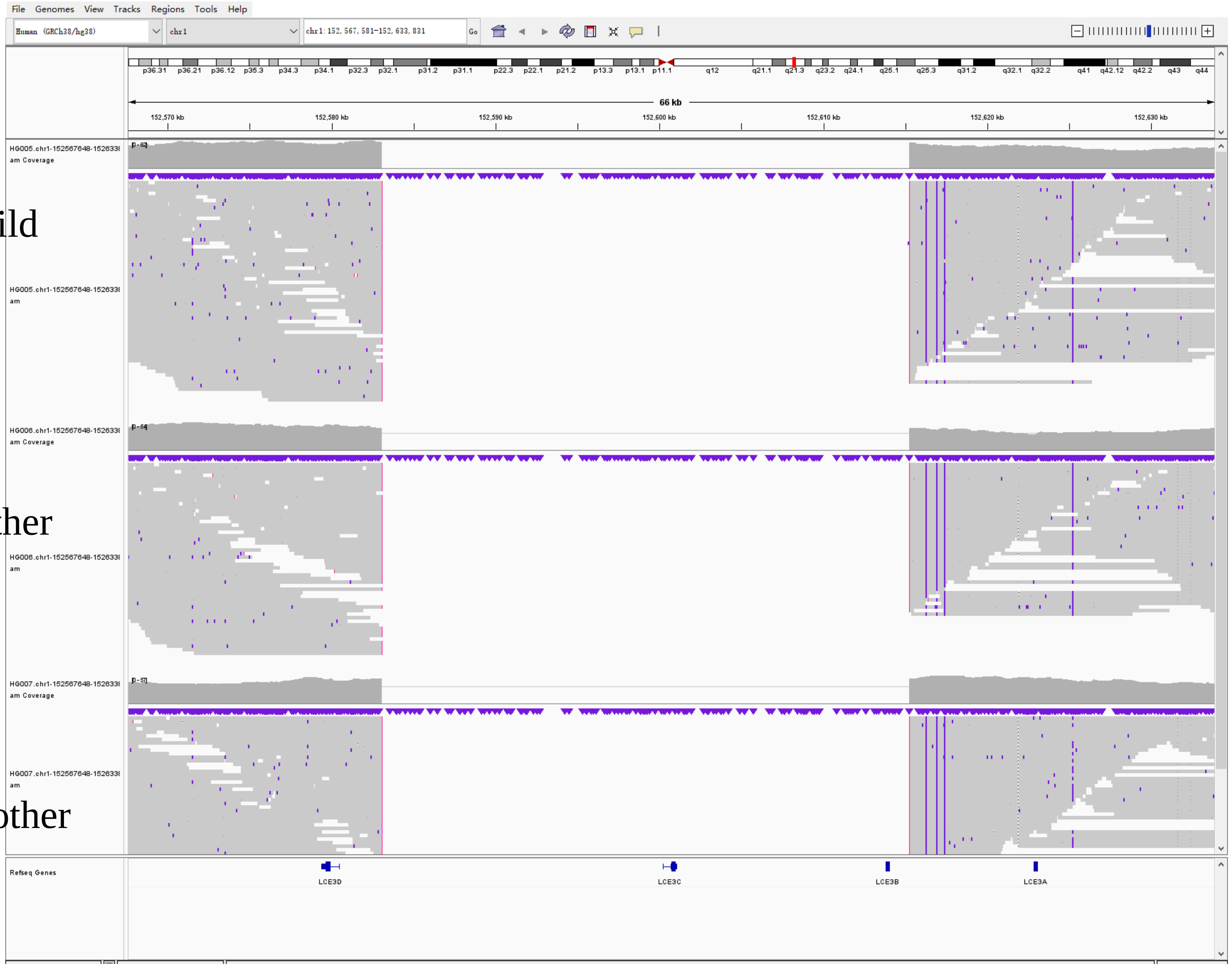

	SVision-pro	Sniffles2
HG005, child<br>DEL: 1/1	1/1	Not called
HG006: father<br>DEL: 1/1	1/1	Not called
HG007, mother<br>DEL: 1/1	1/1	Not called

HiFi long reads

chr1:152,567,581-152,633,831

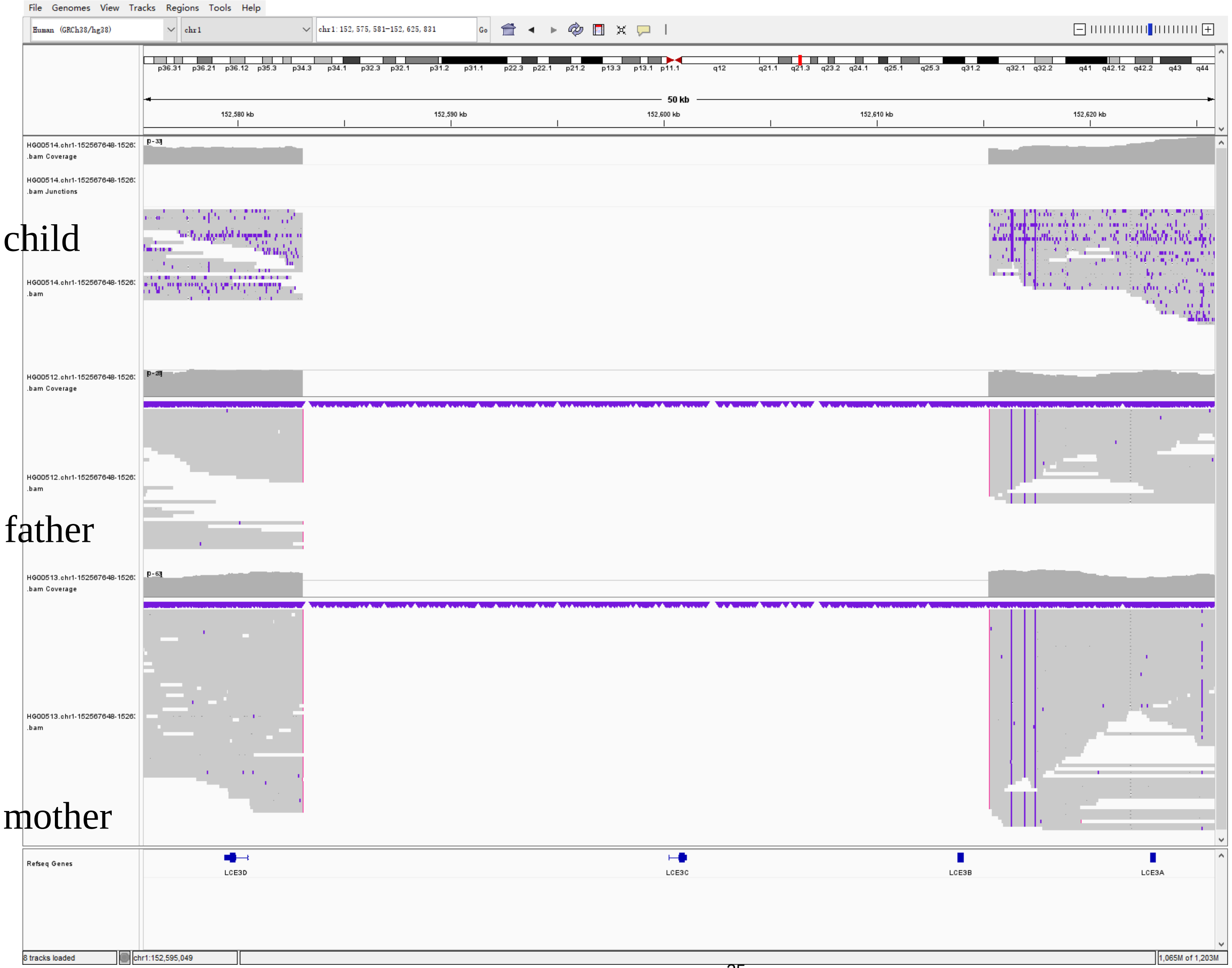

	SVision-pro	Sniffles2
HG00514, child<br>DEL: 1/1	1/1	Not called
HG00512: father<br>DEL: 1/1	1/1	Not called
HG00513, mother<br>DEL: 1/1	1/1	Not called

HiFi long reads

chr1:152,567,581-152,633,831

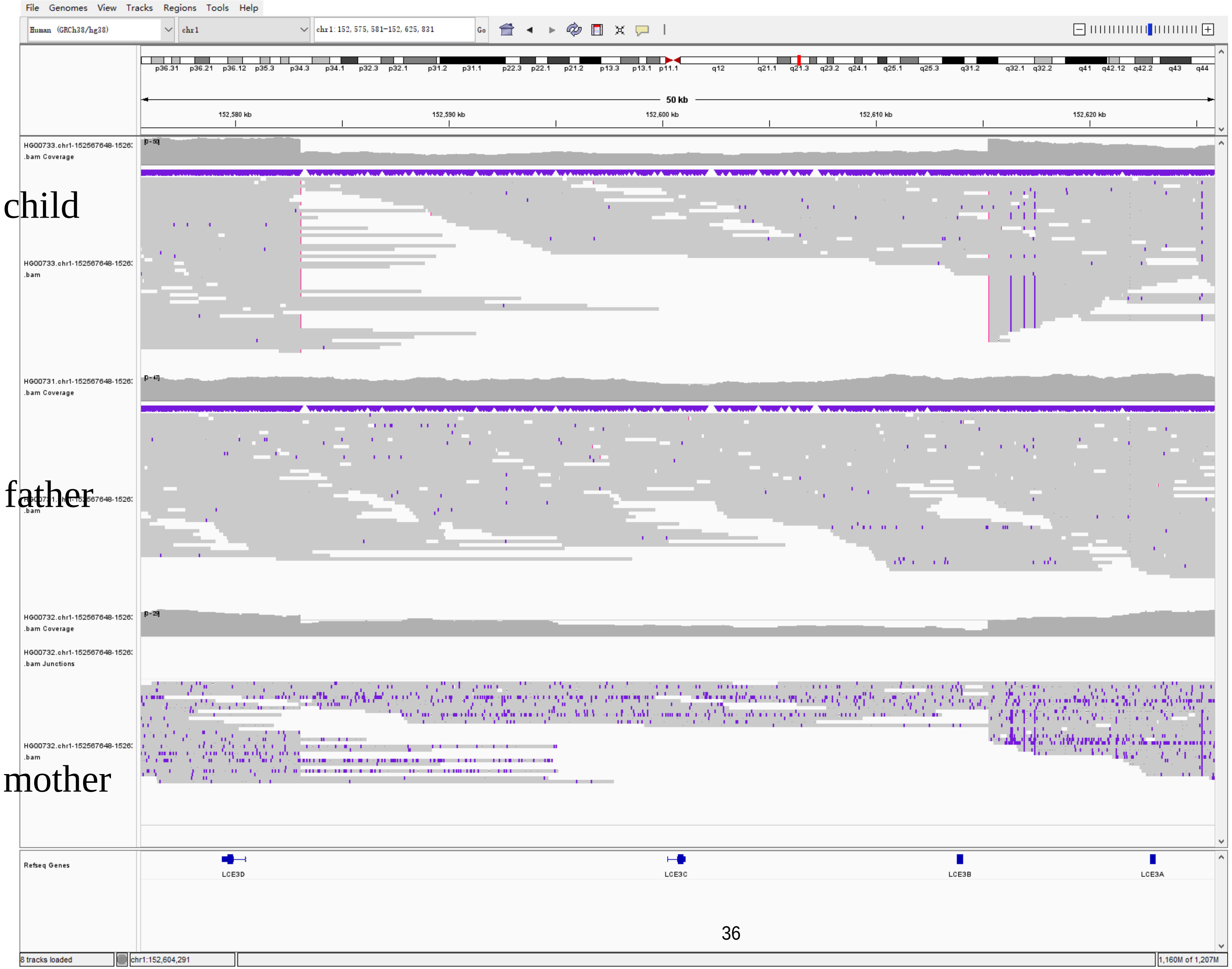

	SVision-pro	Sniffles2
HG00733, child<br>DEL: 0/1	0/1	1/1
HG00731: father<br>DEL: 0/0	0/0	0/0
HG00732, mother<br>DEL: 0/1	0/1	1/1

HiFi long reads

chr1:152,567,581-152,633,831

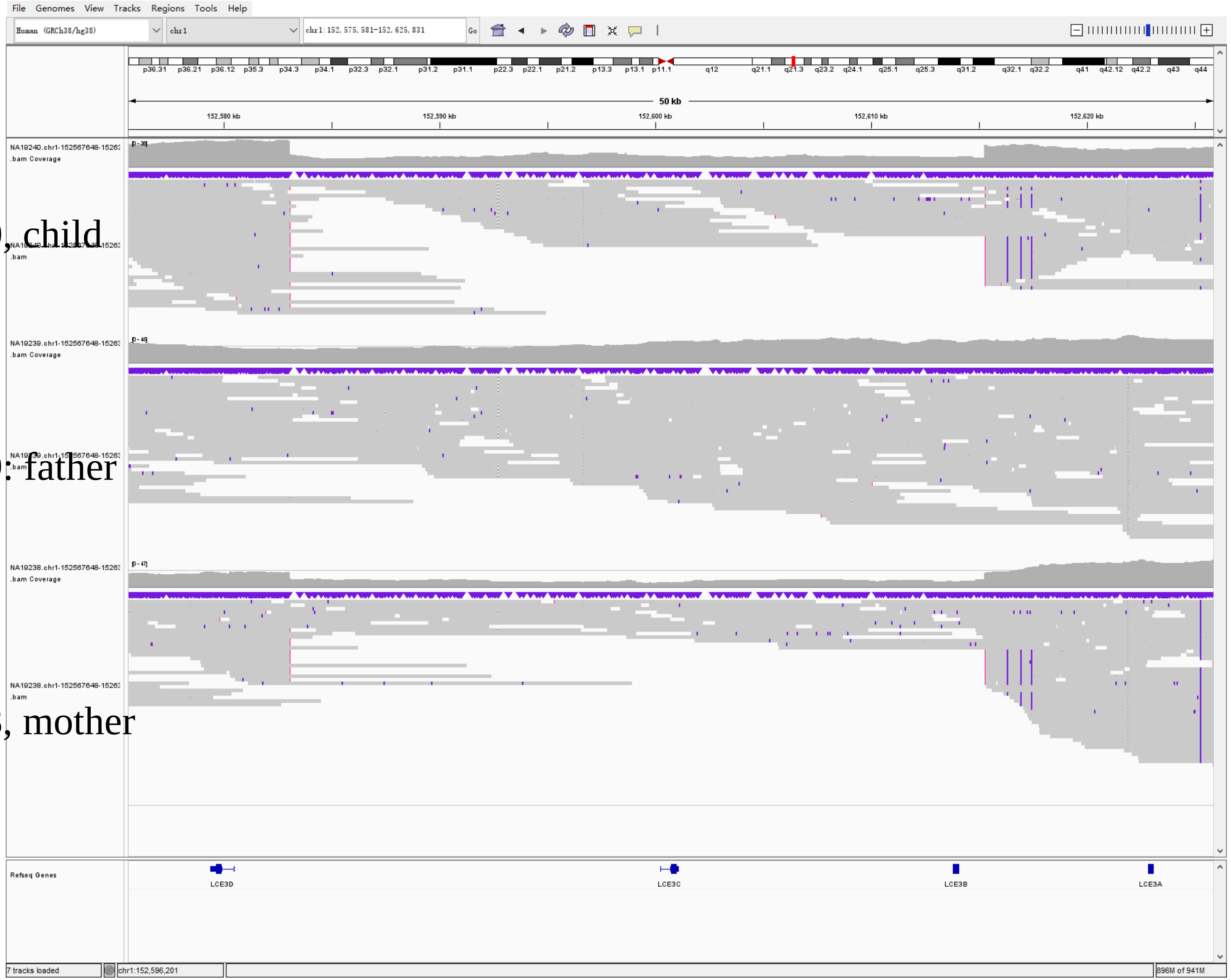

	SVision-pro	Sniffles2
NA19240, child<br>DEL: 0/1	0/1	1/1
NA19239: father<br>DEL: 0/0	0/0	0/0
NA19238, mother<br>DEL: 0/1	0/1	1/1

HiFi long reads

chr1:152,567,581-152,633,831

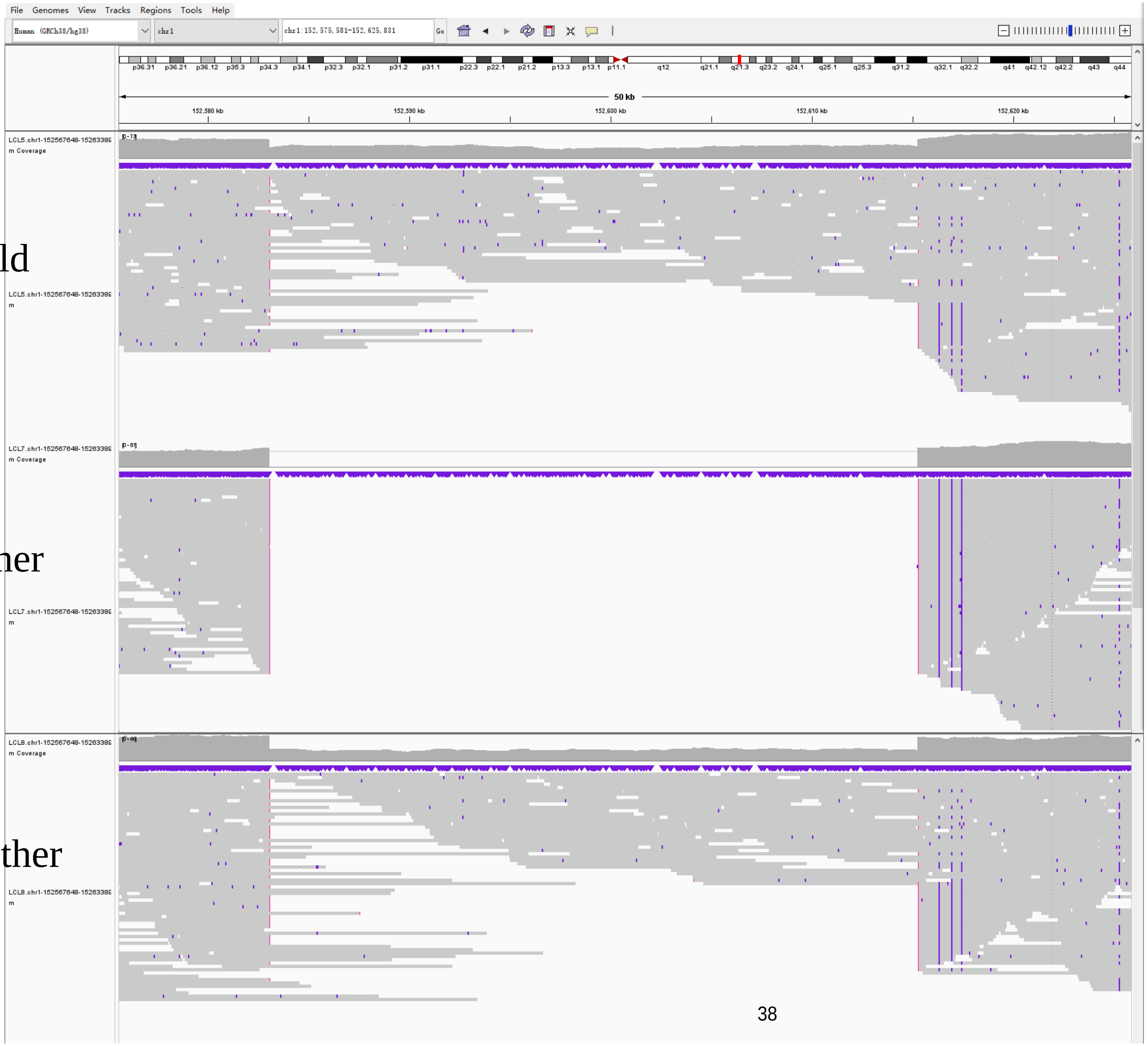

	SVision-pro	Sniffles2
LCL5, child<br>DEL: 0/1	0/1	1/1
LCL7: father<br>DEL: 1/1	1/1	./.
LCL8, mother<br>DEL: 0/1	0/1	1/1

Supplementary File 3. Verification of the complex locus at chr11:34,686,676

HiFi long reads

chr11:34,683,084-34,692,647

LCL5, child

INS: 0/1

INS+DEL: 0/1

LCL6, child

INS: 0/1

INS+DEL: 0/1

LCL7: father

INS: 0/1

INS+DEL: 0/1

LCL8, mother

INS: 1/1

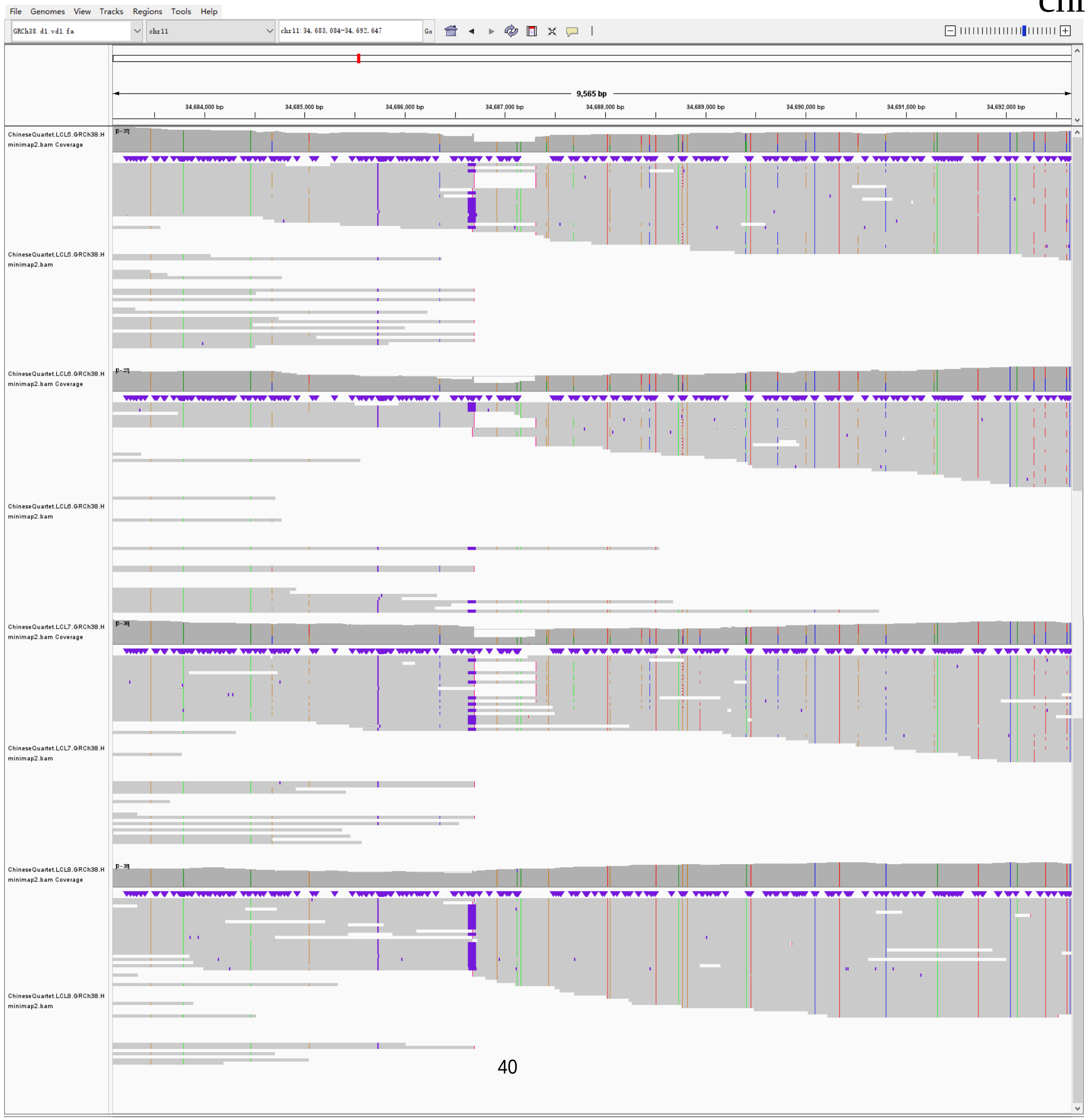

HiFi long reads

chr11:34,683,084-34,692,647

NA19238, mother

INS+DEL: 1/1

NA19239, father

INS : 0/1

INS+DEL: 0/1

NA19240, child

INS+DEL: 1/1

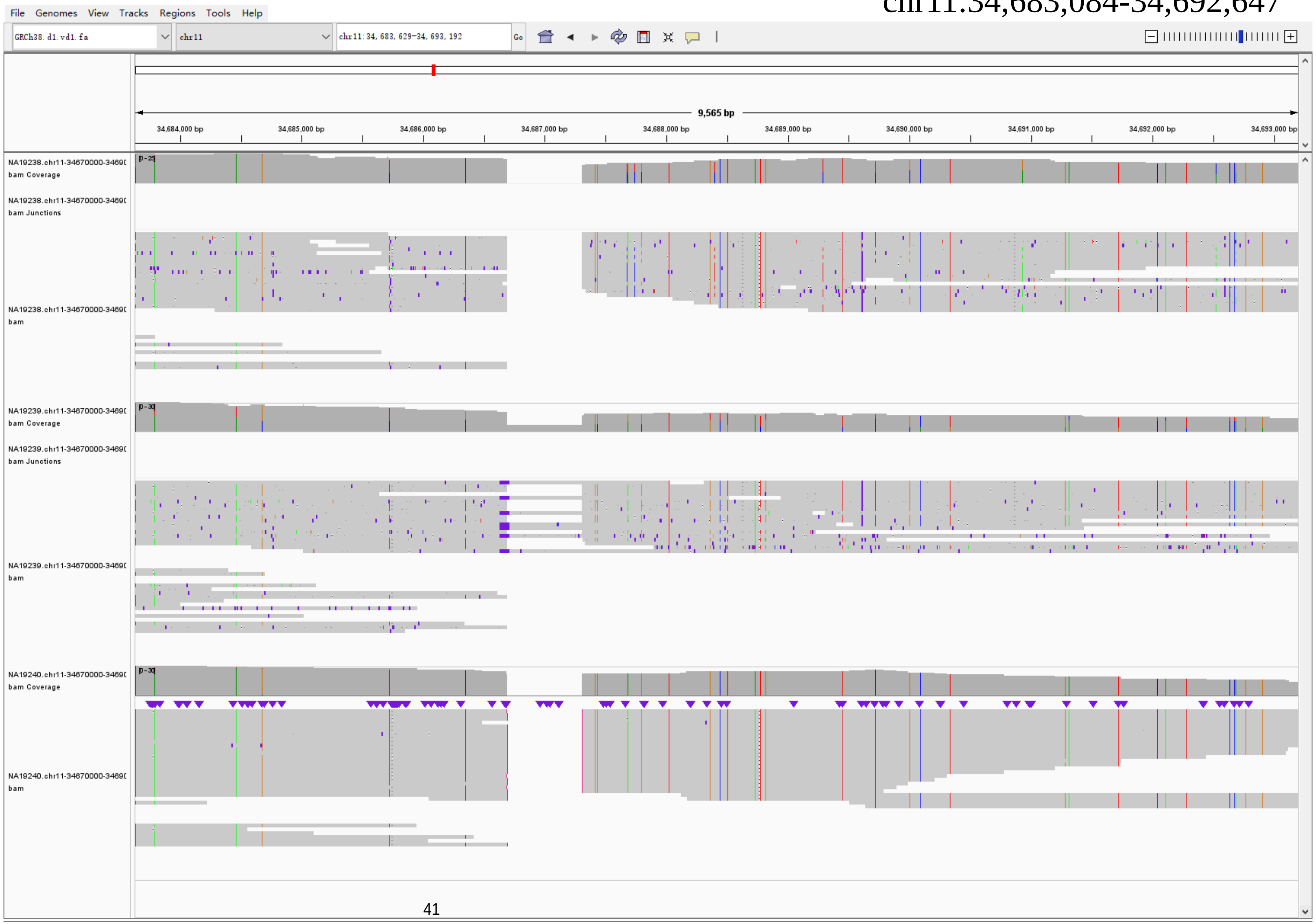

HiFi long reads

chr11:34,683,084-34,692,647

HG00512, father

INS: 1/1

HG00513, mother

INS : 1/1

HG00514, child

INS : 1/1

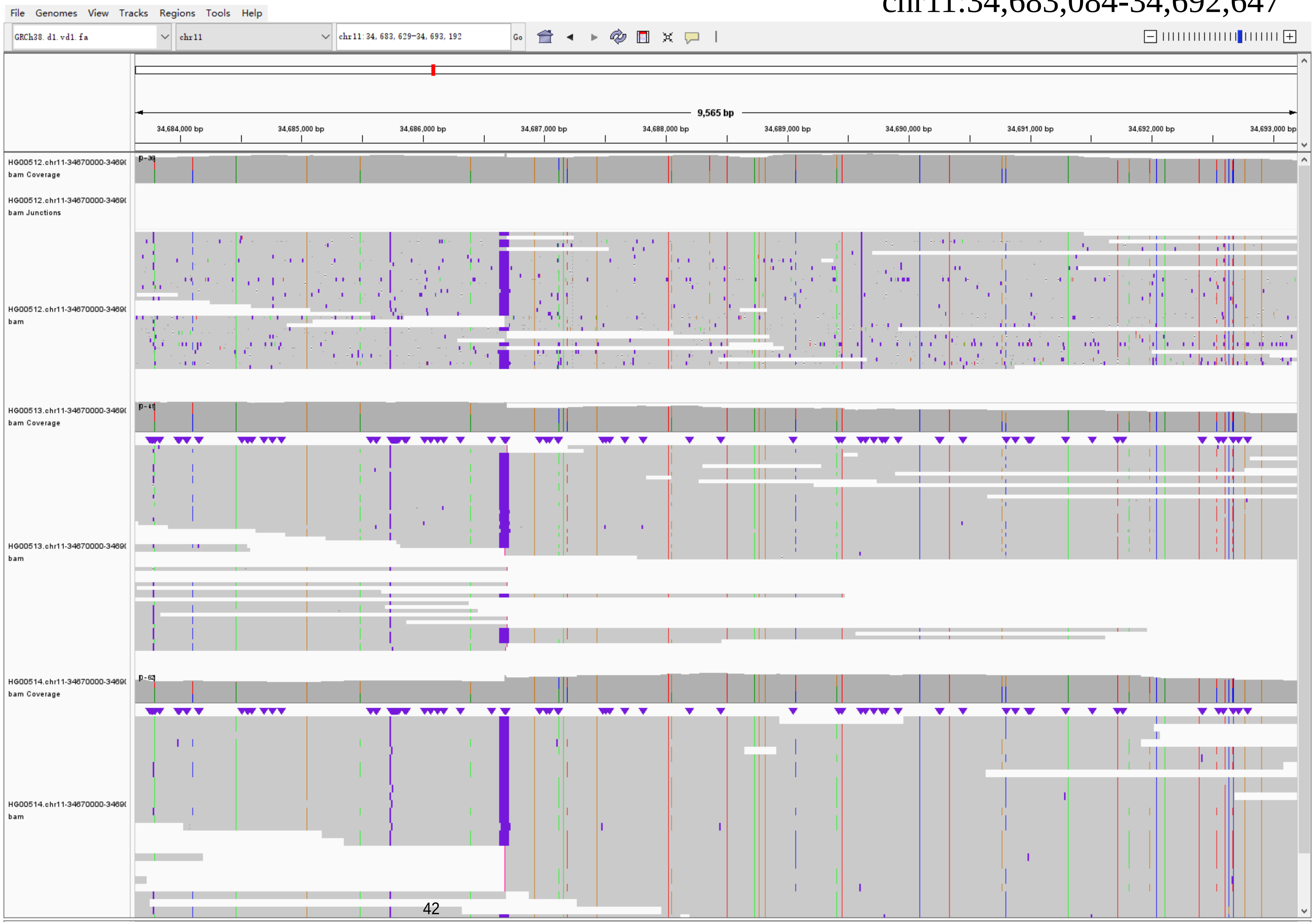

HiFi long reads

chr11:34,683,084-34,692,647

HG00731, father

INS: 0/1

INS+DEL: 0/1

HG00732: mother

INS: 0/1

INS+DEL: 0/1

HG00733, child

INS: 0/1

INS+DEL: 0/1

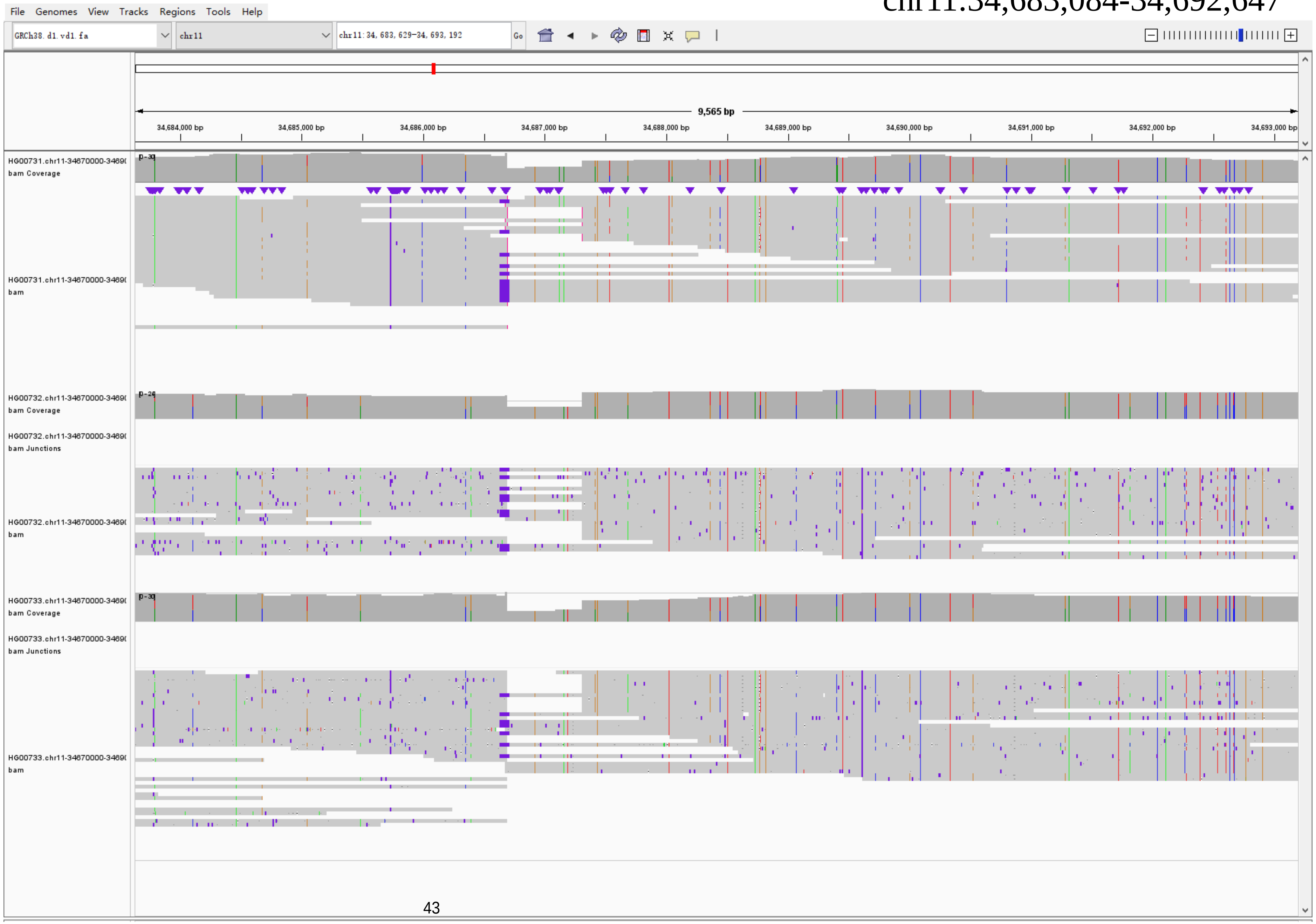

HiFi long reads

chr11:34,683,084-34,692,647

HG002, child

INS: 1/1

HG003: father

INS: 1/1

HG004, mother

INS: 0/1

INS+DEL: 0/1

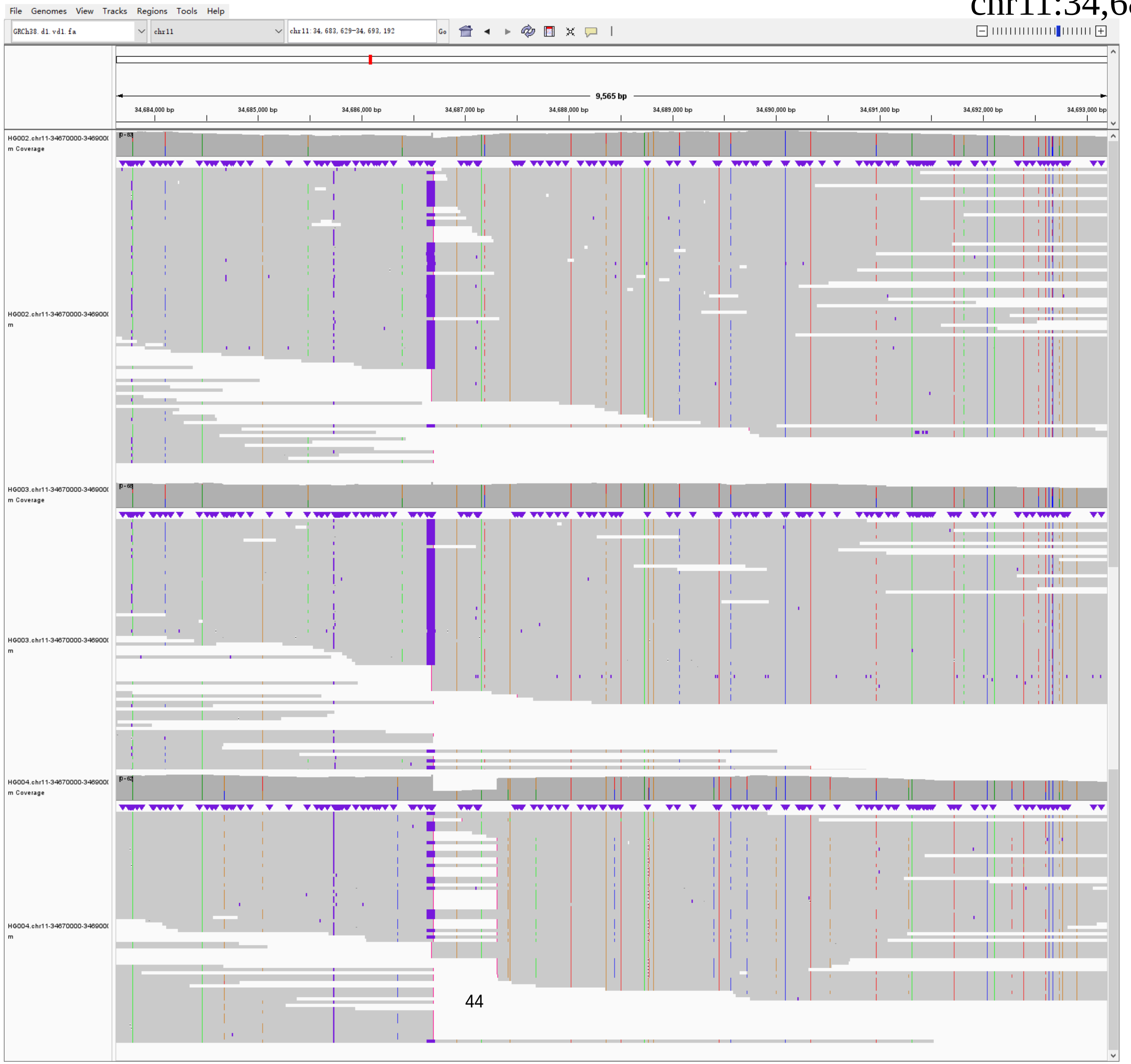

44

HiFi long reads

chr11:34,683,084-34,692,647

HG005, child

INS: 1/1

HG006: father

INS: 1/1

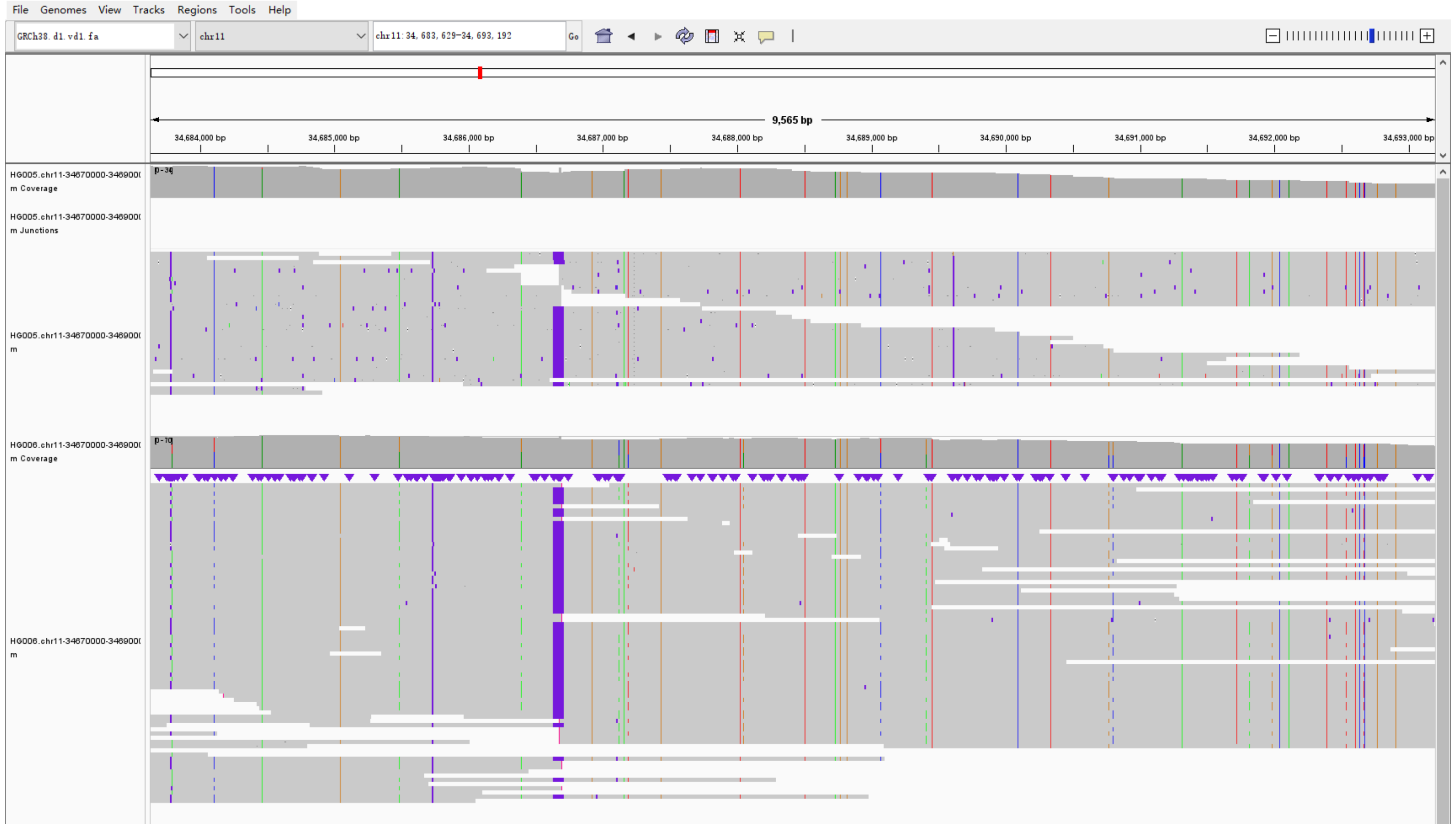

HG007, mother

INS: 1/1

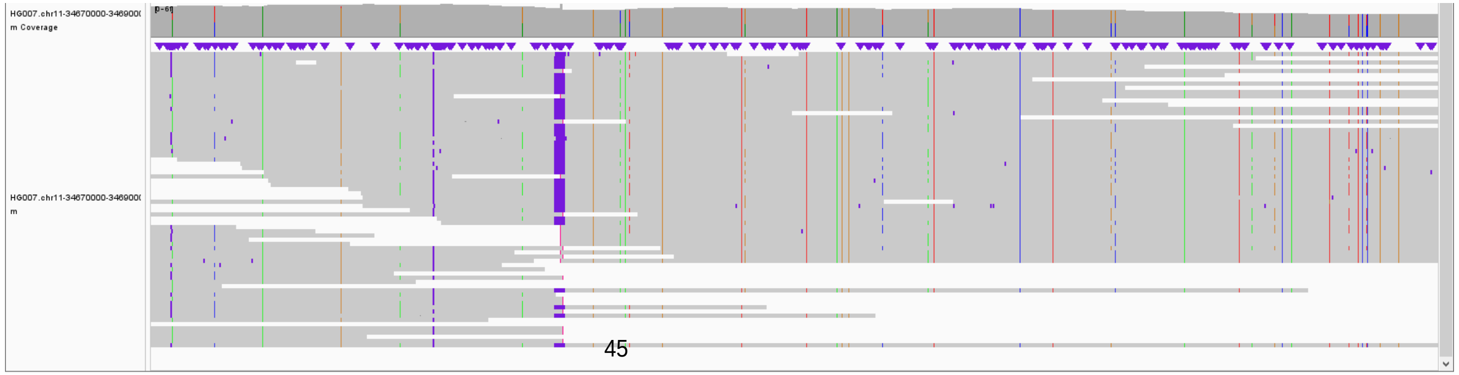

HGSVC2 published assemblies

chr11:34,683,084-34,692,647

NA19238, mother

INS+DEL: 1/1

NA19239, father

INS : 0/1

INS+DEL: 0/1

NA19240, child

INS+DEL: 1/1

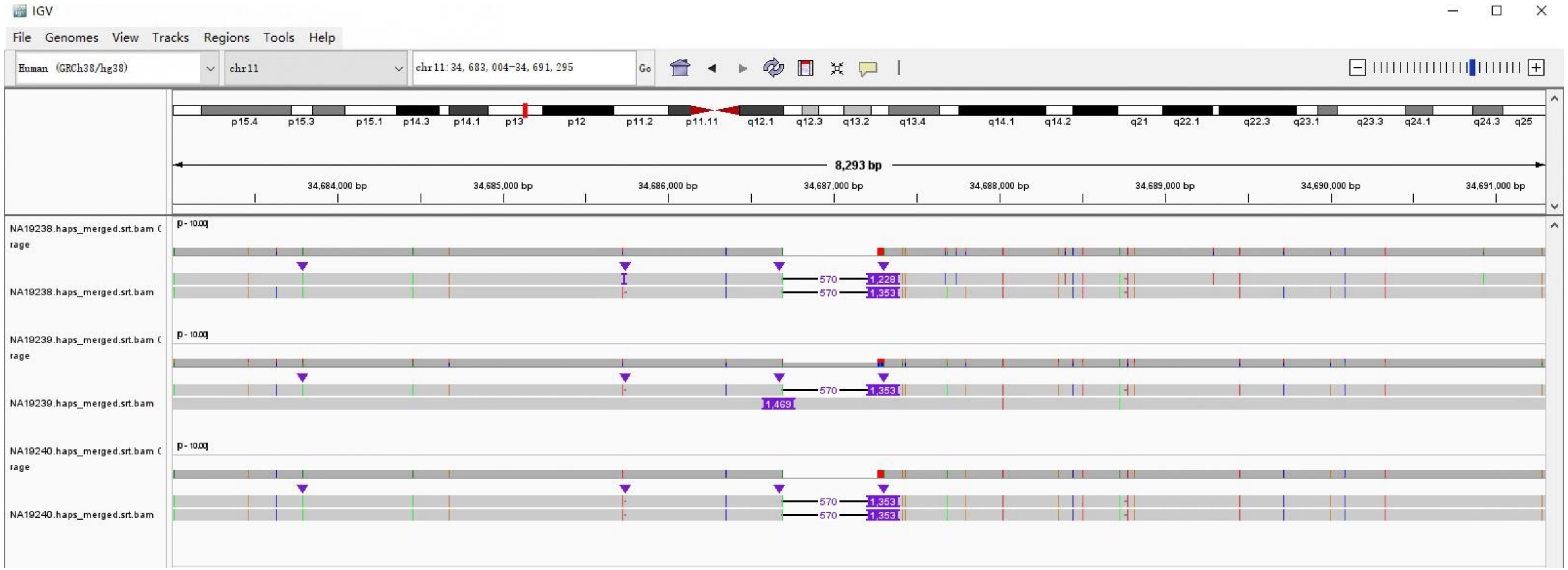

HGSVC2 published assemblies

chr11:34,683,084-34,692,647

HG00512, father

INS: 1/1

HG00513, mother

INS : 1/1

HG00514, child

INS : 1/1

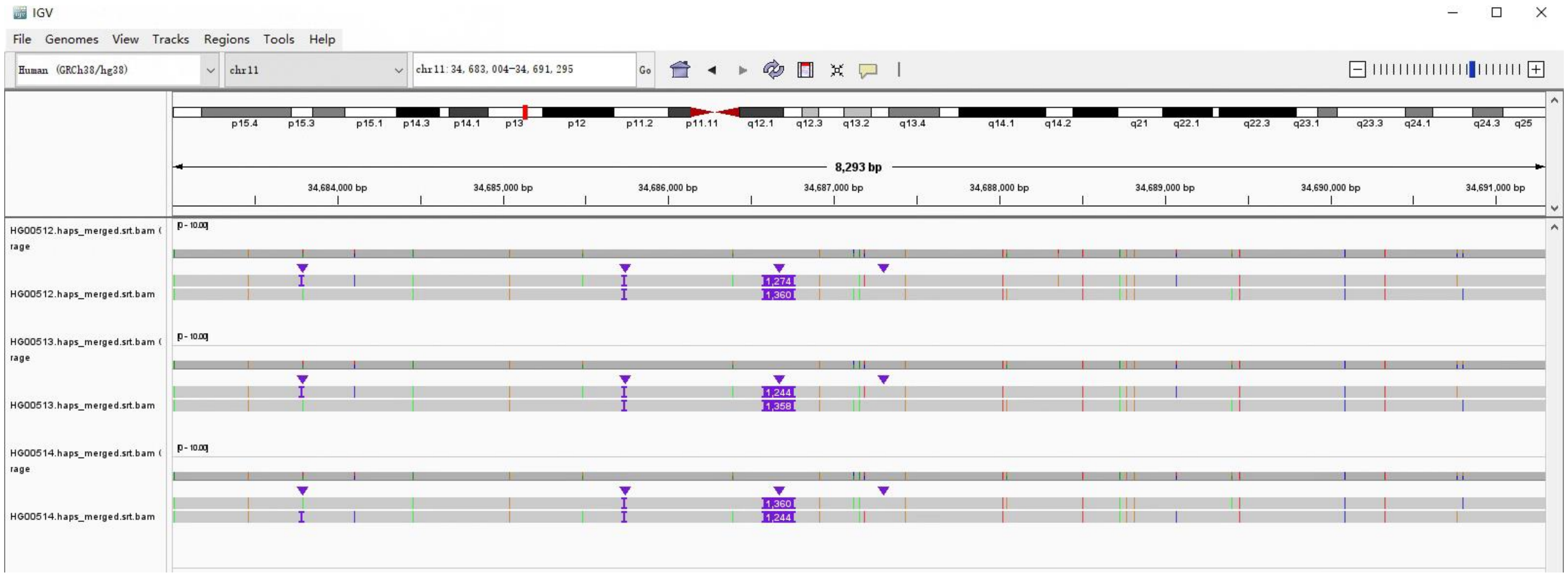

HGSVC2 published assemblies

chr11:34,683,084-34,692,647

HG00731, father

INS: 0/1

INS+DEL: 0/1

HG00732: mother

INS: 0/1

INS+DEL: 0/1

HG00733, child

INS: 0/1

INS+DEL: 0/1

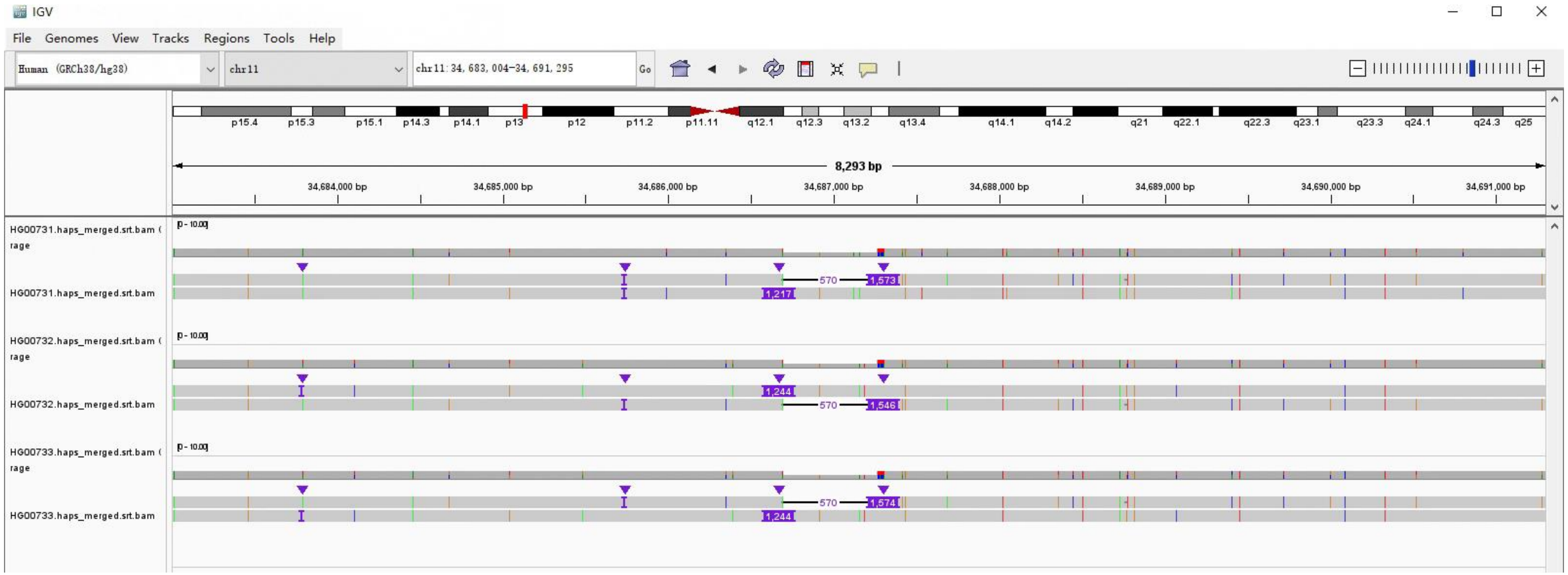

Supplementary File 4. Verification of 26 *de novo* SVs of SVision-pro

chr2 109199495 109199495 59 INS hg002-3-4 svision_pro

HG002

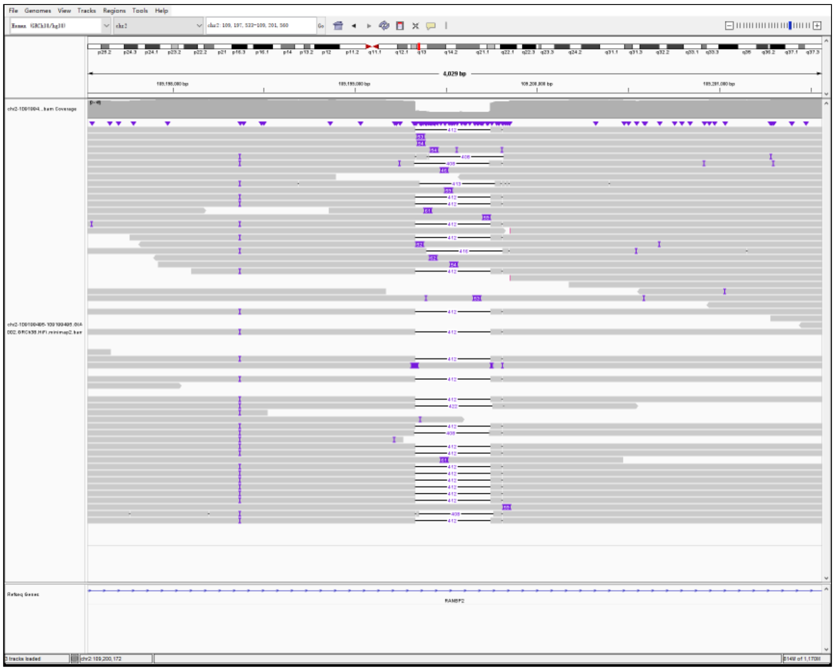

HG003

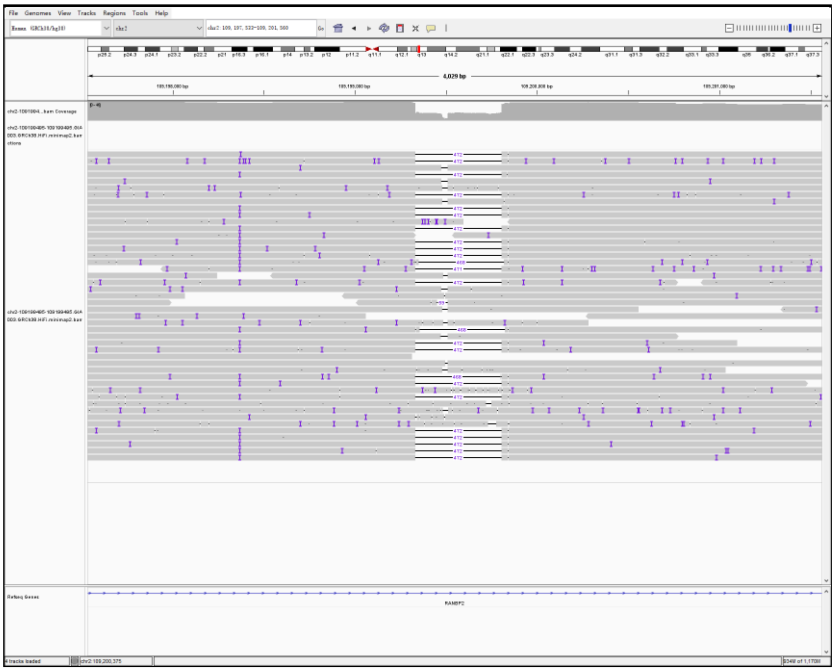

HG004

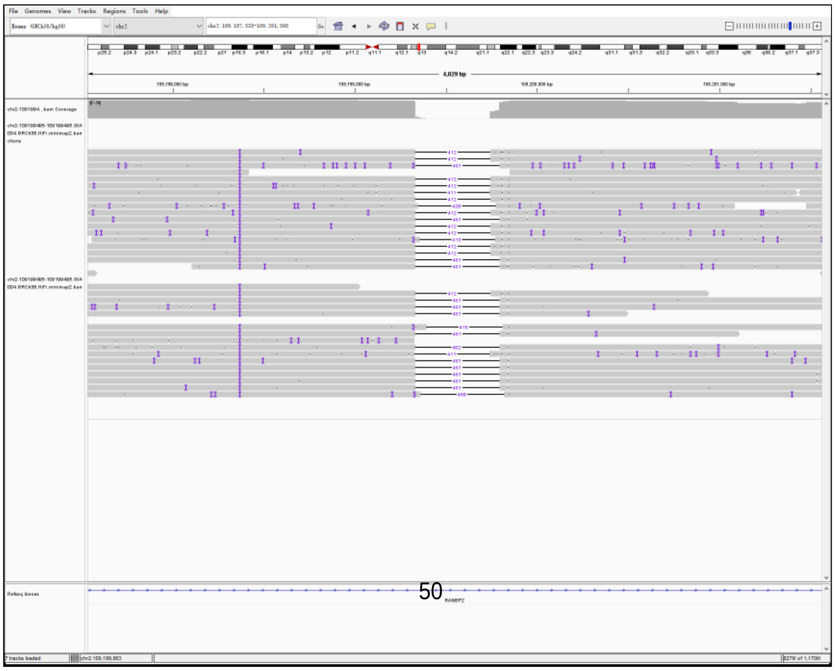

chr3	85552366	85552366	66	INS	hg002-3-4	svision_pro

HG002

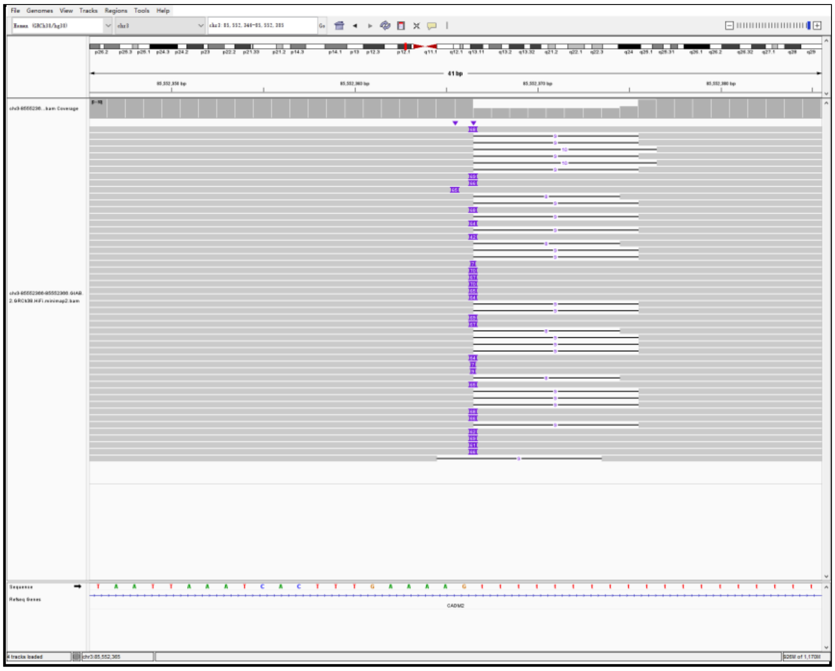

HG003

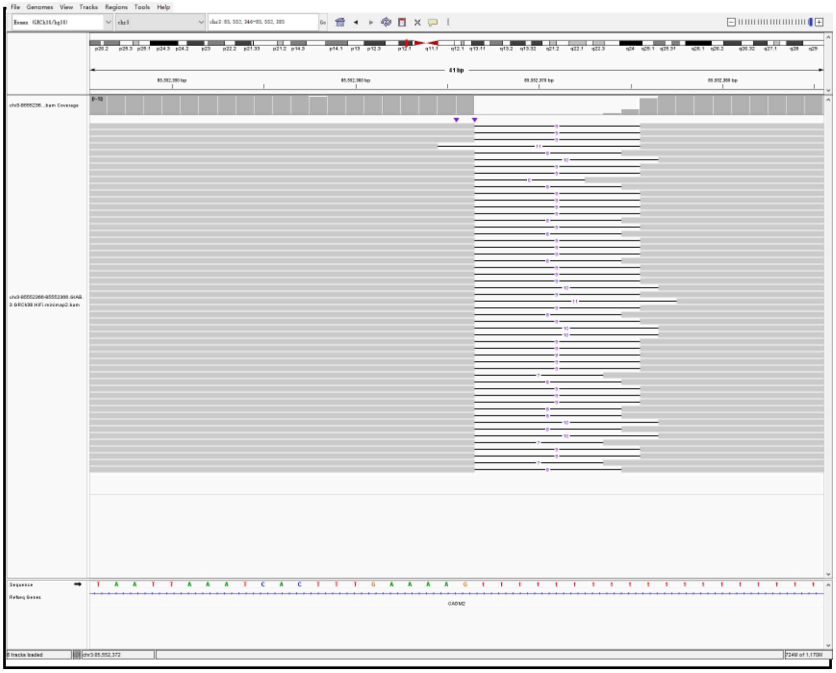

HG004

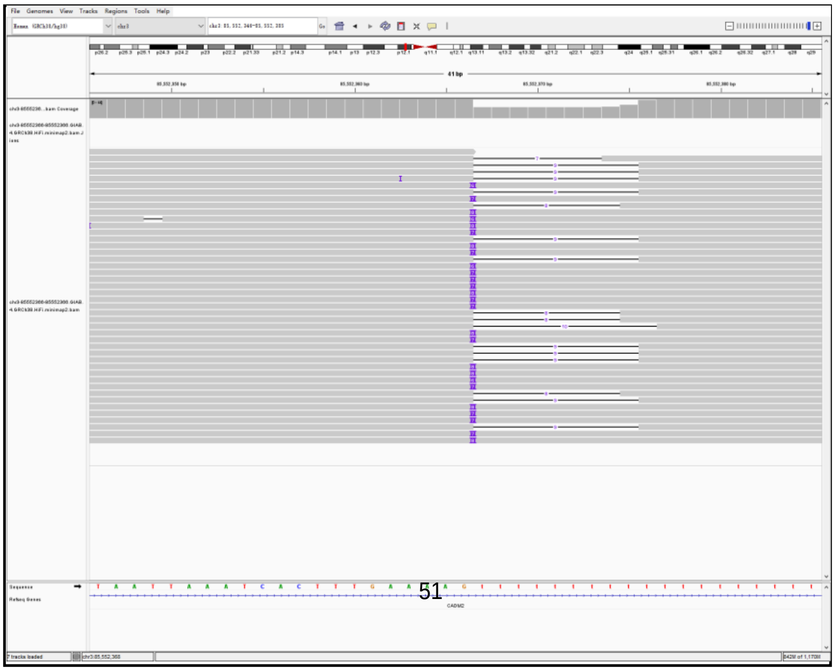

chr5 104502114 104502169 54 DEL hg002-3-4 svision_pro

HG002

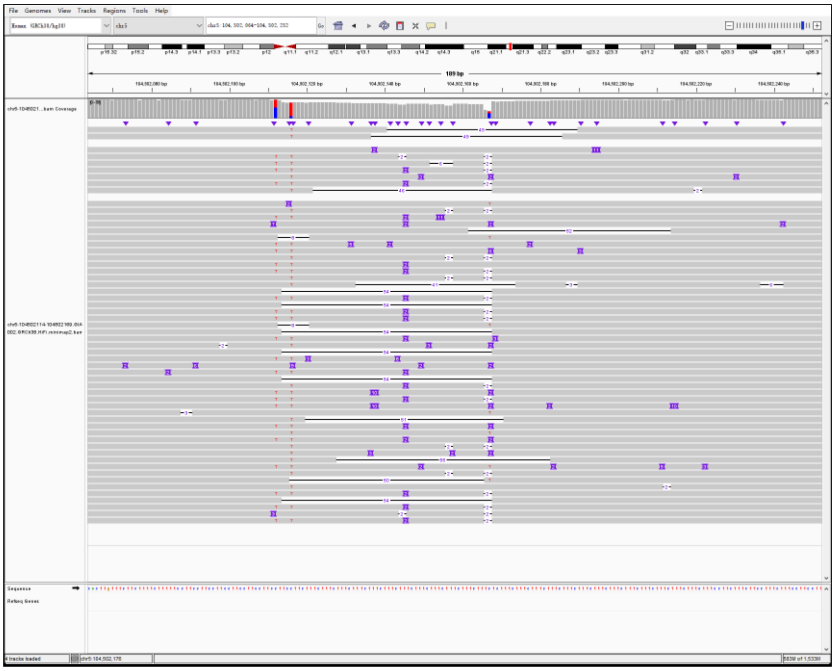

HG003

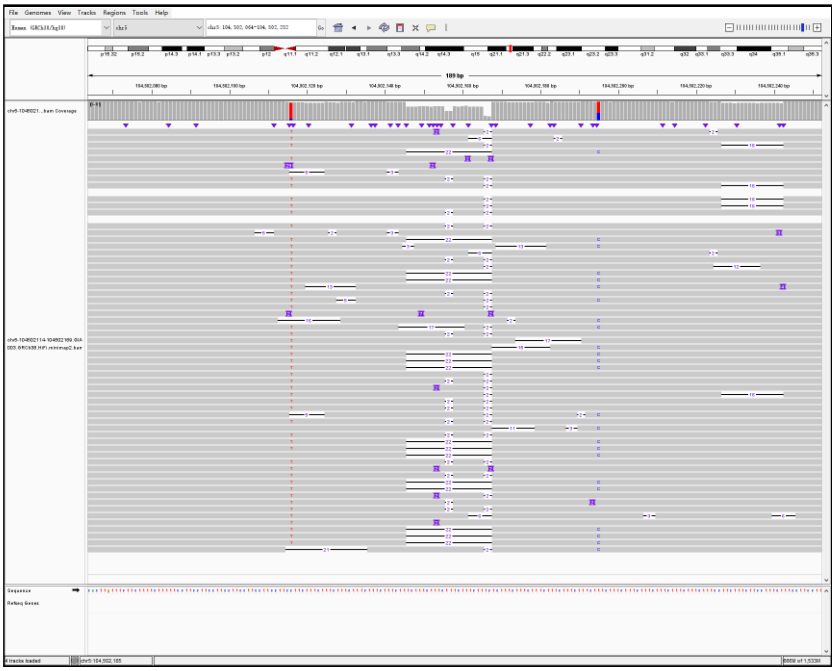

HG004

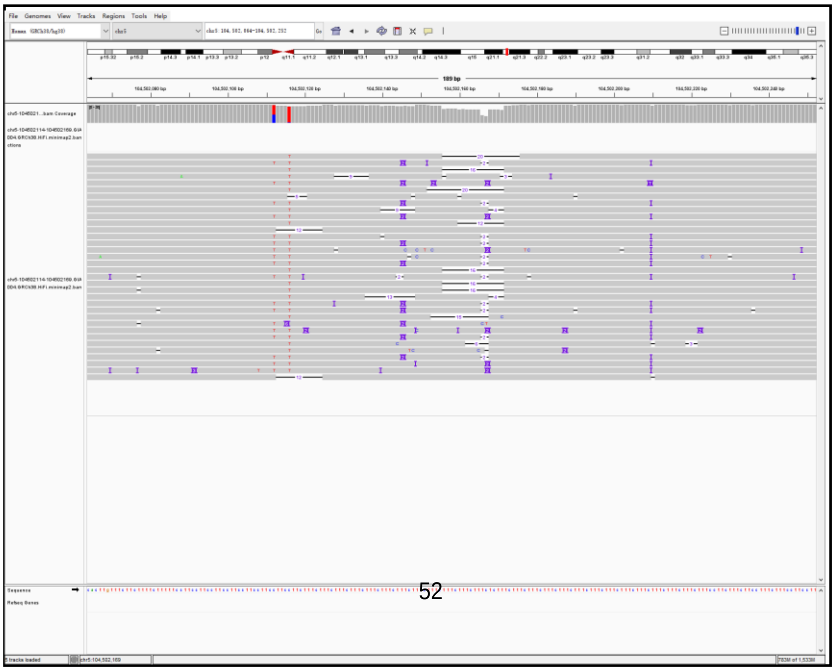

chr11 71584852 71584931 79 DEL hg002-3-4 svision_pro

HG002

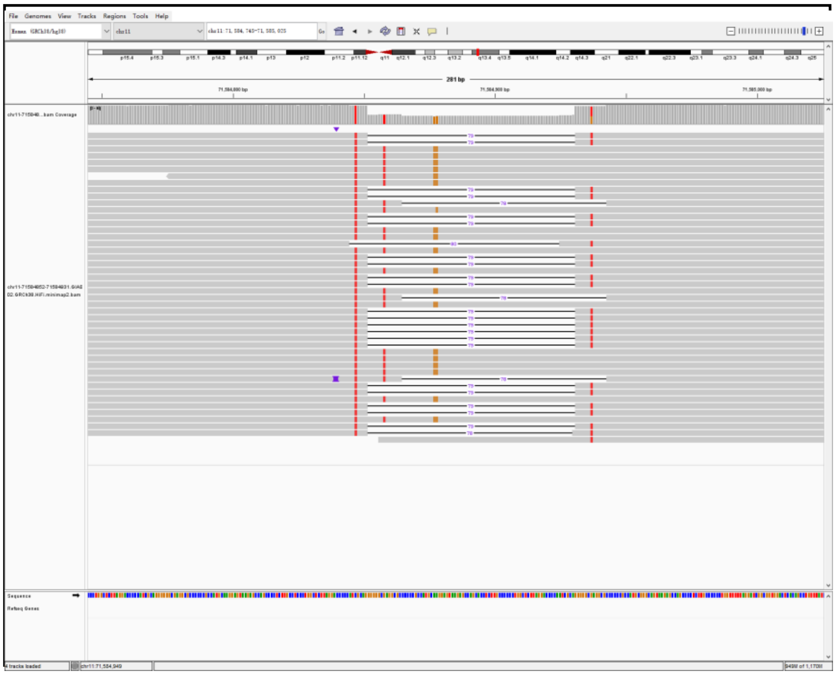

HG003

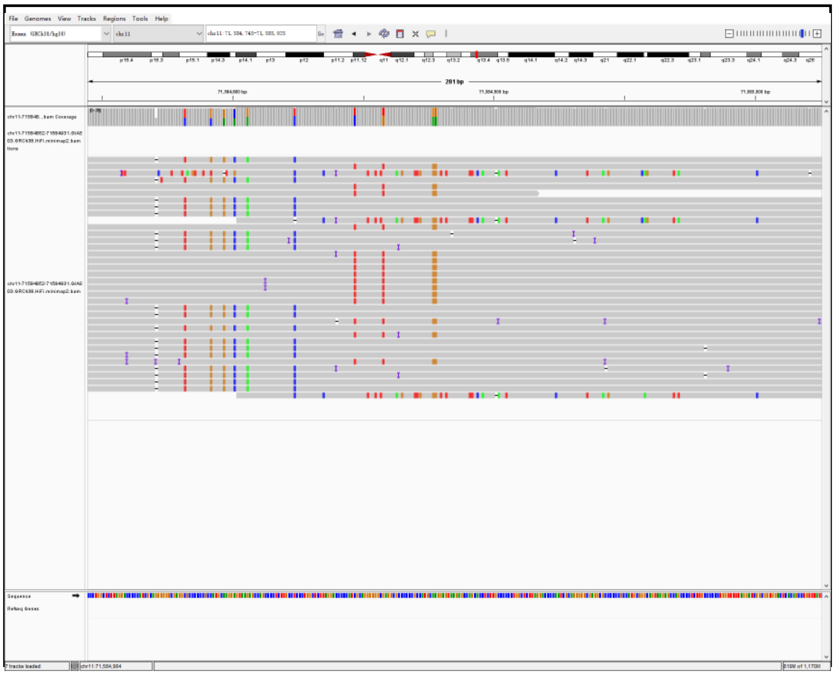

HG004

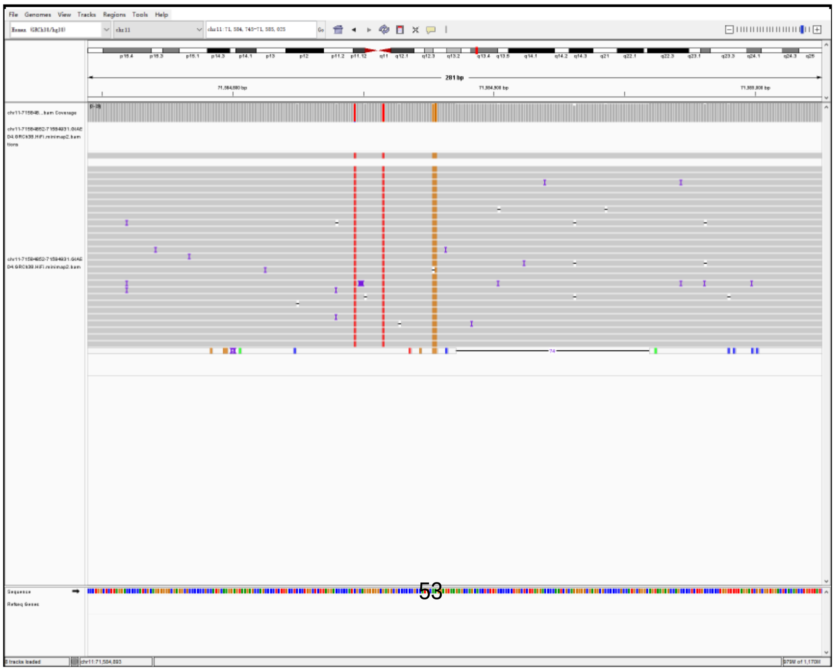

chr14 23280710 23280710 543 INS hg002-3-4 svision_pro

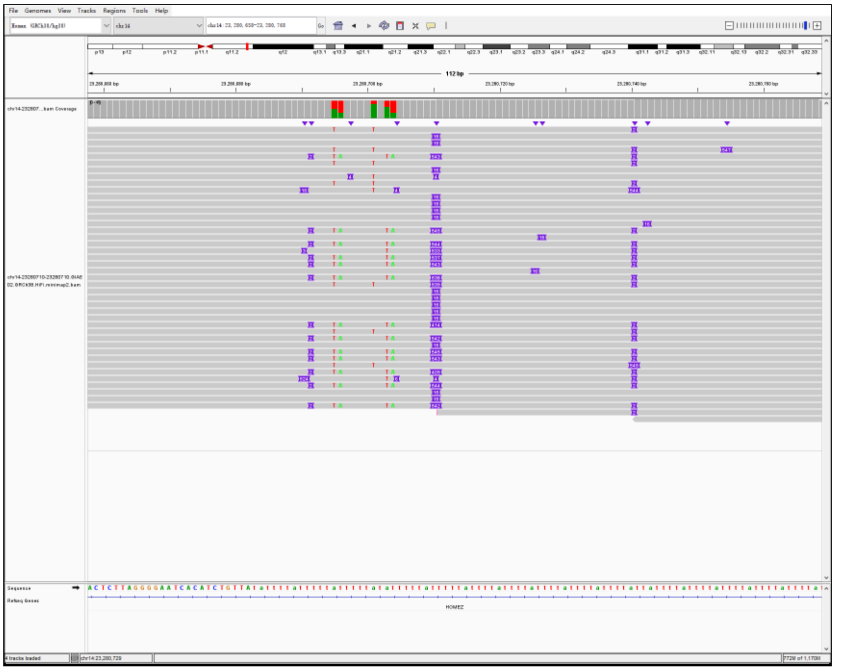

HG002

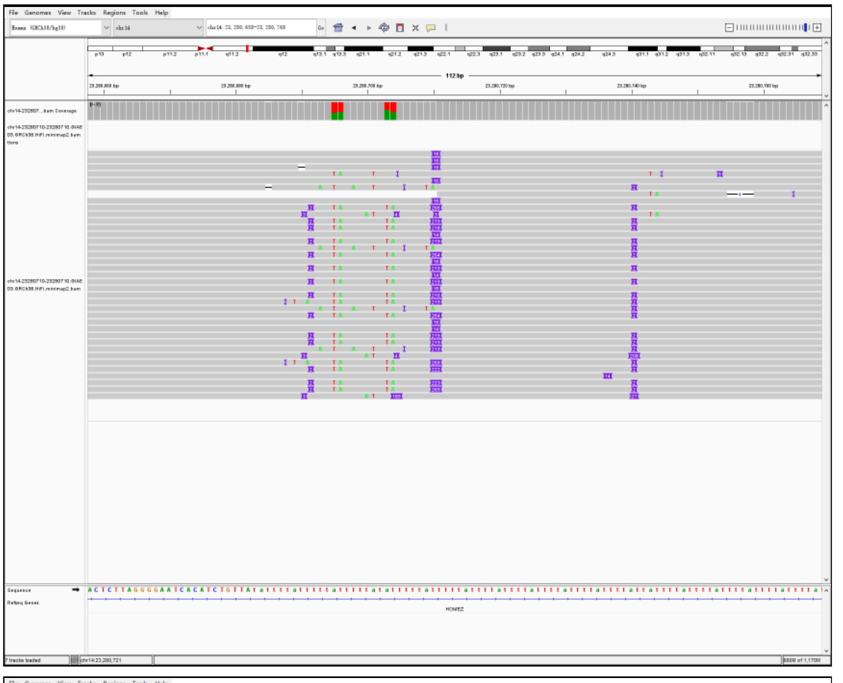

HG003

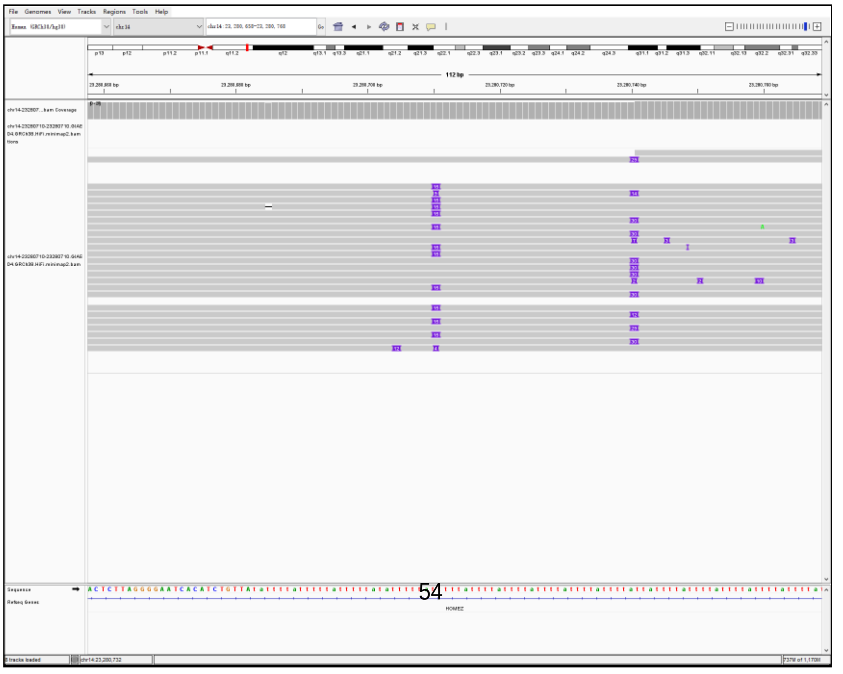

HG004

chr17 53340465 53340572 107 DEL hg002-3-4 svision_pro

HG002

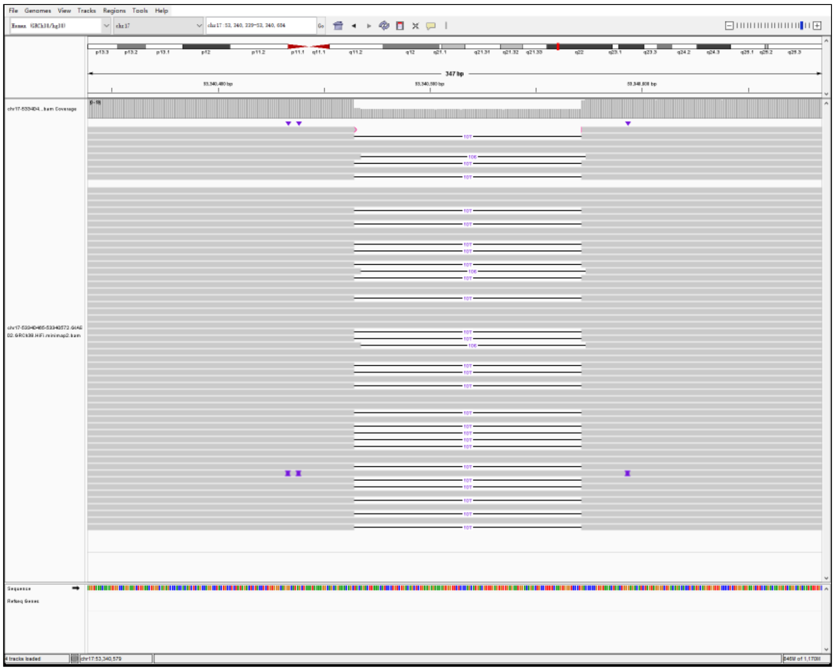

HG003

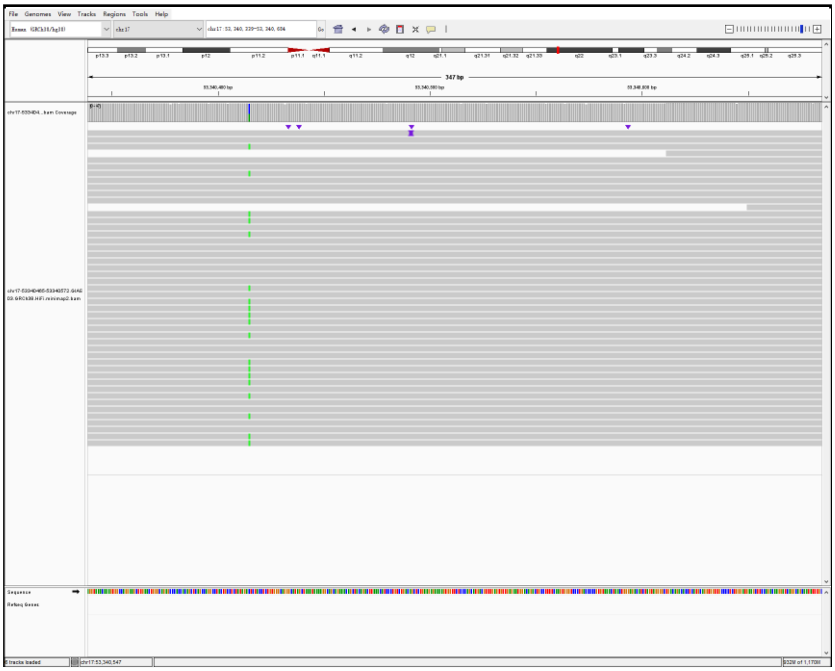

HG004

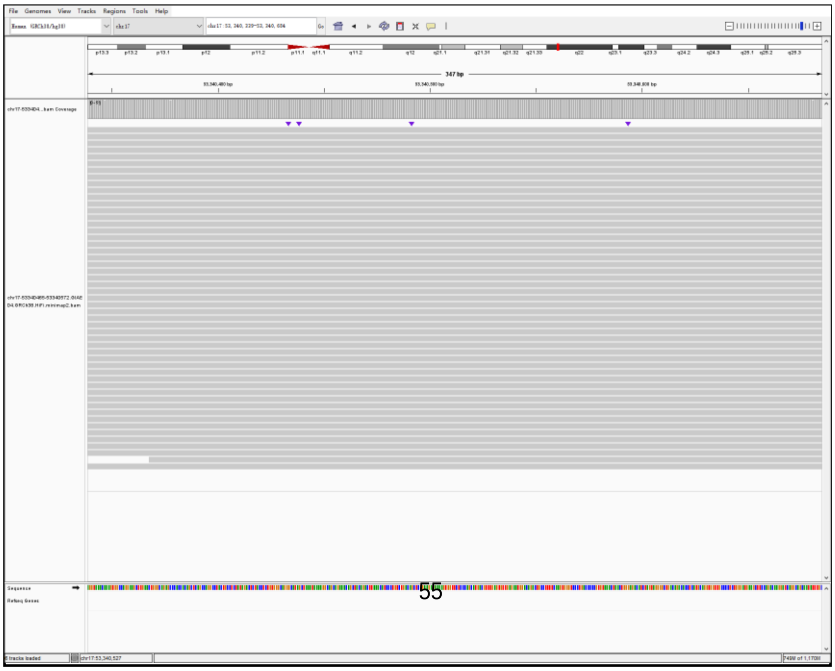

chr3 52028006 52028007 55 INS hg005-6-7 svision_pro

HG002

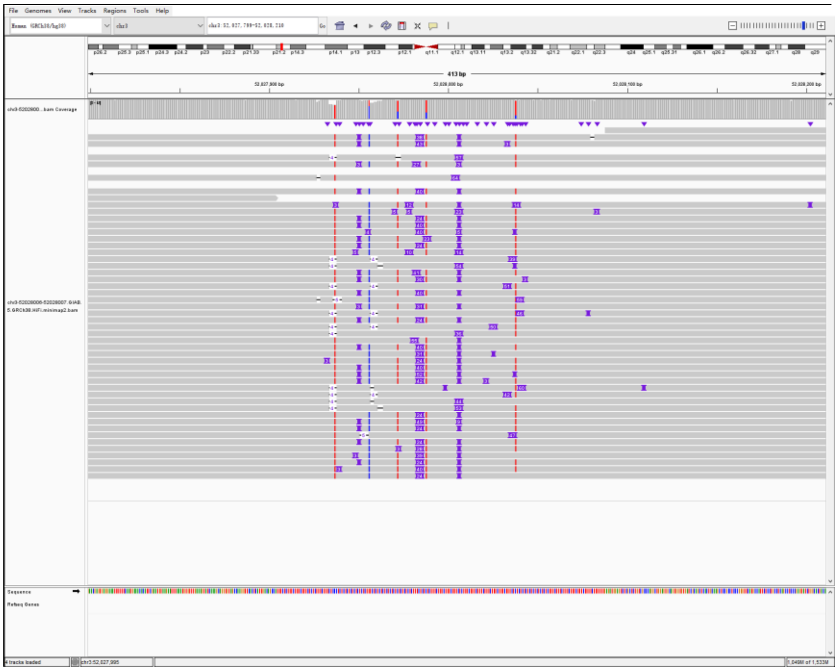

HG003

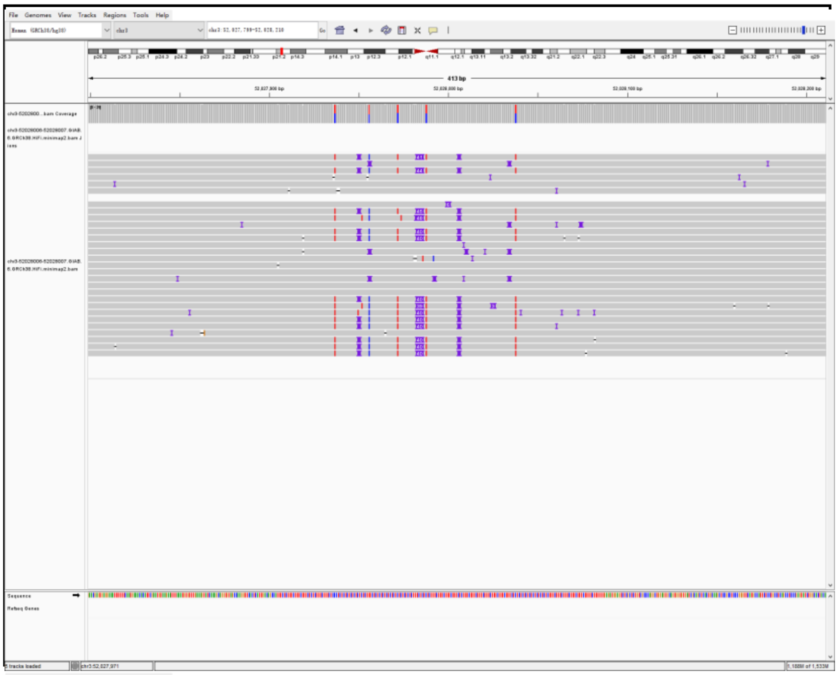

HG004

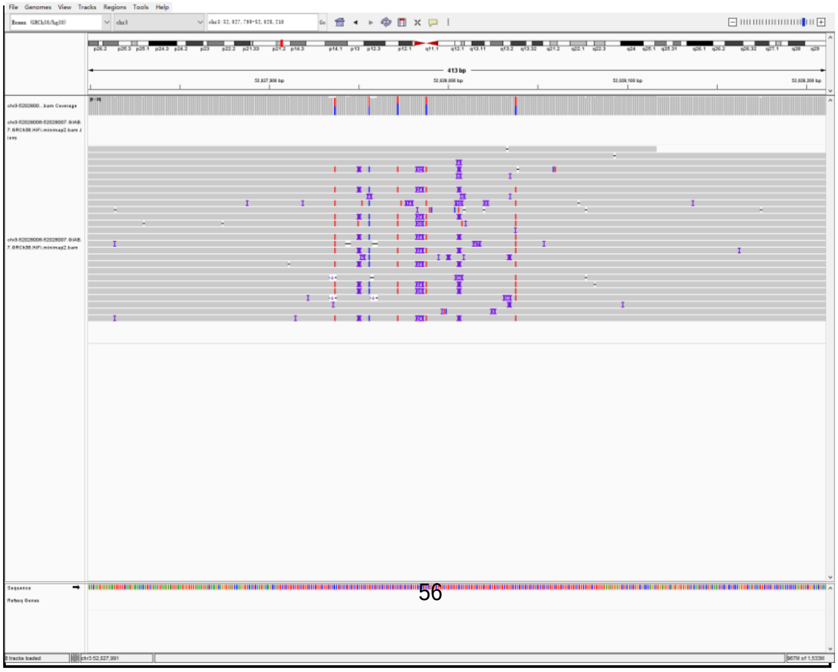

chr5 101696 101803 106 DEL hg005-6-7 svision_pro

HG002

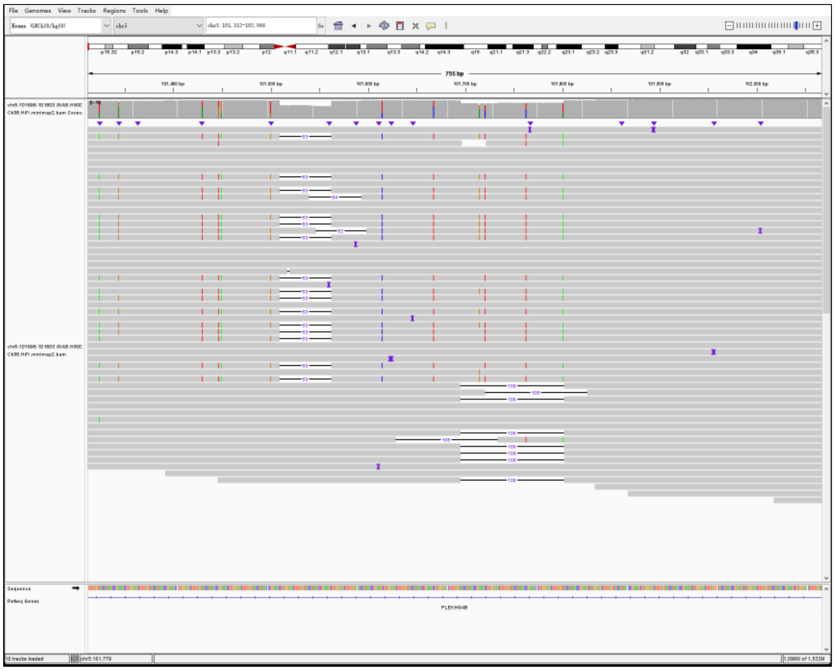

HG003

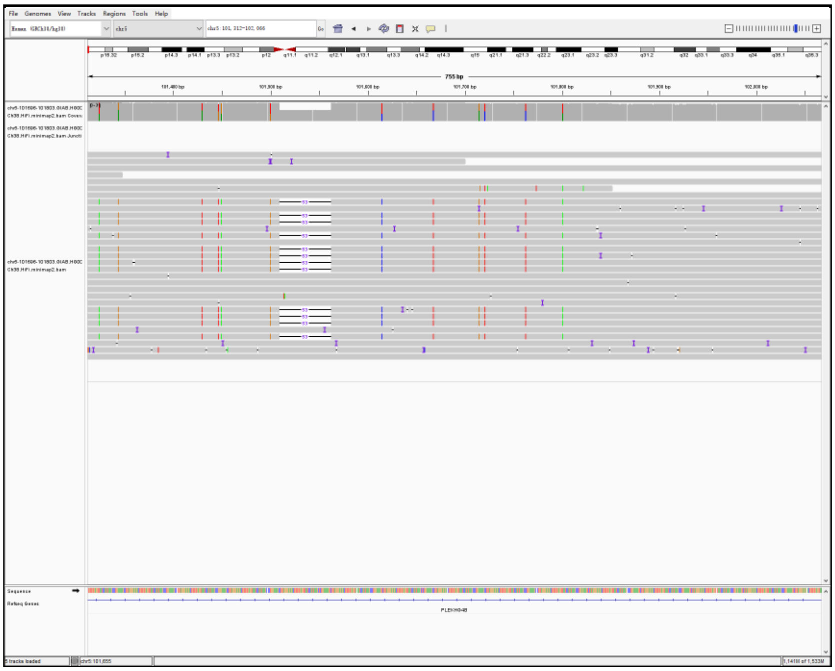

PAV call：
NA

HG004

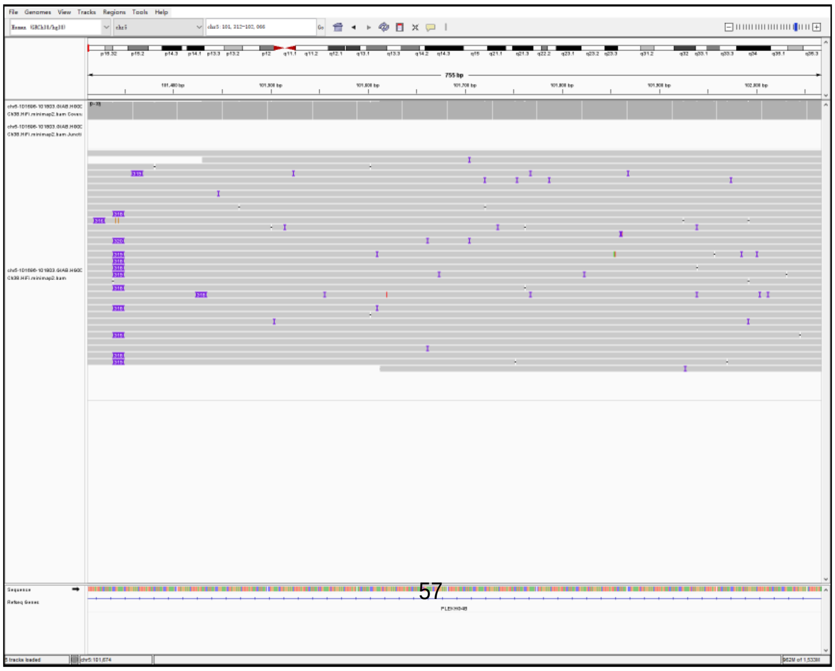

PAV call：
NA

chr11 134530200 134530281 81 DEL hg005-6-7 svision_pro

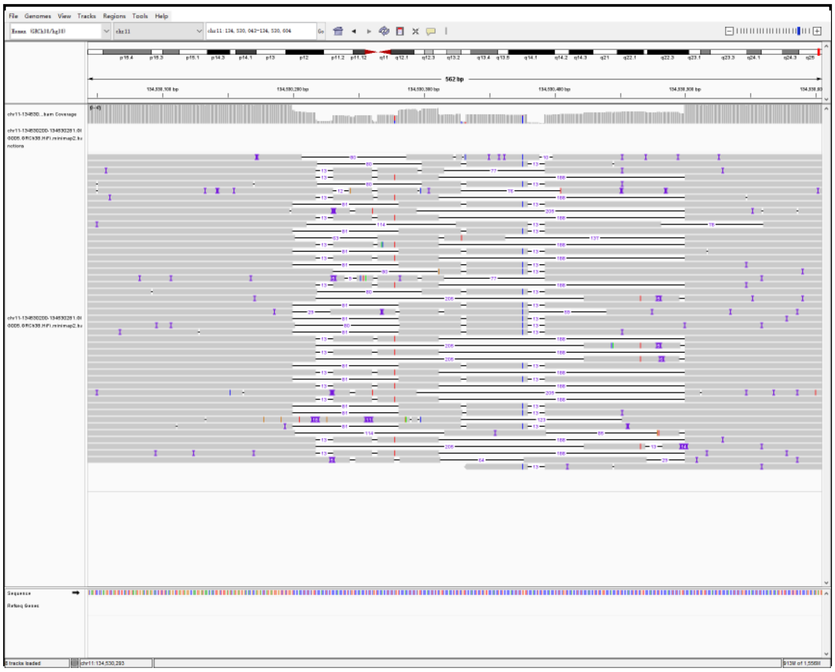

HG005

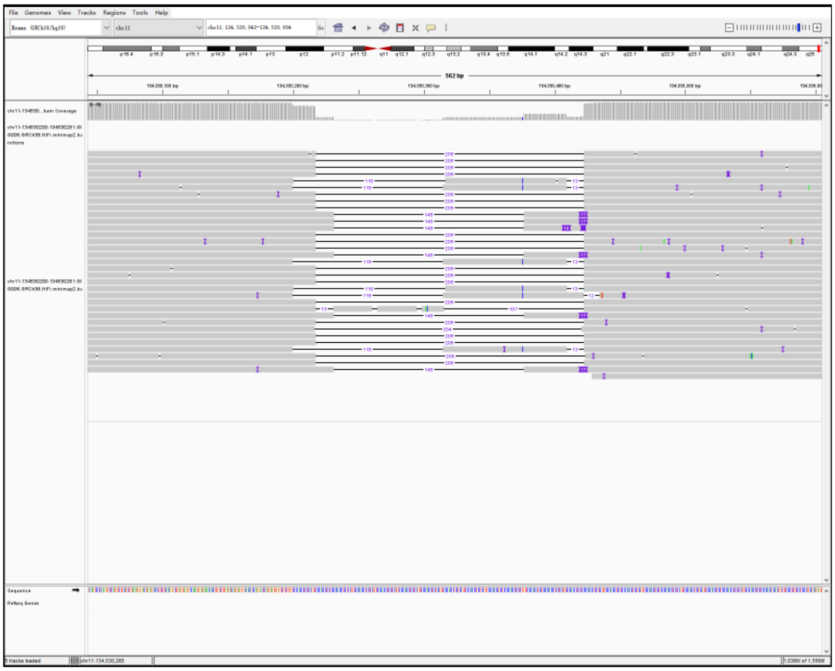

HG006

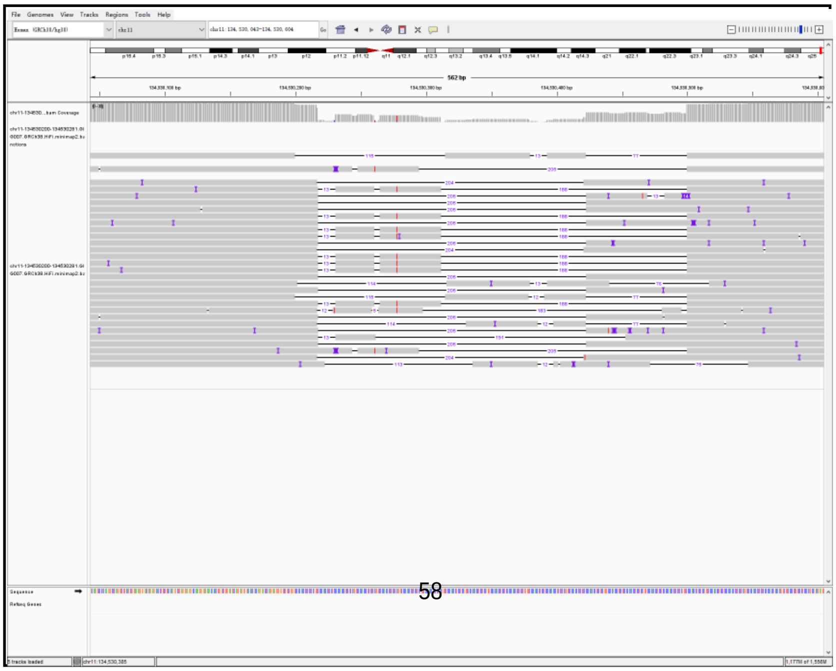

HG007

chr17	43225693	43225693	50	INS	hg005-6-7	svision_pro

HG005

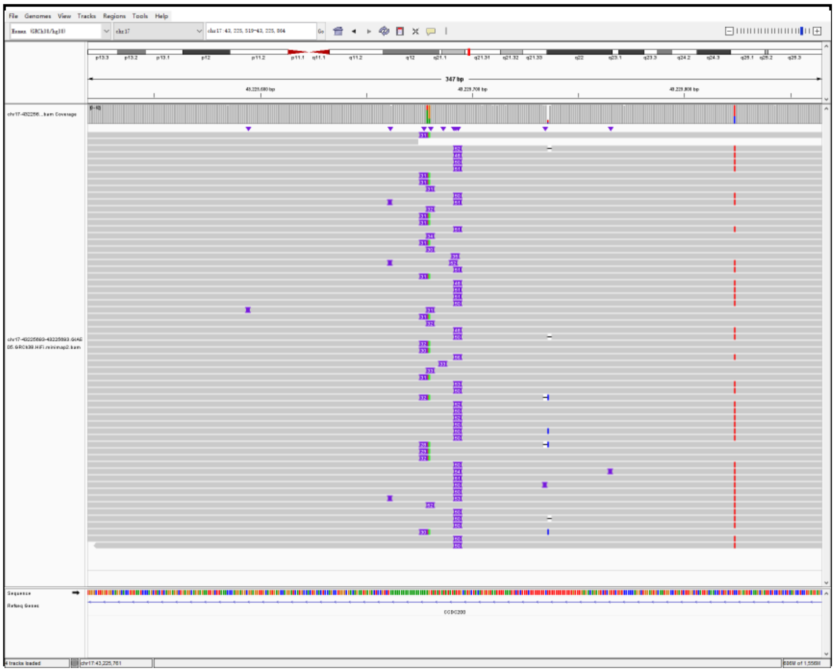

HG006

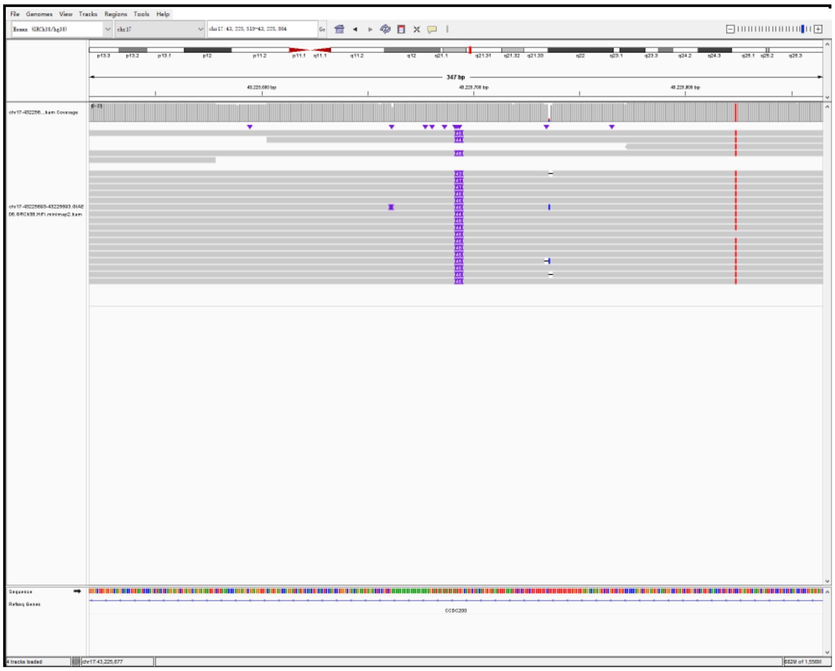

HG007

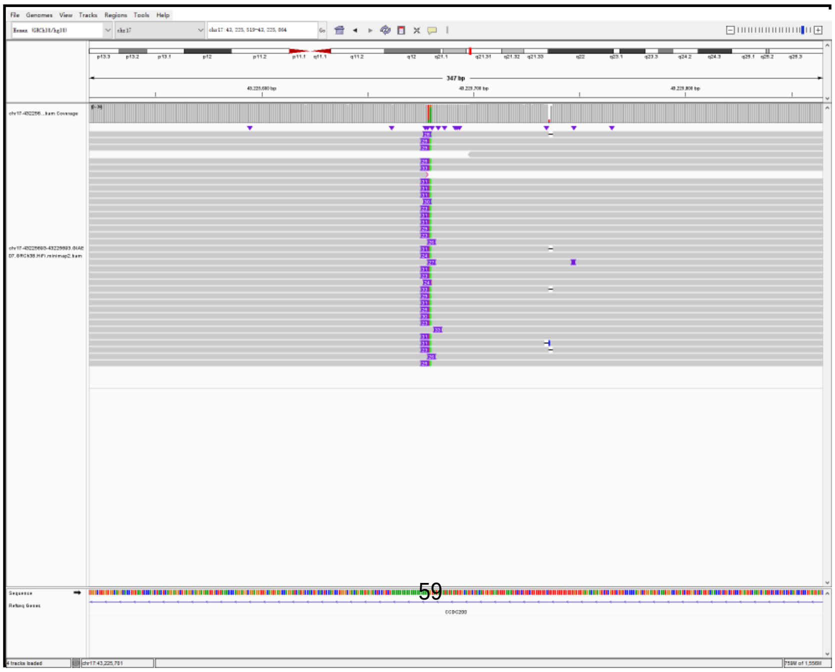

chr17 48538439 48539859 1420 DEL hg005-6-7 svision_pro

HG005

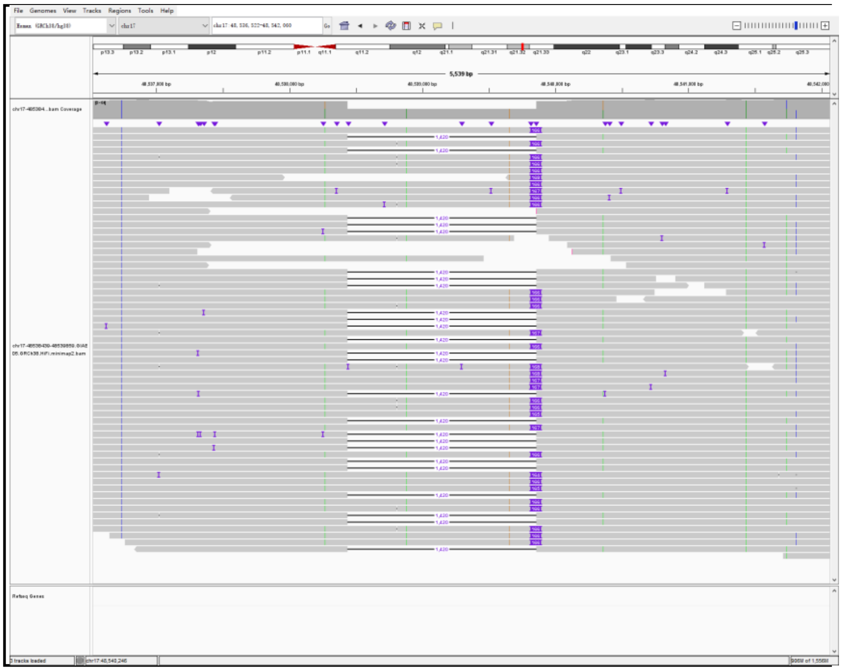

HG006

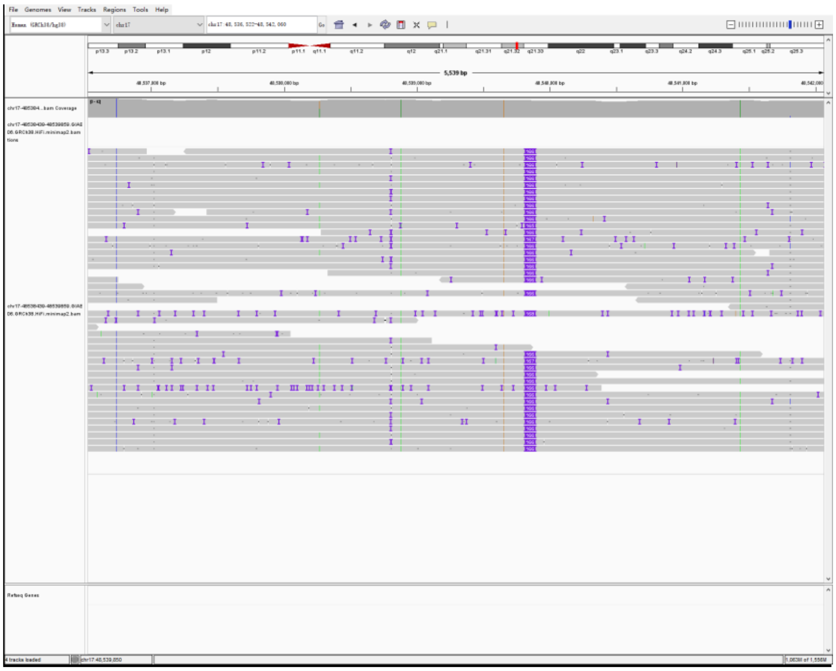

HG007

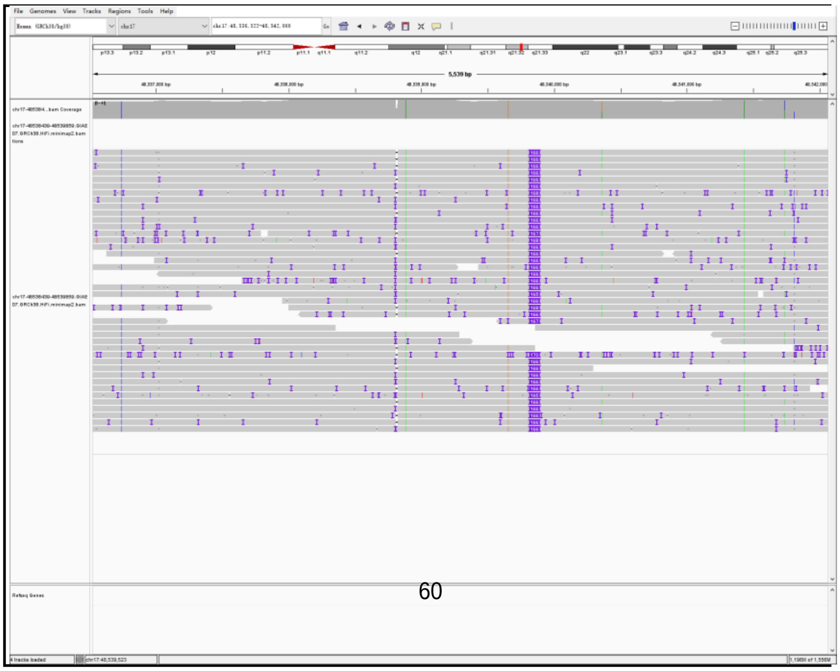

chr17 82586256 82586257 10472 INS hg005-6-7

HG005

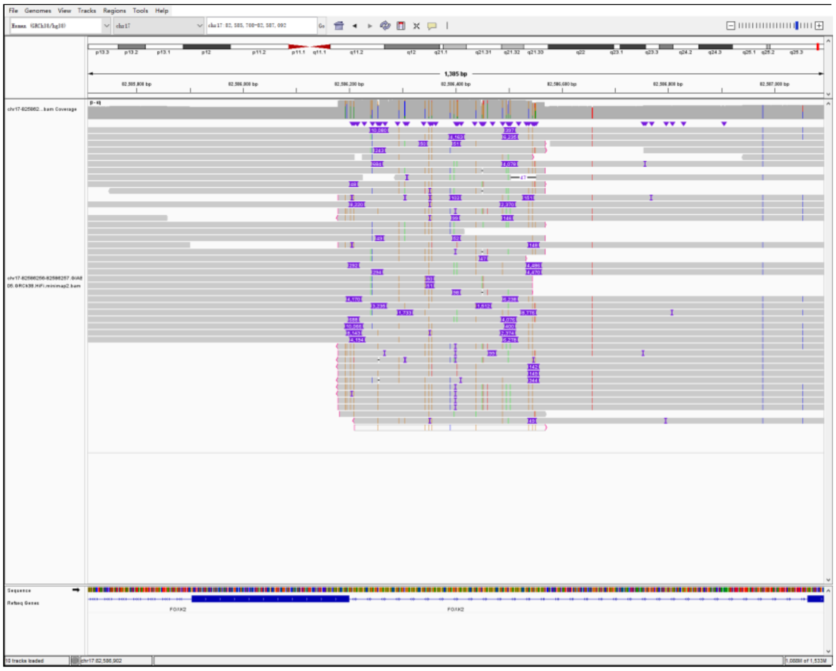

HG006

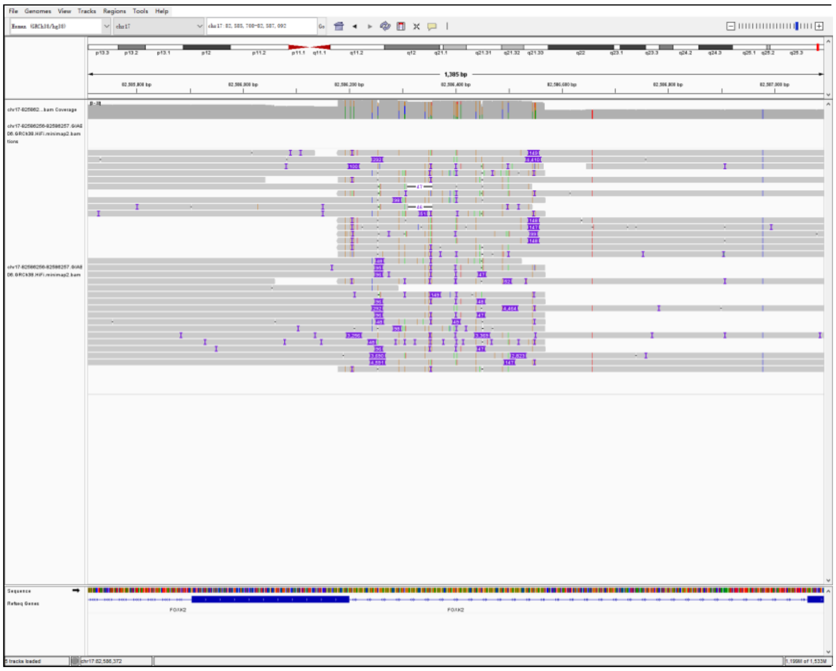

HG007

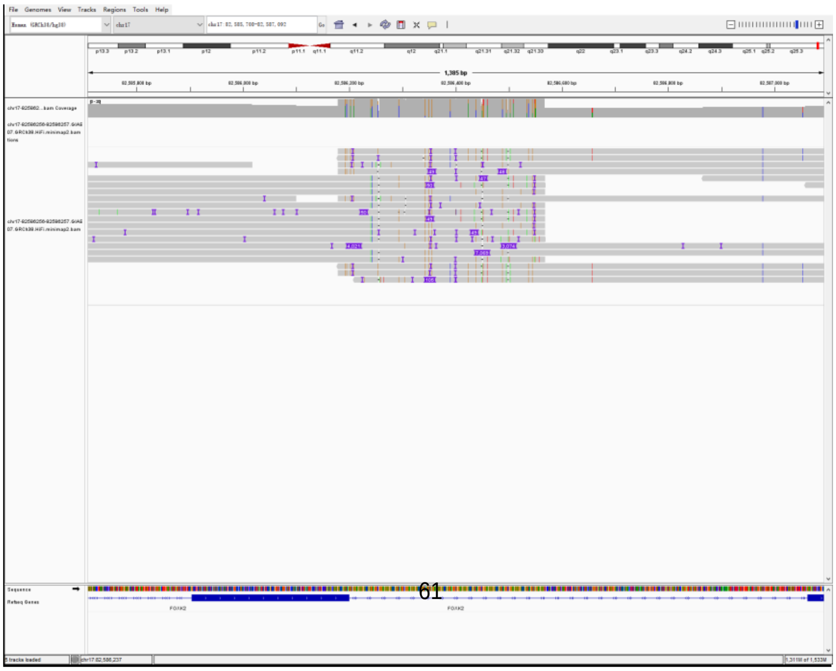

chrX 1589086 1589141 55 DEL hg005-6-7 svision_pro

HG005

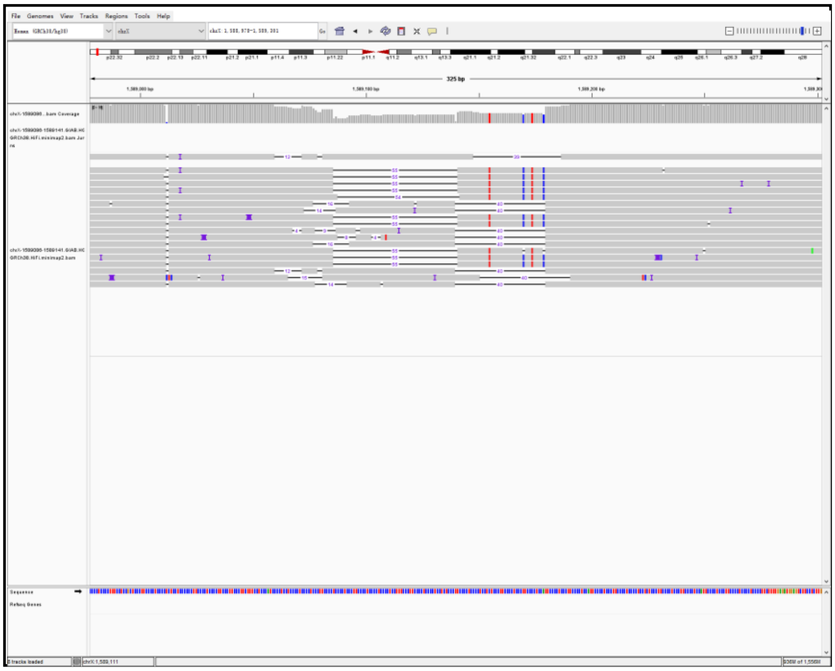

HG006

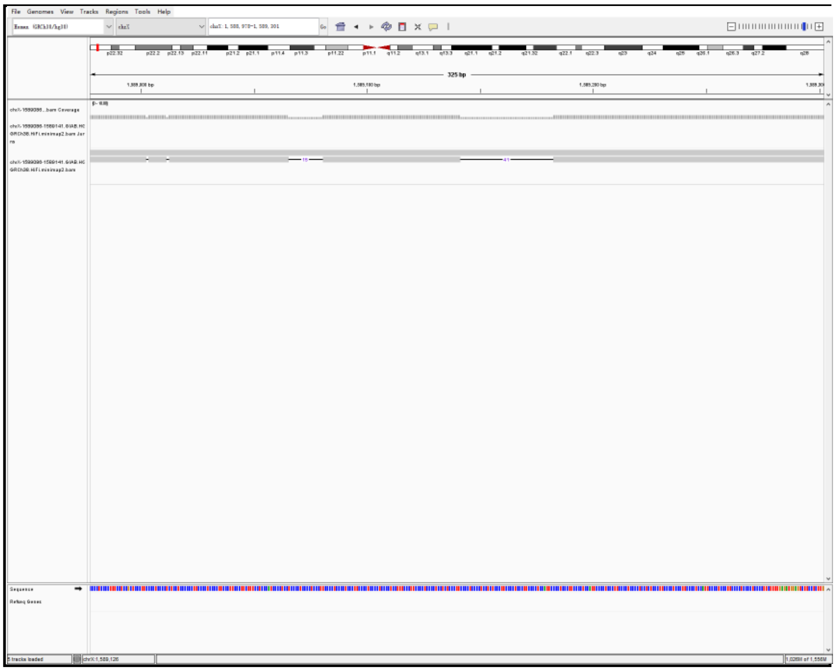

HG007

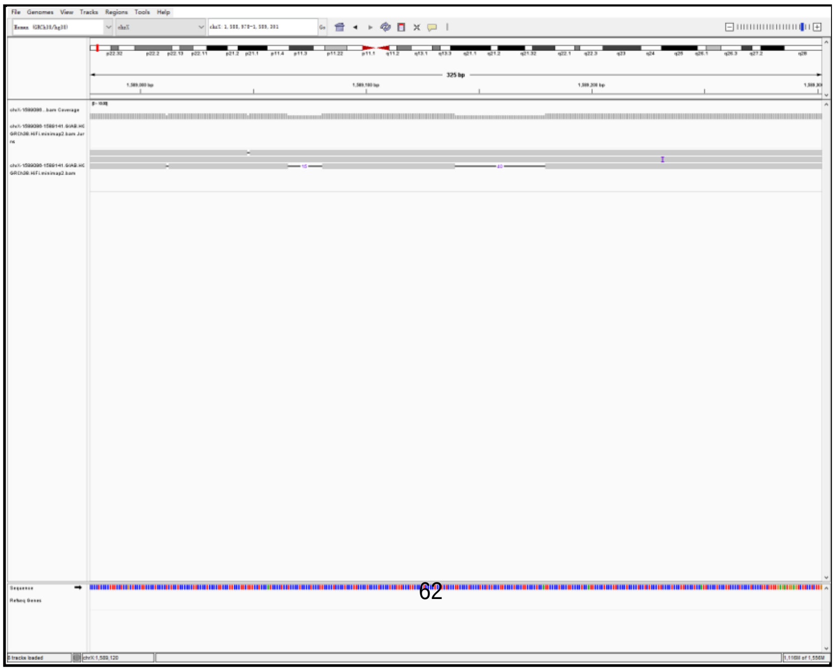

chr1 200816966 200816966 72 INS hg00514-2-3 svision_pro

HG00514

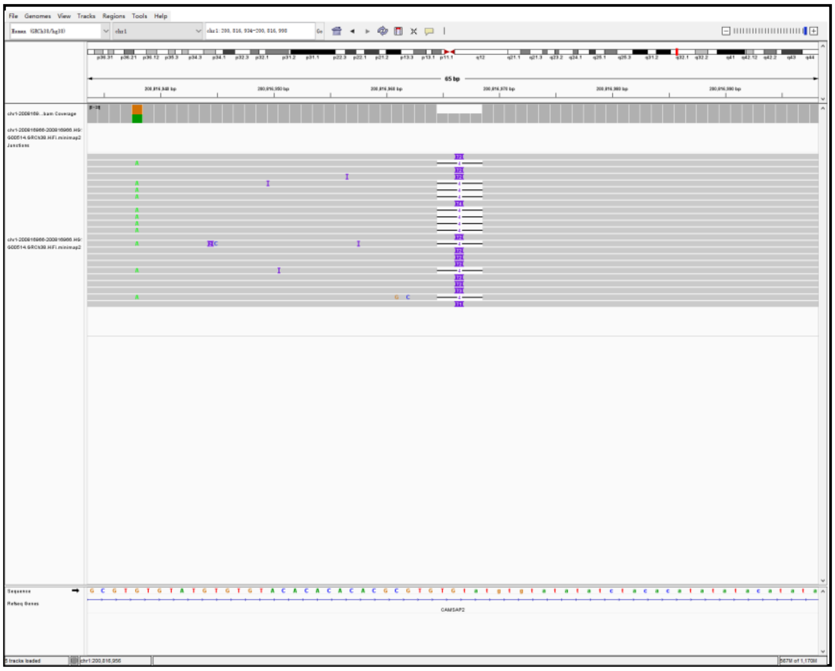

HG00512

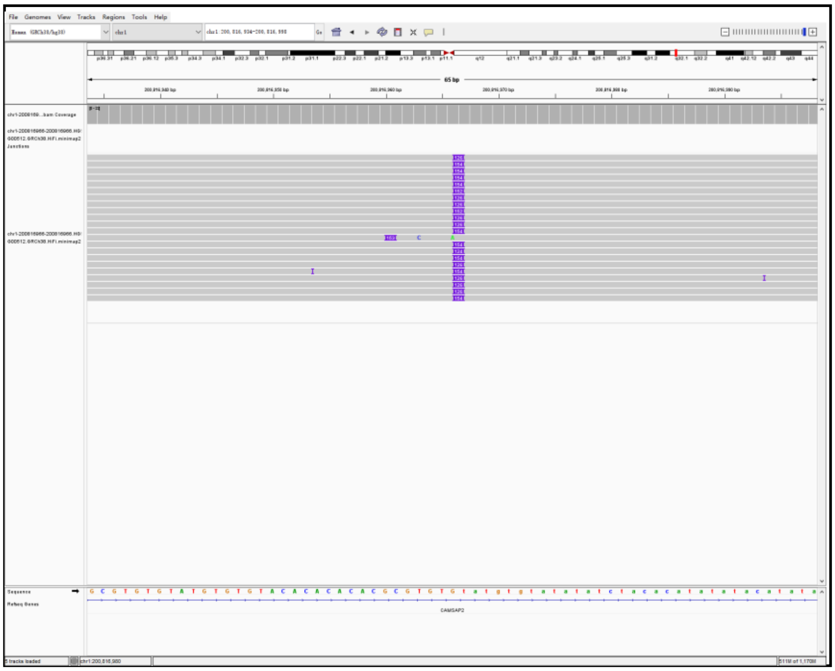

HG00513

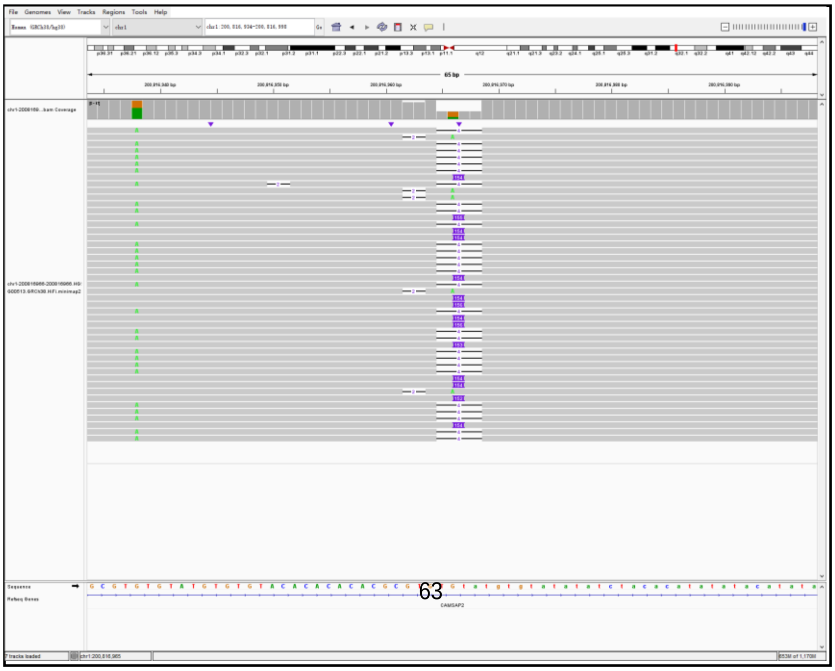

chr6	81760484	81760484	61	INS	hg00514-2-3	svision_pro

HG00514

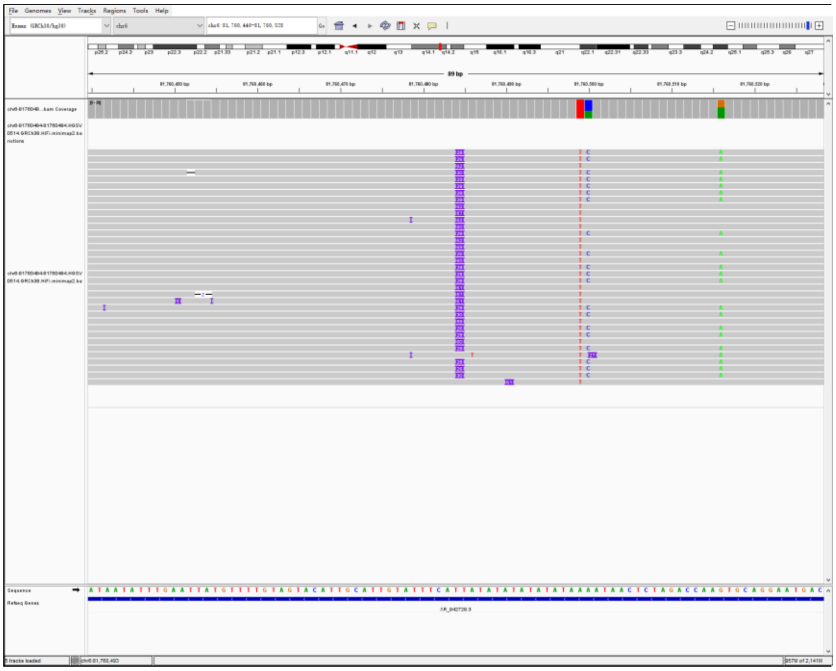

HG00512

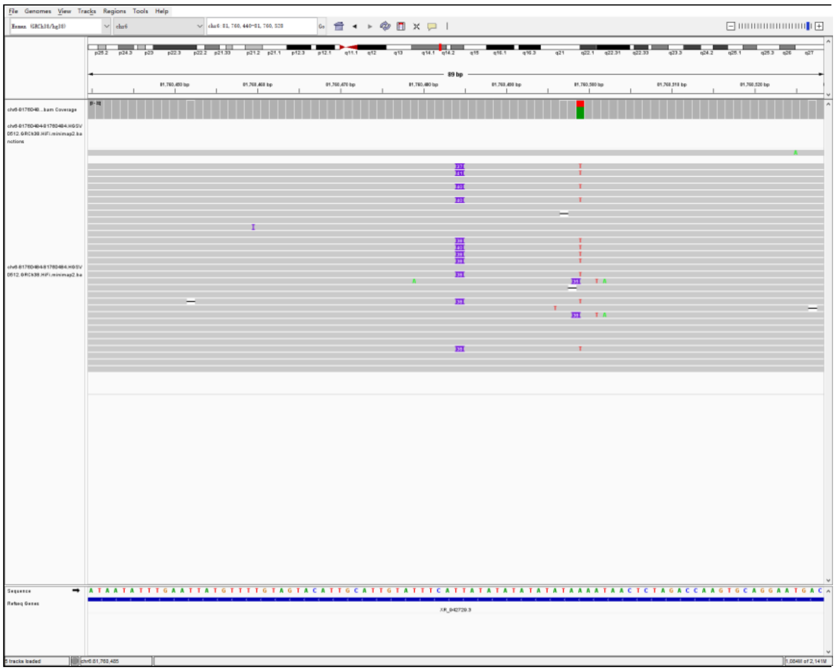

HG00513

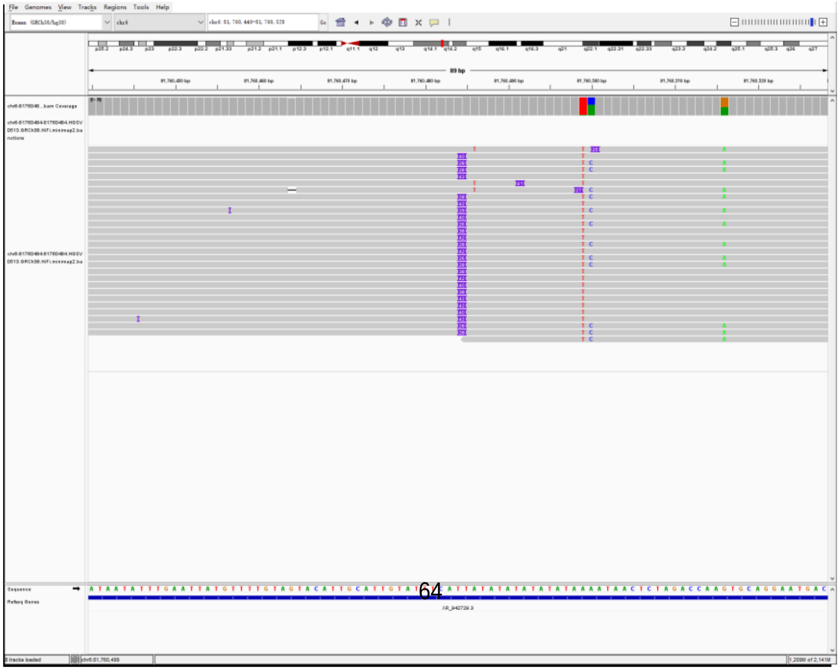

chr18 55156799 55156799 73 INS hg00514-2-3 svision_pro

HG00514

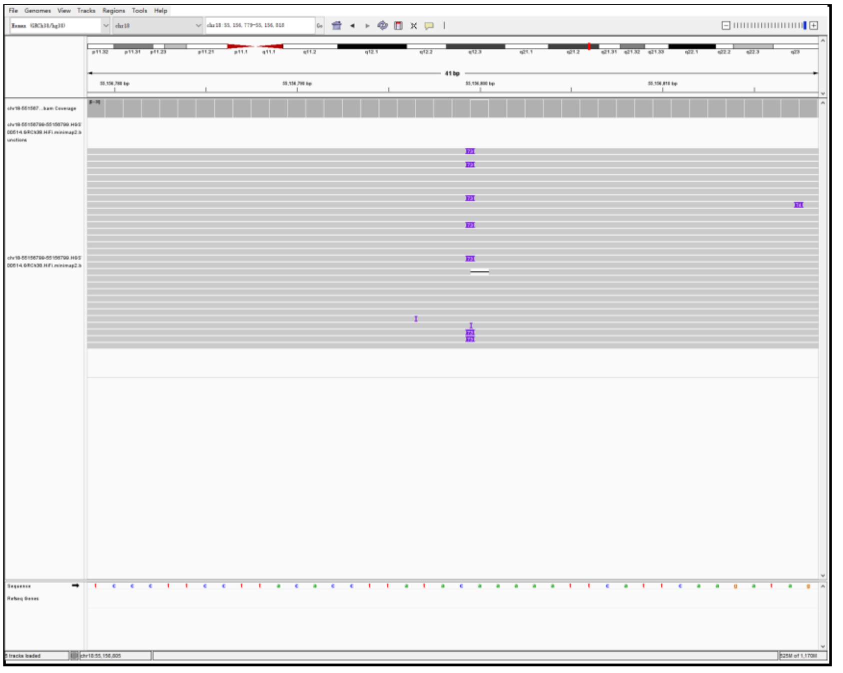

HG00512

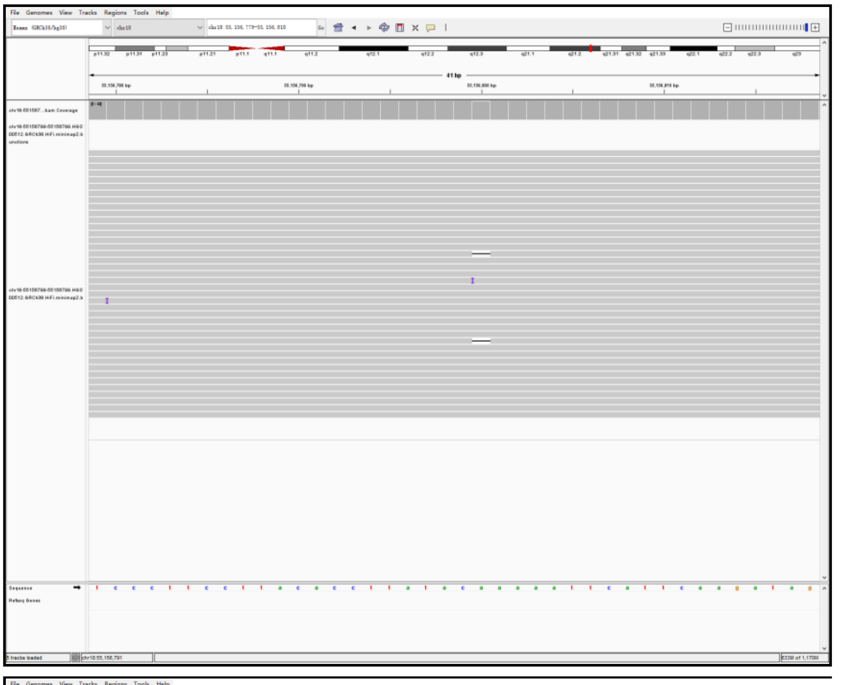

HG00513

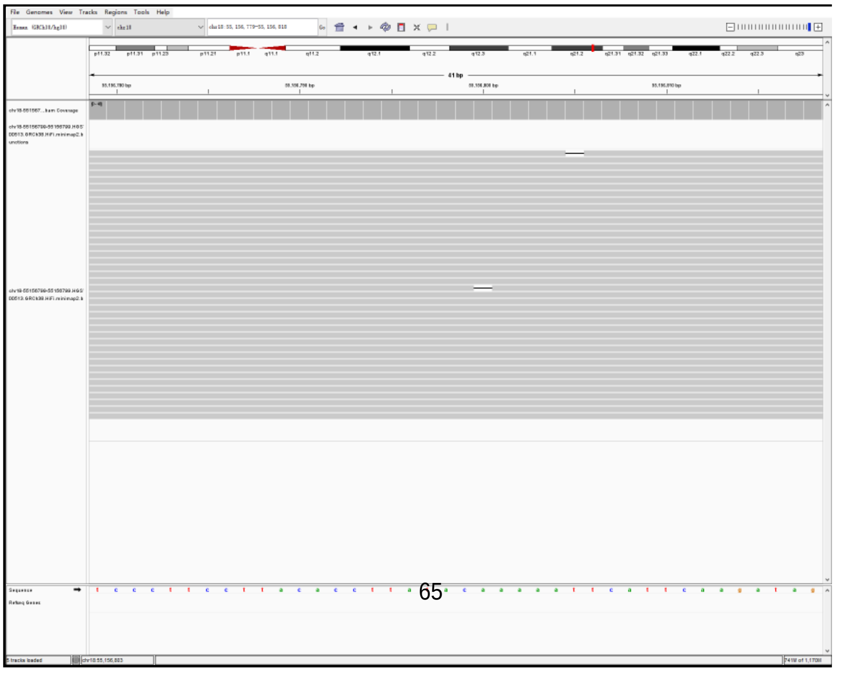

chr19 49414675 49414733 58 DEL hg00514-2-3 svision_pro

HG00514

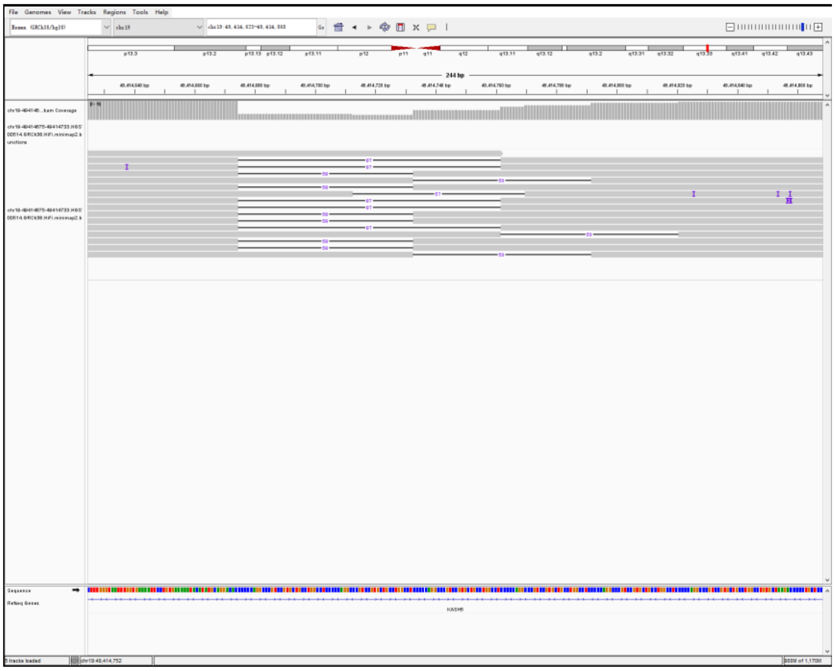

HG00512

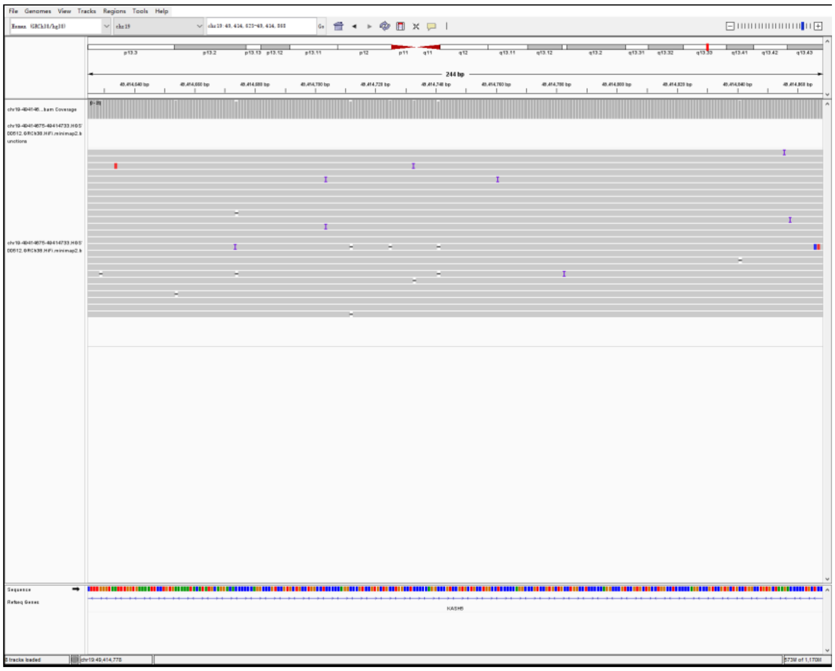

HG00513

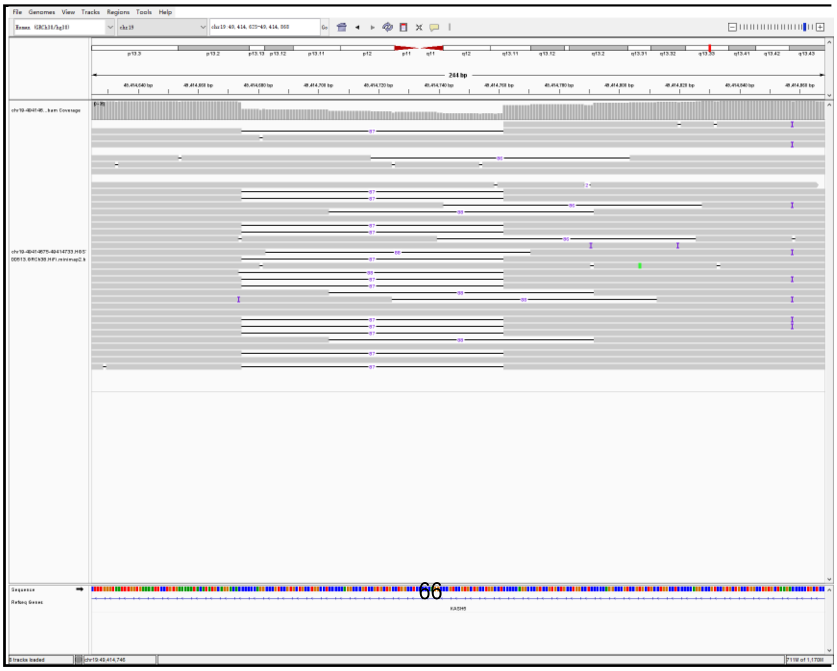

chr20 1149893 1149893 155 INS hg00514-2-3 svision_pro

HG00514

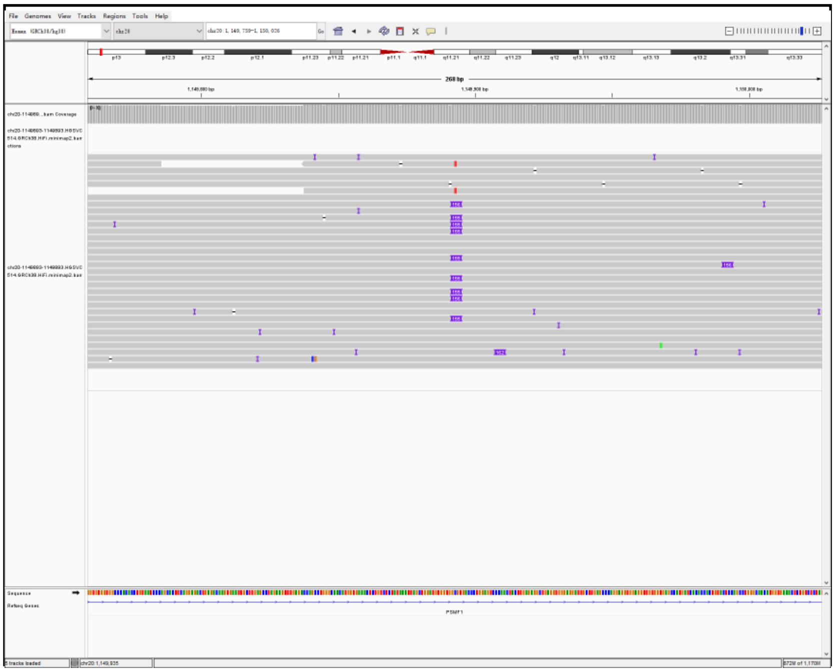

HG00512

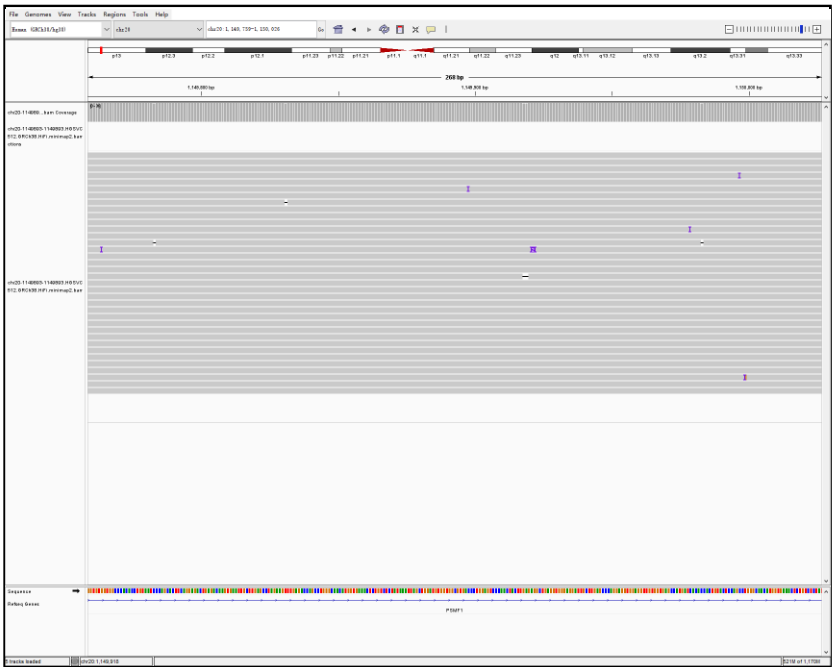

HG00513

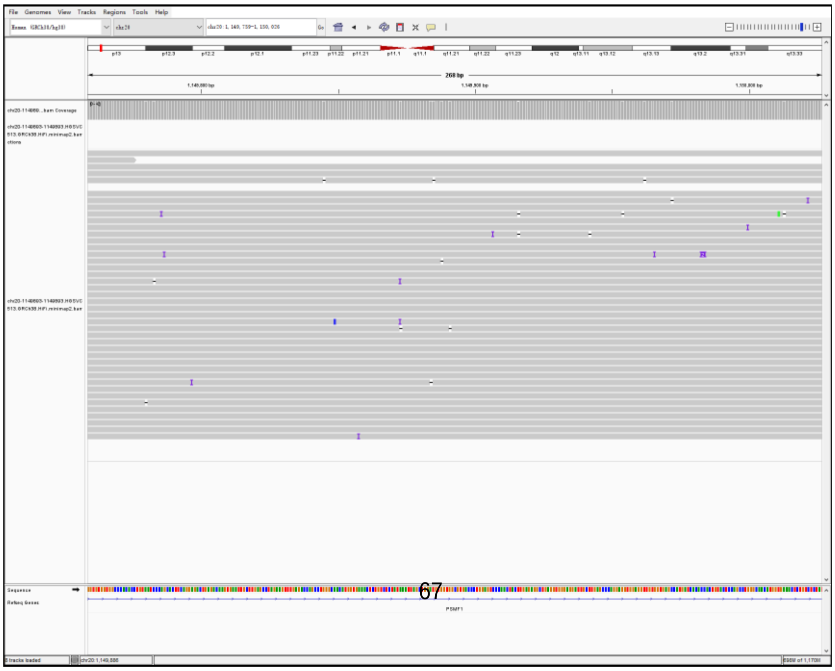

chrX	155575615	155575977	362	DEL	hg00514-2-3	svision_pro

HG00514

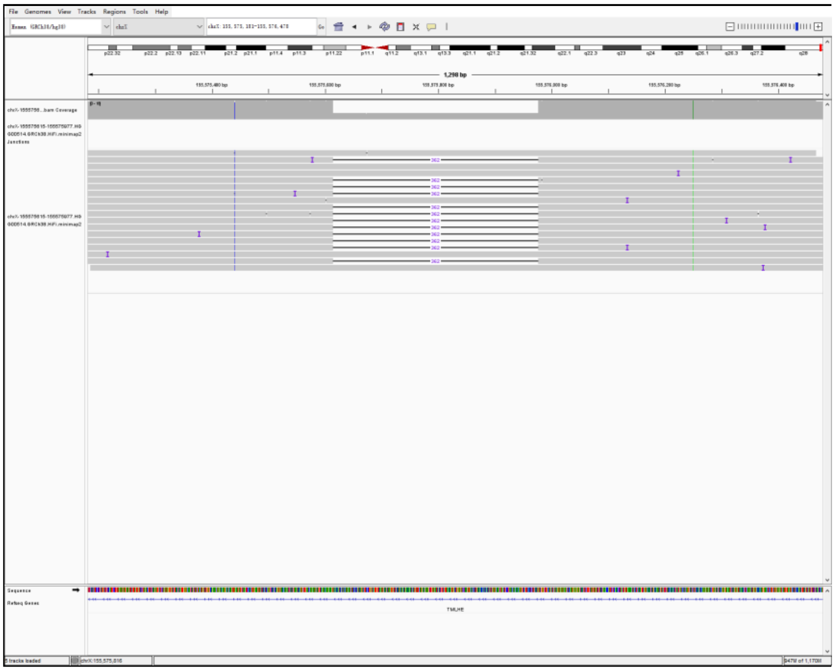

HG00512

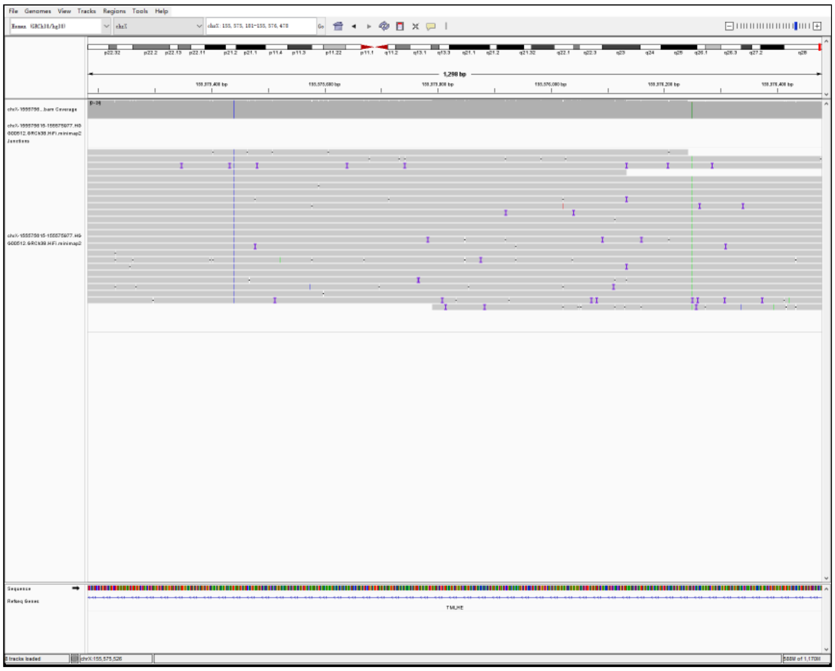

HG00513

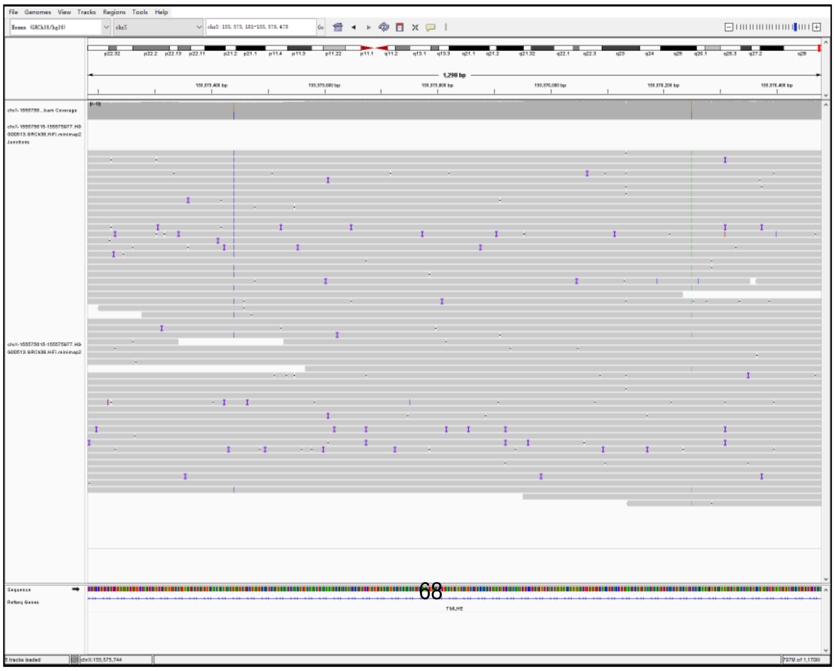

chr2	62406515	62406515	168	INS	hg00733-1-2	svision_pro

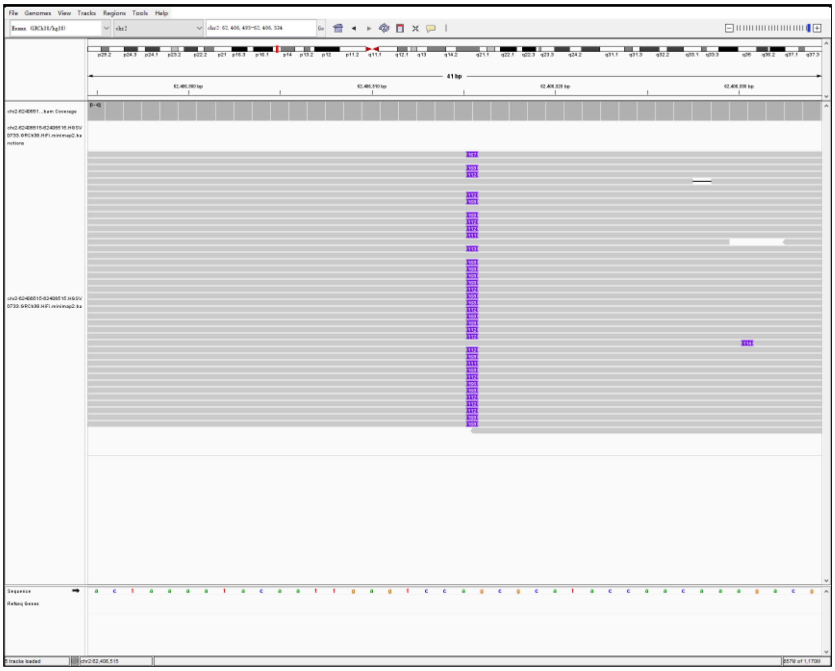

HG00733

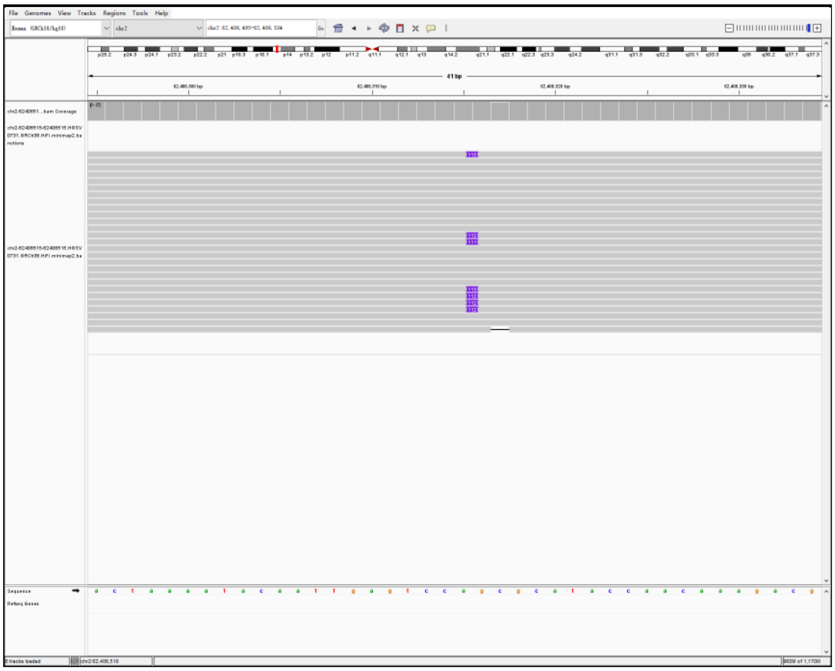

HG00731

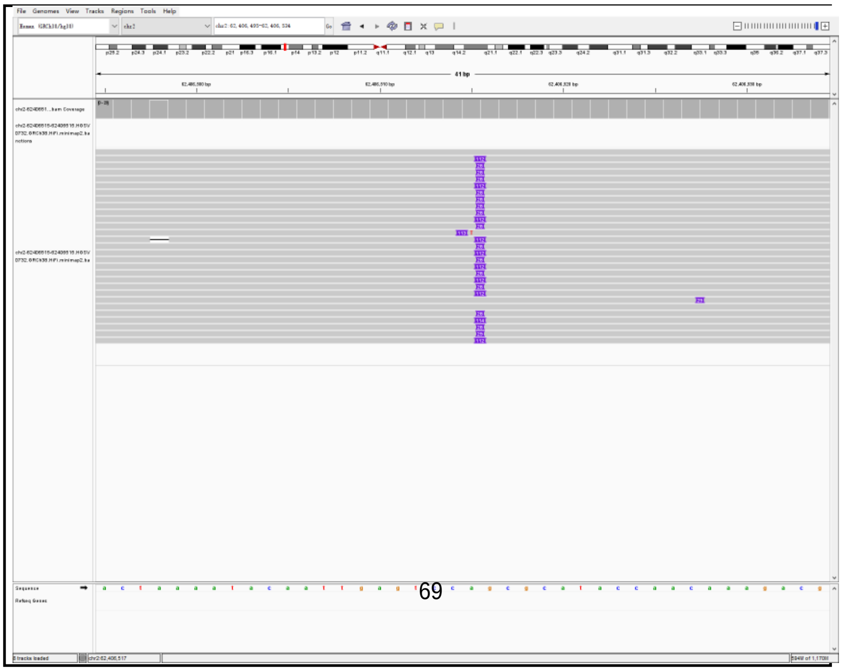

HG00732

chr6 166221900 166222002 102 DEL hg00733-1-2 svision_pro

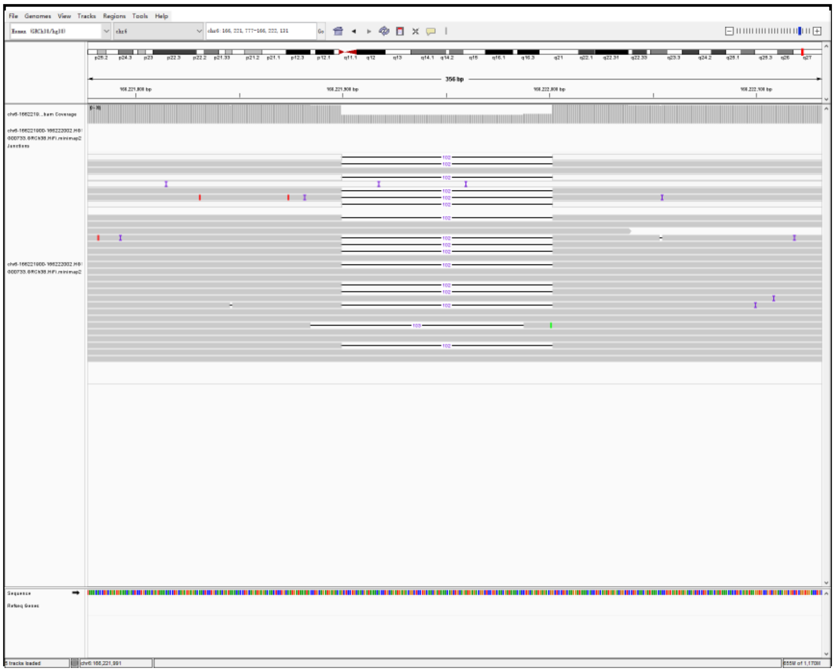

HG00733

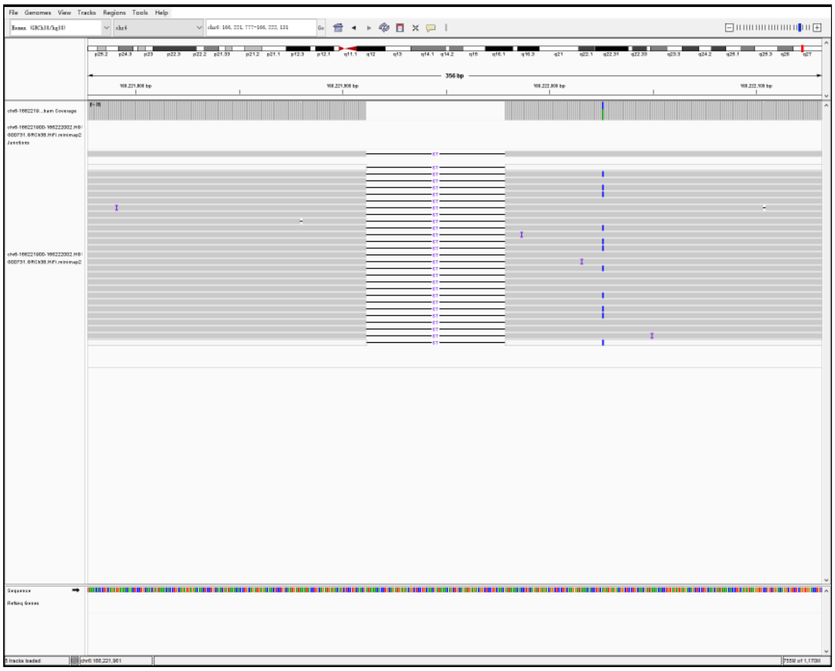

HG00731

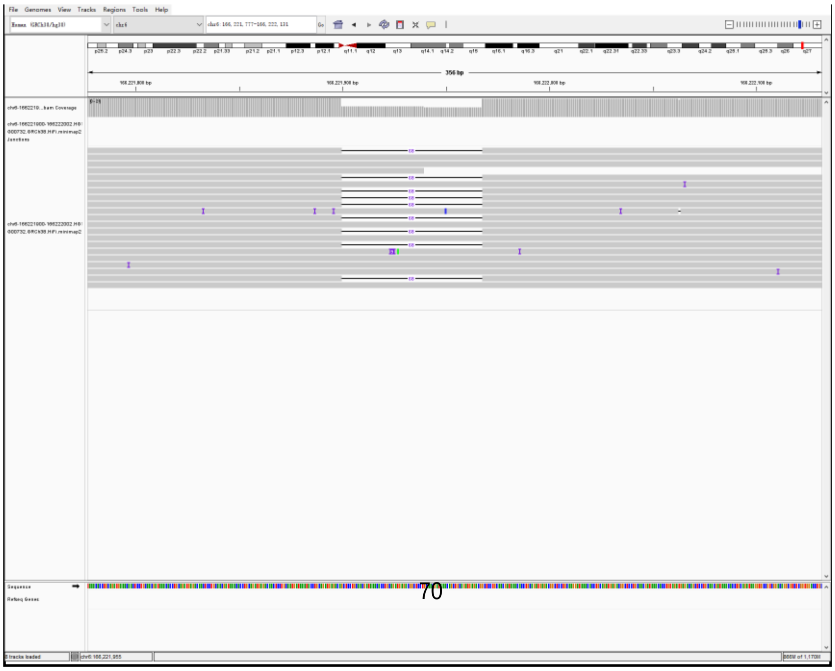

HG00732

chr7 76997297 76997385 87 DEL hg00733-1-2 svision_pro

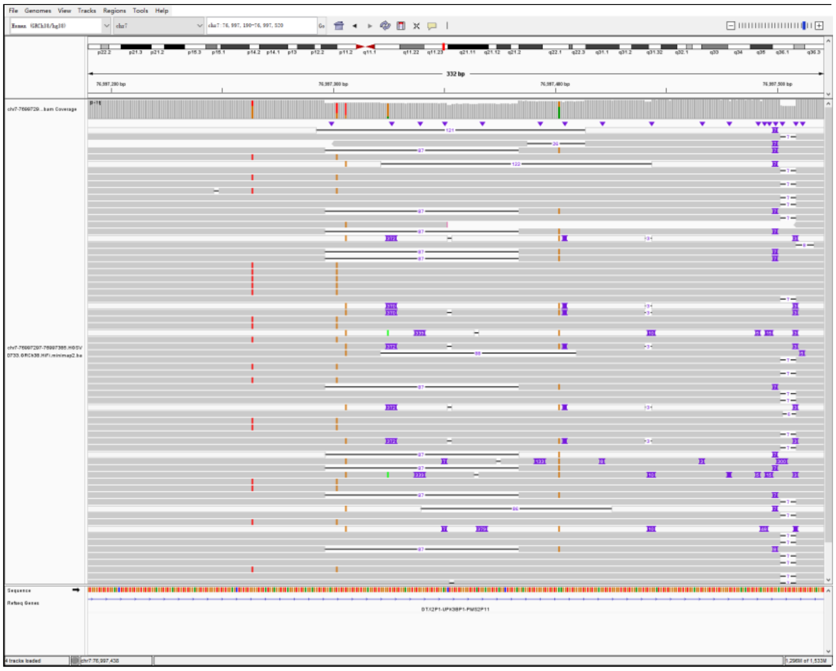

HG00733

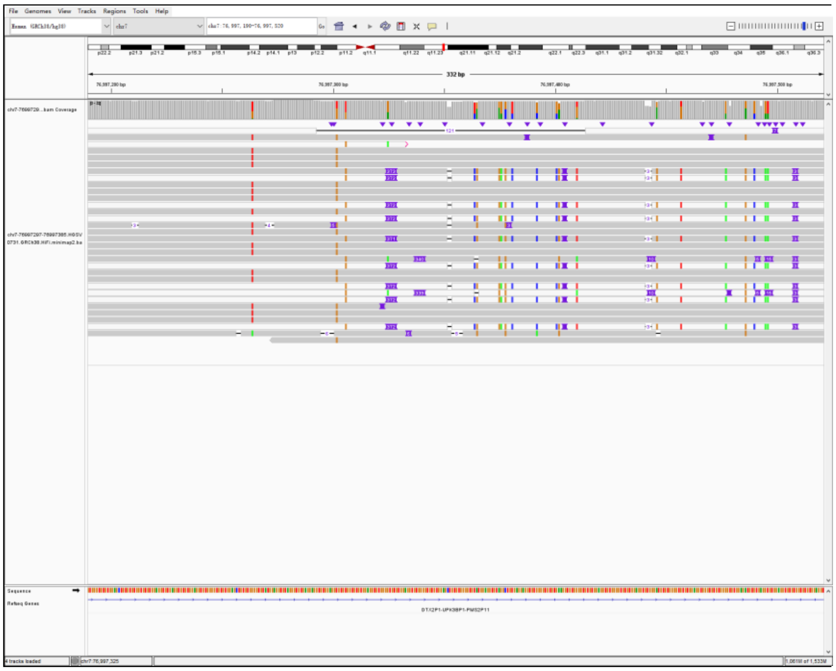

HG00731

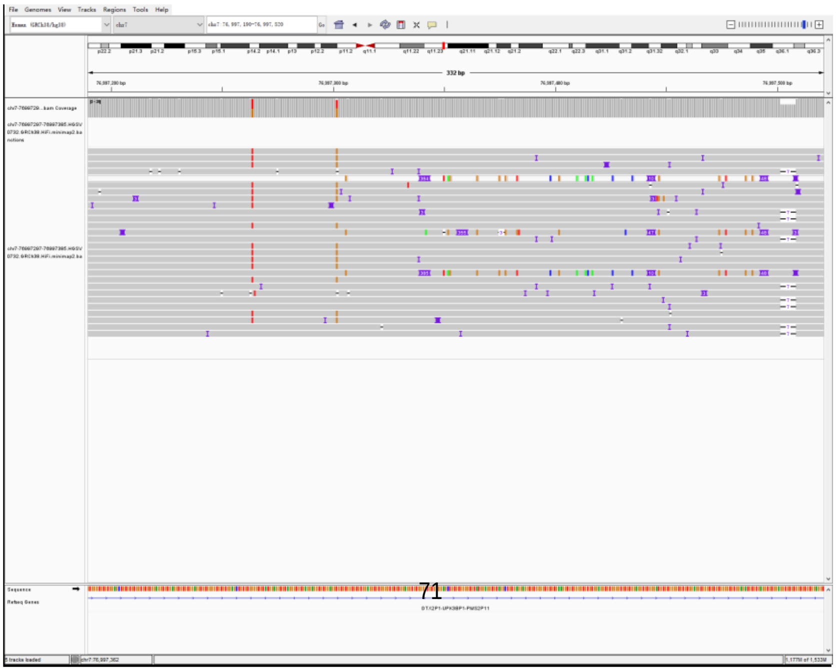

HG00732

chr1	190983080	190983080	52	INS	LCL5-6-7-8	svision_pro

LCL5

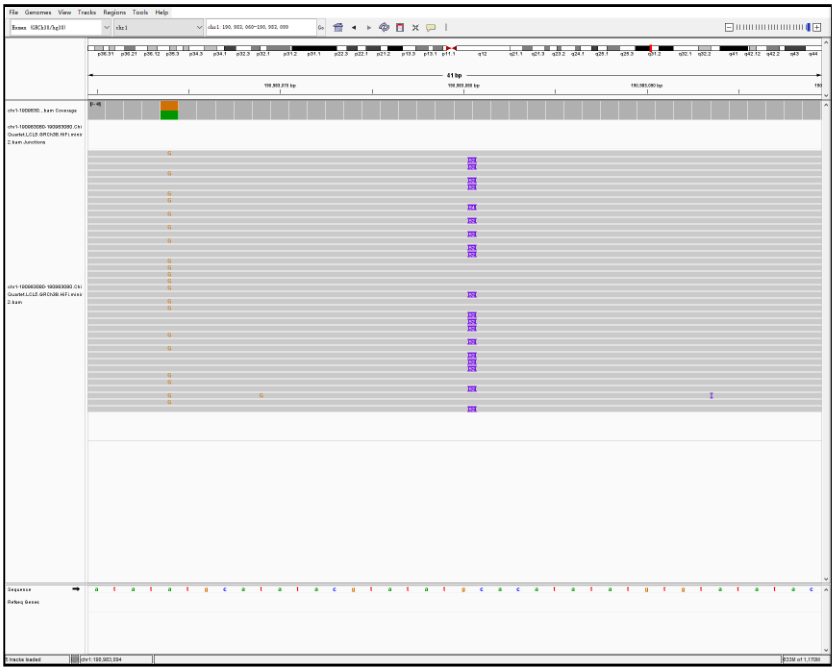

LCL7

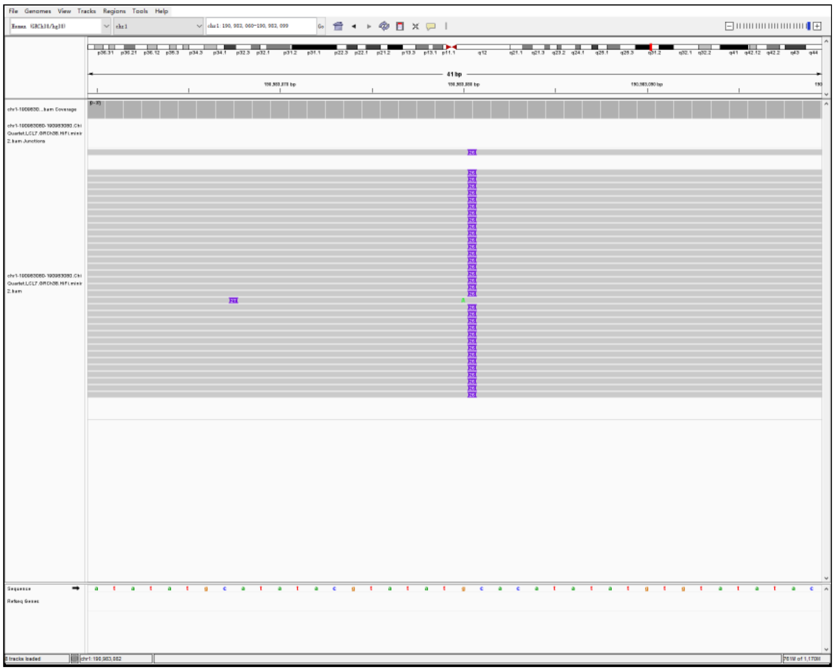

LCL8

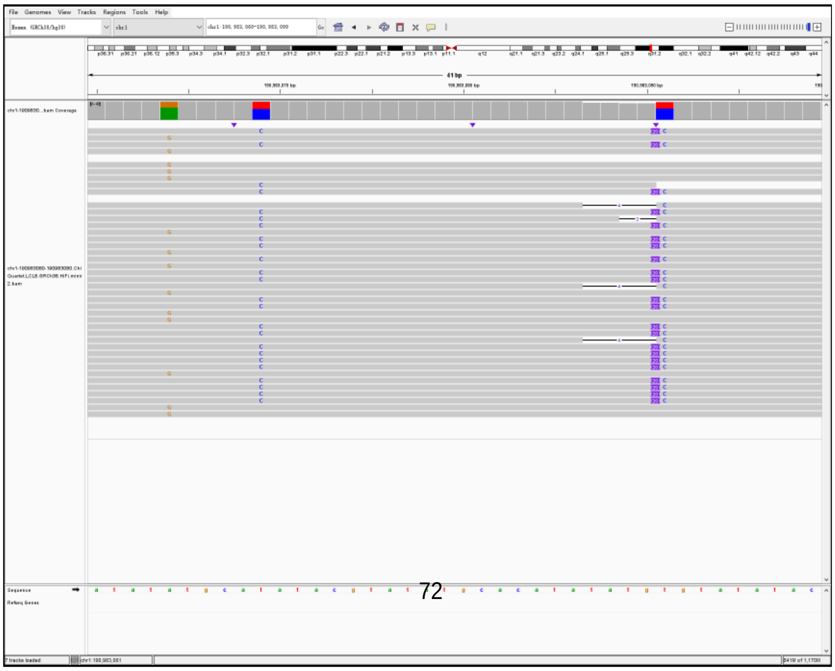

chr7 2492244 2492337 92 DEL lcl5-6-7-8 svision_pro

LCL5

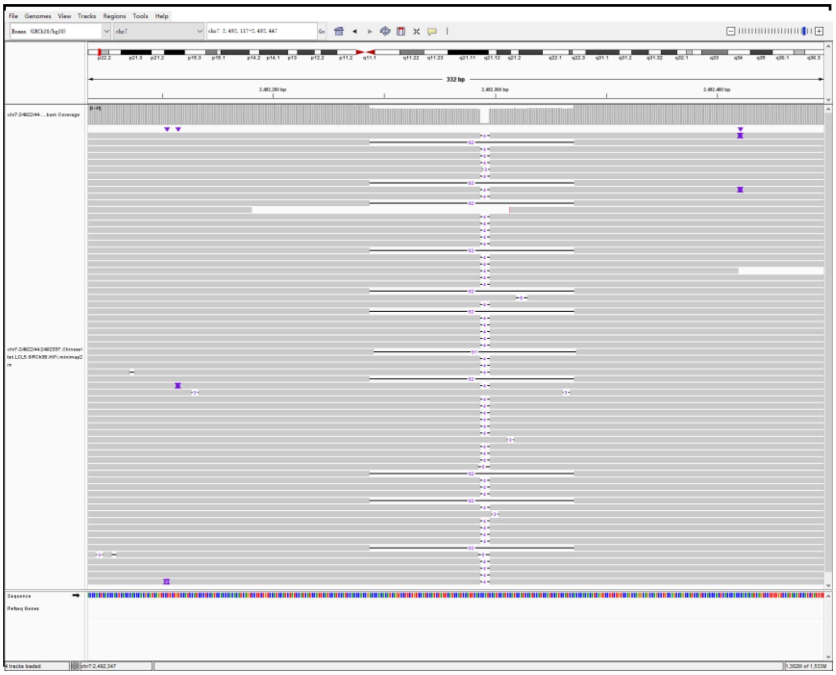

LCL7

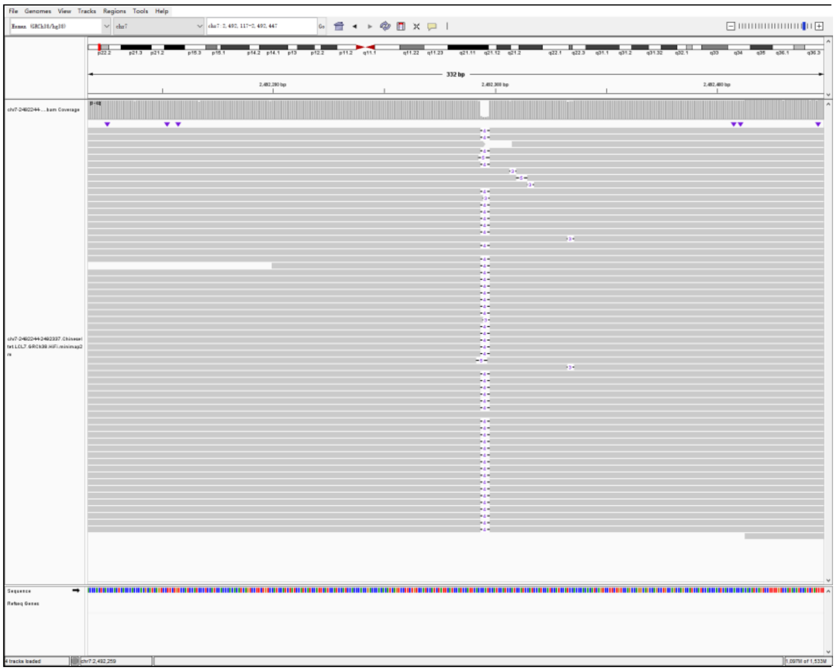

LCL8

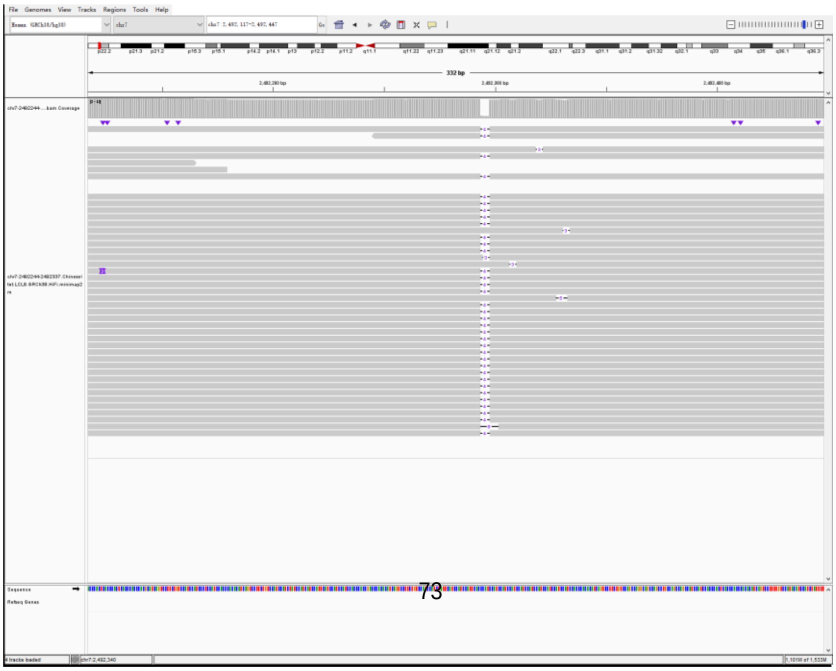

chr10 128035258 128035258 379 INS na19240-38-39 svision_pro

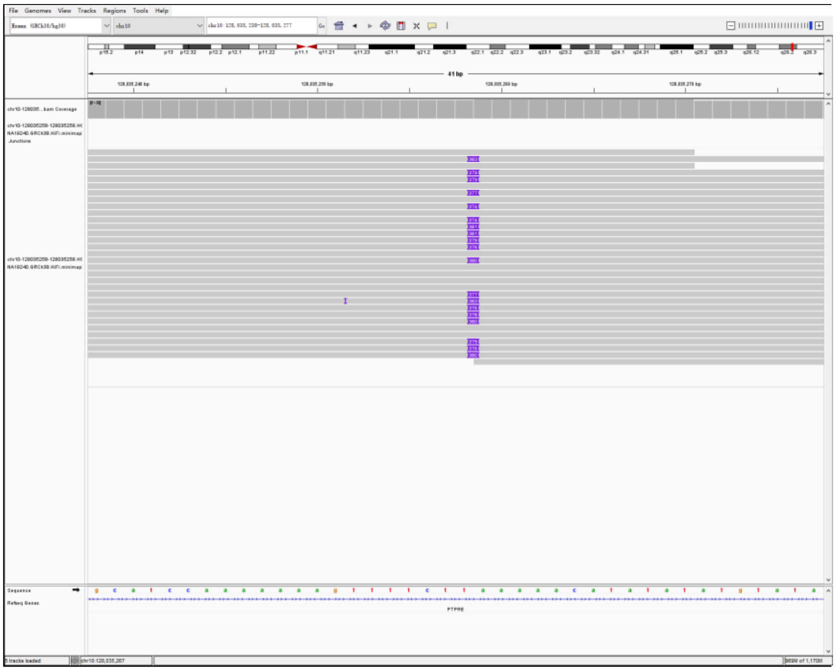

NA19240

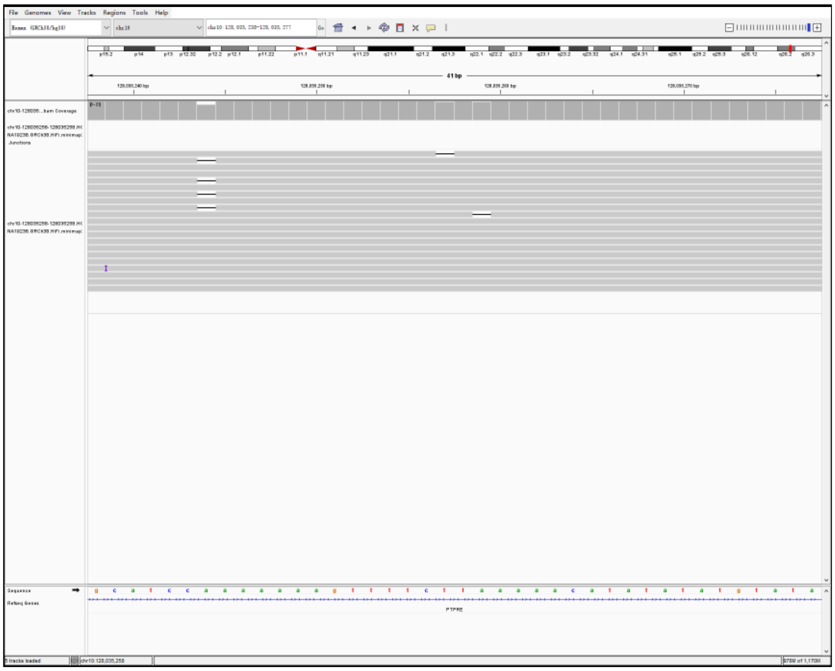

NA19238

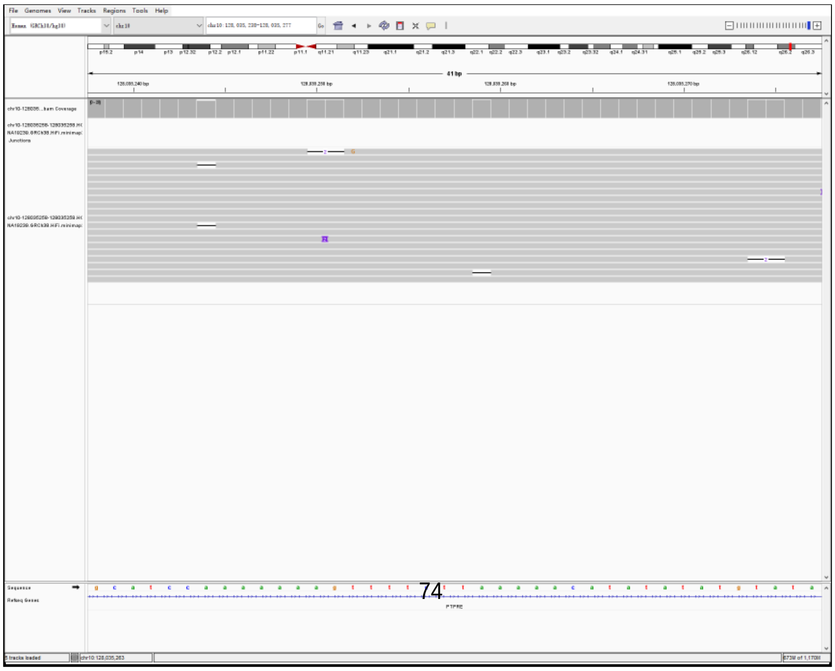

NA19239

chr16 30145094 30145149 55 DEL na19240-38-39 svision_pro

NA19240

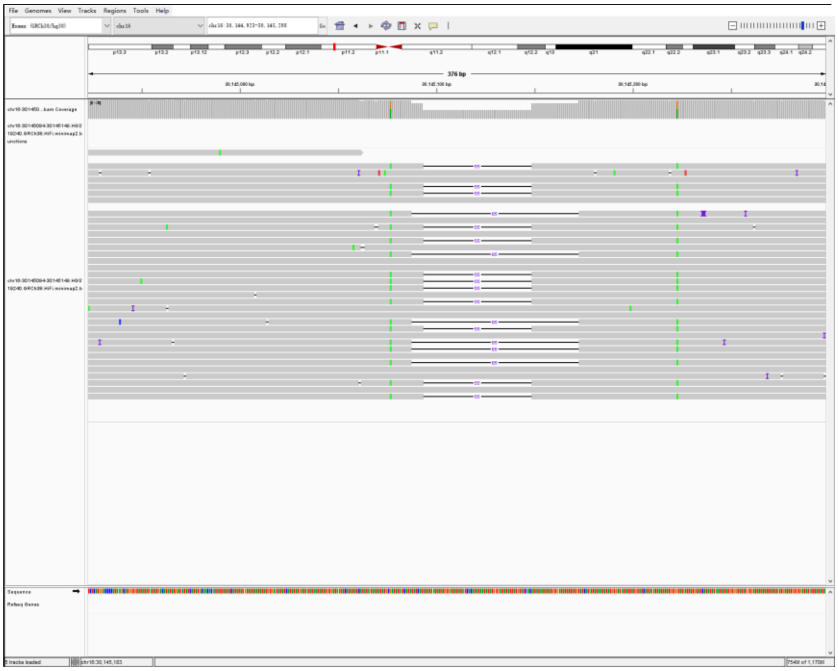

NA19238

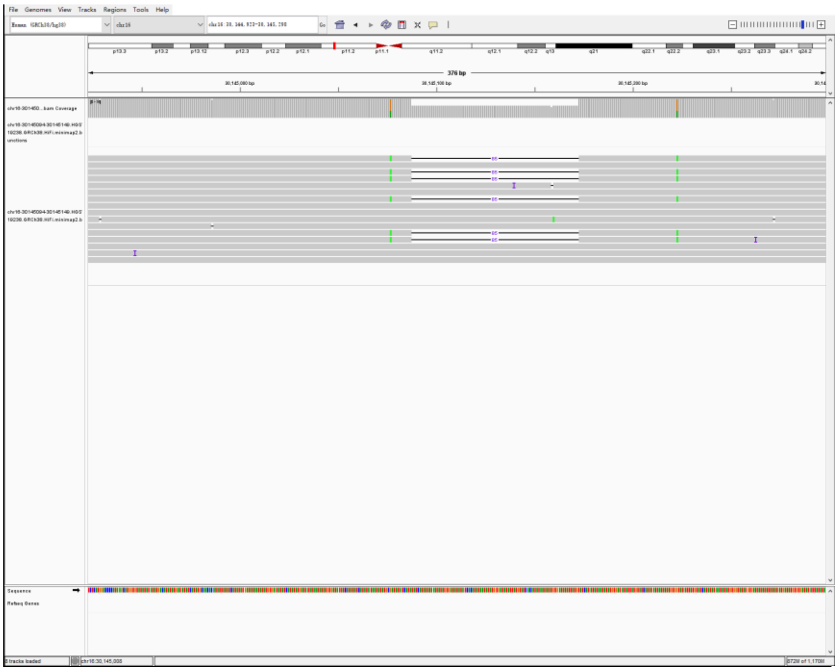

NA19239

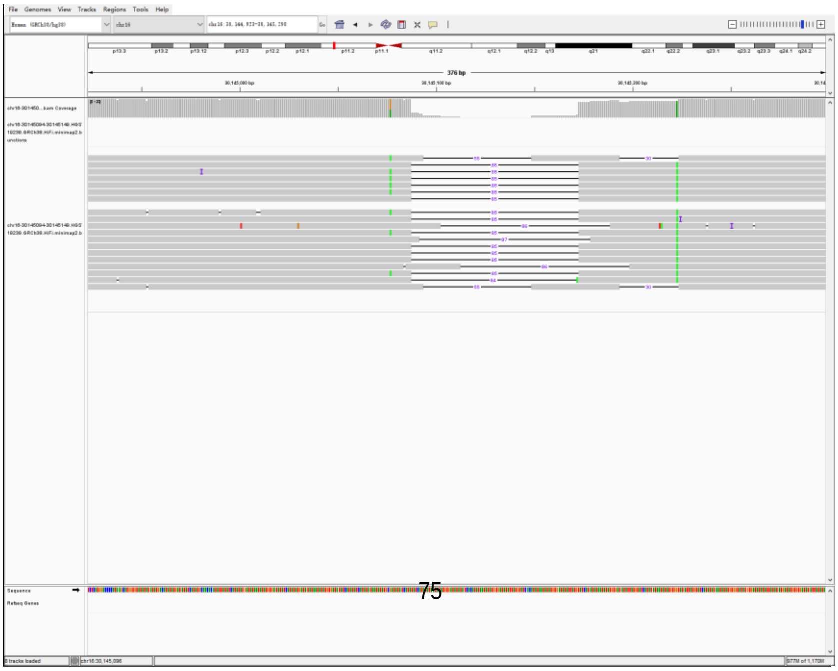

Supplementary File 5. Verification of 19 false-positive *de novo* calls of Sniffles2

chr2 34470763 34511502 40738 DEL hg002-3-4 sniffles2

HG002

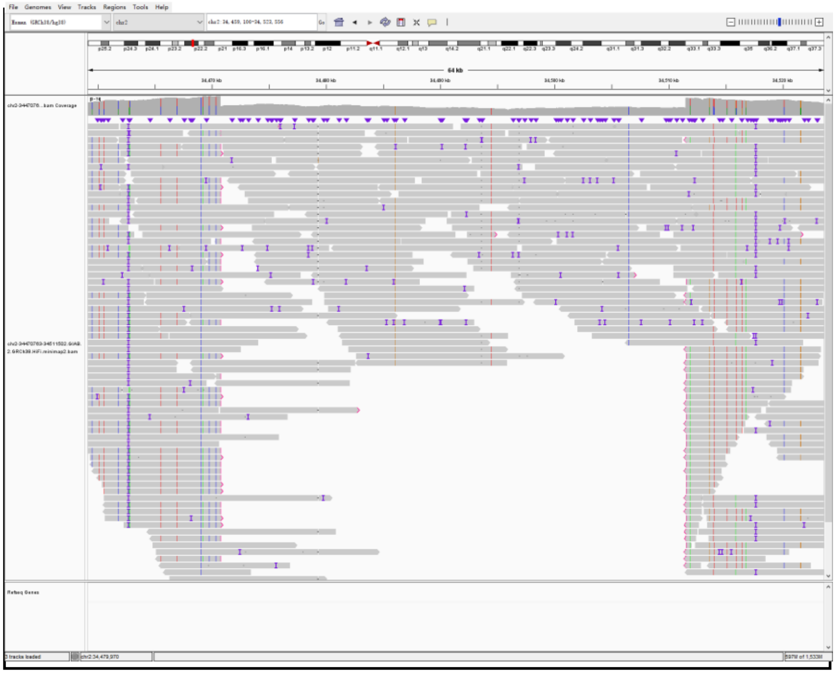

HG003

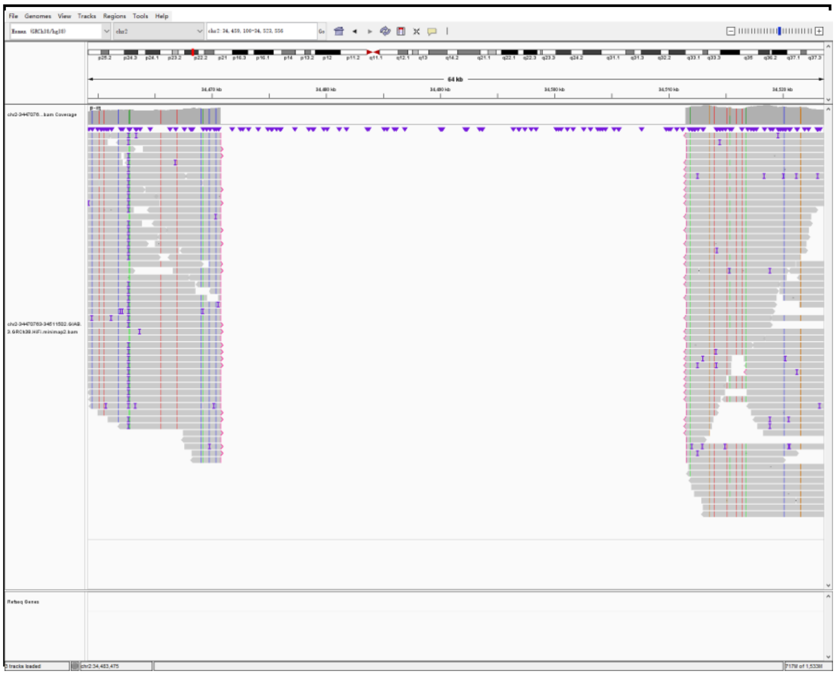

HG004

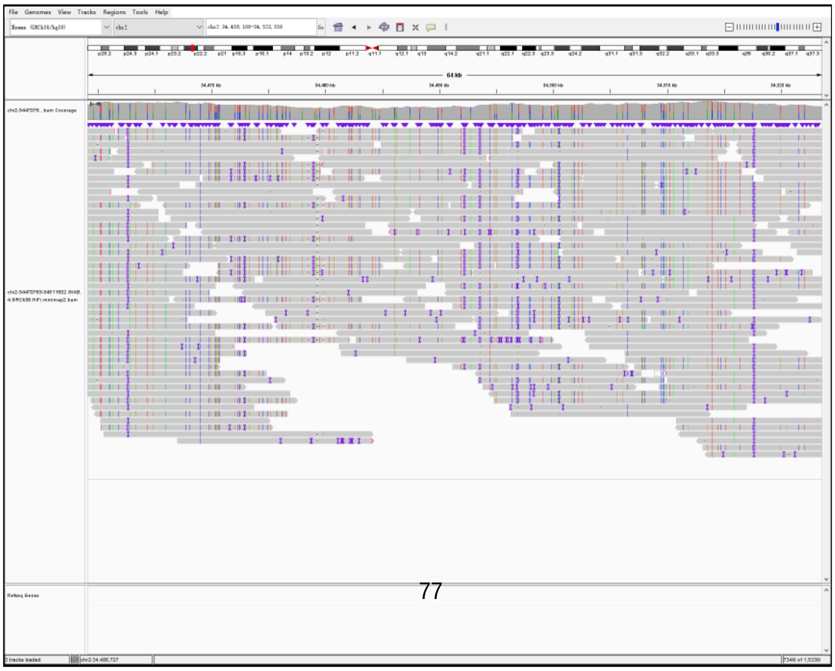

chr9 320274 320275 1586 INS hg002-3-4 sniffles2

HG002

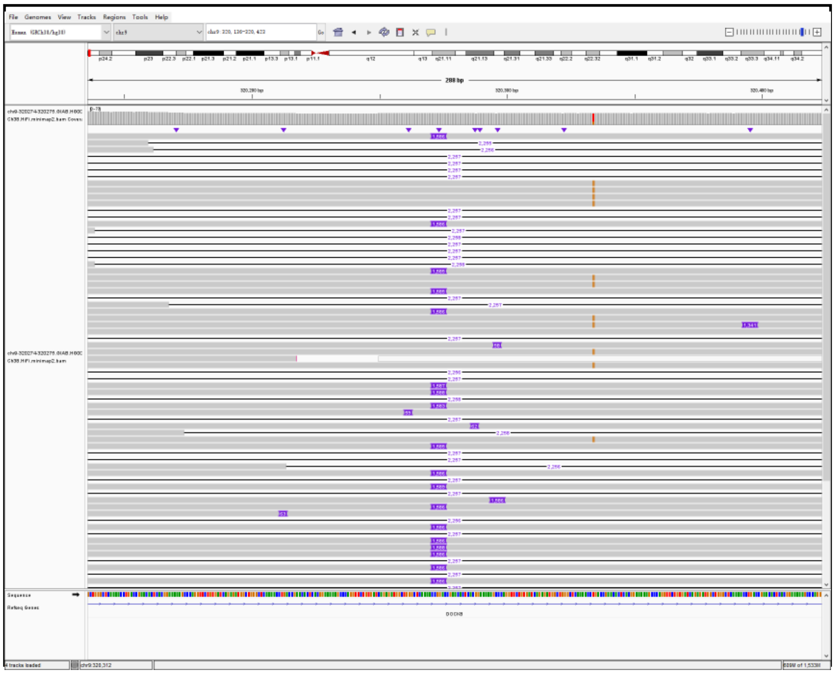

HG003

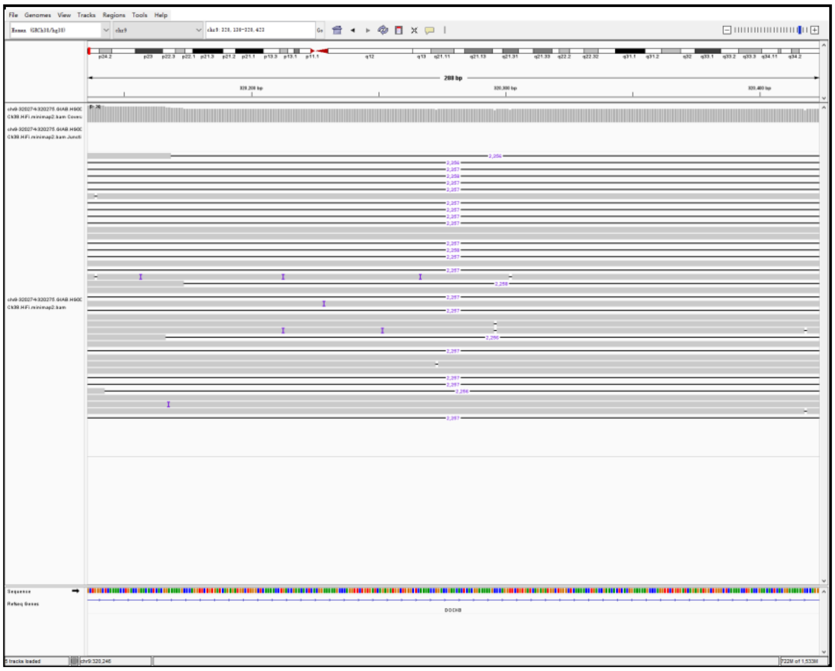

HG004

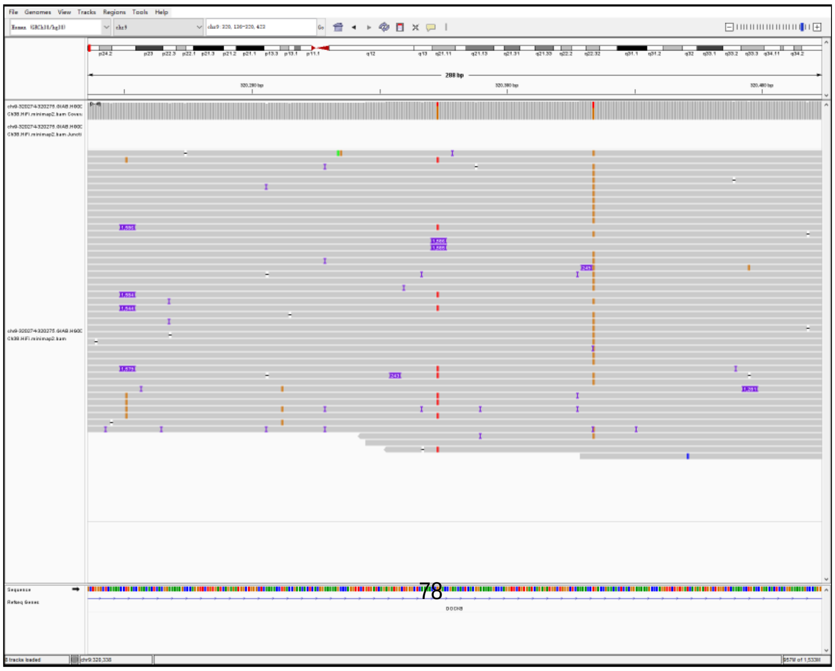

chr15 101542830 101542831 3961 INS hg002-3-4 sniffles2

HG002

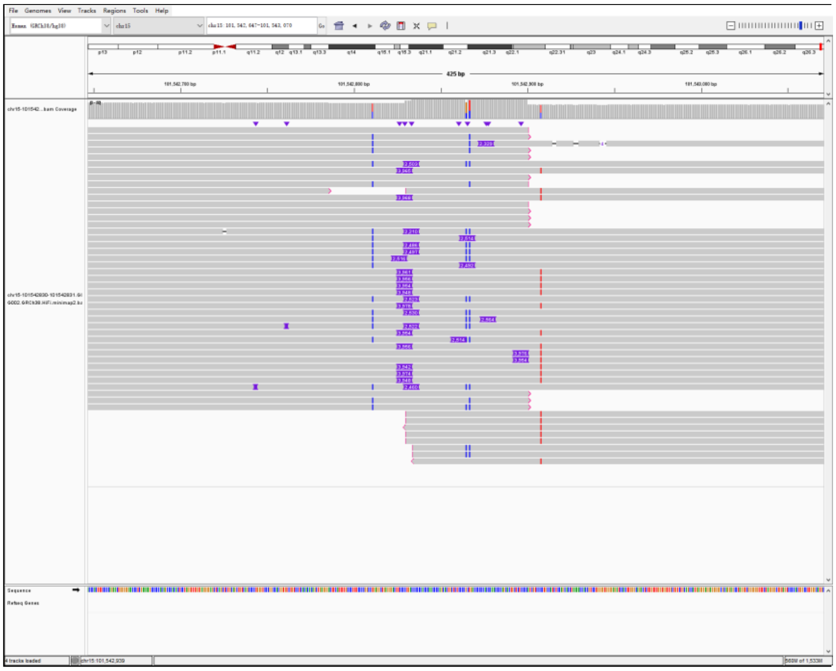

HG003

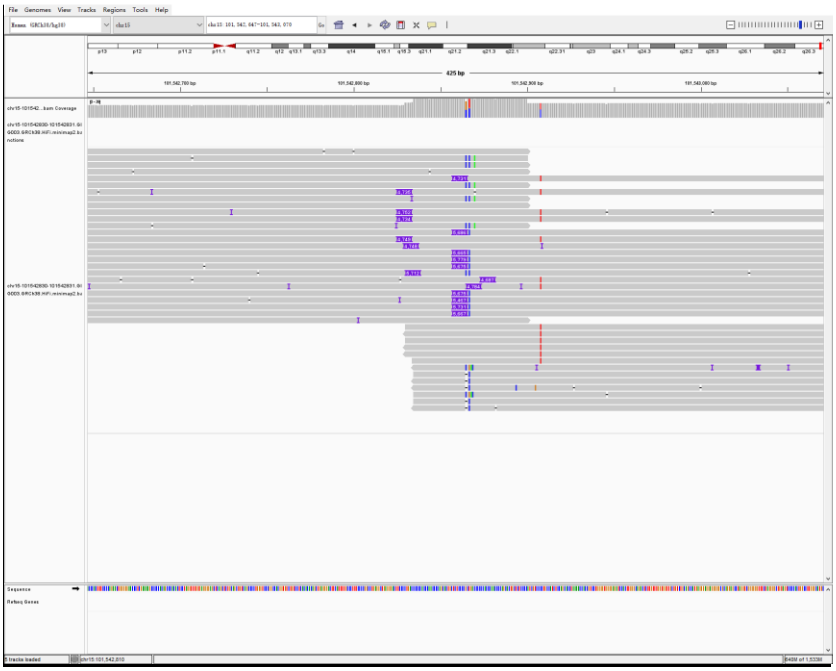

HG004

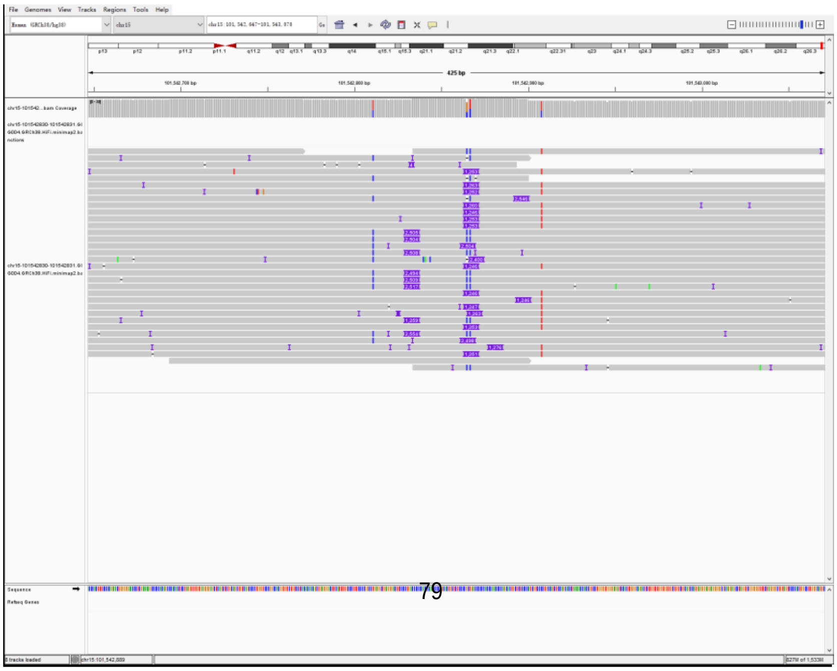

chr5 126164035 126164036 1503 INS hg005-6-7 sniffles2

HG005

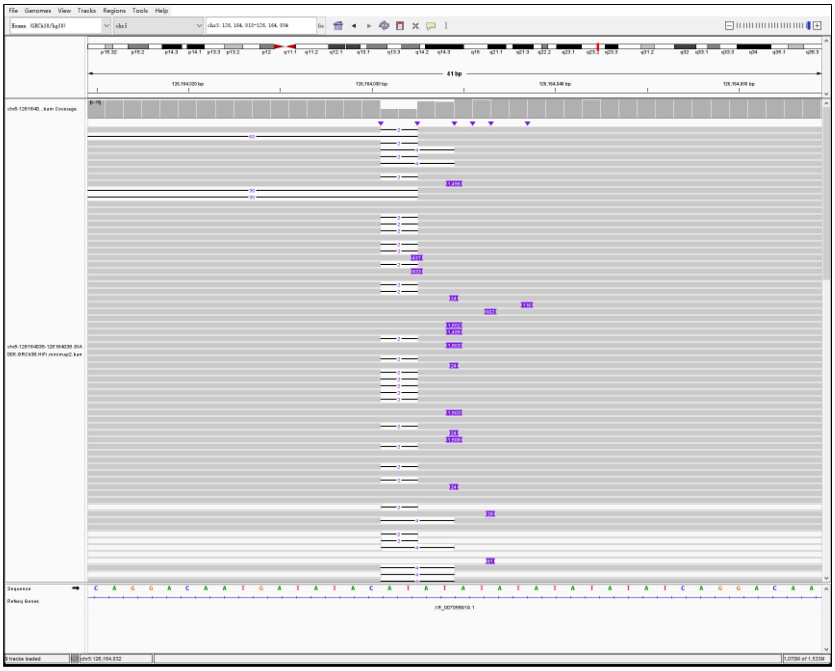

HG006

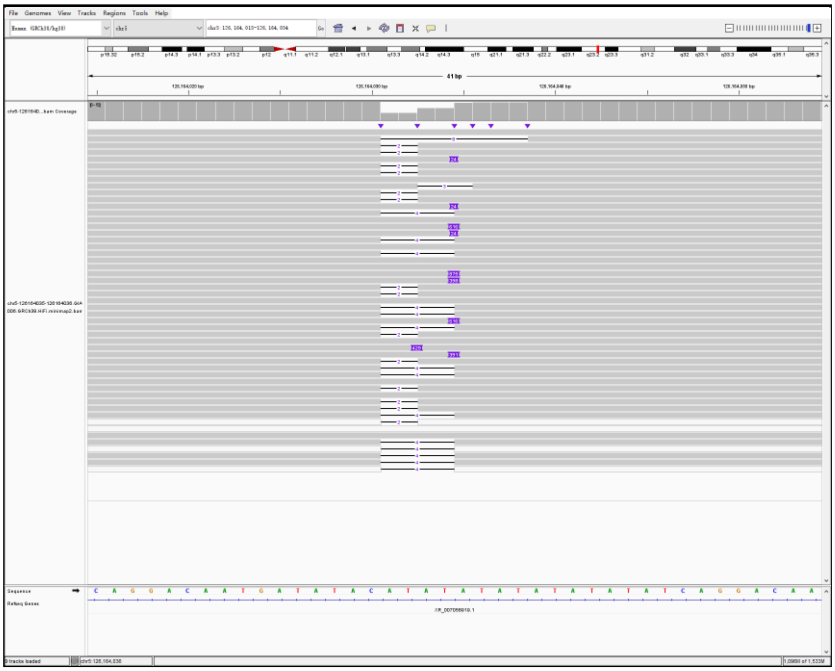

HG006

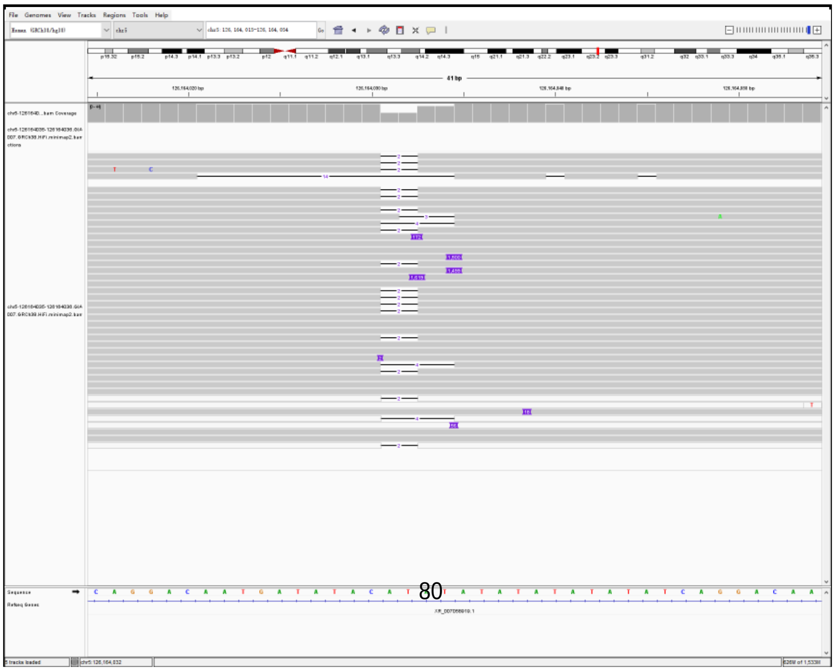

chr7 155891365 155891366 3527 INS hg005-6-7 sniffles2

HG005

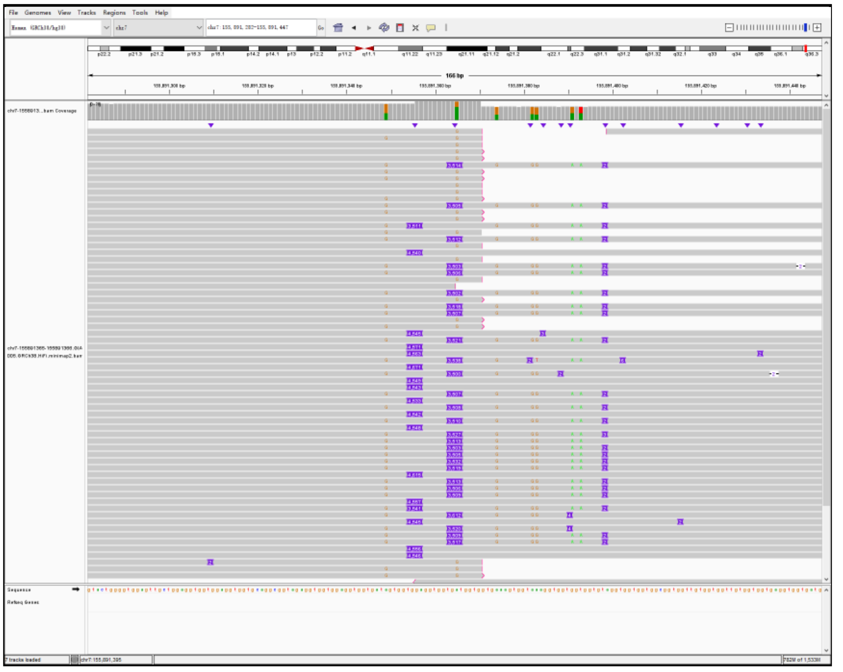

HG006

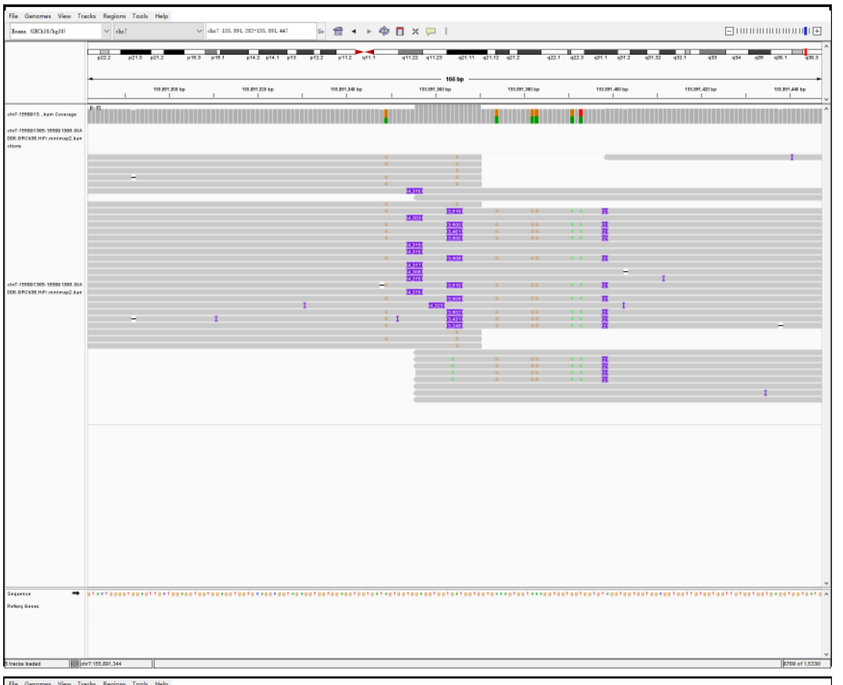

HG006

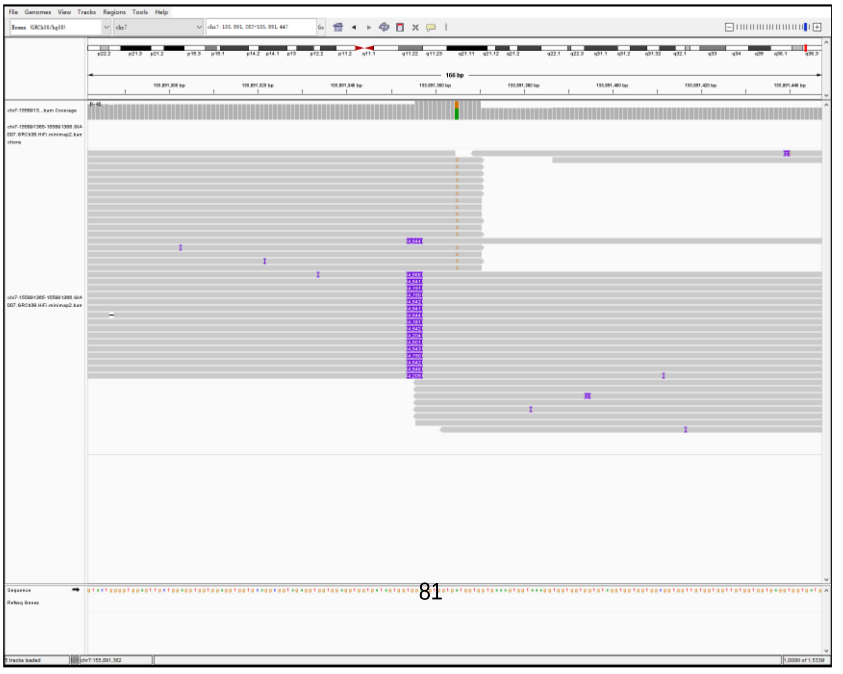

chr9 76305063 76306926 1862 DEL hg005-6-7 sniffles2

HG005

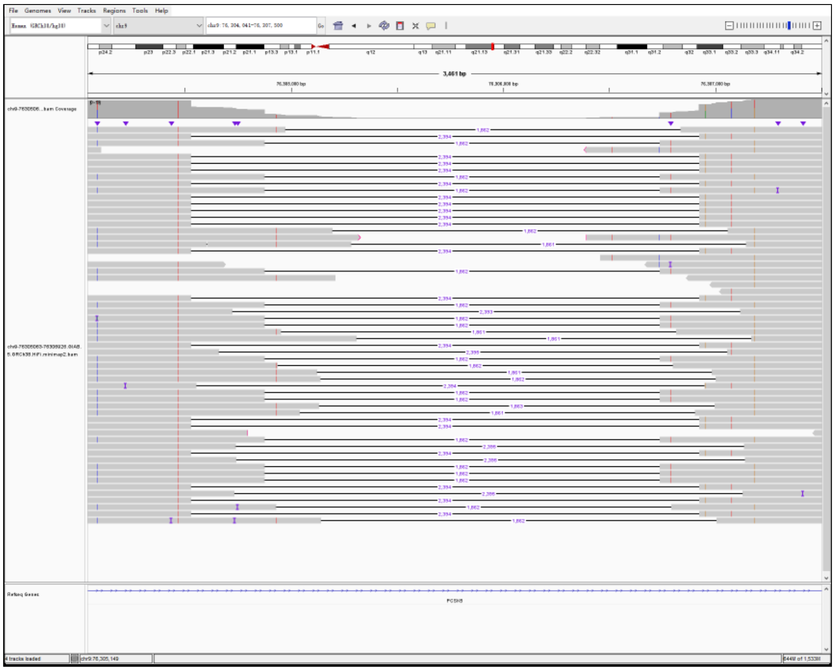

HG006

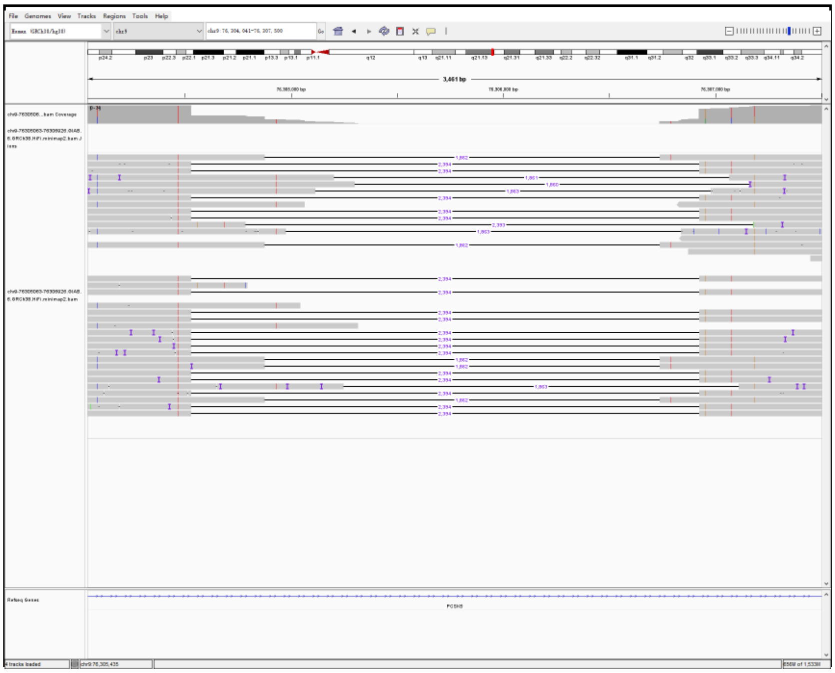

HG006

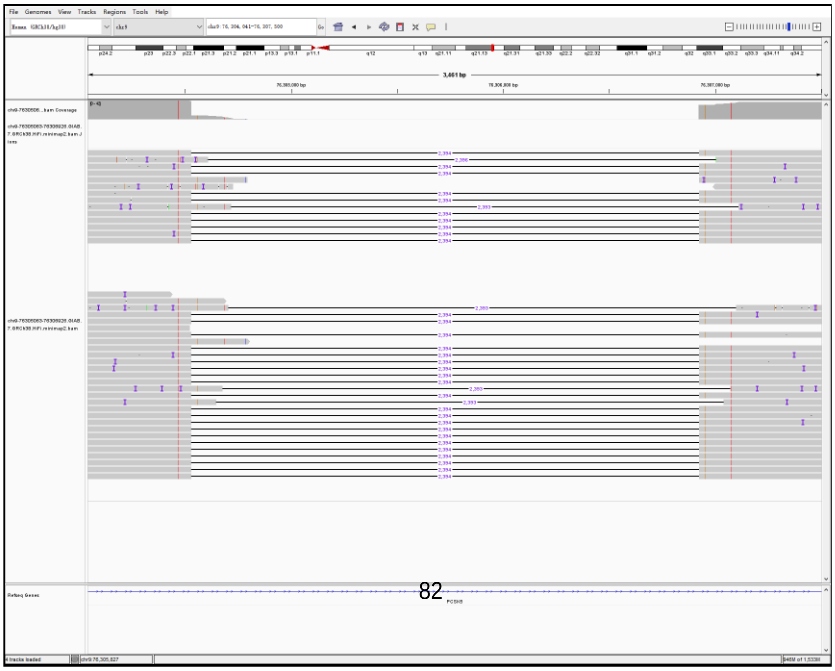

chr9 130958792 130958793 3714 INS hg005-6-7 sniffles2

HG005

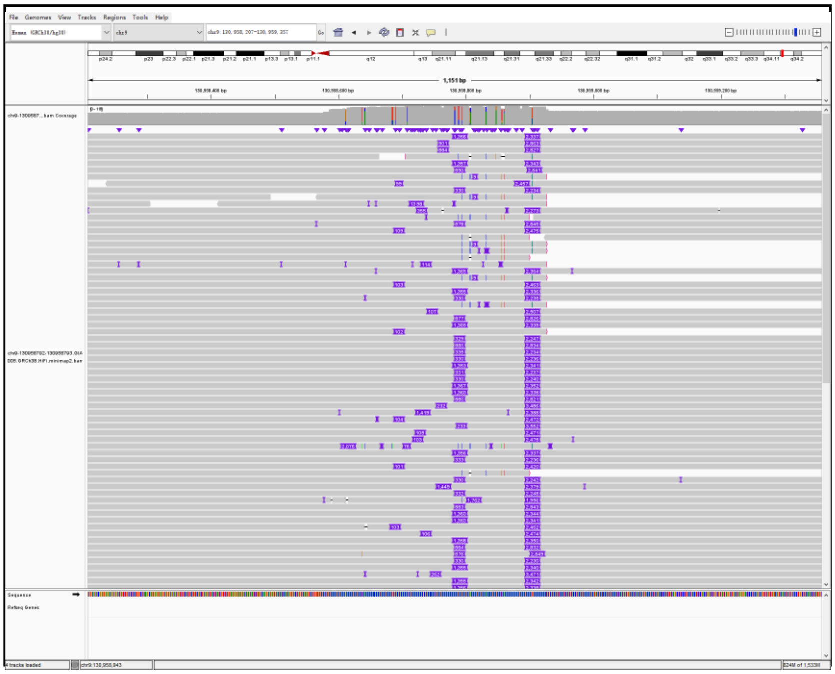

HG006

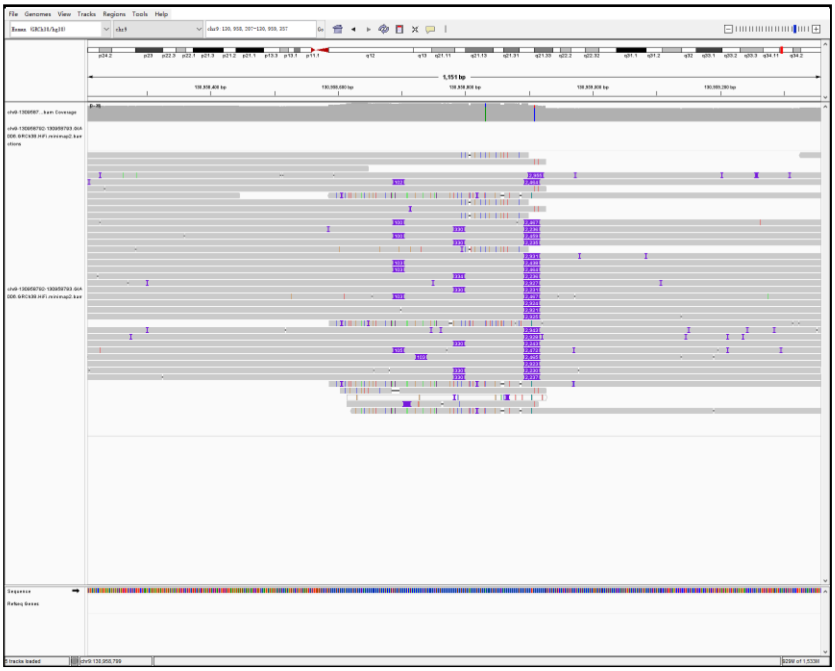

HG006

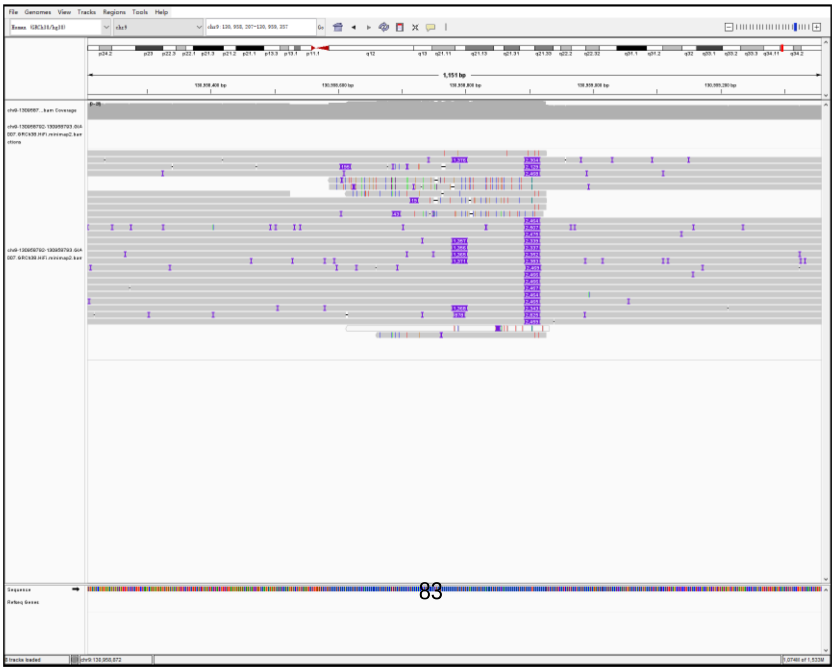

chr11	11246952	11246953	2946	INS	hg005-6-7	sniffles2

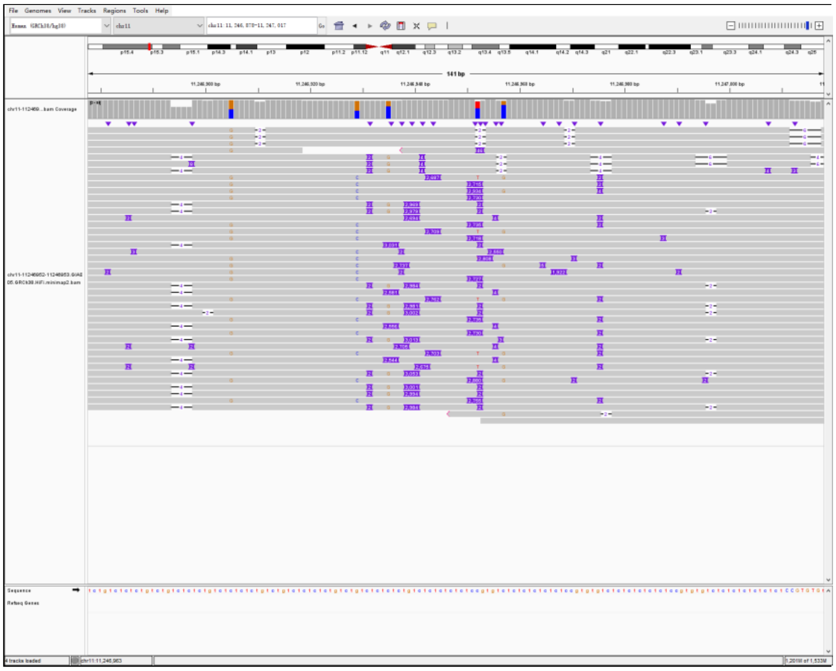

HG005

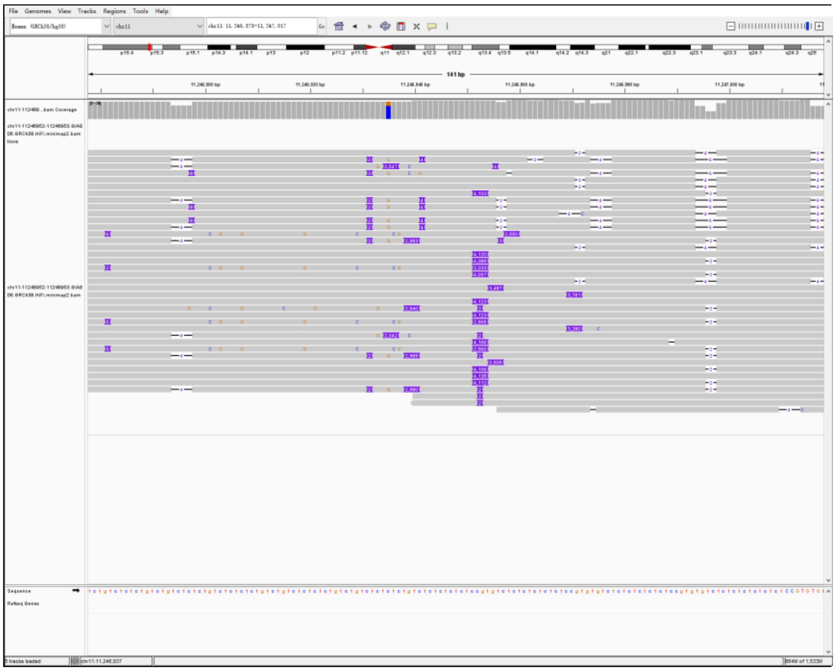

HG006

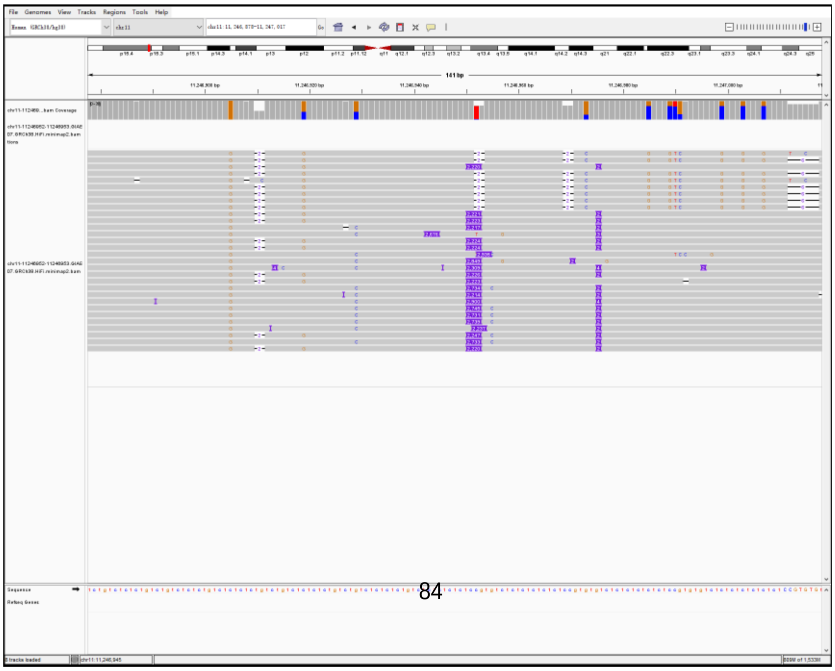

HG006

chr16	68674969	68674970	2927	INS	hg005-6-7	sniffles2

HG005

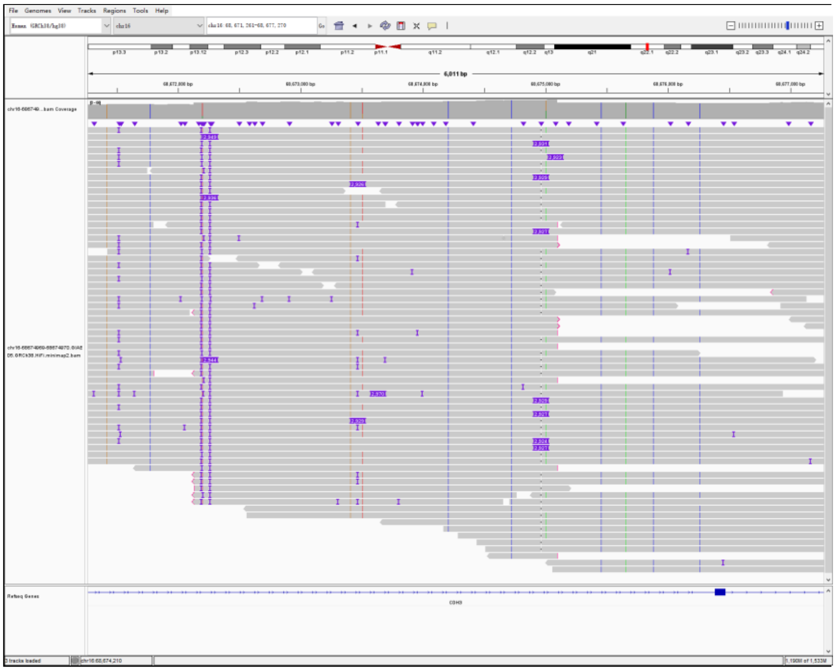

HG006

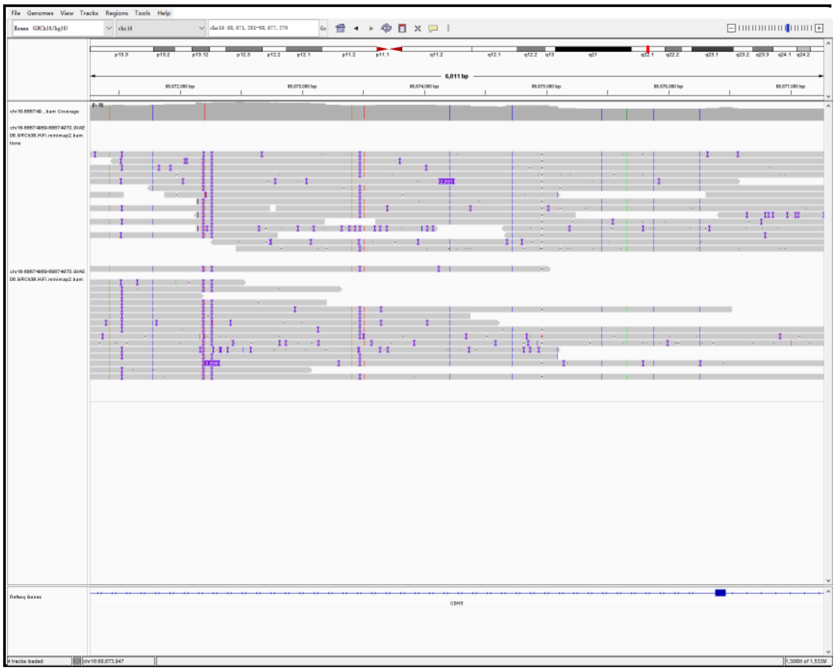

HG006

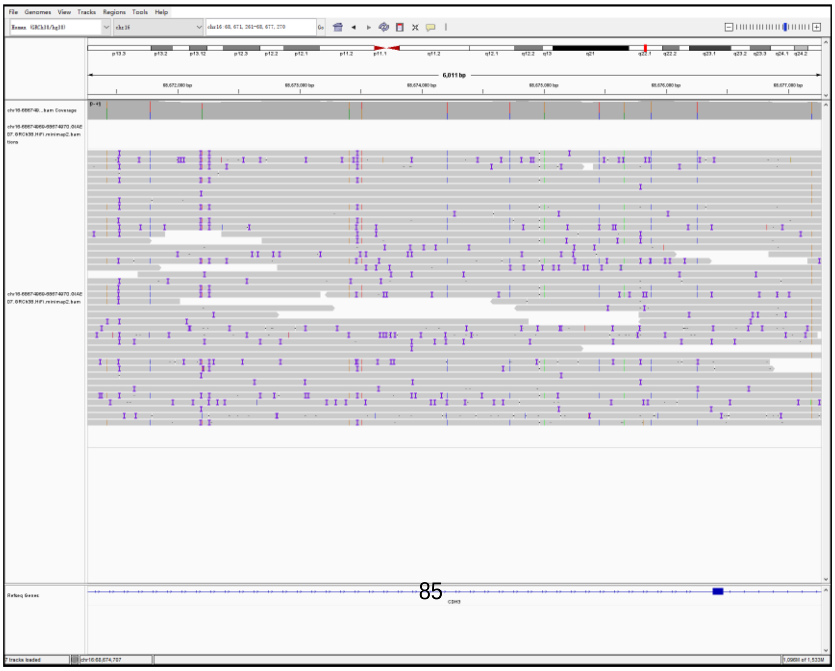

chr7 155328607 155328608 631 INS hg00514-2-3 sniffles2

HG00514

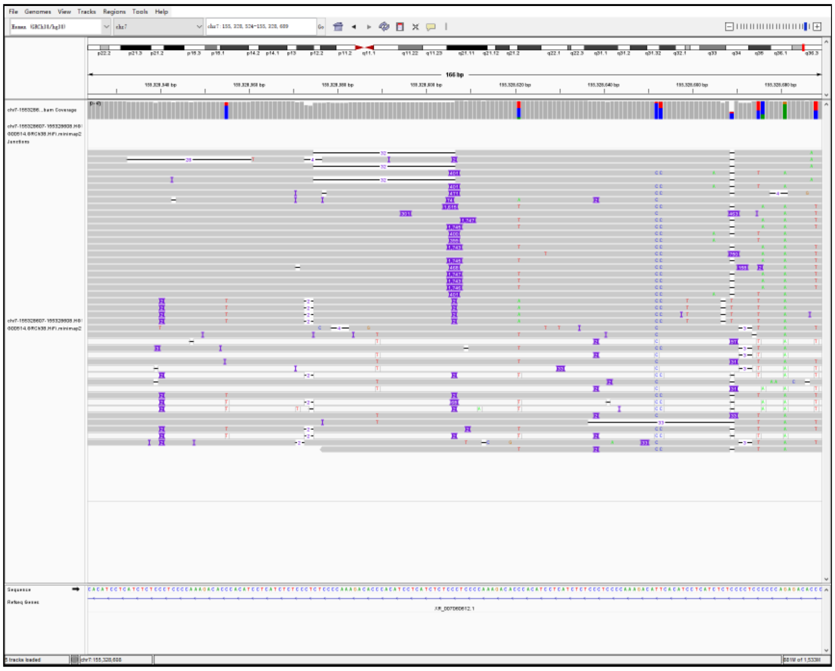

HG00512

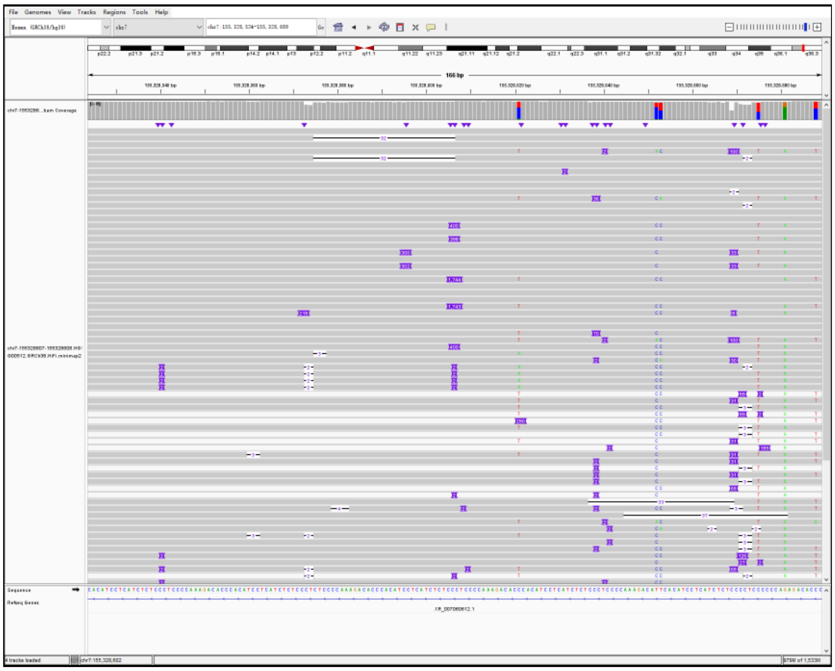

HG00513

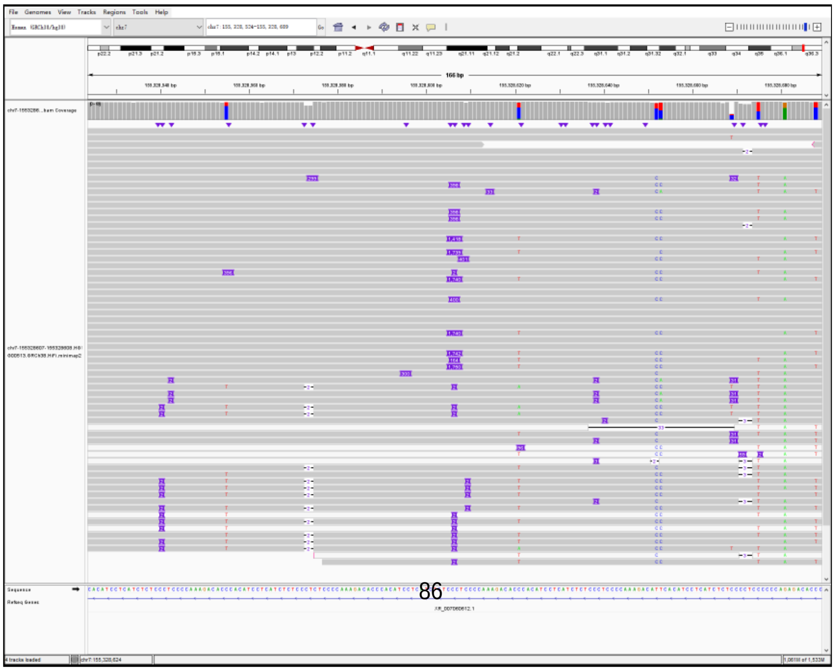

chr2 52522549 52558134 35584 DEL hg00733-1-2 sniffles2

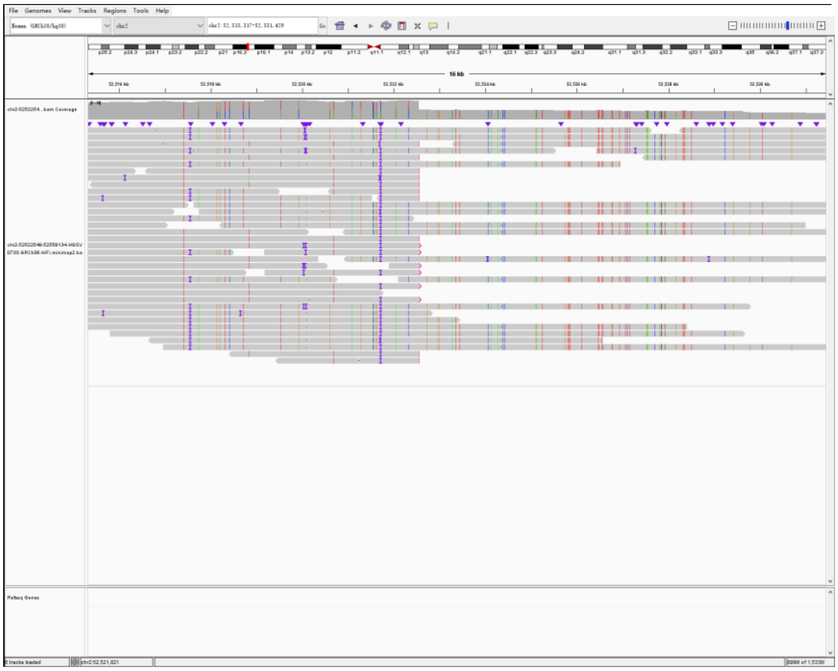

HG00733

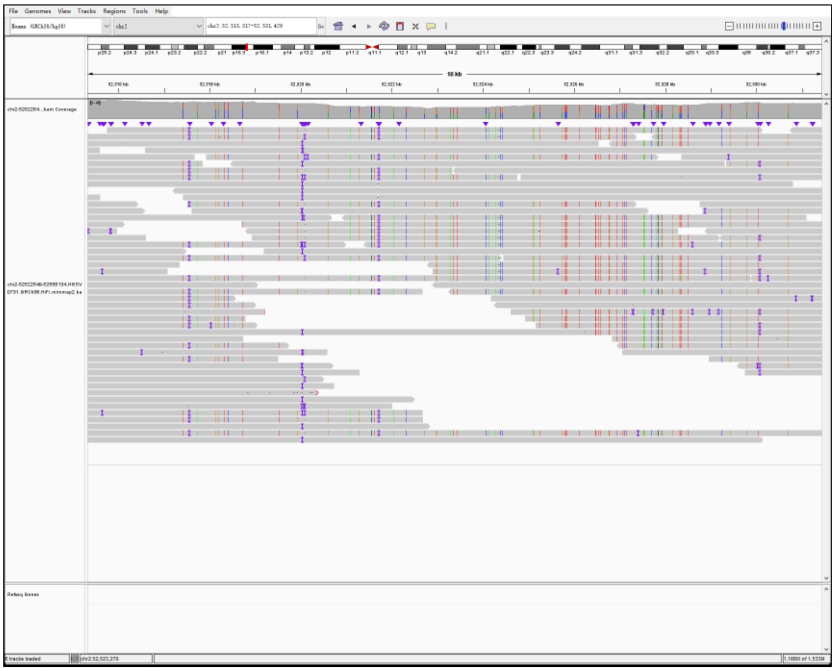

HG00731

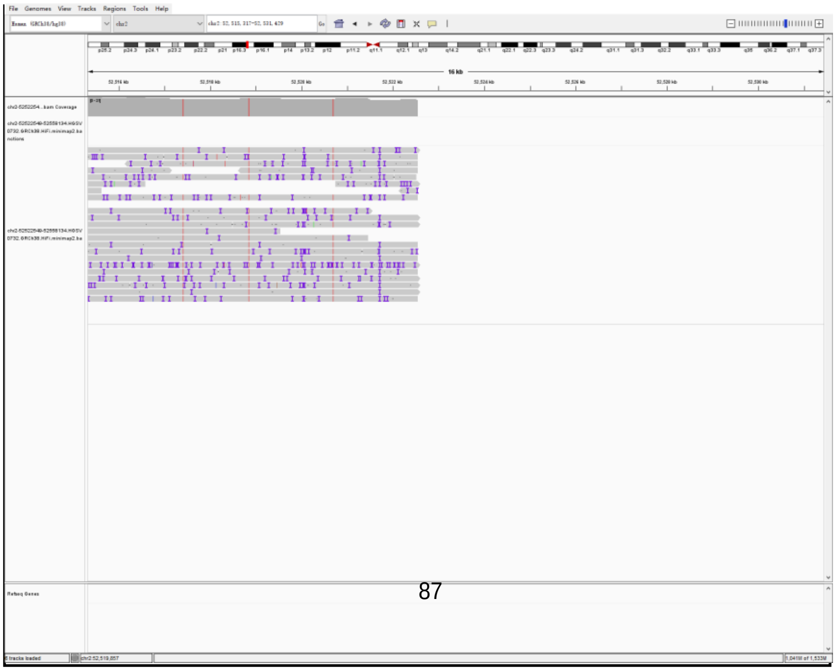

HG00732

chr2 193680304 193680305 3349 INS hg00733-1-2 sniffles2

HG00733

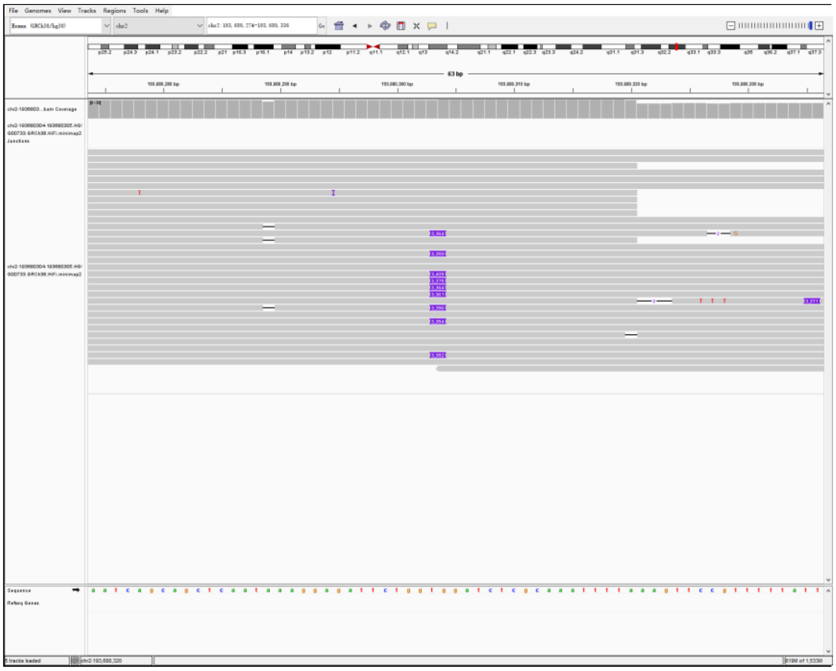

HG00731

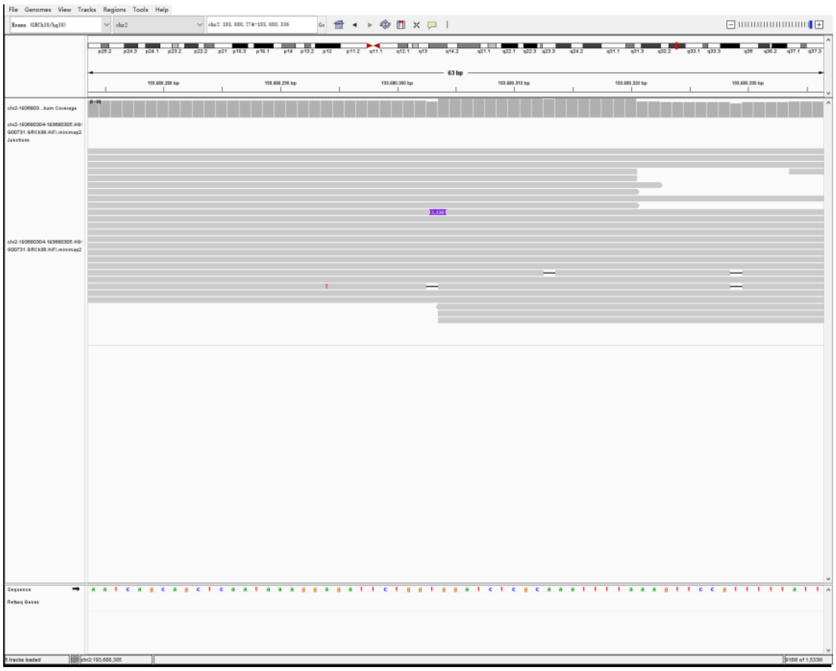

HG00732

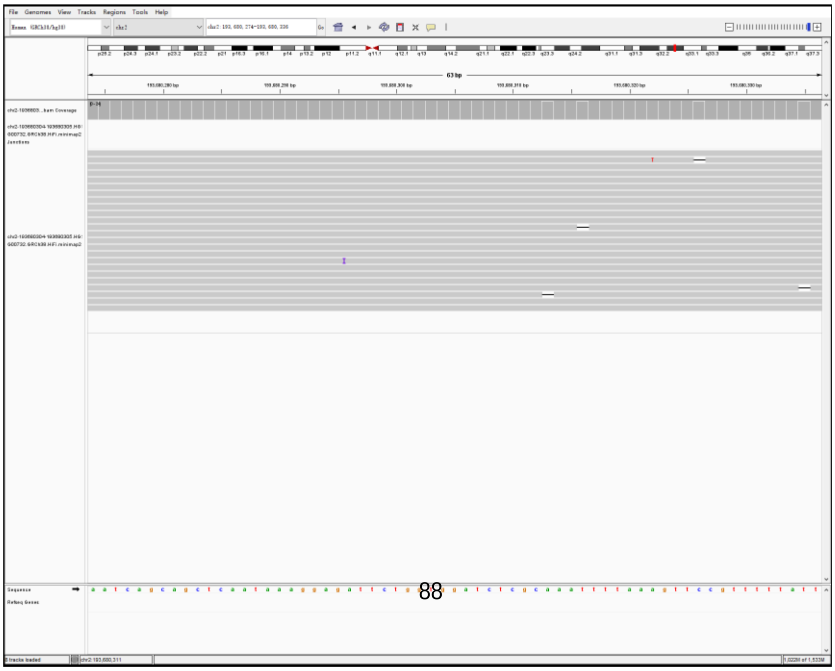

chr4 189235052 189235053 102 INS hg00733-1-2 sniffles2 PRECISE

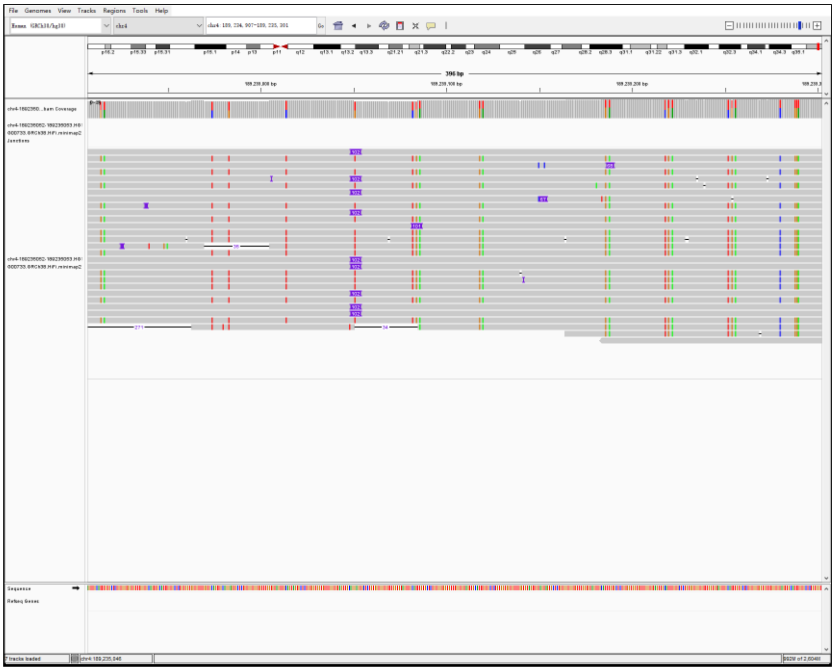

HG00733

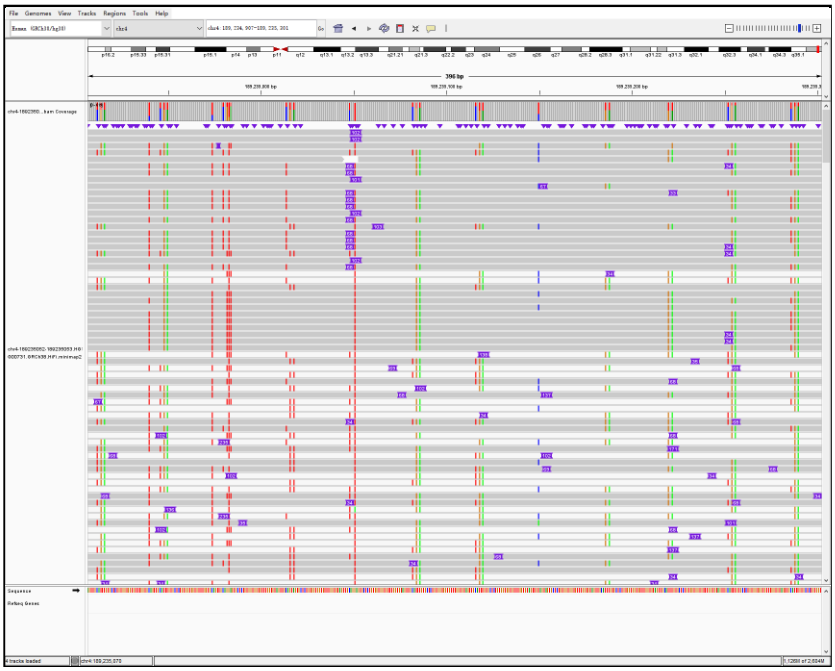

HG00731

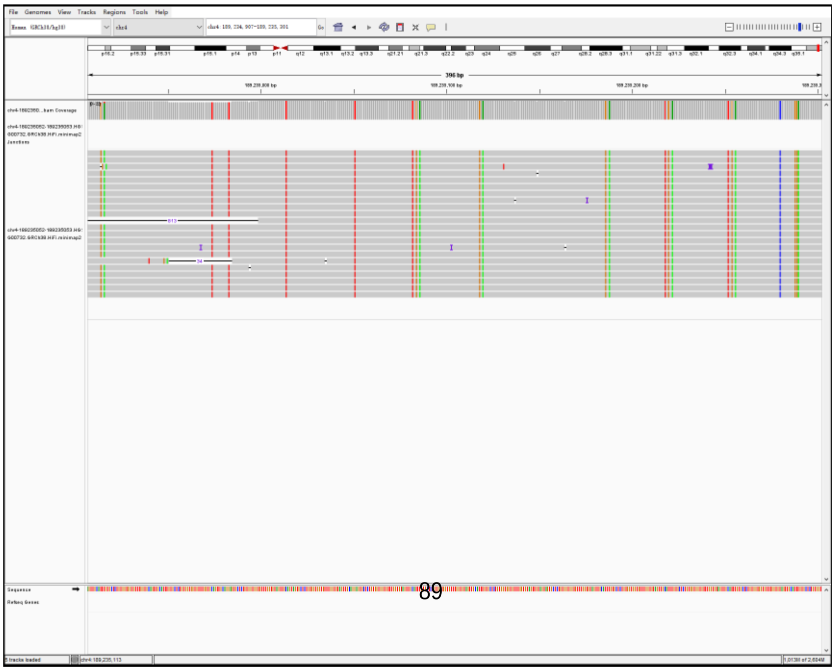

HG00732

chr7 158916029 158916030 4245 INS hg00733-1-2 sniffles2

HG00733

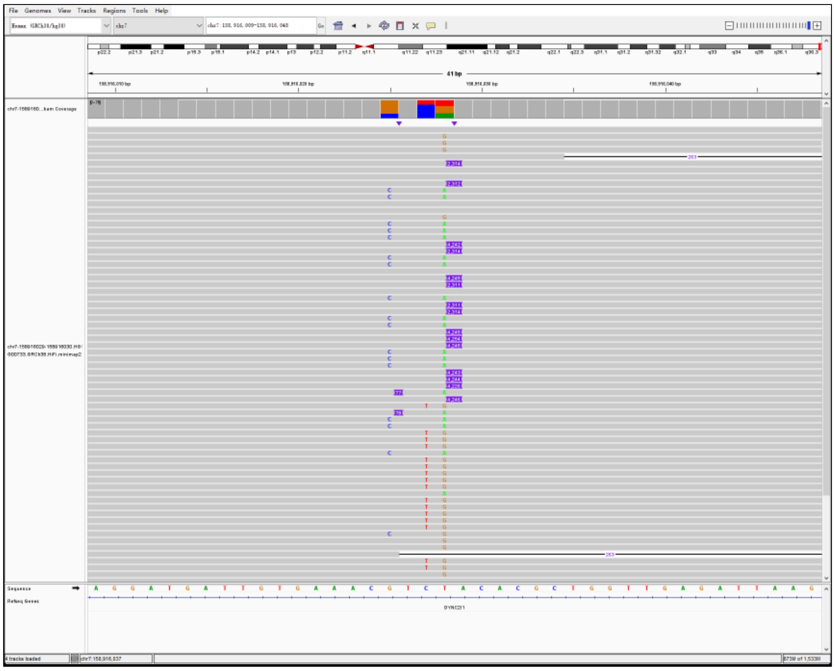

HG00731

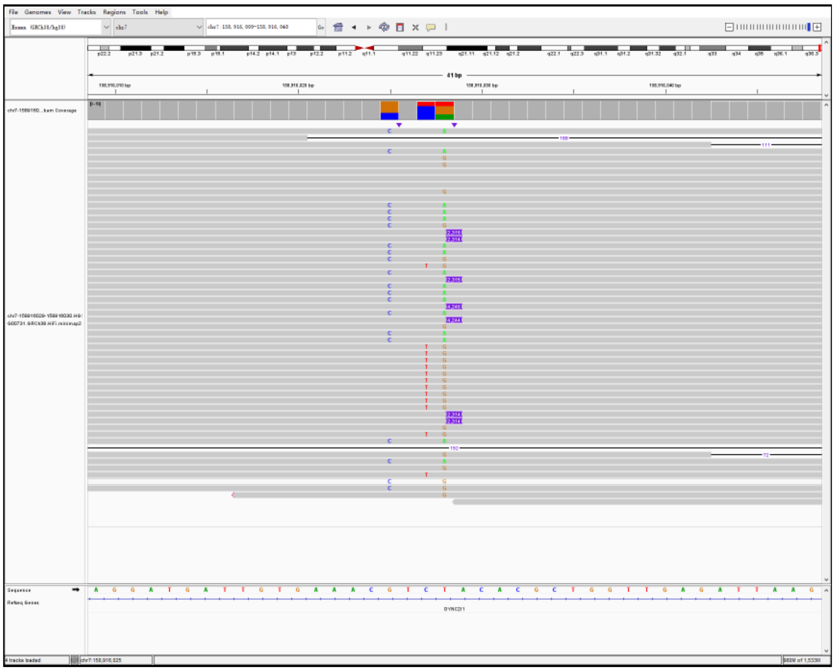

HG00732

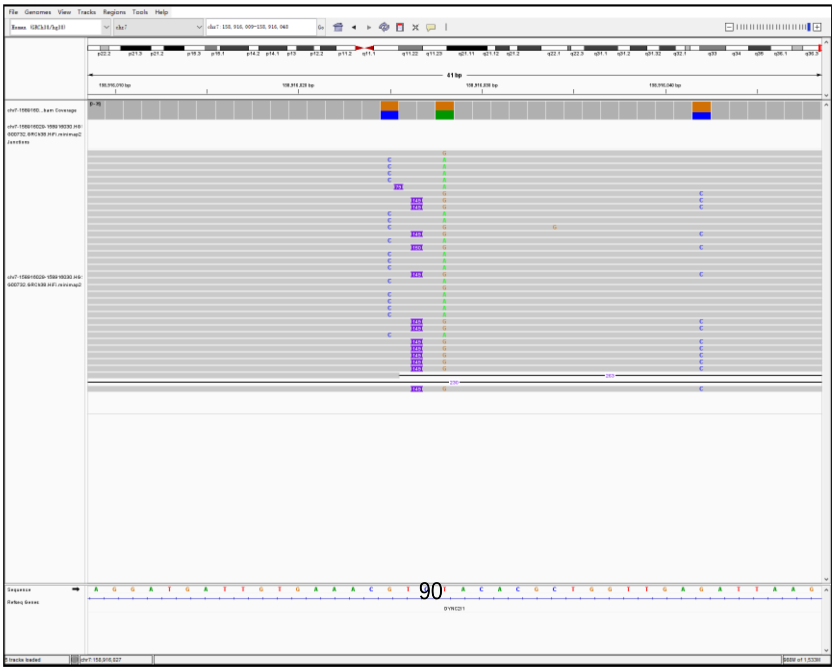

chr14 104300915 104300916 6112 INS hg00733-1-2 sniffles2

HG00733

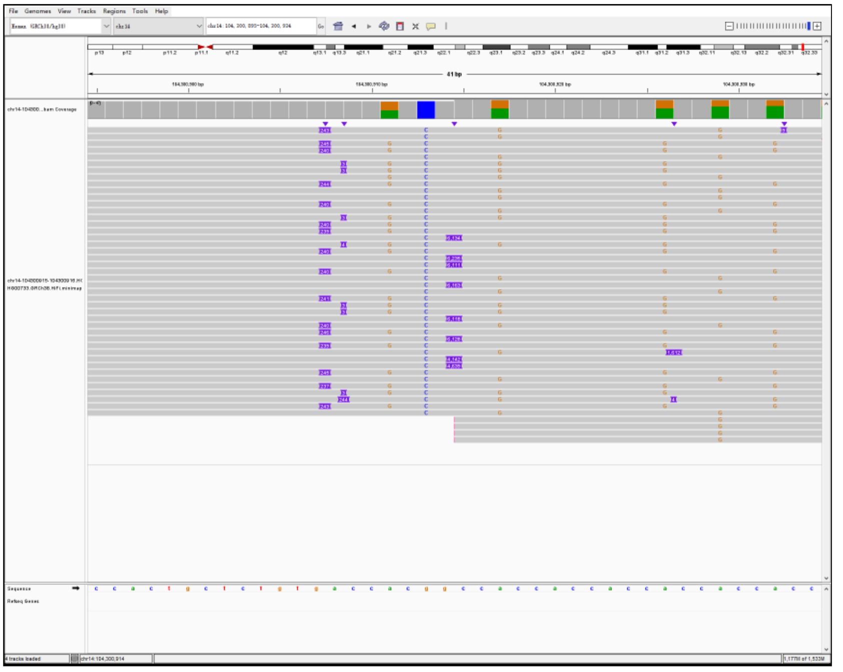

HG00731

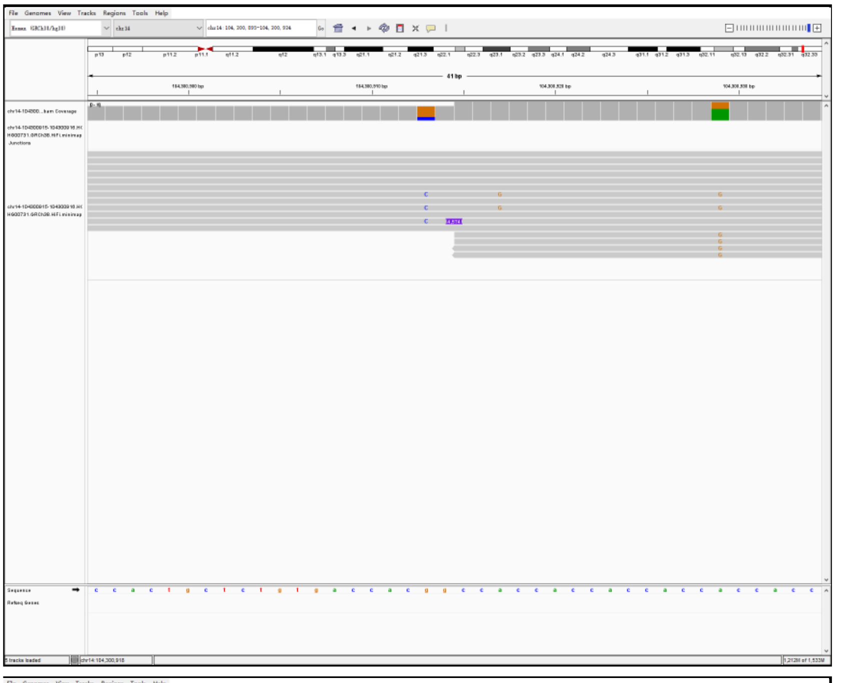

HG00732

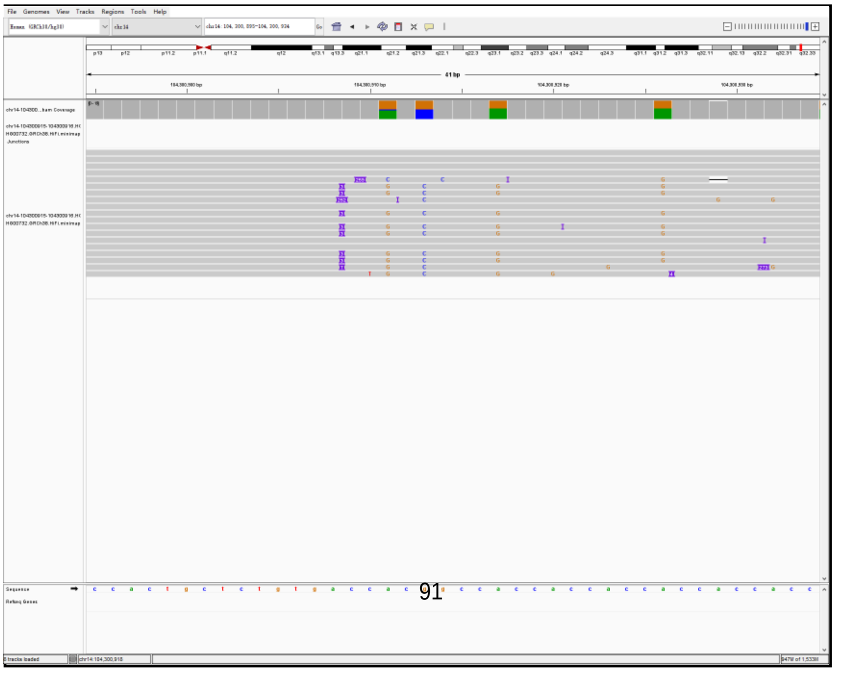

chr13 57178407 57214731 36323 DEL lcl5-6-7-8 sniffles2

LCL5

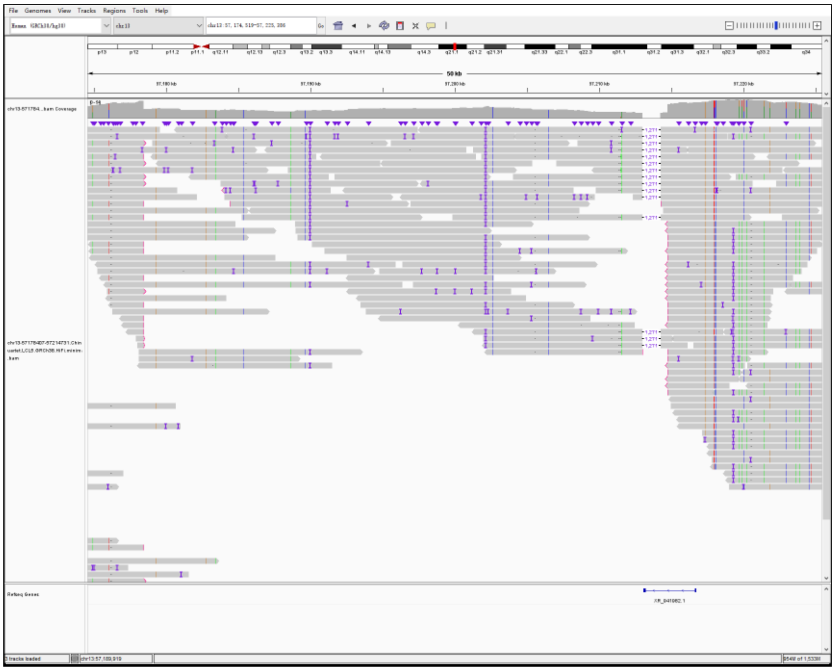

LCL7

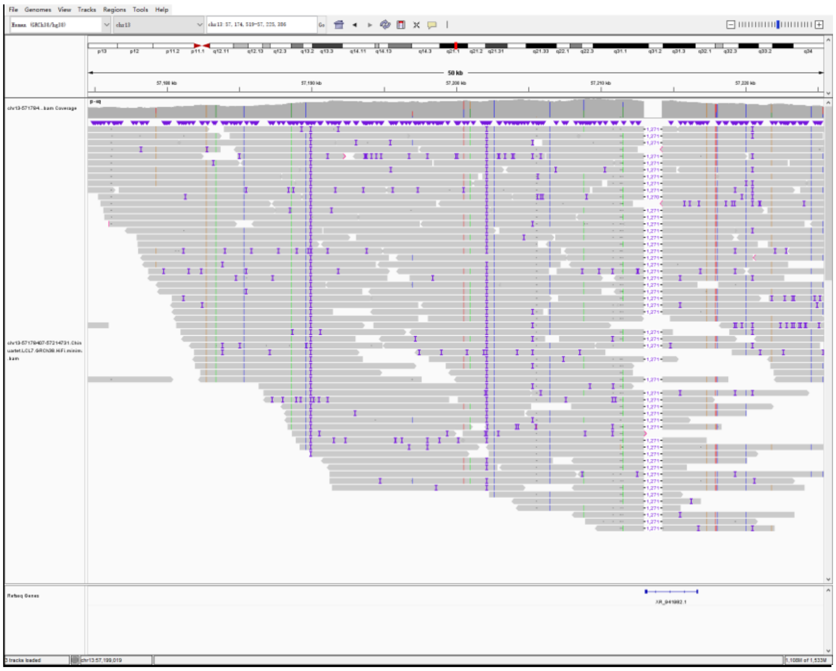

LCL8

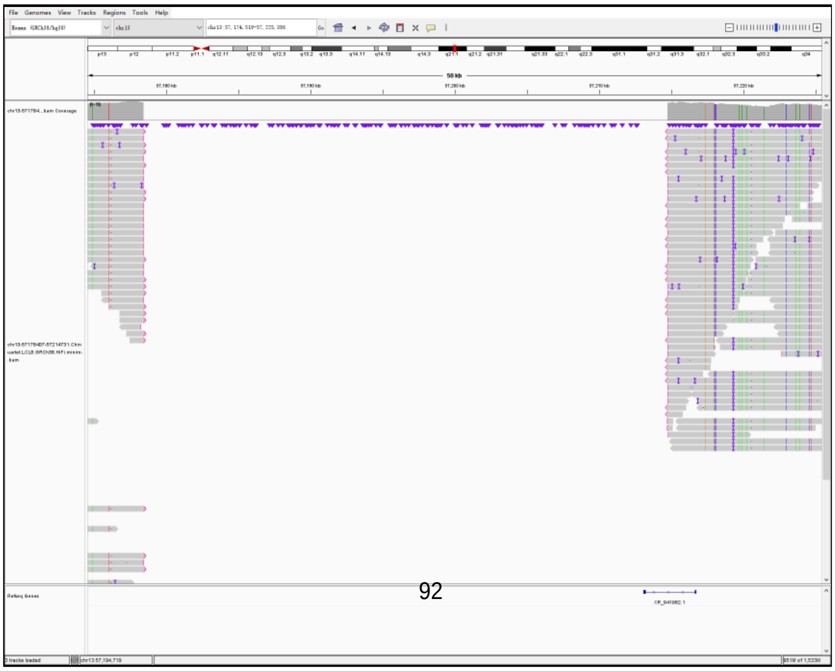

chr6 103289590 103315016 25425 DEL na19240-38-39 sniffles2

NA19240

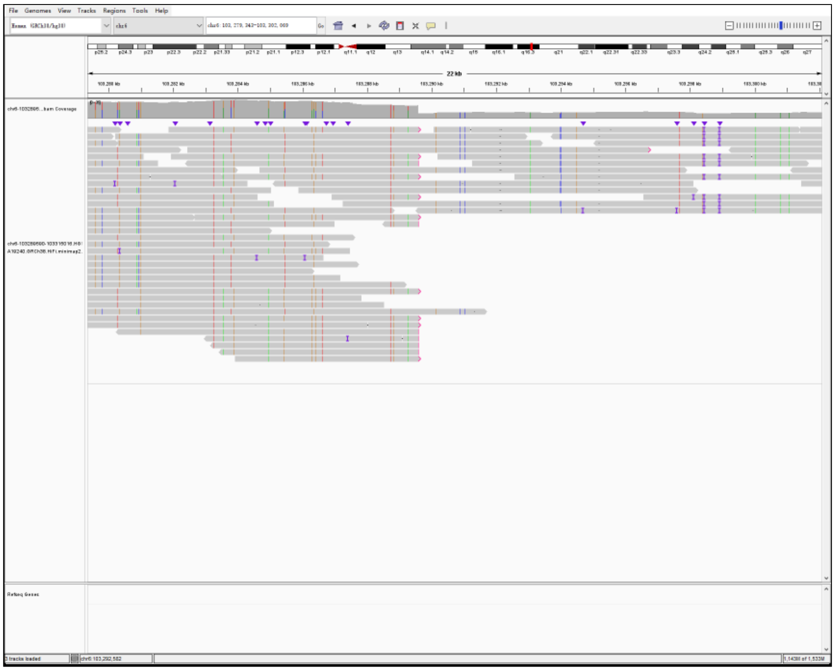

NA19238

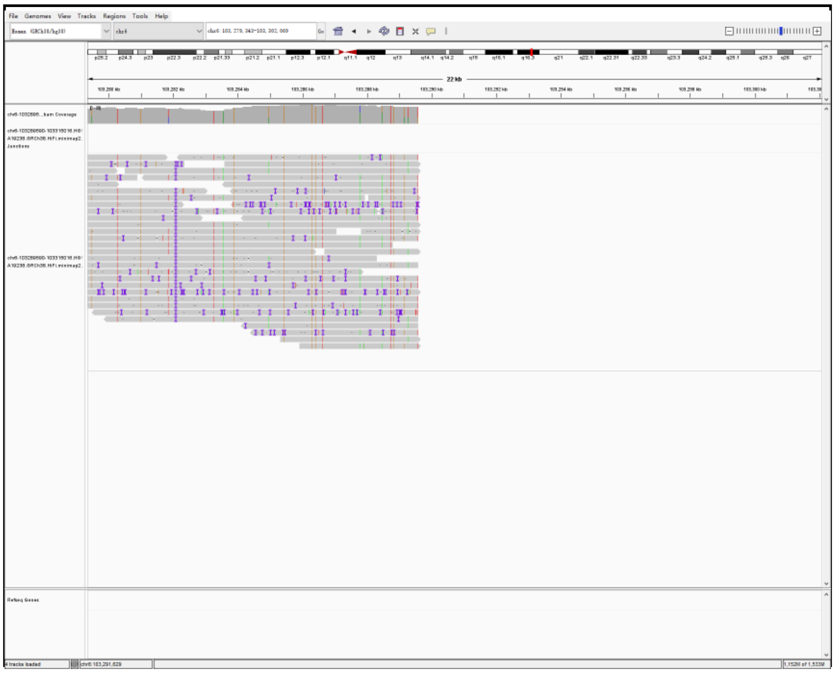

NA19239

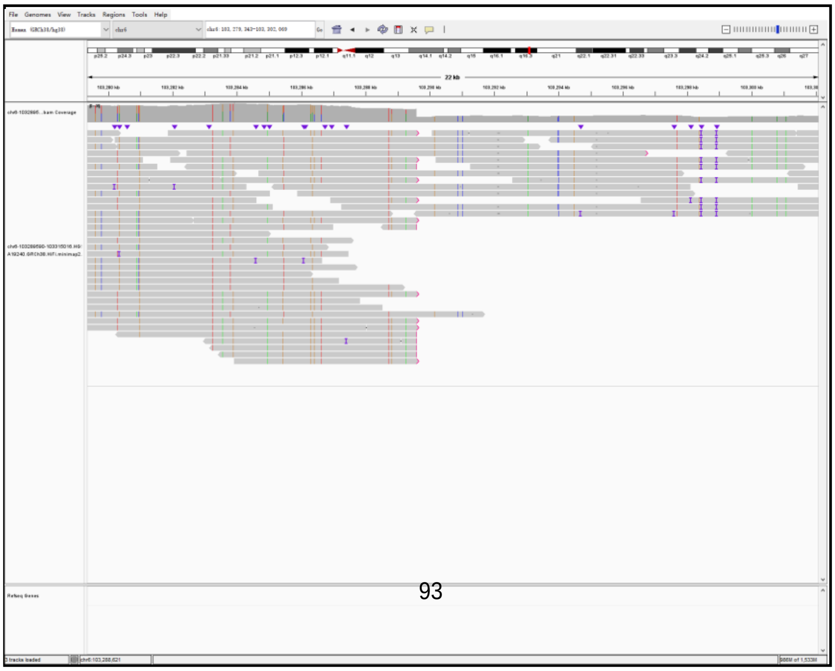

chr20 61389838 61389839 794 INS na19240-38-39 sniffles2

NA19240

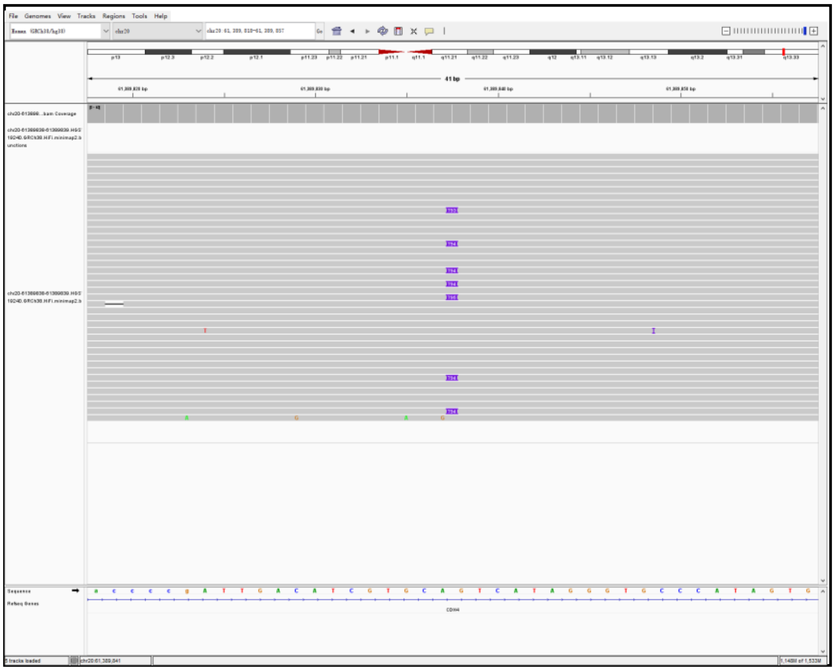

NA19238

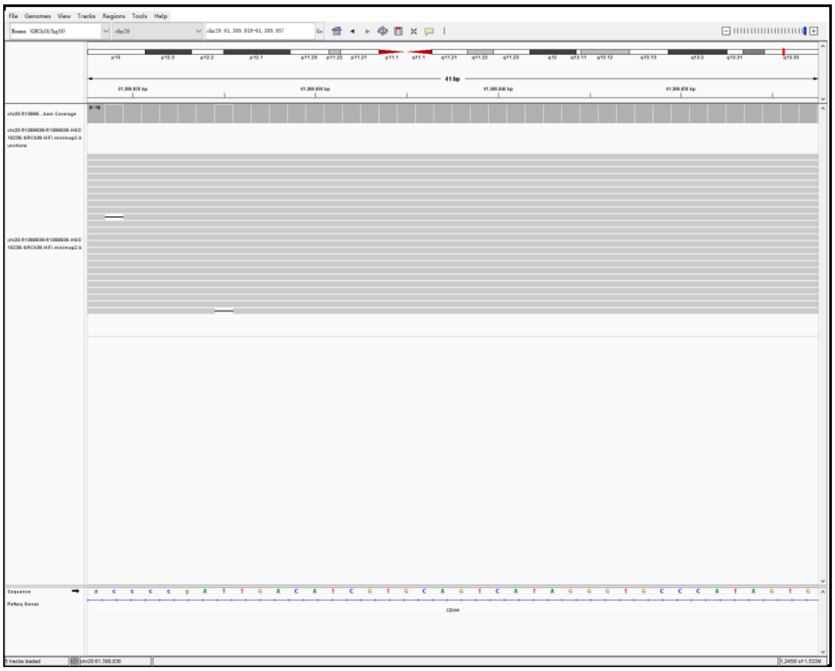

NA19239

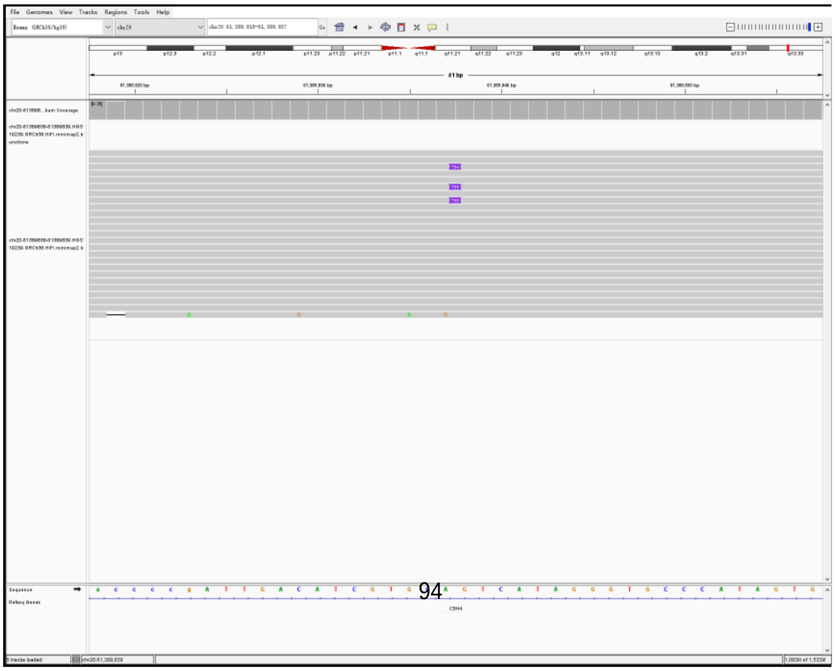

chr22 48990163 48998384 8220 DEL na19240-38-39 sniffles2

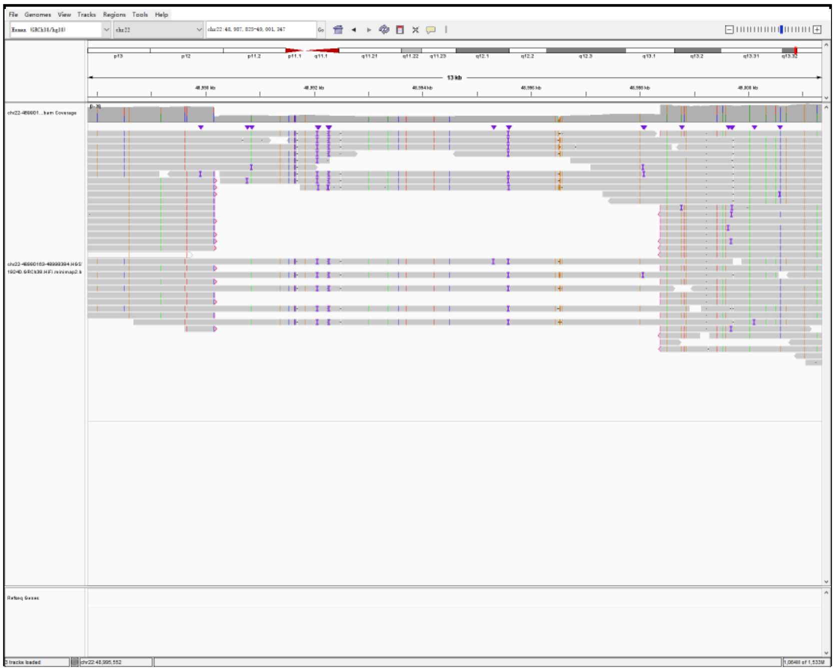

NA19240

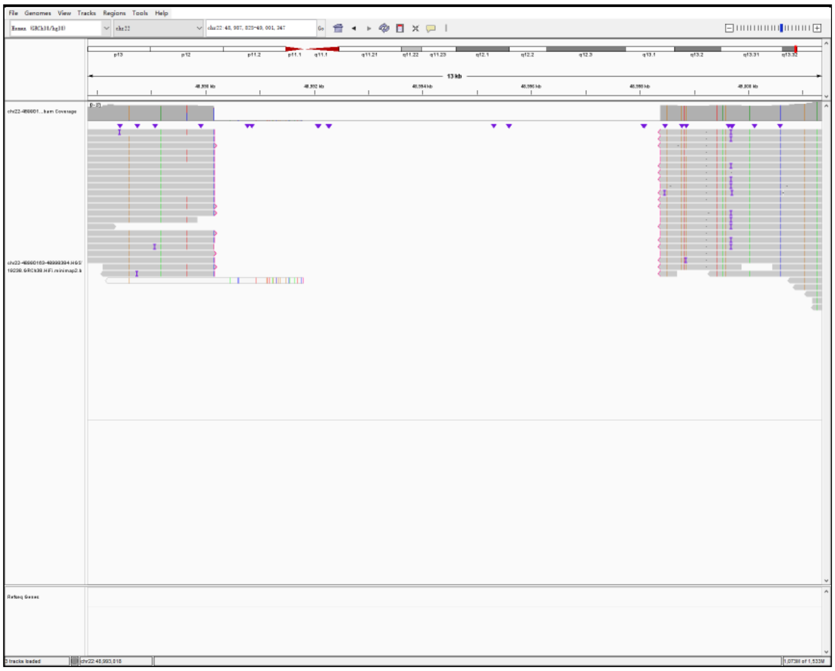

NA19238

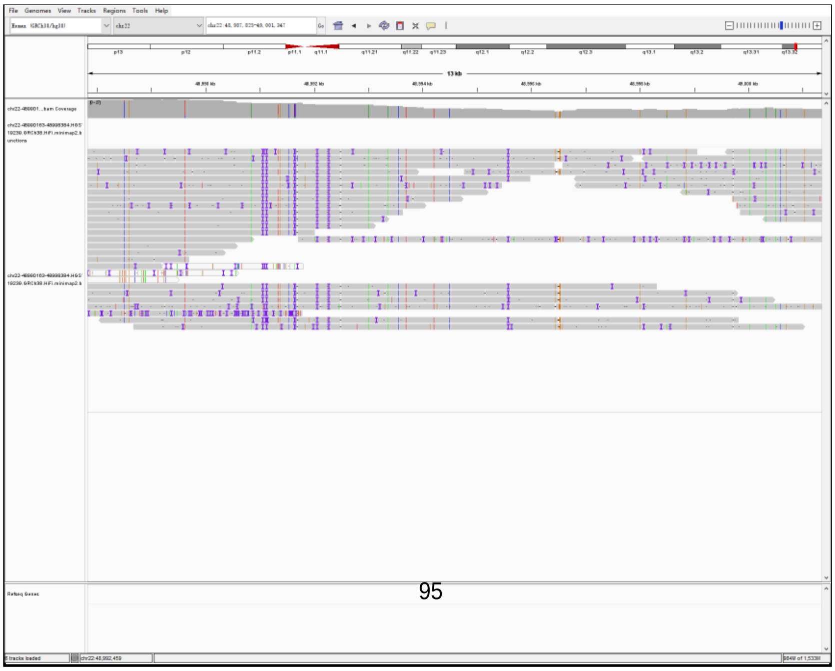

NA19239

Supplementary File 6. Experimental validation of de novo SVs in ChineseQuartet

Sanger sequencing results for ID Denovo-1

chr1-190983080-190983081-INS

- Sanger trace alignment evidences:

	5' Upstream (15bp)		Downstream (15bp) 3'
Reference:	GCATATACGTATATG	CACATATATGTGTATATACGTATATA x1	CACATACGTATATAC
SV1 sequence:	GCATATACGTATATG	CACATATATGTGTATATACGTATATA x2	CACATACGTATATAC
SV2 sequence:	GCATATACGTATATG	CACATATATGCATATACGTATATACACATA	CACATACGTATATAC
SV3 sequence:	GCATATACGTATATG	CACATATATGTGTATATACGTATATA x3	CACATACGTATATAC

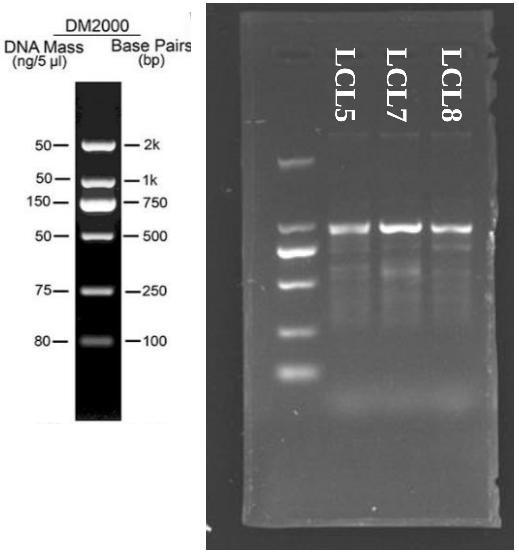

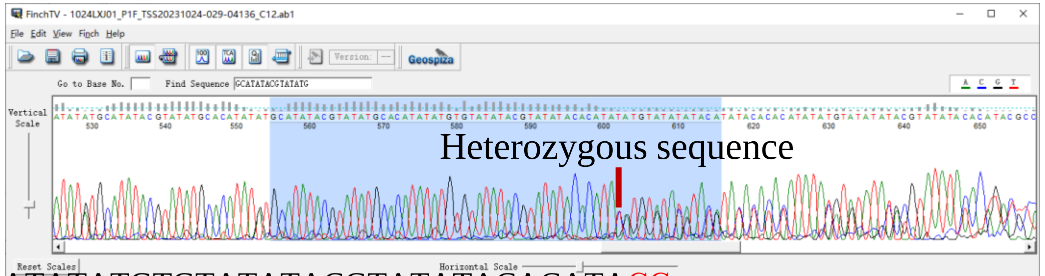

GCATATACGTATATGCACATATATGTGTATATACGTATATACACATACG…..
GCATATACGTATATGCACATATATGTGTATATACGTATATACACATATA…..

Allele 1: Reference

Allele 2: SV3

True-positive de novo SV

LCL7 (Father)

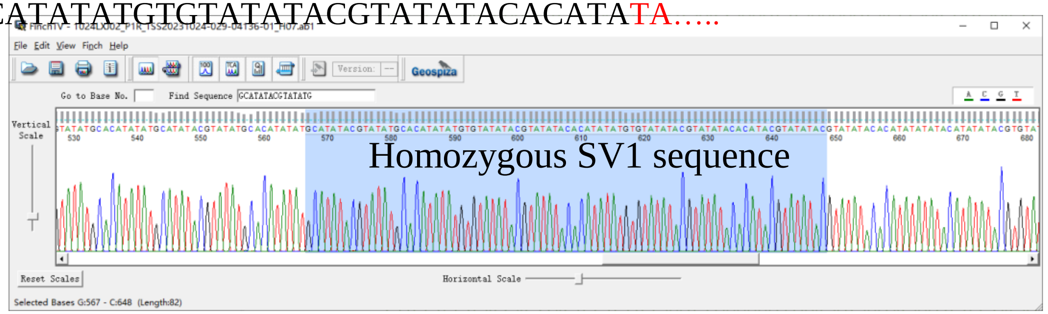

Allele 1: SV1

Allele 2: SV1

LCL8 (Mother)

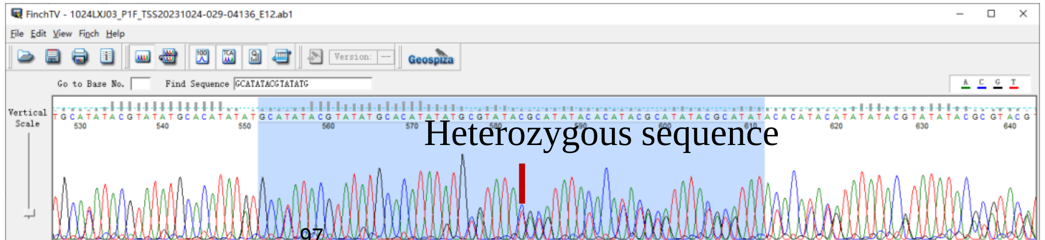

GCATATACGTATATGCACATATATGTGTATA…
GCATATACGTATATGCACATATATGCATATA…

Allele 1: Reference

Allele 2: SV2

F:AATGGTGCCCCTTGAA
R: TCAGTTTCGGATCAACTCA
Target length: 1017

Sanger sequencing results for ID Denovo-2

chr7-2492244-2492245-DEL

- Sanger trace alignment evidences:

	5' Upstream (15bp)	(88bp)	Downstream (30bp) 3'
Reference:	TCCATCCACCCACCG	ACCCACACATCAGTCCTTCTGTCCATCCATCTATTCATCCATCCATTCA CCCATCCATCCATCCATCCATCCATCCATCCATCCATCC	ACCCACACATCAGTCCTTCTGTCCAGCCAG
		(84bp, deleted one 'CCAT' repeat unit)	
SV1 sequence:	TCCATCCACCCACCG	ACCCACACATCAGTCCTTCTGTCCATCCATCTATTCATCCATCCATTCA CCCATCCATCCATCCATCCATCCATCCATCCCCAT	ACCCACACATCAGTCCTTCTGTCCAGCCAG
SV2 sequence:	TCCATCCACCCACCG		ACCCACACATCAGTCCTTCTGTCCAGCCAG

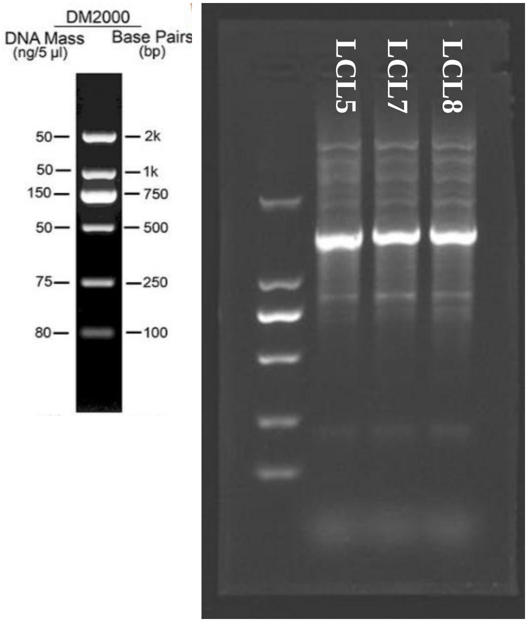

F:ATCCTCCCATCCATACACCA
R: CTGAGTTCACTCGGCAAACAT
Target length: 1438

LCL5 (Child)

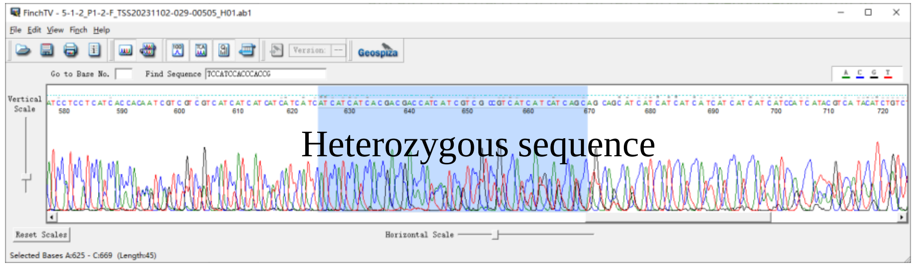

Allele 1: SV1

Allele 2: SV2

True-positive de novo SV

LCL7 (Father)

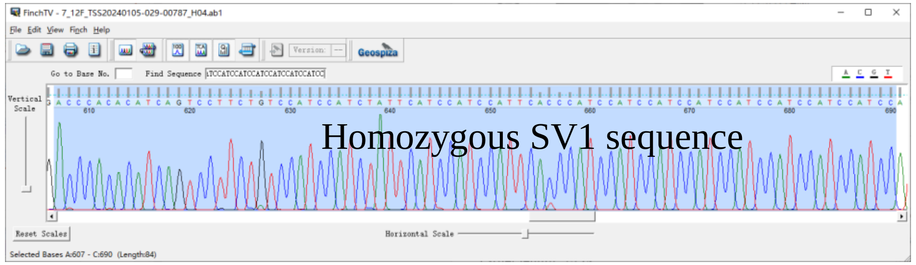

Allele 1: SV1

Allele 2: SV1

LCL8 (Mother)

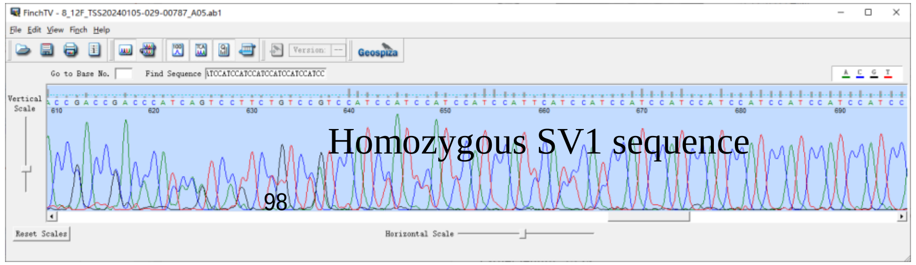

Allele 1: SV1

Allele 2: SV1

Supplementary File 7. Experimental validation of other callers' False-positive (FP) de novo SVs in ChineseQuartet

(See Supplementary Table 10 for details)

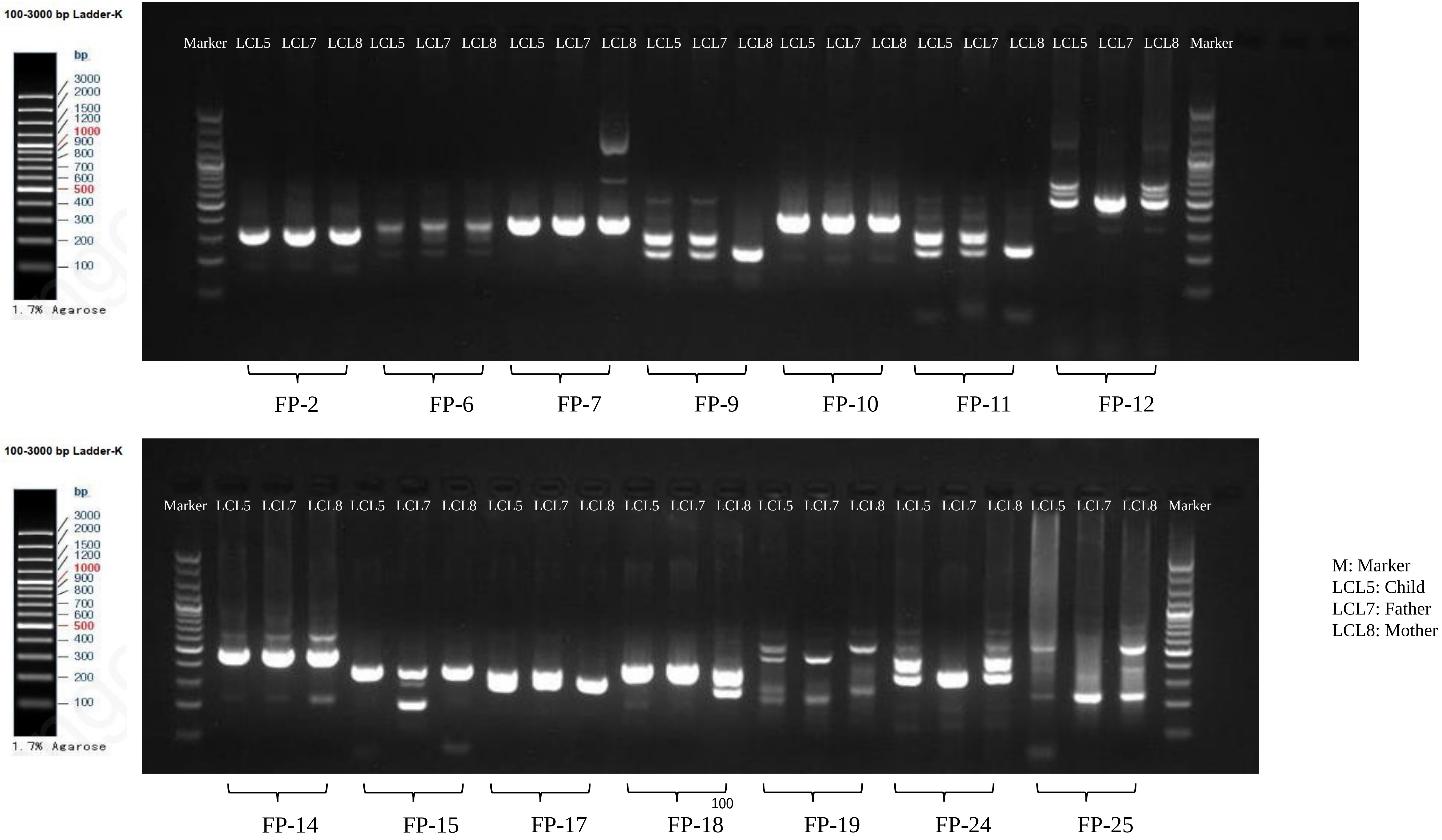
100-3000 bp Ladder-K
bp
3000
2000
1500
1200
1000
900
800
700
600
500
400
300
200
100
1.7% Agarose
Marker LCL5 LCL7 LCL8 LCL5 LCL7 LCL8 LCL5 LCL7 LCL8 LCL5 LCL7 LCL8 LCL5 LCL7 LCL8 LCL5 LCL7 LCL8 LCL5 LCL7 LCL8 Marker
FP-2
FP-6
FP-7
FP-9
FP-10
FP-11
FP-12
100-3000 bp Ladder-K
bp
3000
2000
1500
1200
1000
900
800
700
600
500
400
300
200
100
1.7% Agarose
Marker LCL5 LCL7 LCL8 LCL5 LCL7 LCL8 LCL5 LCL7 LCL8 LCL5 LCL7 LCL8 LCL5 LCL7 LCL8 LCL5 LCL7 LCL8 LCL5 LCL7 LCL8 Marker
FP-14
FP-15
FP-17
FP-18
100
FP-19
FP-24
FP-25
M: Marker
LCL5: Child
LCL7: Father
LCL8: Mother

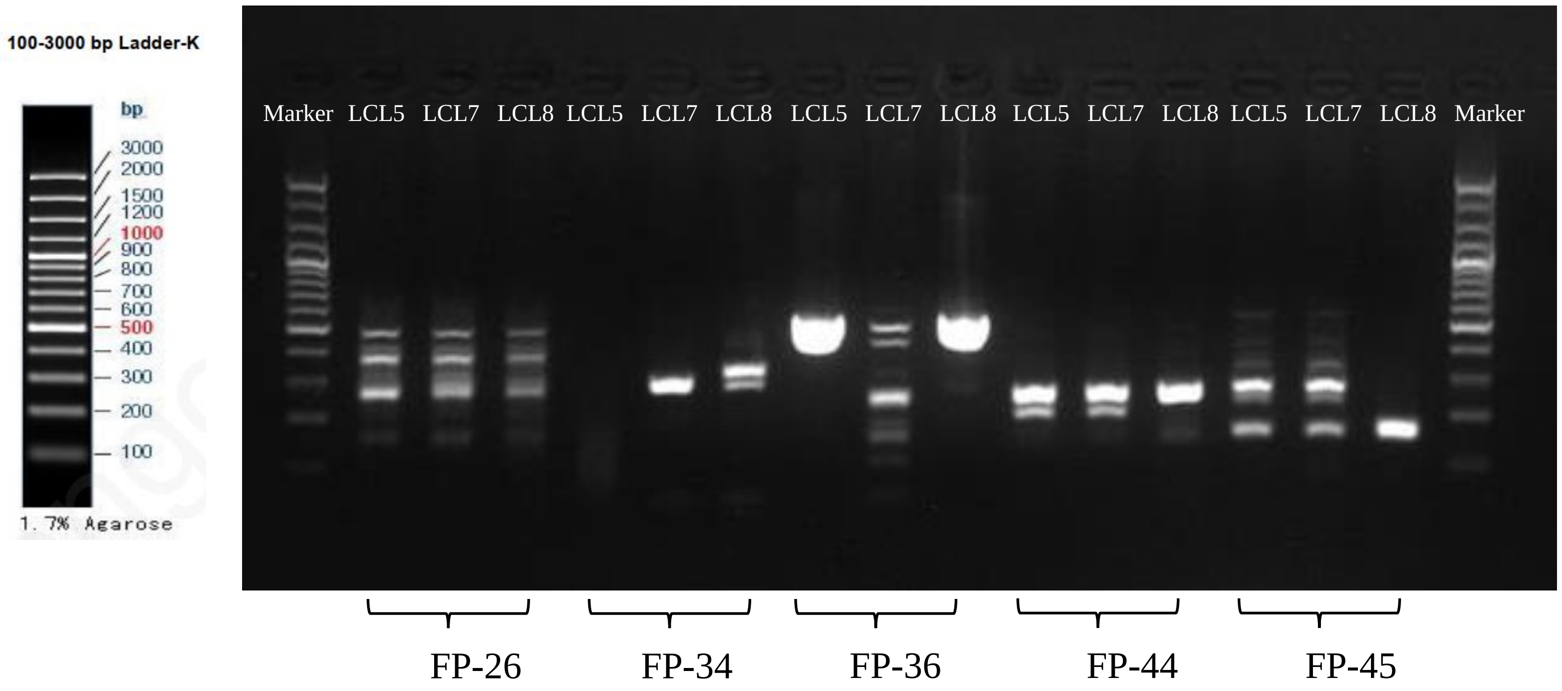

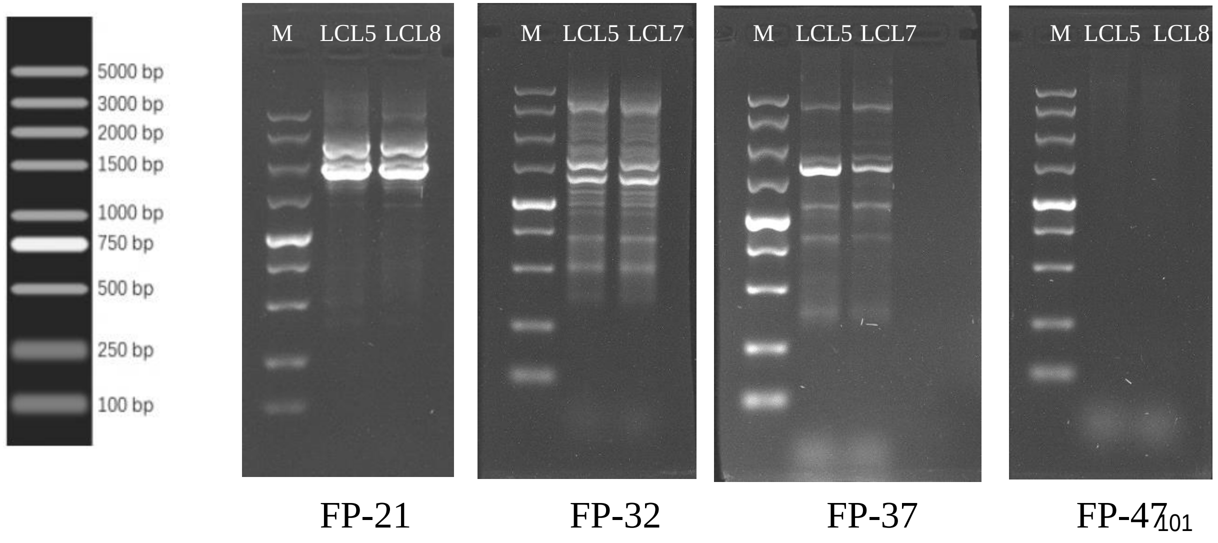

M: Marker
LCL5: Child
LCL7: Father
LCL8: Mother

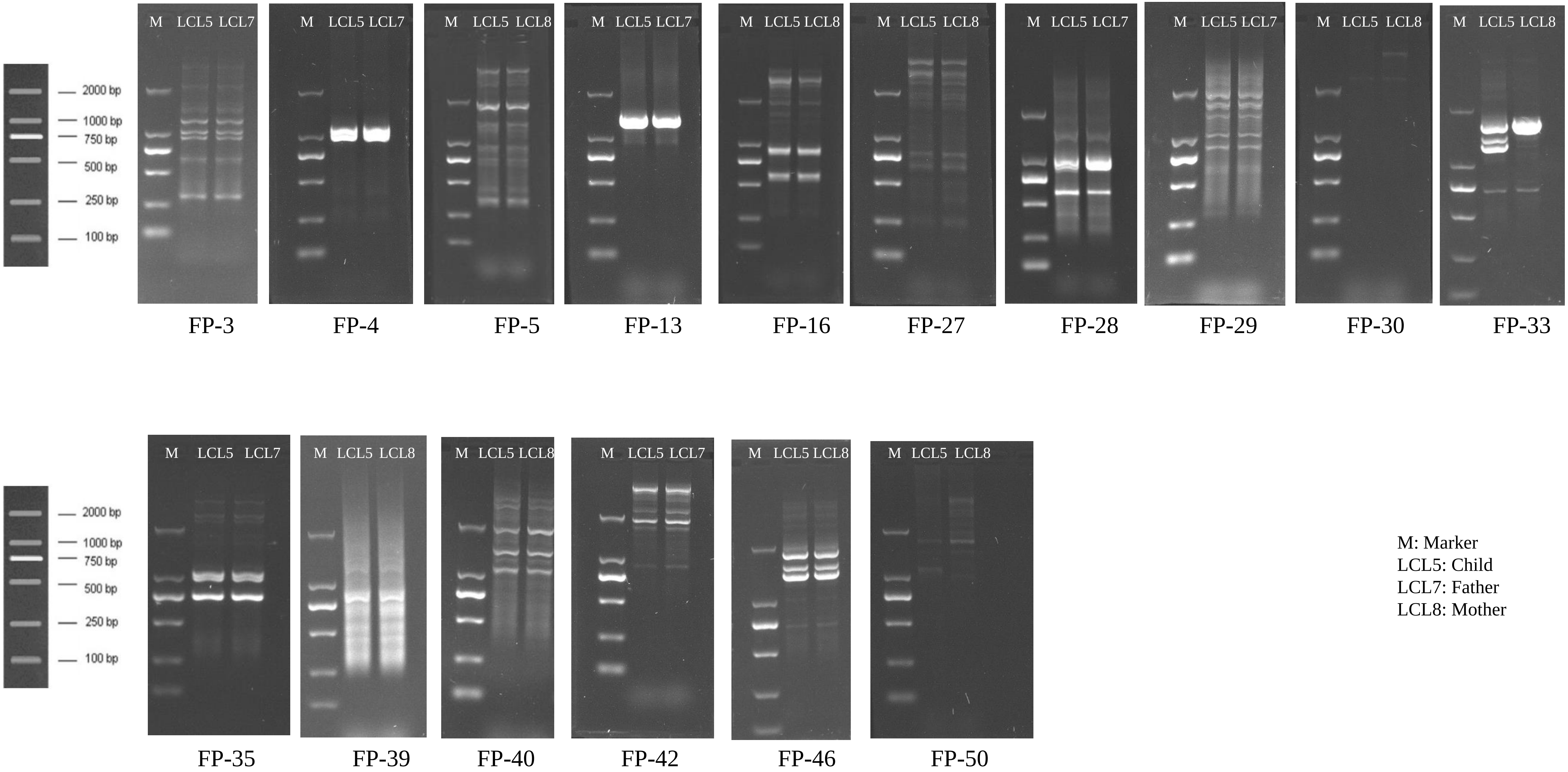
2000 bp
1000 bp
750 bp
500 bp
250 bp
100 bp
M LCL5 LCL7
FP-3
M LCL5 LCL7
FP-4
M LCL5 LCL8
FP-5
M LCL5 LCL7
FP-13
M LCL5 LCL8
FP-16
M LCL5 LCL8
FP-27
M LCL5 LCL7
FP-28
M LCL5 LCL7
FP-29
M LCL5 LCL8
FP-30
M LCL5 LCL8
FP-33
M LCL5 LCL7
FP-35
M LCL5 LCL8
FP-39
M LCL5 LCL8
FP-40
M LCL5 LCL7
FP-42
M LCL5 LCL8
FP-46
M LCL5 LCL8
FP-50
M: Marker
LCL5: Child
LCL7: Father
LCL8: Mother

Sanger sequencing results for ID FP-2 chr1-80703238-80703304-DUP

■ Sanger trace alignment evidences:

5' Upstream (15bp) ... Downstream (15bp) 3'

Reference: CAGGAAACAACAGGT ... CCATTGTGGAAGTCA

SV sequence: CAGGAAACAACAGGT GCTGGAGAGGATGTGGAGAAATAGGAA….CTGTTGGTGGGACTGTAAACTAGTTCAA CCATTGTGGAAGTCA

(SV sequence length: 95bp)

LCL5 (Child) Sanger trace: CAGGAAACAACAGGTGCTGGAGAGGATGTGGAGAAATAGGAA….CTGTTGGTGGGACTGTAAACTAGTTCAACCATTGTGGAAGTCA

(95bp)

LCL7 (Father) Sanger trace: CAGGAAACAACAGGTGCTGGAGAGGATGTGGAGAAATAGGAA….CTGTTGGTGGGACTGTAAACTAGTTCAACCATTGTGGAAGTCA

(95bp)

■ Sanger trace raw evidences: (The SV sequence in Sanger trace is marked in Blue)

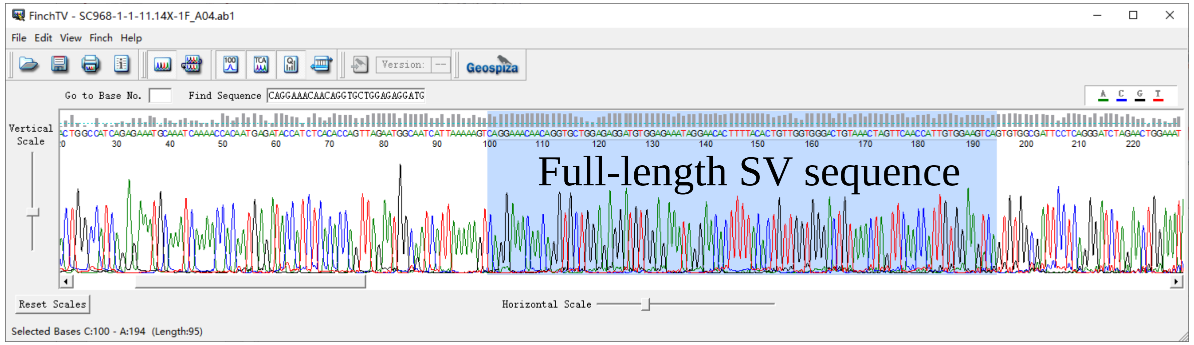

5' primer Sanger trace of LCL5 (Child)

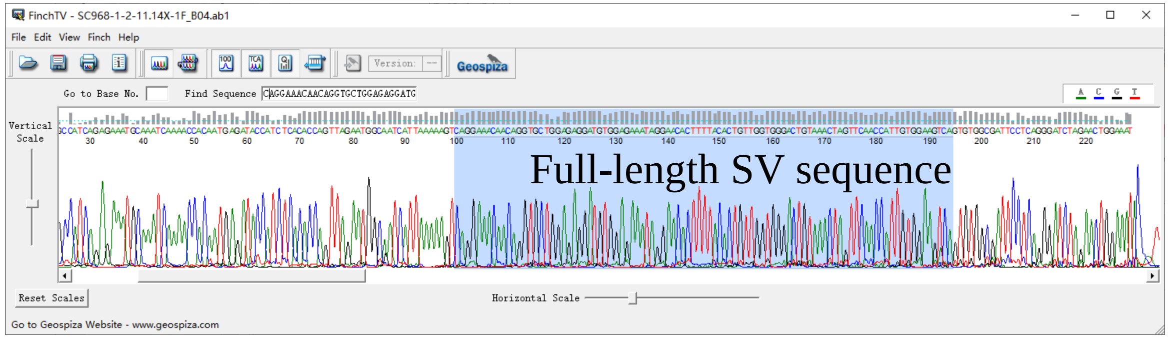

5' primer Sanger trace of LCL7 (Father)

■ Conclusion:

This SV is present in the Child and Father genomes, therefore, it is not a *de novo* SV

Sanger sequencing results for ID FP-3 chr1-104527609-104527610-INS

■ Sanger trace alignment evidences:

5’ Upstream (15bp) … Downstream (15bp) 3’

Reference: ATAATGTGTGTTTAT … AATTTATGGCTATAC

SV sequence: ATAATGTGTGTTTAT AAGAATTTATGGCGGCCGGGCGCGGT….AAAAAAAAAAAAAAAAAAAAAAAAAAAG AATTTATGGCTATAC

(SV sequence length: 353bp)

LCL5 (Child) Sanger trace: ATAATGTGTGTTTATAAGAATTTATGGC (28bp)

LCL7 (Father) Sanger trace: ATAATGTGTGTTTATAAGAATTTATGGC (28bp)

■ Sanger trace raw evidences: (The SV sequence in Sanger trace is marked in Blue)

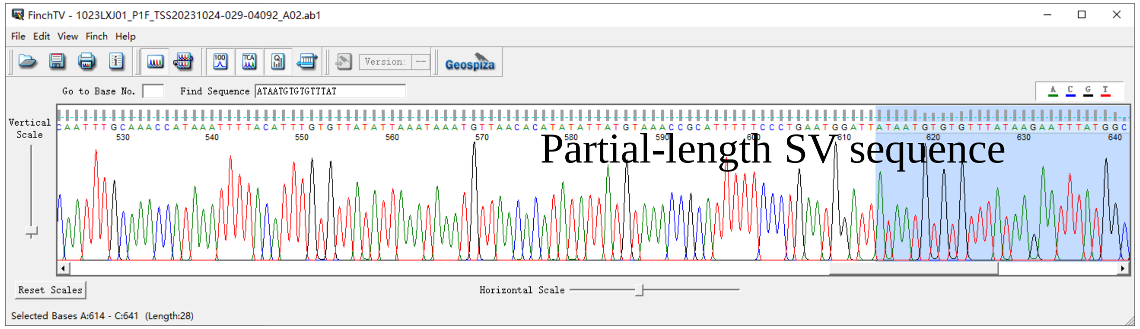

5’ primer Sanger trace of LCL5 (Child)

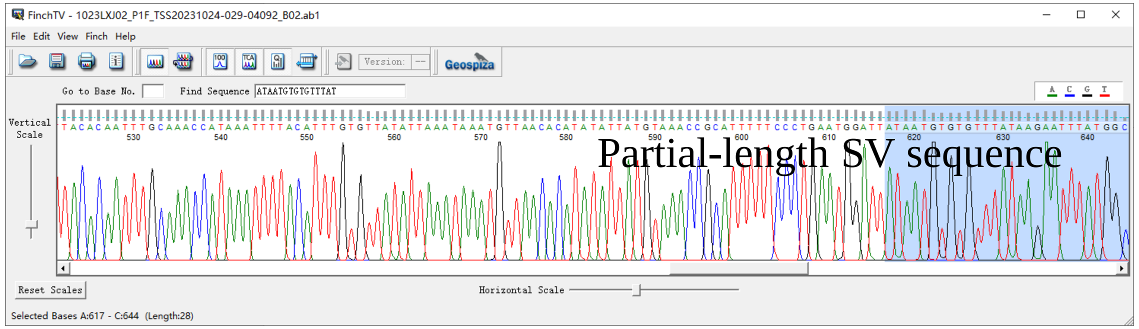

5’ primer Sanger trace of LCL7 (Father)

■ Conclusion:

This SV is present in the Child and Father genomes, therefore, it is not a *de novo* SV

Sanger sequencing results for ID FP-4 chr1-182533306-182533413-DUP

- Sanger trace alignment evidences:

5’ Upstream (15bp) Downstream (15bp) 3’

Reference: ATTATATGGATTAA TTCAACAGCTTCACT

SV sequence: ATTATATGGATTAA TTTTTTTTTTTTTTTTTGTTCAACAATGT……GCCATAAAATGTCCATTATATGGATTAA TTCAACAGCTTCACT

(SV sequence length: 138bp)

TTTTTTTTTTTTTTTTTGTTCAACAATGT……GCCATAAAATGTCCATTATATGGATTAATTCAACAGCTTCACT (123bp) LCL5 (Child) 3’Sanger trace

TTTTTTTTTTTTTTTTTGTTCAACAATGT……GCCATAAAATGTCCATTATATGGATTAATTCAACAGCTTCACT (123bp) LCL7 (Father) 3’Sanger trace

- Sanger trace raw evidences: (The SV sequence in Sanger trace is marked in Blue)

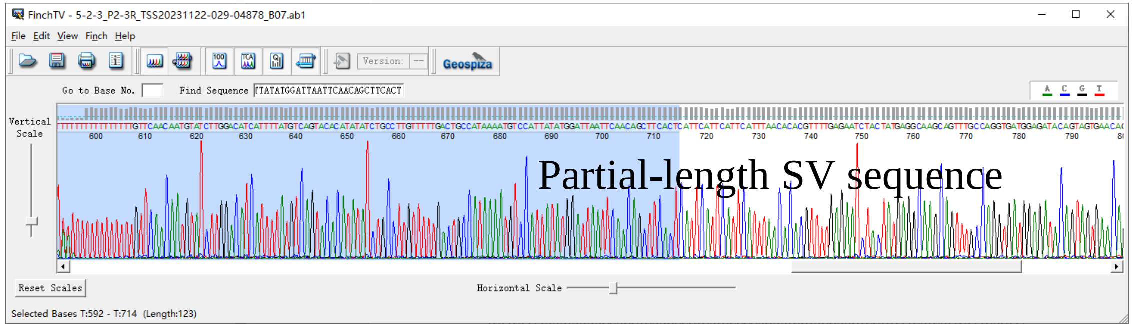

3’ primer Sanger trace of LCL5 (Child) (reverse complement sequence)

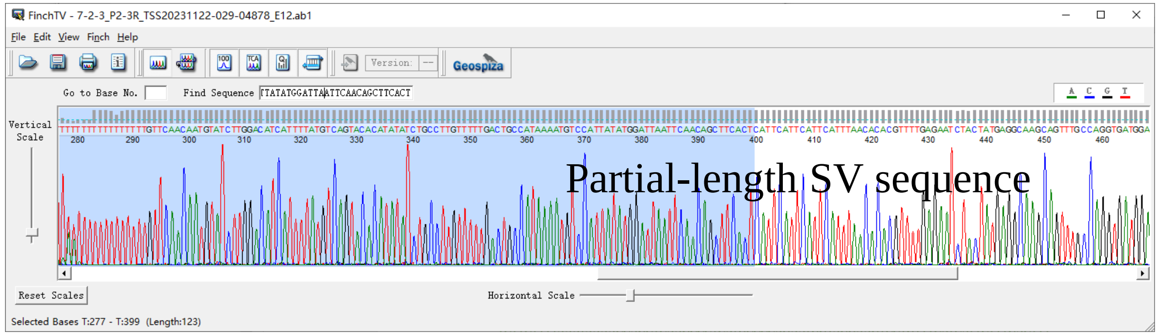

3’ primer Sanger trace of LCL7 (Father) (reverse complement sequence)

- Conclusion:

This SV is present in the Child and Father genomes, therefore, it is not a *de novo* SV

Sanger sequencing results for ID FP-7 chr1-234182900-234184003-DEL

■ Sanger trace alignment evidences:

	5' Upstream (15bp)	(1119bp)	Downstream (15bp) 3'
Reference:	GTCCATTTTCTATAC	AATCATTGGTCTTTTGTTTCTCTATTTTTCT…. TAAGAGACCTATCAGCTATAACAAA	AAAAGGAGCACTCAG
SV sequence:	GTCCATTTTCTATAC		AAAAGGAGCACTCAG

(SV sequence length: 30bp)

LCL5 (Child) Sanger trace: GTCCATTTTCTATACAAAAGGAGCACTCAG (30bp)

LCL7 (Father) Sanger trace: GTCCATTTTCTATACAAAAGGAGCACTCAG (30bp)

■ Sanger trace raw evidences: (The SV sequence in Sanger trace is marked in Blue)

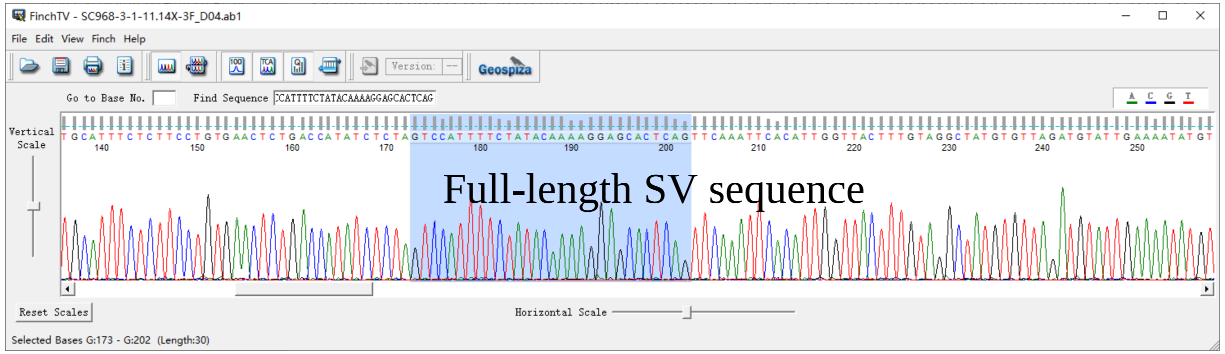

5' primer Sanger trace of LCL5 (Child)

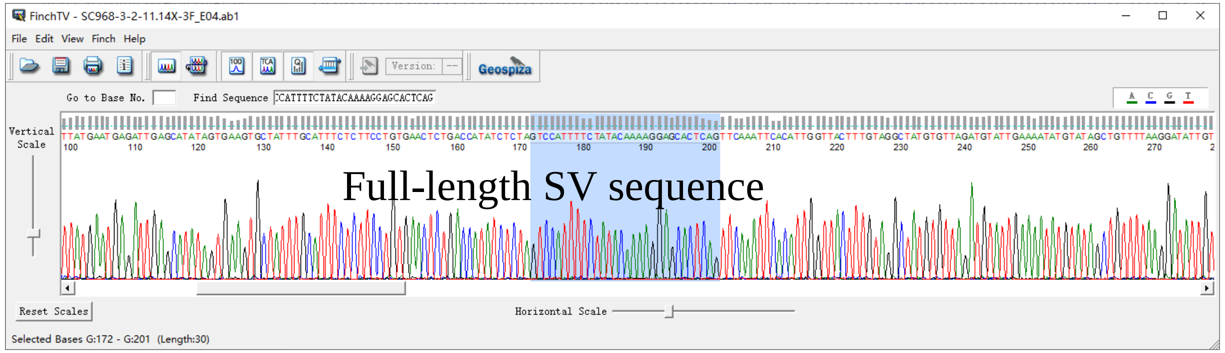

5' primer Sanger trace of LCL7 (Father)

■ Conclusion:

This SV is present in the Child and Father genomes, therefore, it is not a *de novo* SV

Sanger sequencing results for ID FP-9 chr2-118042427-118042503-DUP

■ Sanger trace alignment evidences:

5’ Upstream (15bp) Downstream (15bp) 3’

Reference: TTCCACAATTTTGCA ATTGCGAATTGTGCA

SV sequence: TTCCACAATTTTGCA ATTGCGAATTGTGCACCTATAAACAT…..TTGTATAATGACTTCTTTTCCTCTGGGTAG ATTGCGAATTGTGCA

(SV sequence length: 97bp)

LCL5 (Child) Sanger trace (97bp): TTTTGCAATTGCGAATTGTGCACCTATAAACATGTGTGTG……TTTTTTGTATAATGACTTCTTTTCCTCTGGGTAGATTGCGAATTGTGCA

LCL7 (Father) Sanger trace (97bp): TTTTGCAATTGCGAATTGTGCACCTATAAACATGTGTGTG……TTTTTTGTATAATGACTTCTTTTCCTCTGGGTAGATTGCCAATTGTGCA

■ Sanger trace raw evidences:

(The SV sequence in Sanger trace is marked in Blue)

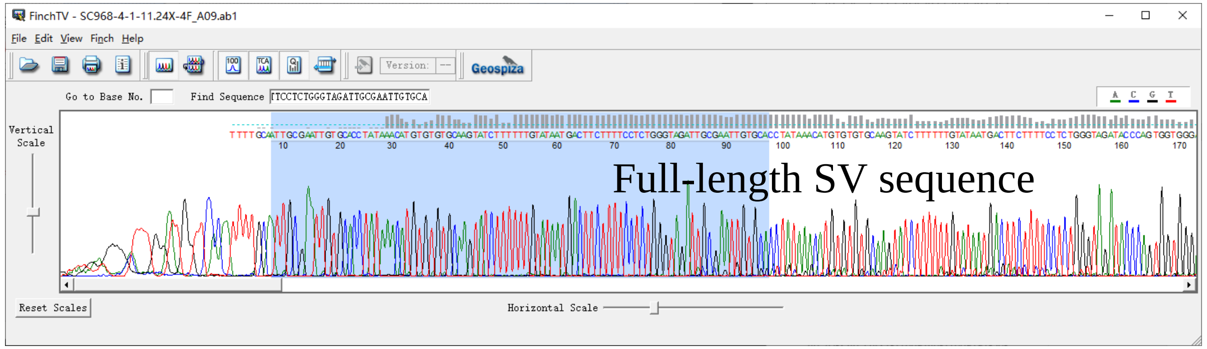

5’ primer Sanger trace of LCL5 (Child)

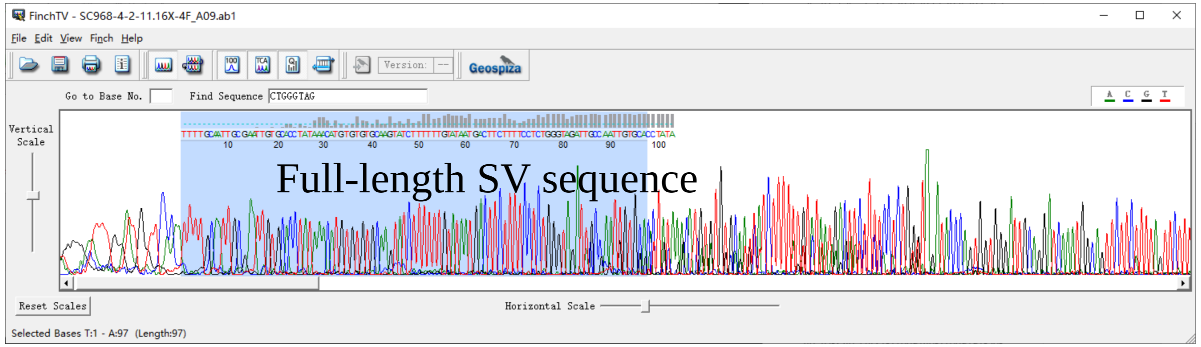

5’ primer Sanger trace of LCL7 (Father)

■ Conclusion:

This SV is present in the Child and Father genomes, therefore, it is not a *de novo* SV

Sanger sequencing results for ID FP-10 chr2-188711114-188711115-INS

Sanger trace alignment evidences:

5' Upstream (15bp) Downstream (15bp) 3'

Reference: TTTACTGCAATCTCT GAACATAAATTGTGA

SV sequence: TTTACTGCAATCTCT GAACATAAATTGTGAAGATTTCAT…….ATTTCTTATGCCTGTCTTTACTGCAATCTCT GAACATAAATTGTGA

(SV sequence length: 121bp)

LCL5 (Child) Sanger trace: TTTACTGCAATCTCTGAACATAAATTGTGAAGATTTCAT…….ATTTCTTATGCCTGTCTTTACTGCAATCTCTGAACATAAATTGTGA (121bp)

LCL7 (Father) Sanger trace: TTTACTGCAATCTCTGAACATAAATTGTGAAGATTTCAT…….ATTTCTTATGCCTGTCTTTACTGCAATCTCTGAACATAAATTGTGA (121bp)

Sanger trace raw evidences:

(The SV sequence in Sanger trace is marked in Blue)

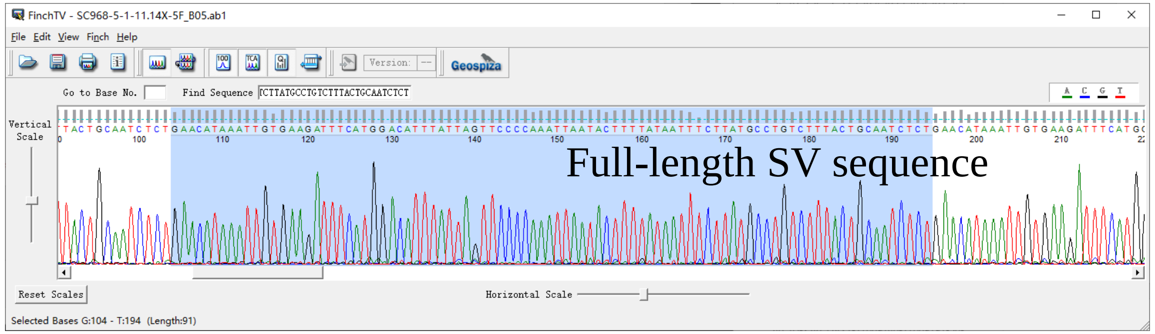

5' primer Sanger trace of LCL5 (Child)

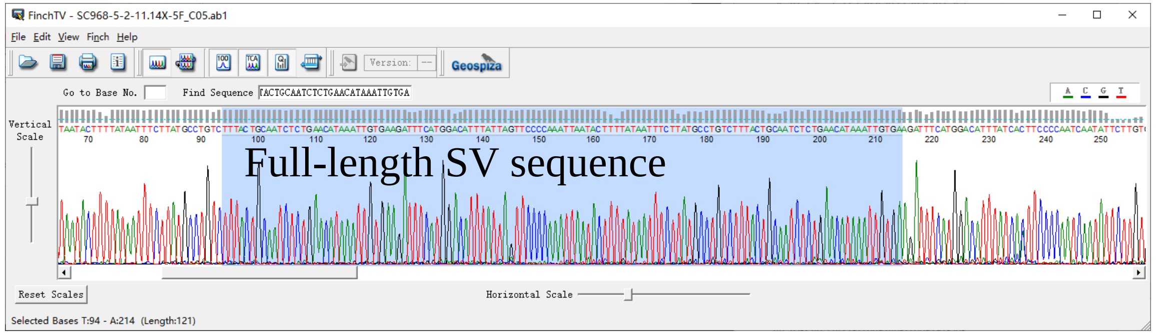

5' primer Sanger trace of LCL7 (Father)

Conclusion:

This SV is present in the Child and Father genomes, therefore, it is not a *de novo* SV

Sanger sequencing results for ID FP-11 chr3-8382823-8382904-DUP

■ Sanger trace alignment evidences:

5’ Upstream (15bp) Downstream (15bp) 3’

Reference: CTGACCCAGCACCAT AAATATAGTTGGTGA

SV sequence: CTGACCCAGCACCAT AAAGGATTCCTTTGTTGGTTCTATG…..ATGTTAAGTTTCATGCCTGACCCAGCACCAT AAATATAGTTGGTGA

(SV sequence length: 110bp)

LCL5 (Child) Sanger trace

LCL8 (Mother) Sanger trace TCTATGGGACTCTTCCTATGTTCTCAAACCATGTTAAGTTTCATGCCTGACCCAGCACCATAAATATAGTTGGTGA

(76bp)

■ Sanger trace raw evidences:

(The SV sequence in Sanger trace is marked in Blue)

NA

5’ primer Sanger trace of LCL5 (Child)

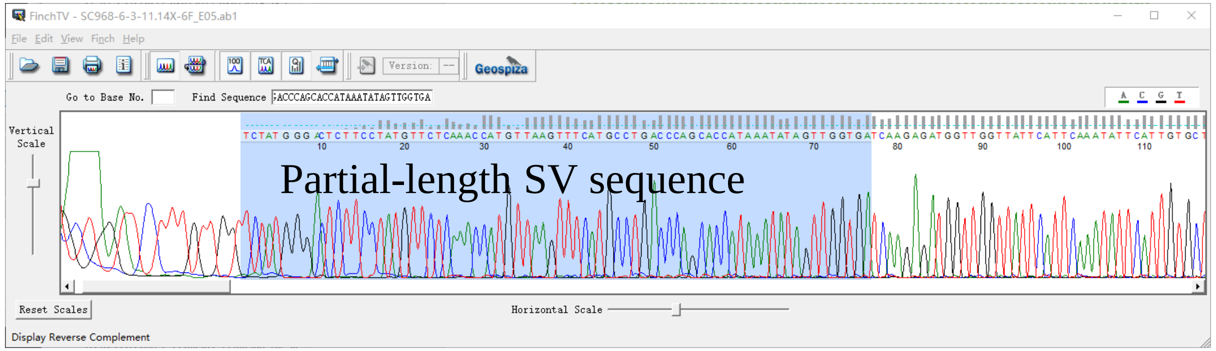

5’ primer Sanger trace of LCL8 (Mother)

■ Conclusion:

This SV is present in the Mother genomes, therefore, it is not a *de novo* SV

Sanger sequencing results for ID FP-12 chr3-160684562-160684744-DEL

■ Sanger trace alignment evidences:

5’ Upstream (15bp) (369bp) Downstream (15bp) 3’

Reference: CGCTACACATGCGCA CACACACACACACAGTTGCAATCATCCT….ACCTCCCGCTACACATGTACACACATG CACACACACACACAG

SV sequence: CGCTACACATGCGCA CACACACACACACAG

(SV sequence length: 30bp)

LCL5 (Child) Sanger trace: CGCTACACATGCGCACACACACACACACAG (30bp)

LCL7 (Father) Sanger trace: CGCTACACATGCGCACACACACACACACAG (30bp)

■ Sanger trace raw evidences: (The SV sequence in Sanger trace is marked in Blue)

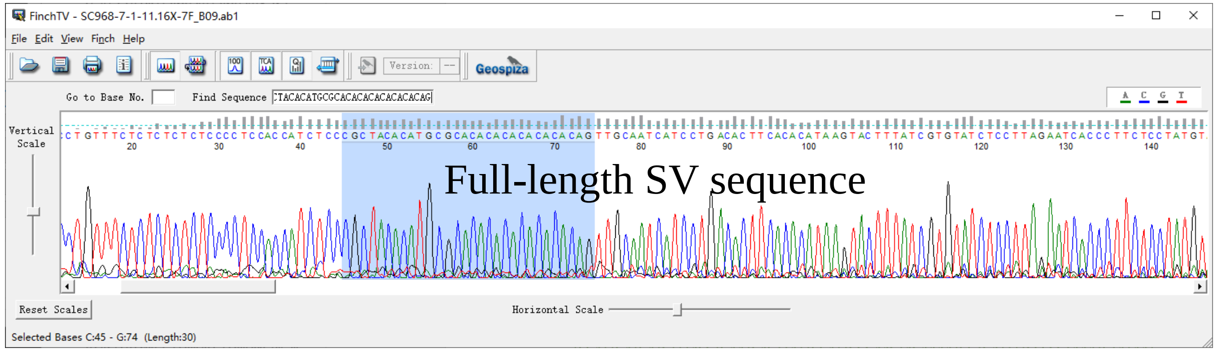

5’ primer Sanger trace of LCL5 (Child)

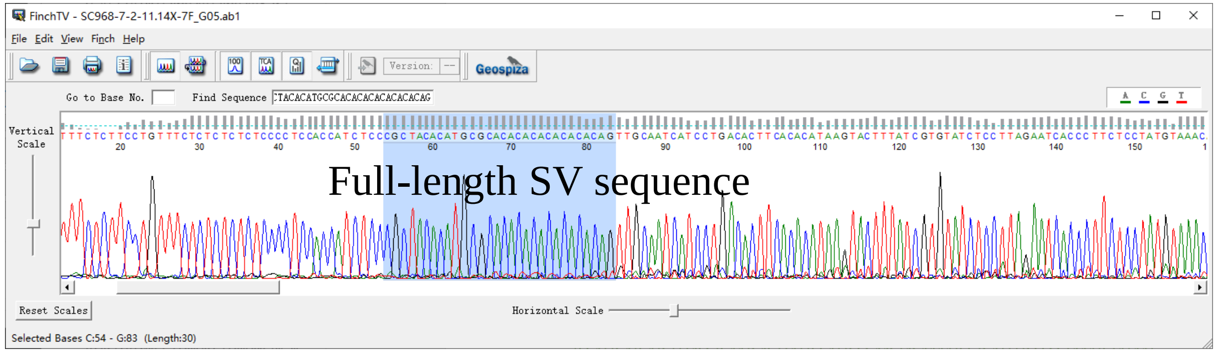

5’ primer Sanger trace of LCL7 (Father)

■ Conclusion:

This SV is present in the Child and Father genomes, therefore, it is not a *de novo* SV

Sanger sequencing results for ID FP-13 chr3-196164997-196165124-DUP

- Sanger trace alignment evidences:

5’ Upstream (15bp) Downstream (15bp) 3’

Reference: GGTGTGTGTGTGTGT GTGTGTGTGTCTGGG

SV sequence: GGTGTGTGTGTGTGT GTGTGTGTGTCTGGGGGGTTGTGTGC…..TGTGTGTATCTGTTGTGTGTTGTGTGGTAC GTGTGTGTGTCTGGG

(SV sequence length: 156bp)

GGTGTGTGTGTGTGTGTGTGTGTGTCTGGGGGGTTGTGTGC…..TGTGTGTATCTGTTGTGTGTTGTGTGGTACGTGTGTGTGTCTGGG LCL5 (Child) 3’Sanger trace (156bp)

GGTGTGTGTGTGTGTGTGTGTGTGTCTGGGGGGTTGTGTGC…..TGTGTGTATCTGTTGTGTGTTGTGTGGTACGTGTGTGTGTCTGGG LCL7 (Father) 3’Sanger trace (156bp)

- Sanger trace raw evidences: (The SV sequence in Sanger trace is marked in Blue)

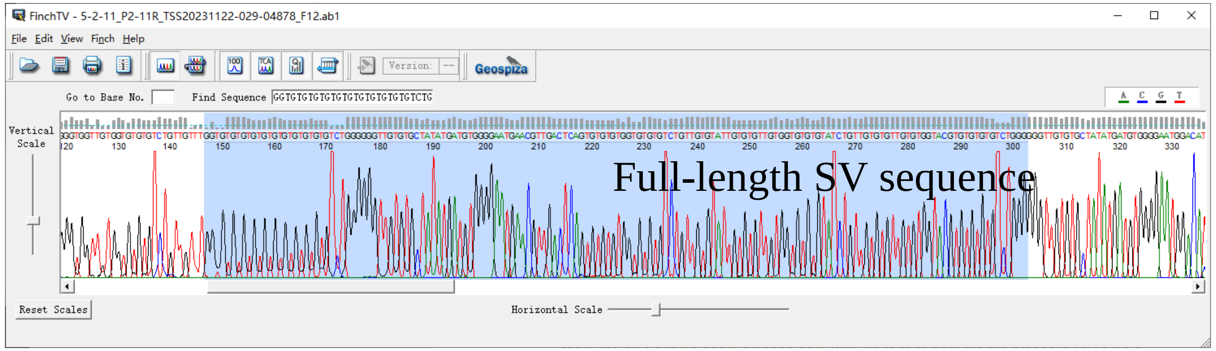

3’ primer Sanger trace of LCL5 (Child)
(reverse complement sequence)

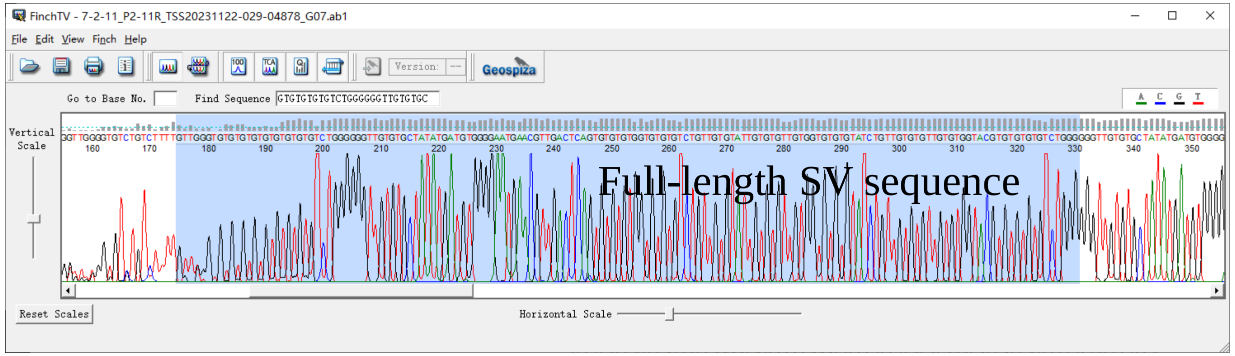

3’ primer Sanger trace of LCL7 (Father)
(reverse complement sequence)

- Conclusion:

This SV is present in the Child and Father genomes, therefore, it is not a *de novo* SV

Sanger sequencing results for ID FP-14 chr4-70419680-70419681-INS

■ Sanger trace alignment evidences:

5’ Upstream (15bp) Downstream (15bp) 3’

Reference: ACCCTTTATTTCCTT CTCCTGCCTGATTGC

SV sequence: ACCCTTTATTTCCTT AGTTCACTCATGCTTTGGCTCTCTGTTTG…..TTCCTAATTGAATACCCTTTATTTCCTT CTCCTGCCTGATTGC

(SV sequence length: 254bp)

TTGGTGTATAAGAATGCTTGTGATTTTT……TTCCTAATTGAATACCCTTTATTTCCTTCTCCTGCCTGATTGC (203bp) LCL5 (Child) 3’Sanger trace

TTGGTGTATAAGAATGCTTGTGATTTTT……TTCCTAATTGAATACCCTTTATTTCCTTCTCCTGCCTGATTGC (203bp) LCL8 (Mother) 3’Sanger trace

■ Sanger trace raw evidences: (The SV sequence in Sanger trace is marked in Blue)

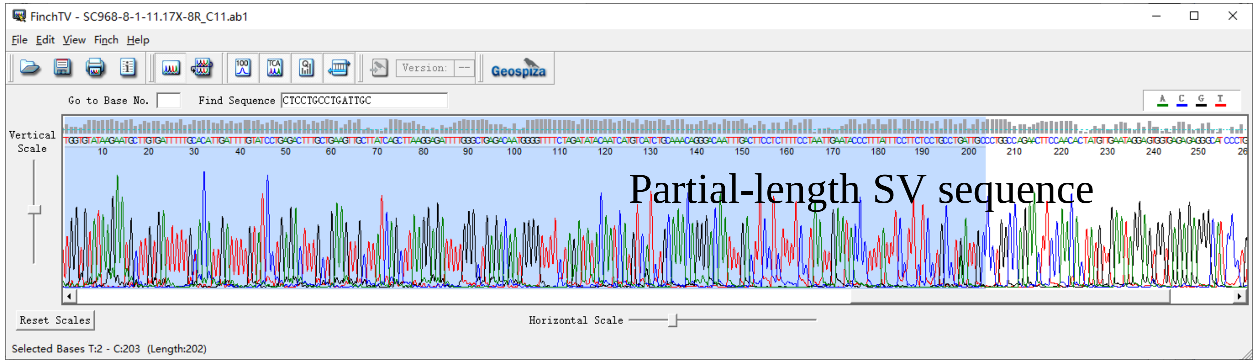

3’ primer Sanger trace of LCL5 (Child) (reverse complement sequence)

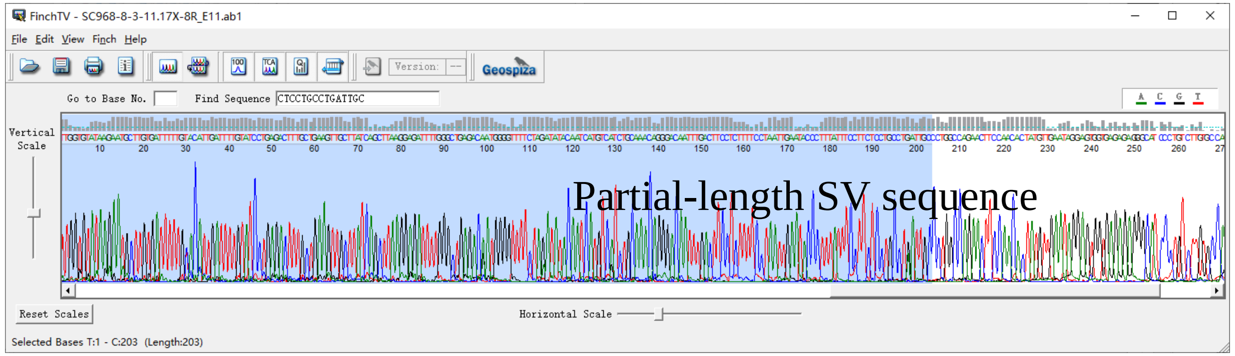

3’ primer Sanger trace of LCL8 (Mother) (reverse complement sequence)

■ Conclusion:

This SV is present in the Child and Mother genomes, therefore, it is not a *de novo* SV

Sanger sequencing results for ID FP-15 chr4-140017765-140017766-INS

Sanger trace alignment evidences:

5’ Upstream (15bp) Downstream (15bp) 3’

Reference: GAGTATCTAGTGGTT AAGAAAGGGGGATAG

SV sequence: GAGTATCTAGTGGTT AAGAAAGGGGGATAGCAGCTACTTG…..ACTCCGTCTCAAAAAAAAAAAAAAAAAAGA AAGAAAGGGGGATAG

(SV sequence length: 180bp)

LCL5 (Child) Sanger trace: GAGTATCTAGTGGTTAAGAAAGGGGGATAGCAGCTACTTG…..ACTCCGTCTCAAAAAAAAAAAAAAAAA (164bp)

LCL8 (Mother) Sanger trace: GAGTATCTAGTGGTTAAGAAAGGGGGATAGCAGCTACTTG…..ACTCCGTCTCAAAAAAAAAAAAAAAAA (164bp)

Sanger trace raw evidences:

(The SV sequence in Sanger trace is marked in Blue)

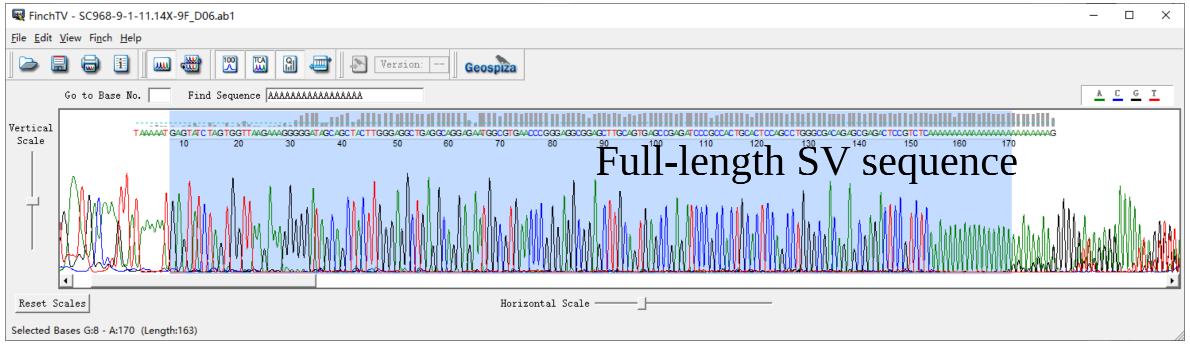

5’ primer Sanger trace of LCL5 (Child)

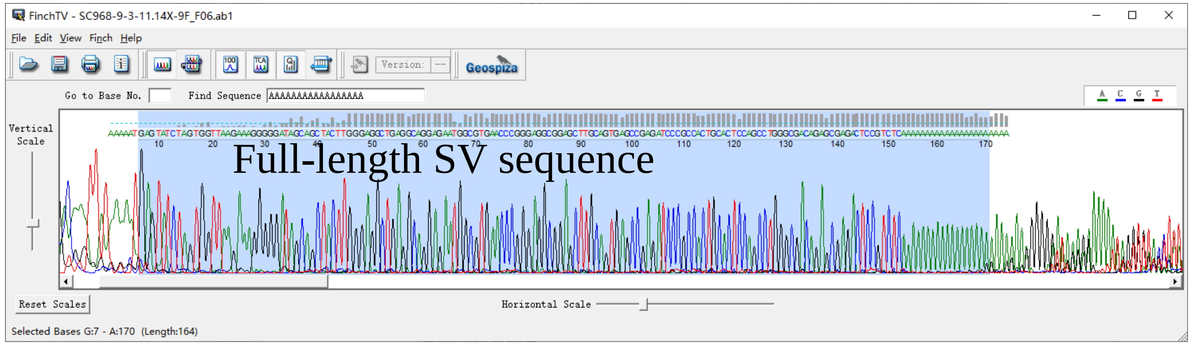

5’ primer Sanger trace of LCL8 (Mother)

Conclusion:

This SV is present in the Child and Mother genomes, therefore, it is not a *de novo* SV

Sanger sequencing results for ID FP-16   chr4-179485069-179485070-INS

■ Sanger trace alignment evidences:

5’ Upstream (15bp) Downstream (15bp) 3’

Reference: AACACACAGACACAC ATATATATAAACTGA

SV sequence: AACACACAGACACAC TATATATATTATATGATATATATATATATATATATATATATATATATATATATATAT ATATATATAAACTGA

(SV sequence length: 87bp)

LCL5 (Child) Sanger trace (79bp): AACACACAGACACACTATATATATTATATGATATATATATATATATATATATATATATATATATATATATATATATATATA

LCL8 (Mother) Sanger trace (72bp): AACACACAGACACACTATATATATTATATGATATATATATATATATATATATATATATATATATATATATATA

■ Sanger trace raw evidences:

(The SV sequence in Sanger trace is marked in Blue)

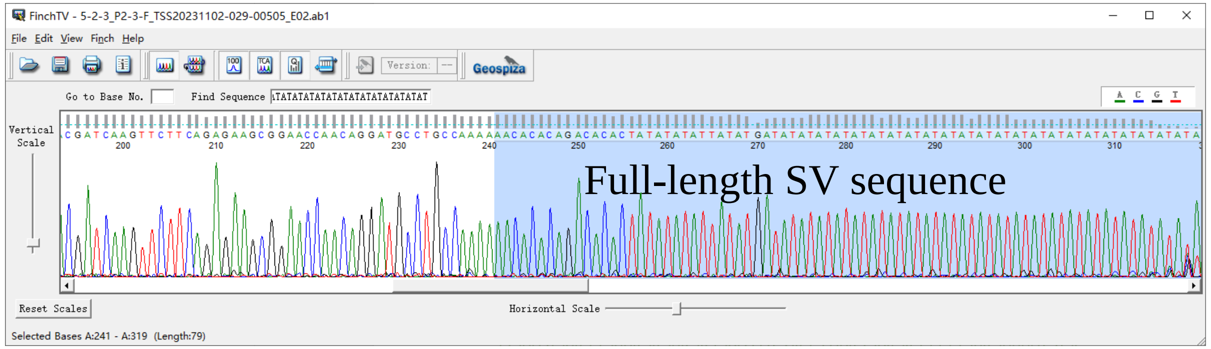

5’ primer Sanger trace of LCL5 (Child)

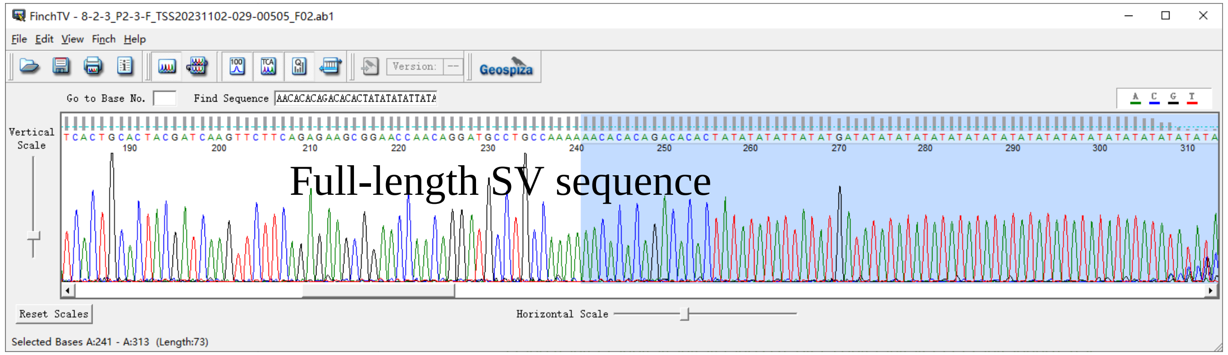

5’ primer Sanger trace of LCL8 (Mother)

■ Conclusion:

This SV is present in the Child and Mother genomes, therefore, it is not a *de novo* SV

Sanger sequencing results for ID FP-17 chr5-73775880-73775931-DEL

■ Sanger trace alignment evidences:

5’ Upstream (15bp) (50bp) Downstream (15bp) 3’

Reference: AAGTGTATATTCAAG TCTTGAATATACATGACTCTCCAAAAACACATAATTTGAATCAACACAA AATTTAGGTTGGAAA

SV sequence: AAGTGTATATTCAAG AATTTAGGTTGGAAA

(SV sequence length: 30bp)

LCL5 (Child) Sanger trace: AAGTGTATATTCAAGAATTTAGGTTGGAAA (30bp)

LCL8 (Mother) Sanger trace: AAGTGTATATTCAAGAATTTAGGTTGGAAA (30bp)

■ Sanger trace raw evidences:

(The SV sequence in Sanger trace is marked in Blue)

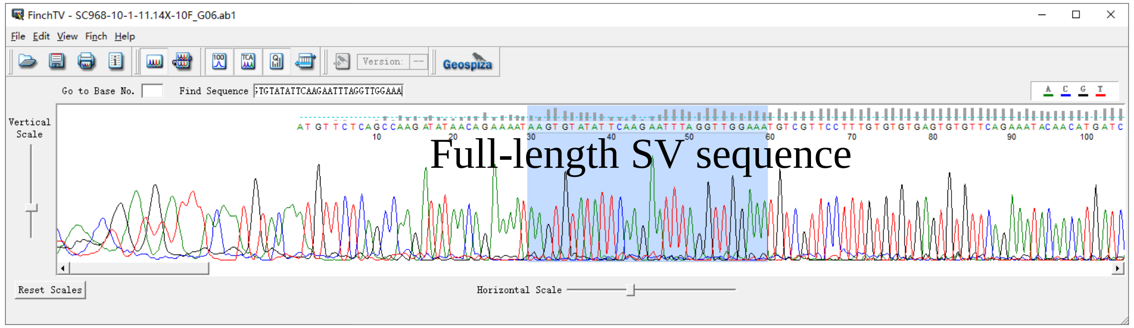

5’ primer Sanger trace of LCL5 (Child)

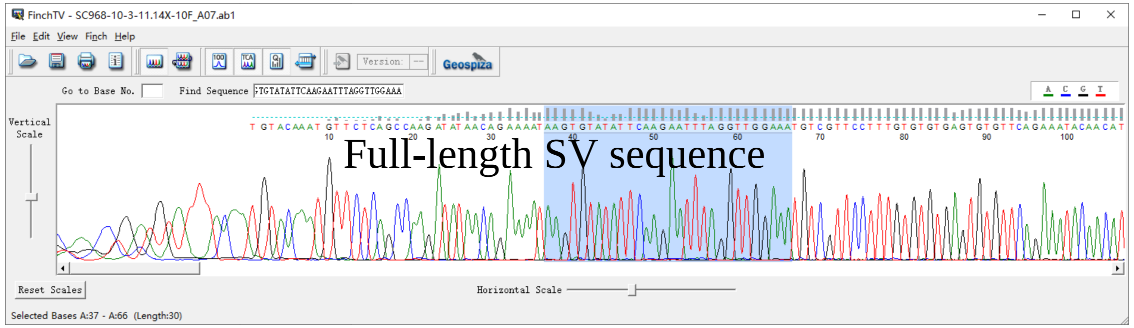

5’ primer Sanger trace of LCL8 (Mother)

■ Conclusion:

This SV is present in the Child and Mother genomes, therefore, it is not a *de novo* SV

Sanger sequencing results for ID FP-18 chr5-96896874-96896975-DUP

- Sanger trace alignment evidences:

5’ Upstream (15bp) Downstream (15bp) 3’

Reference: TGTCAGTCAACCATA TTTATTCTGCTTGCT

SV sequence: TGTCAGTCAACCATA TAAGTCATATGTTGGGTAACGATAGA……AAATAGCTATATATTGTCAGTCAACCATA TTTATTCTGCTTGCT

(SV sequence length: 130bp)

LCL5 (Child) Sanger trace: TGTCAGTCAACCATATAAGTCATATGTTGGGTAACGATAGA……AAATAGCTATATATTGTCAGTCAACCATATTTATTCTGCTTGCT (130bp)

LCL7 (Father) Sanger trace: TGTCAGTCAACCATATAAGTCATATGTTGGGTAACGATAGA……AAATAGCTATATATTGTCAGTCAACCATATTTATTCTGCTTGCT (130bp)

- Sanger trace raw evidences: (The SV sequence in Sanger trace is marked in Blue)

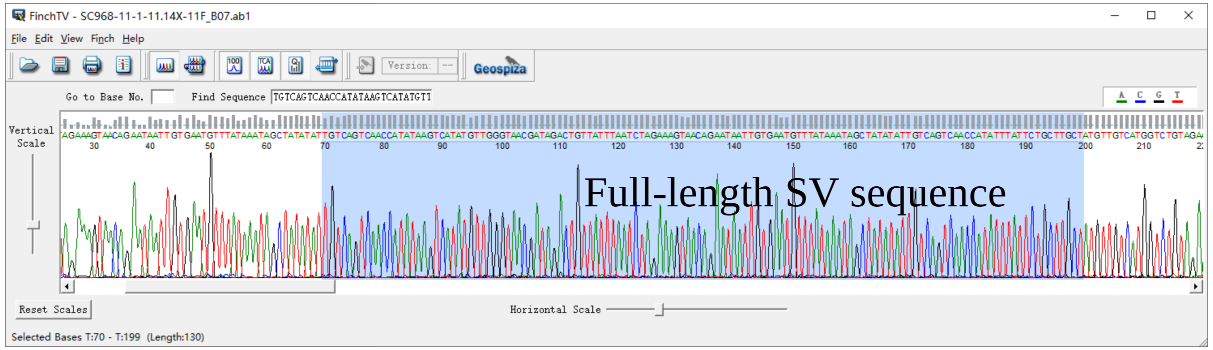

5’ primer Sanger trace of LCL5 (Child)

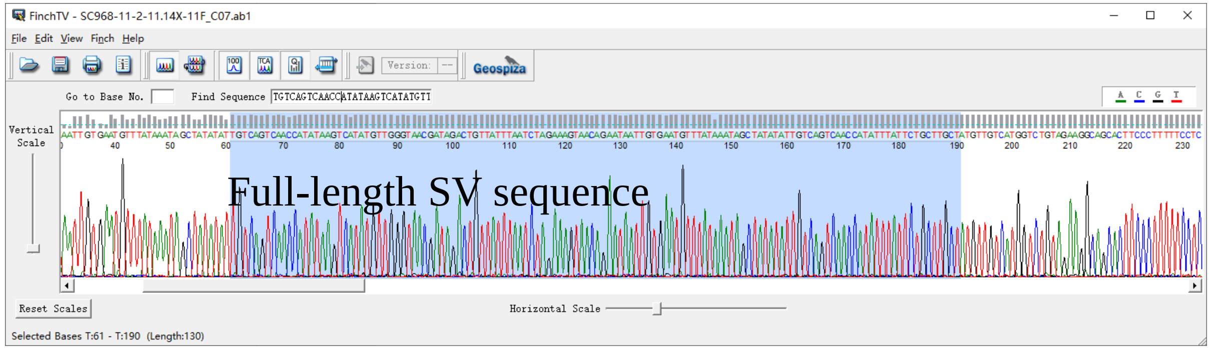

5’ primer Sanger trace of LCL7 (Father)

- Conclusion:

This SV is present in the Child and Father genomes, therefore, it is not a *de novo* SV

Sanger sequencing results for ID FP-19 chr5-131849355-131849356-INS

■ Sanger trace alignment evidences:

5’ Upstream (15bp) Downstream (15bp) 3’

Reference: TTATATATATAAAGA AACCTCTTTTCTTTA

SV sequence: TTATATATATAAAGA AACCTCTTTTCTTTATATATATAAAGAAACCTCTTTTCTTTATATATATAAAGA AACCTCTTTTCTTTA

(SV sequence length: 84bp)

LCL5 (Child) Sanger trace

LCL8 (Mother) Sanger trace TTATATATATAAAGAAACCTCTTTTCTTTATATATATAAAGAAACCTCTTTTCTTTATATATATAAAGAAACCTCTTTTCTTTA (84bp)

■ Sanger trace raw evidences: (The SV sequence in Sanger trace is marked in Blue)

NA

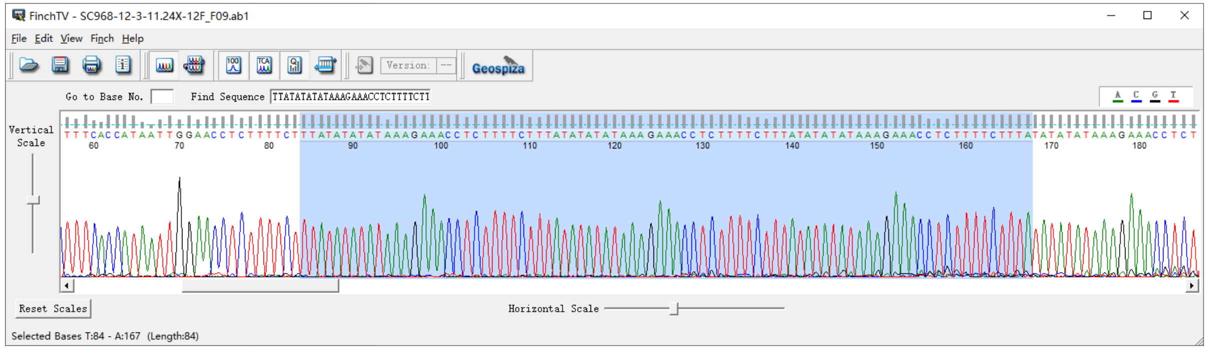

5’ primer Sanger trace of LCL5 (Child) 5’ primer Sanger trace of LCL8 (Mother)

■ Conclusion:

This SV is present in Mother genomes, therefore, it is not a *de novo* SV

Sanger sequencing results for ID FP-21 chr6-149816245-149816245-INS

■ Sanger trace alignment evidences:

5' Upstream (15bp) Downstream (15bp) 3'

Reference: TGGTTCCCCACTTCC GGGGTTTCACTATGT

SV sequence: TGGTTCCCCACTTCC GGCTTCATCTTTCTGGCTTTTTTTTTT…CACGCCCAGCTAATTTTTTTTAGTAGCGAC GGGGTTTCACTATGT

(SV sequence length: 1767bp)

LCL5 (Child) Sanger trace

LCL7 (Father) Sanger trace TGGTTCCCCACTTCCGGCTTCATCTTTCTGGCTTTTTTTTTT (41bp)

■ Sanger trace raw evidences:

(The SV sequence in Sanger trace is marked in Blue)

NA

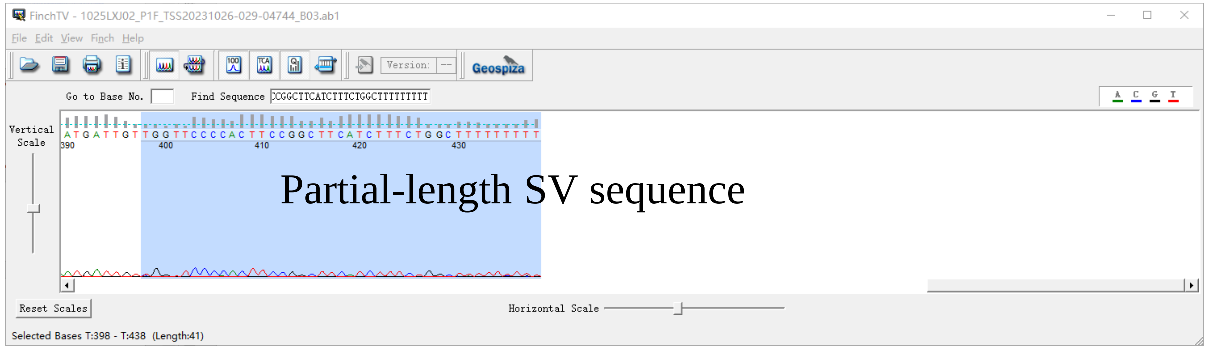

5' primer Sanger trace of LCL5 (Child)

5' primer Sanger trace of LCL7 (Father)

■ Conclusion:

This SV is present in the Father genomes, therefore, it is not a *de novo* SV

Sanger sequencing results for ID FP-24 chr8-134867673-134867674-INS

■ Sanger trace alignment evidences:

5’ Upstream (15bp) Downstream (15bp) 3’

Reference: GCCCAGAGAAGACAT CCACAACCCTTGCTT

SV sequence: GCCCAGAGAAGACAT CCAGTGCTTAGTCCAACTTCACATCTG….ACCCCAAATGCAGGGGCTCACTCCCTCCA CCACAACCCTTGCTT

(SV sequence length: 144bp)

LCL5 (Child) Sanger trace (144bp): GCCCAGAGAAGACATCCAGTGCTTAGTCCAACTTCACATCTG…..ACCCCAAATGCAGGGGCTCACTCCCTCCACCACAACCCTTGCTT

LCL8 (Mother) Sanger trace (144bp): GCCCAGAGAAGACATCCAGTGCTTAGTCCAACTTCACATCTG…..ACCCCAAATGCAGGGGCTCACTCCCTCCACCACAACCCTTGCTT

■ Sanger trace raw evidences:

(The SV sequence in Sanger trace is marked in Blue)

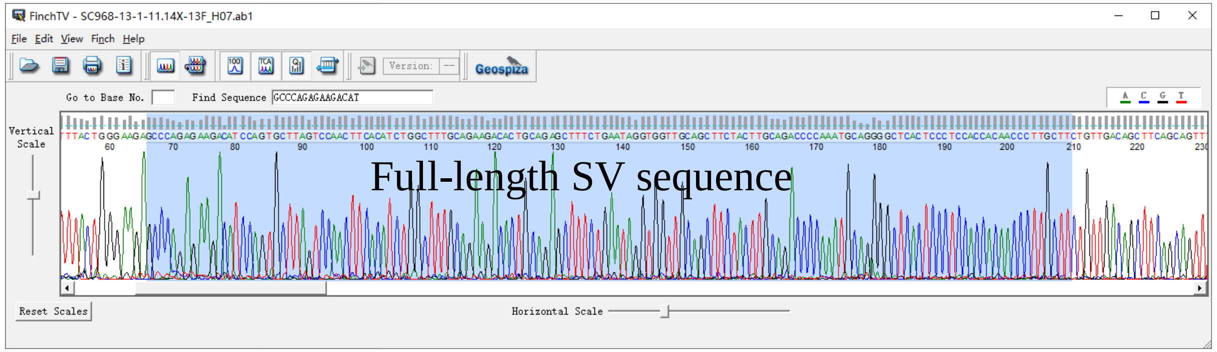

5’ primer Sanger trace of LCL5 (Child)

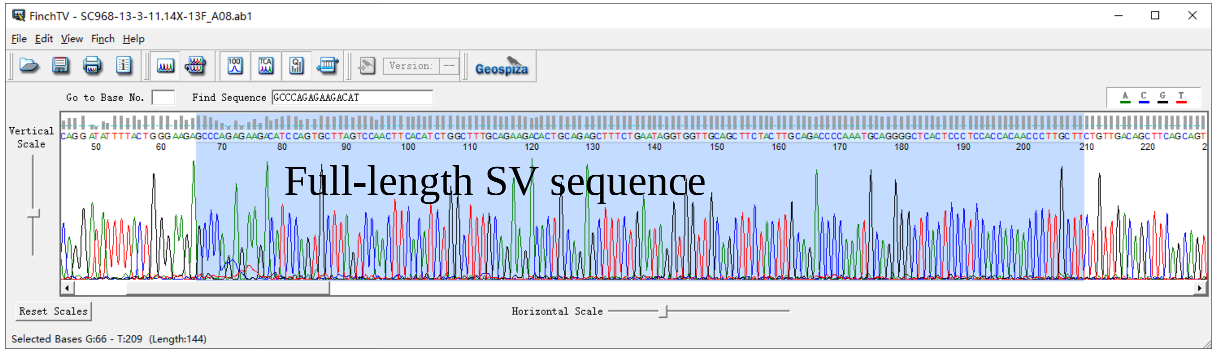

5’ primer Sanger trace of LCL8 (Mother)

■ Conclusion:

This SV is present in the Child and Mother genomes, therefore, it is not a *de novo* SV

Sanger sequencing results for ID FP-28 chr11-55264125-55271067-DEL

■ Sanger trace alignment evidences:

5' Upstream (15bp) (7040bp) Downstream (15bp) 3'

Reference: CACCATGGAAATATA ATTACACCTCTTTAAGTTTAACAAAAAT……TTATGTCTTGAATTGCATTCTAATGTT ATTAAAACTCATTTA

SV sequence: CACCATGGAAATATA ATTAAAACTCATTTA

(SV sequence length: 30bp)

LCL5 (Child) Sanger trace: CACCATGGAAATATAATTAAAACTCATTTA (30bp)

LCL7 (Father) Sanger trace: CACCATGGAAATATAATTAAAACTCATTTA (30bp)

■ Sanger trace raw evidences: (The SV sequence in Sanger trace is marked in Blue)

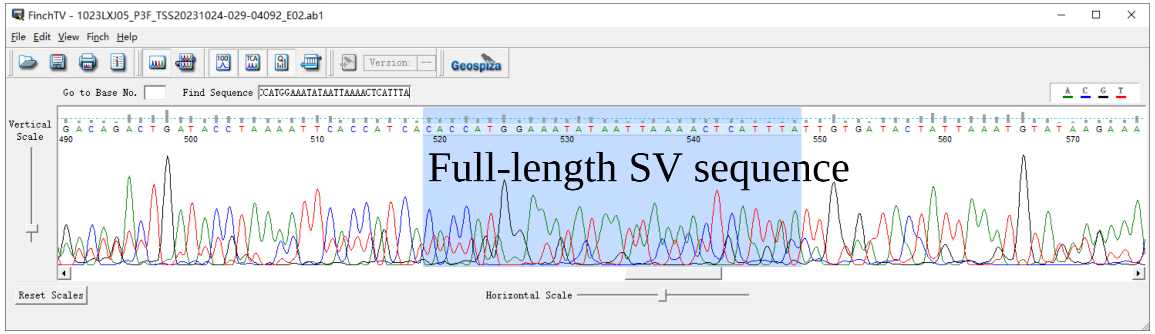

5' primer Sanger trace of LCL5 (Child)

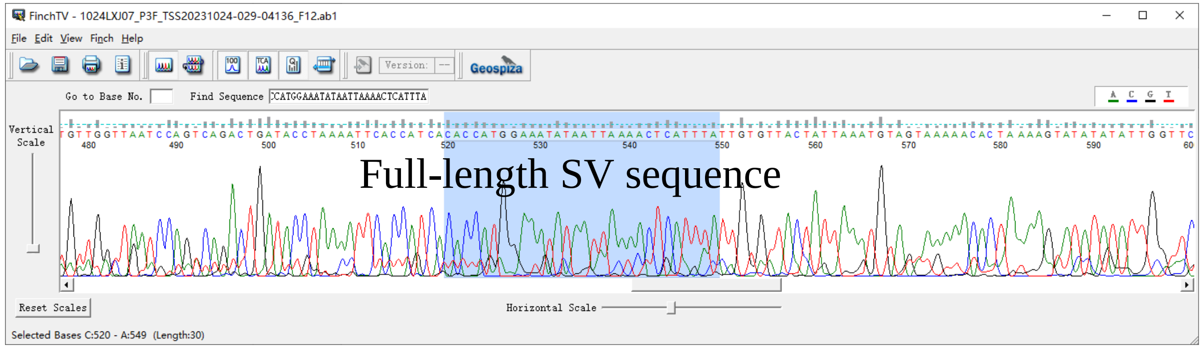

5' primer Sanger trace of LCL7 (Father)

■ Conclusion:

This SV is present in the Child and Father genomes, therefore, it is not a *de novo* SV

Sanger sequencing results for ID FP-33 chr12-47941205-47941206-INS

■ Sanger trace alignment evidences:

5’ Upstream (15bp) Downstream (15bp) 3’

Reference: TAAGAGAGGATTTCA AACAGAAGTGTTCAA

SV sequence: TAAGAGAGGATTTCA TGGATGCATTAAGTCGAAATGTCCGCGAG…...AAGTCGAAATGTCCGCGAGGCCTCC AACAGAAGTGTTCAA

(SV sequence length: 847bp)

LCL5 (Child) Sanger trace: ATTTCATGGATGCATTAAG……ATCTGGTGGACTGGACG (347bp)

LCL8 (Mother) Sanger trace: GAGGATTTCATGGATGCATTAAGTCGAAATG…..AAGATGAAGGTGAGGCAGGCTAGATT (367bp)

■ Sanger trace raw evidences: (The SV sequence in Sanger trace is marked in Blue)

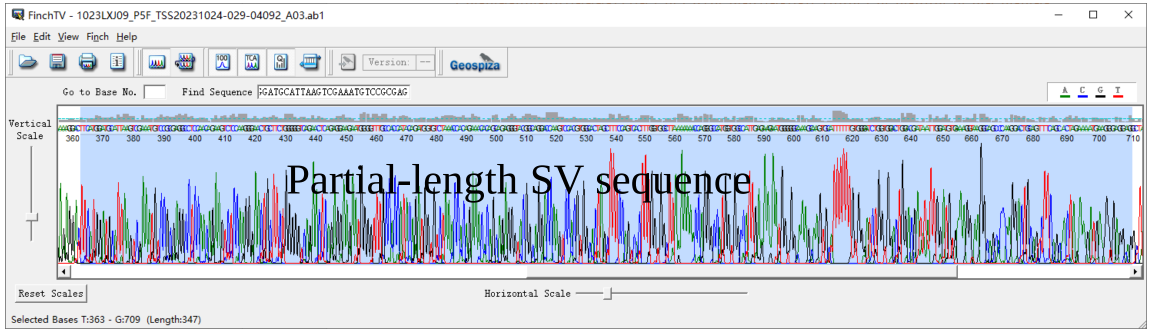

5’ primer Sanger trace of LCL5 (Child)

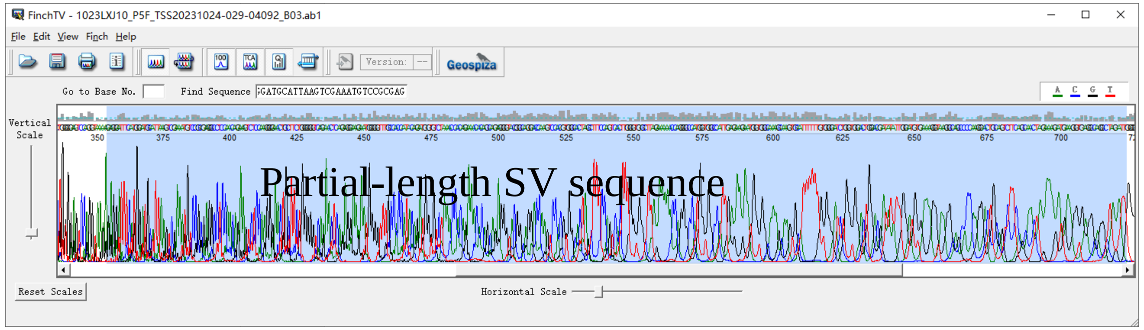

5’ primer Sanger trace of LCL8 (Mother)

■ Conclusion:

This SV is present in the Child and Mother genomes, therefore, it is not a *de novo* SV

Sanger sequencing results for ID FP-34 chr12-85640293-85640294-INS

■ Sanger trace alignment evidences:

5’ Upstream (15bp) Downstream (15bp) 3’

Reference: TCAGAAACCTGATGG CTCAGCCAAAAAAGT

SV sequence: TCAGAAACCTGATGG CAGAGCCAAAAAAGTGACTAAAAAGAATGAATTAAGAGGTCAGCGAAAAA CTCAGCCAAAAAAGT

(SV sequence length: 80bp)

LCL5 (Child) Sanger trace (80bp): TCAGAAACCTGATGGCAGAGCCAAAAAAGTGACTAAAAAGAATGAATTAAGAGGTCAGCGAAAAACTCAGCCAAAAAAGT

LCL8 (Mother) Sanger trace (65bp): TCAGAAACCTGATGGCAGAGCCAAAAAAGTGACTAAAAAGAATGAATTAAGAGGTCAGCGAAAAA

■ Sanger trace raw evidences:

(The SV sequence in Sanger trace is marked in Blue)

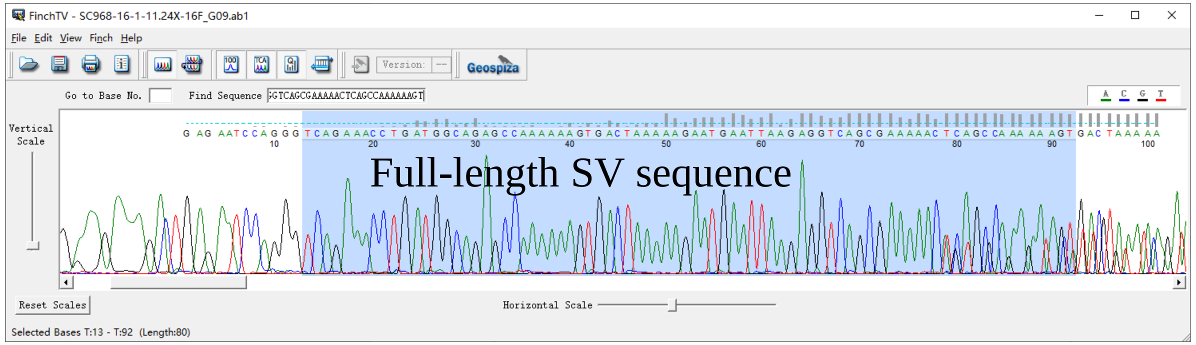

5’ primer Sanger trace of LCL5 (Child)

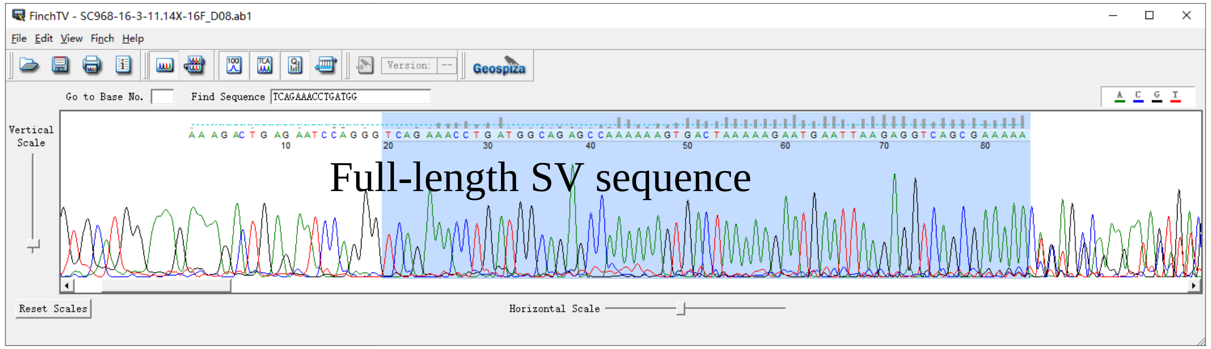

5’ primer Sanger trace of LCL8 (Mother)

■ Conclusion:

This SV is present in the Child and Mother genomes, therefore, it is not a *de novo* SV

Sanger sequencing results for ID FP-35 chr12-90349355-90349427-DUP

■ Sanger trace alignment evidences:

5’ Upstream (15bp) Downstream (15bp) 3’

Reference: TTTAGAATCTATGGA TTTTGACAGTAGTTT

SV sequence: TTTAGAATCTATGGA TATGCAAGTGTCATCAGATGAATTGA……CTCTAAATCATGCAGGGCCATGCCTGC TTTTGACAGTAGTTT

(SV sequence length: 193bp)

LCL5 (Child) Sanger trace (93bp) TTTAGAATCTATGGATATGCAAGTGTCATCAGAT…AAACCTCTAAATCATGCA

LCL7 (Father) Sanger trace (99bp) TTTAGAATCTATGGATATGCAAGTGTCATCAGAT……AAACCTCTAAATCATGCAGGTGCA

■ Sanger trace raw evidences: (The SV sequence in Sanger trace is marked in Blue)

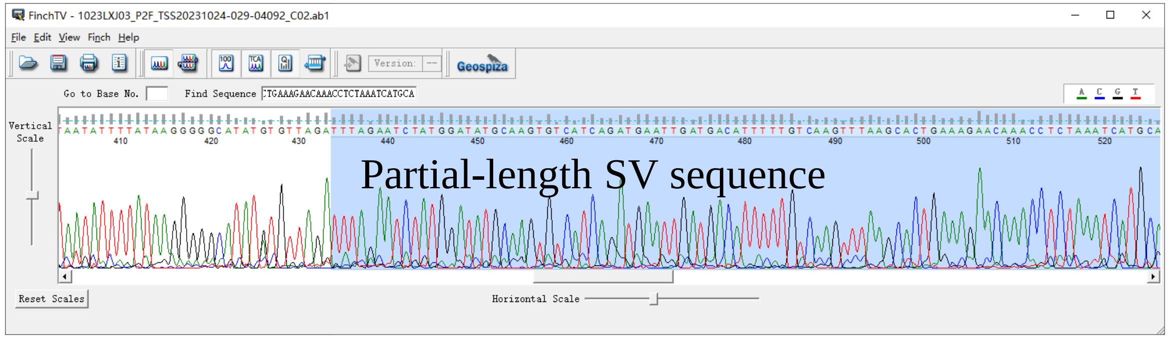

5’ primer Sanger trace of LCL5 (Child)

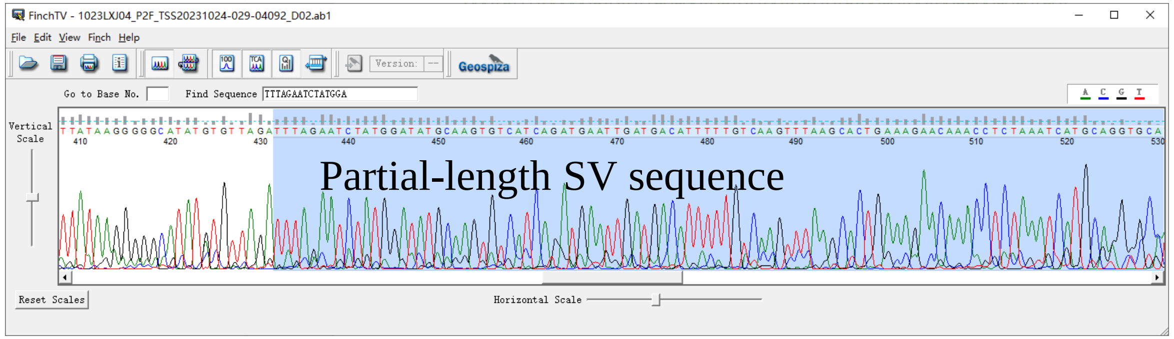

5’ primer Sanger trace of LCL7 (Father)

■ Conclusion:

This SV is present in the Child and Father genomes, therefore, it is not a *de novo* SV

Sanger sequencing results for ID FP-36 chr13-57178407-57214731-DEL

■ Sanger trace alignment evidences:

5’ Upstream (15bp) (36847bp) Downstream (15bp) 3’

Reference: CACTGCAAGCTCCGC CTCCCAGGTTCACACCATTCTCTTGCCTC....TCCTCATCCTCAATTGTAGATGGGTTTG CAATCCTCATCCTCA

SV sequence: CACTGCAAGCTCCGC CAATCCTCATCCTCA

(SV sequence length: 30bp)

LCL5 (Child) Sanger trace: CACTGCAAGCTCCGCCAATCCTCATCCTCA (30bp)

LCL8 (Mother) Sanger trace: CACTGCAAGCTCCGCCAATCCTCATCCTCA (30bp)

■ Sanger trace raw evidences: (The SV sequence in Sanger trace is marked in Blue)

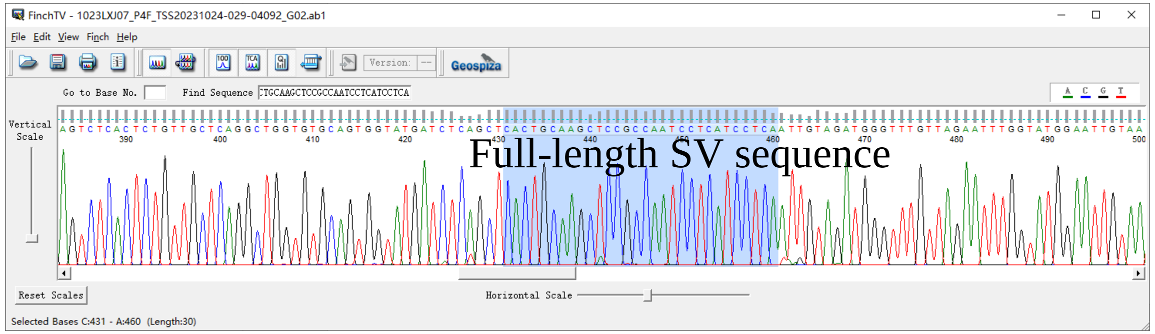

5’ primer Sanger trace of LCL5 (Child)

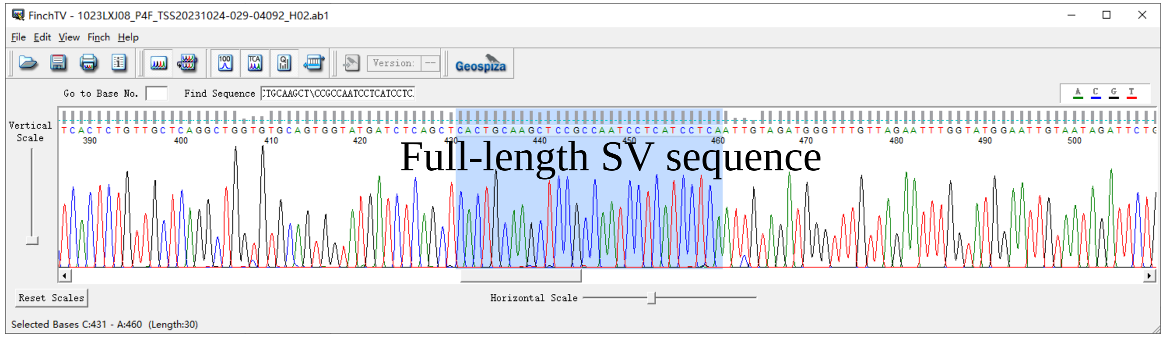

5’ primer Sanger trace of LCL8 (Mother)

■ Conclusion:

This SV is present in the Child and Mother genomes, therefore, it is not a *de novo* SV

Sanger sequencing results for ID FP-44 chr18-57864626-57864677-DEL

■ Sanger trace alignment evidences:

5’ Upstream (15bp) (50bp) Downstream (15bp) 3’

Reference: TTATCAAGACAGGTG GAAAGTGCTTTGAAACATGTTTAATAAATTACTTATCCAGCACTTAGCTA GTCCCTGCTACATGC

SV sequence: TTATCAAGACAGGTG GTCCCTGCTACATGC

(SV sequence length: 30bp)

LCL5 (Child) Sanger trace: TTATCAAGACAGGTGGTCCCTGCTACATGC (30bp)

LCL7 (Father) Sanger trace: TTATCAAGACAGGTGGTCCCTGCTACATGC (30bp)

■ Sanger trace raw evidences: (The SV sequence in Sanger trace is marked in Blue)

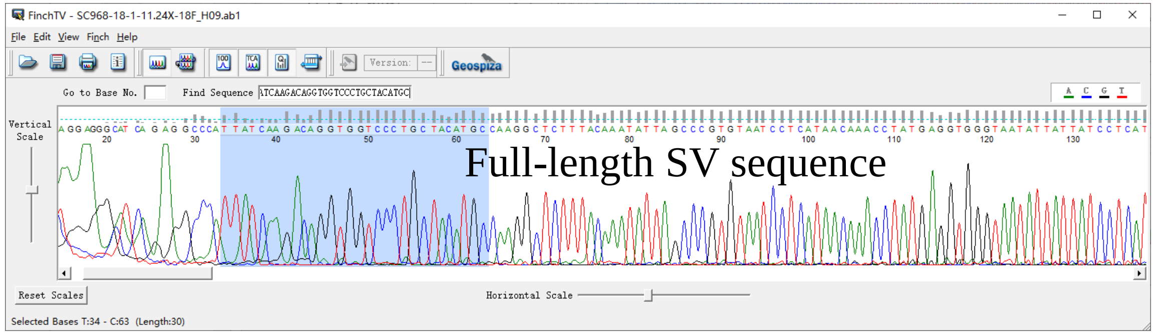

5’ primer Sanger trace of LCL5 (Child)

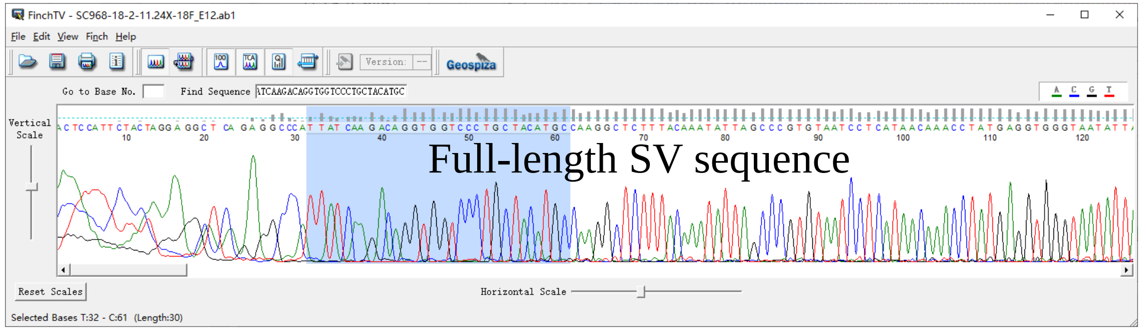

5’ primer Sanger trace of LCL7 (Father)

■ Conclusion:

This SV is present in the Child and Father genomes, therefore, it is not a *de novo* SV

Sanger sequencing results for ID FP-45 chr20-23314663-23314664-INS

■ Sanger trace alignment evidences:

5’ Upstream (15bp) ... Downstream (15bp) 3’

Reference: CATTCCTCCAGTCTG ... CTGGTCAAAGCAATT

SV sequence: CATTCCTCCAGTCTG CTTCCCACAGGCTGCACCATACTTCCAC…..AGAACTAGAATGTCATTCCTCCAGTCTG CTGGTCAAAGCAATT

(SV sequence length: 139bp)

LCL5 (Child) Sanger trace (139bp): CATTCCTCCAGTCTGCTTCCCACAGGCTGCACCATACTTCCAC…..AGAACTAGAATGTCATTCCTCCAGTCTGCTGGTCAAAGCAATT

LCL7 (Father) Sanger trace (139bp): CATTCCTCCAGTCTGCTTCCCACAGGCTGCACCATACTTCCAC…..AGAACTAGAATGTCATTCCTCCAGTCTGCTGGTCAAAGCAATT

■ Sanger trace raw evidences: (The SV sequence in Sanger trace is marked in Blue)

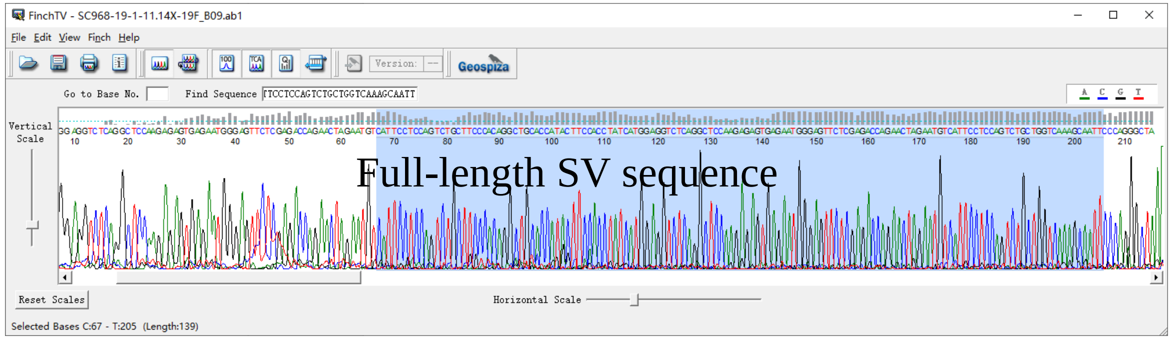

5’ primer Sanger trace of LCL5 (Child)

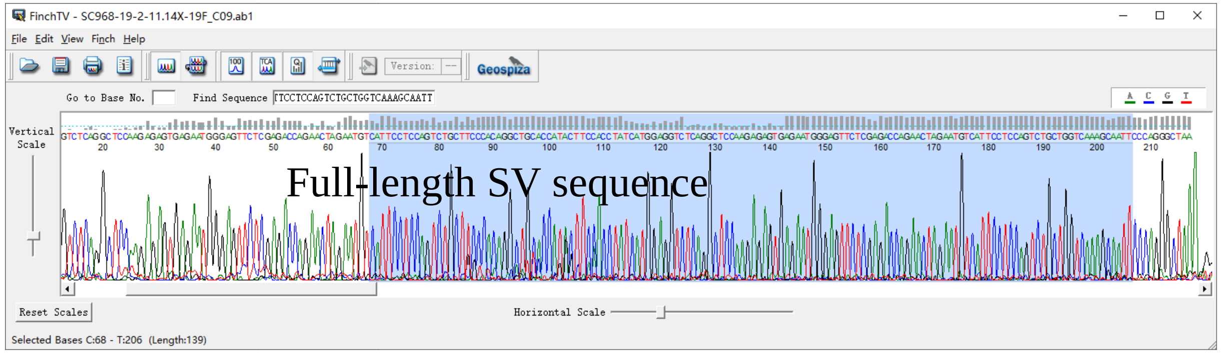

5’ primer Sanger trace of LCL7 (Father)

■ Conclusion:

This SV is present in the Child and Father genomes, therefore, it is not a *de novo* SV

Sanger sequencing results for ID FP-46 chr20-44557062-44557581-DUP

Sanger trace alignment evidences:

5' Upstream (15bp) ... Downstream (15bp) 3'

Reference: TCTATCTATTCTATT ... CACCTGTAGTCCCAG

SV sequence: TCTATCTATTCTATT AAGGTCGTATATAAATAAATGATTTT....AAAAAAAATTAGCCAGCCGTGGTGGCGGG CACCTGTAGTCCCAG

(SV sequence length: 1184bp)

LCL5 (Child) Sanger trace (464bp): TCTATCTATTCTATTAAGGTCGTATATA….. CAGCTGGGTGACAGGTGAC

LCL8 (Mother) Sanger trace (462bp): TCTATCTATTCTATTAAGGTCGTATATA….. CAGCTGGGTGACAGGTG

Sanger trace raw evidences:

(The SV sequence in Sanger trace is marked in Blue)

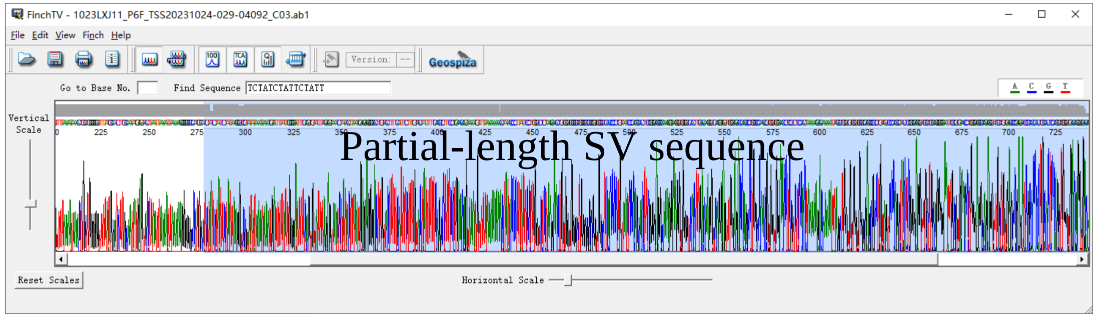

5' primer Sanger trace of LCL5 (Child)

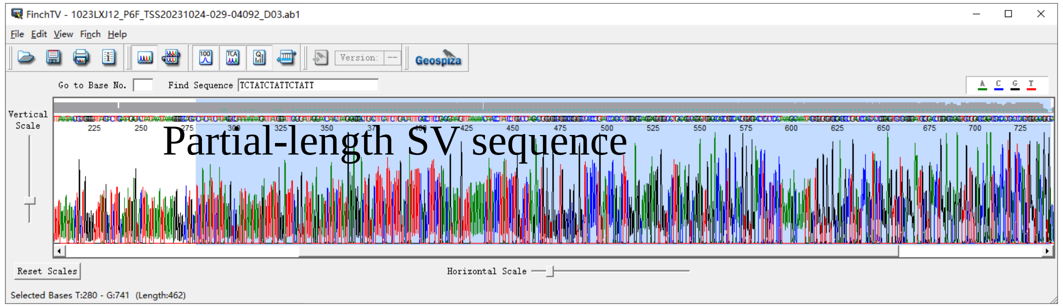

5' primer Sanger trace of LCL8 (Mother)

Conclusion:

This SV is present in the Child and Mother genomes, therefore, it is not a *de novo* SV

Supplementary File 8. Retrieved eight CSVs from HCC1395 N-T paired cell line

chr2_221242363_chr6_52995665_+-, TRA and chr2_221242434_chr2_221250908_++, INV

IGV evidence

HCC1395

HCC1395BL

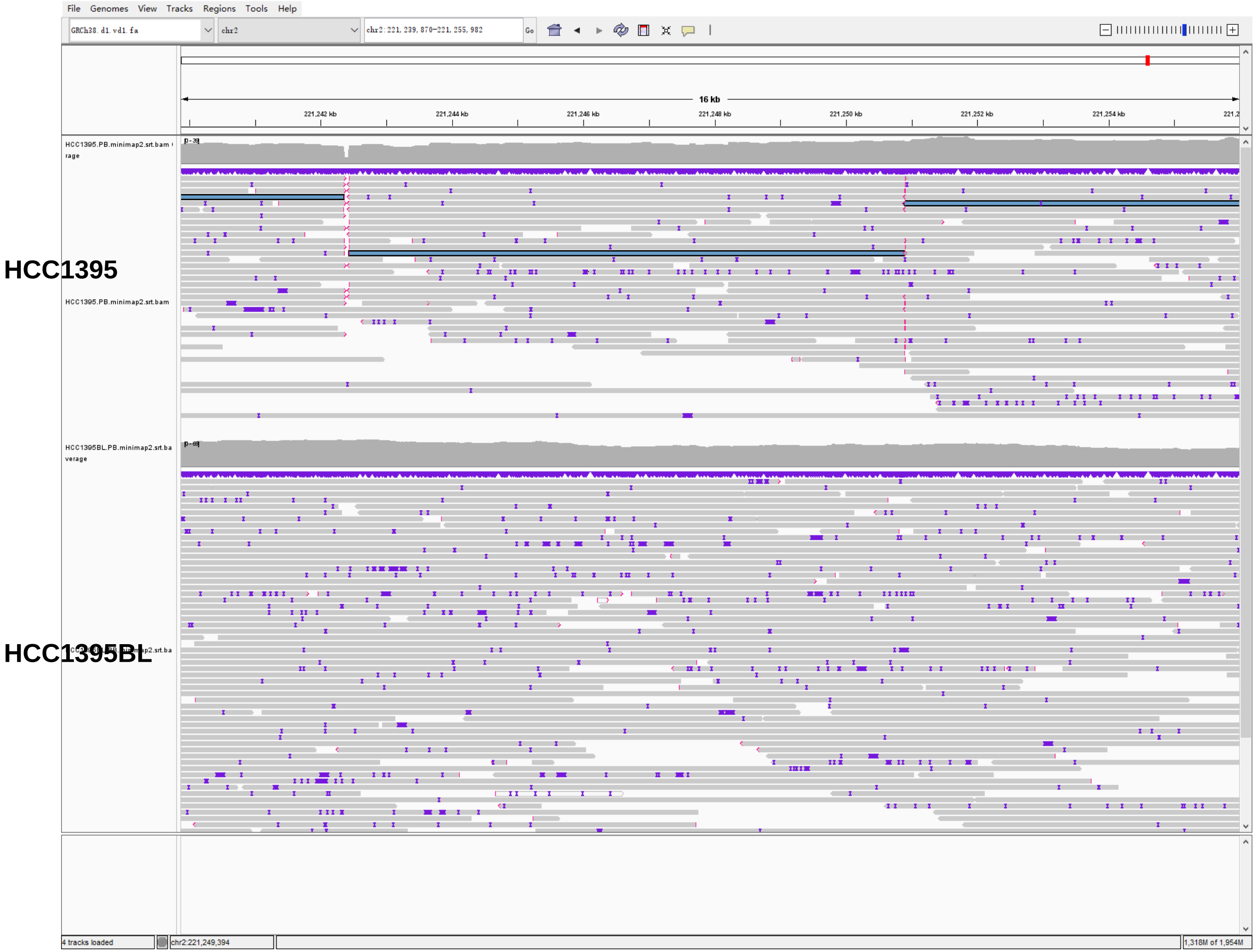

Read Dotplot evidence

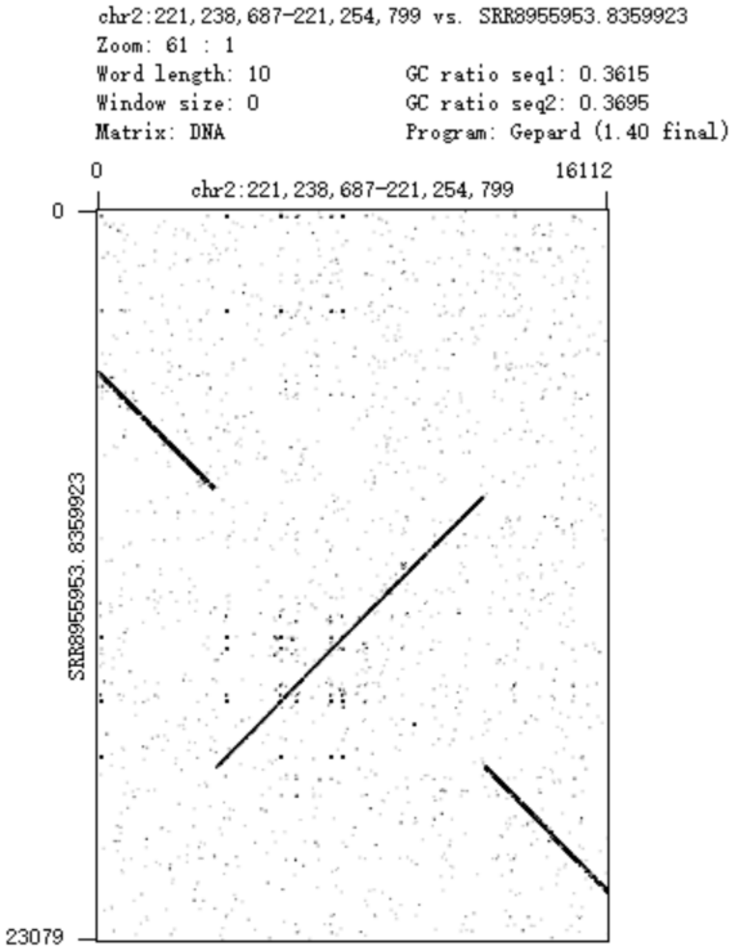

Callset		Types	Details
**Previous:**	two SSVs	a **TRA** and a **INV**	**TRA**: chr2-221242363-chr6-52995665, **INV**: 8499bp, chr2-221242434-221250908
**SVision-pro:**	one CSV	**dDUP+DEL+INV**	**dDUP**: 202bp, chr6-52995665-52995866, **DEL**: 71bp, chr2-221242364-221242435, **INV**: 8464bp, chr2-221242434-221250898

chr2_239644967_chr2_239644967_+-, INS

IGV evidence

HCC1395

HCC1395BL

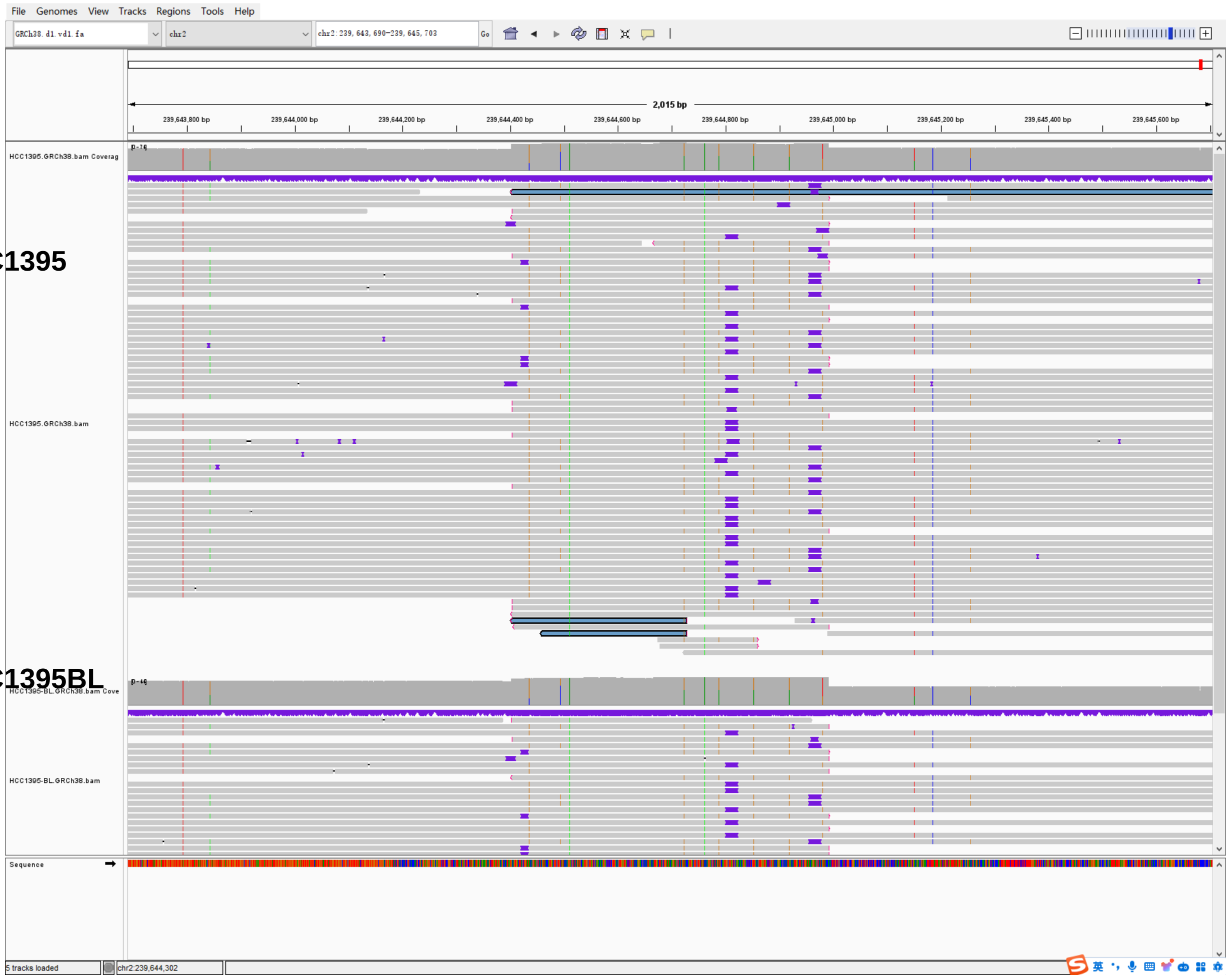

Read Dotplot evidence

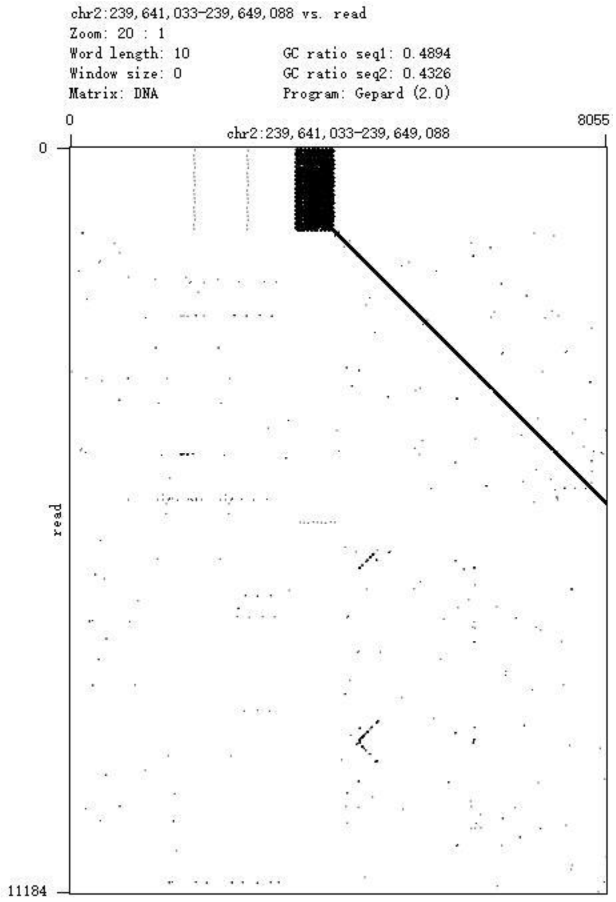

Callset		Types	Details
**Previous:**	one SSVs	a **INS**	**INS**: 654bp, chr2-239644967-239644967
**SVision-pro:**	one CSV	**INS+tDUP**	**INS**: 325bp, chr2-239644728-239644728, **tDUP**: 324bp, chr2-239644404-239644727

chr4_91316996_chr4_91317164_++, INV and chr4_91316993_chr4_91349253, INV

IGV evidence

Read Dotplot evidence

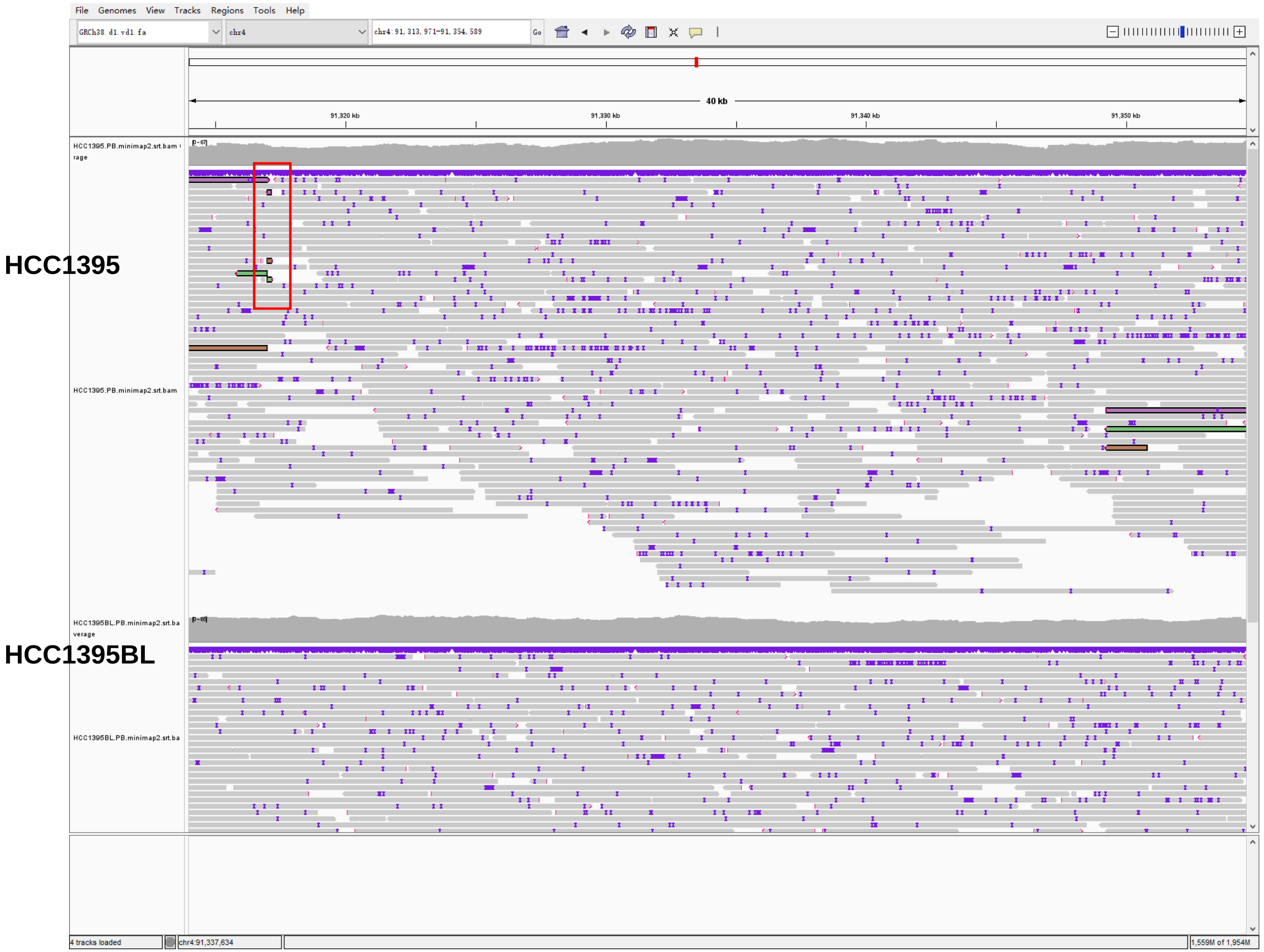

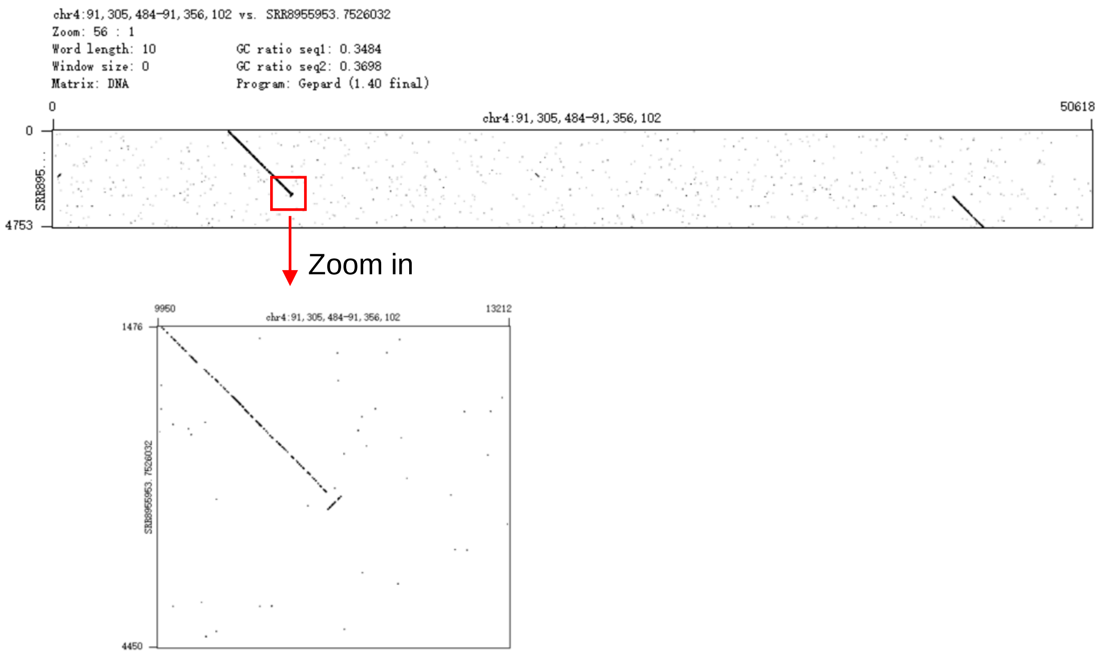

Callset		Types	Details
**Previous:**	two SSVs	two **INVs**	**INV**: 166bp, chr4-91316996-91317164, **INV**: 32273bp, chr4-91316993-91349253
**SVision-pro:**	one CSV	**INV+DEL**	**INV**: 168bp, chr4-91317011-91317179, **DEL**:32090bp, chr4-91317163-91349253

chr6_106354503_chr6_106362128_+-, DEL

IGV evidence

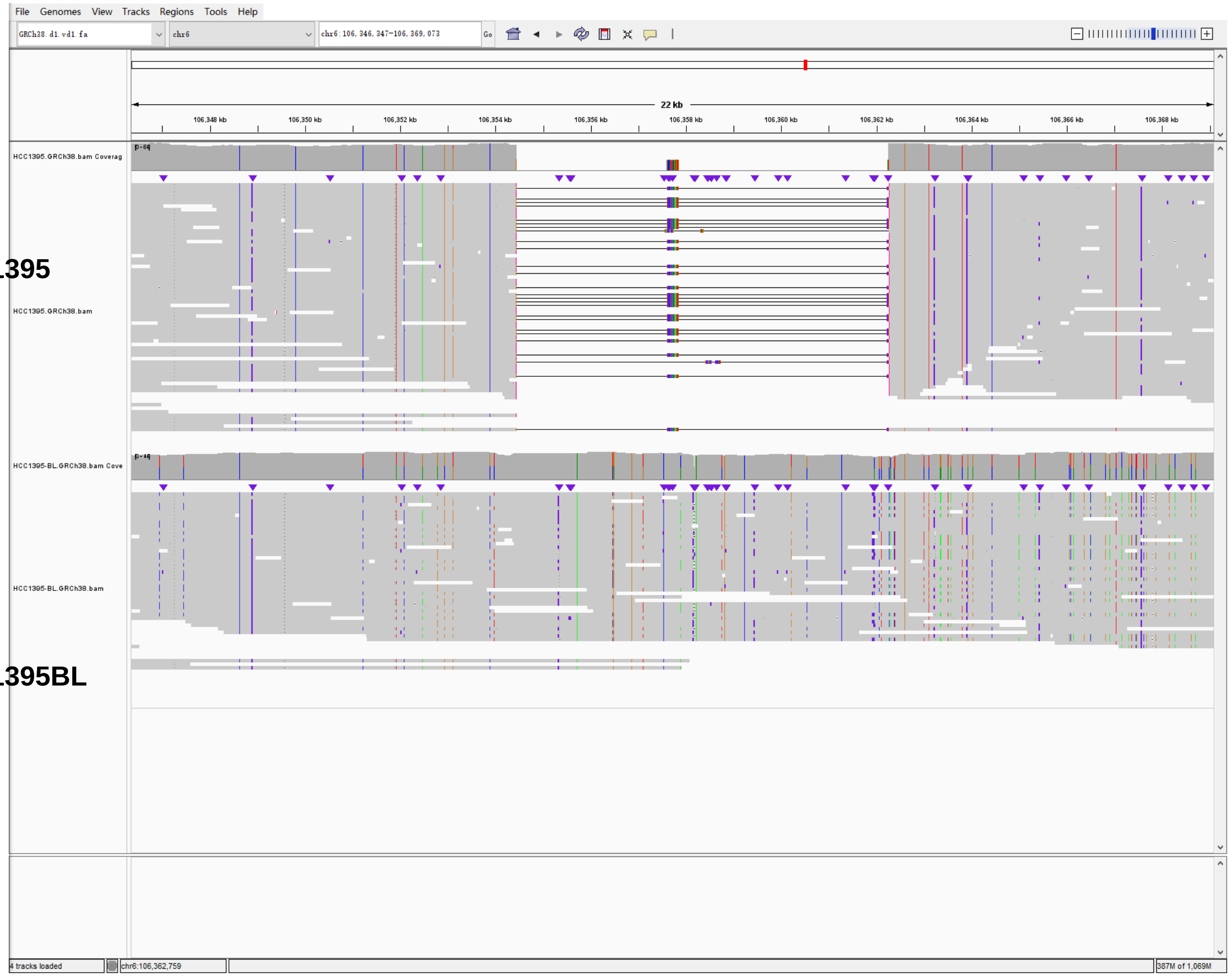

HCC1395

HCC1395BL

Read Dotplot evidence

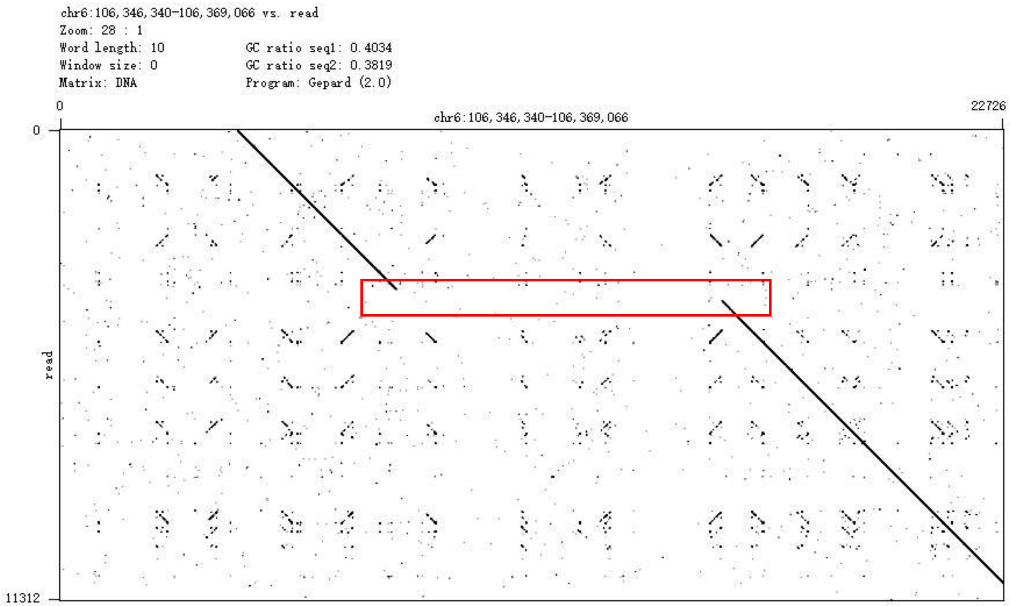

Callset		Types	Details
**Previous:**	one SSVs	one **DEL**	**DEL**: 7700bp, chr6-106354503-106362128
**SVision-pro:**	one CSV	**INV+DEL**	**INS**: 266bp, chr6-106354434-106354434, **DEL**:7831bp, chr6-106354435-106362266

chr7_54618433_chr7_54621260_-+, DUP **and** chr7_54618592_chr10_37277919_++, TRA

IGV evidence

HCC1395

HCC1395BL

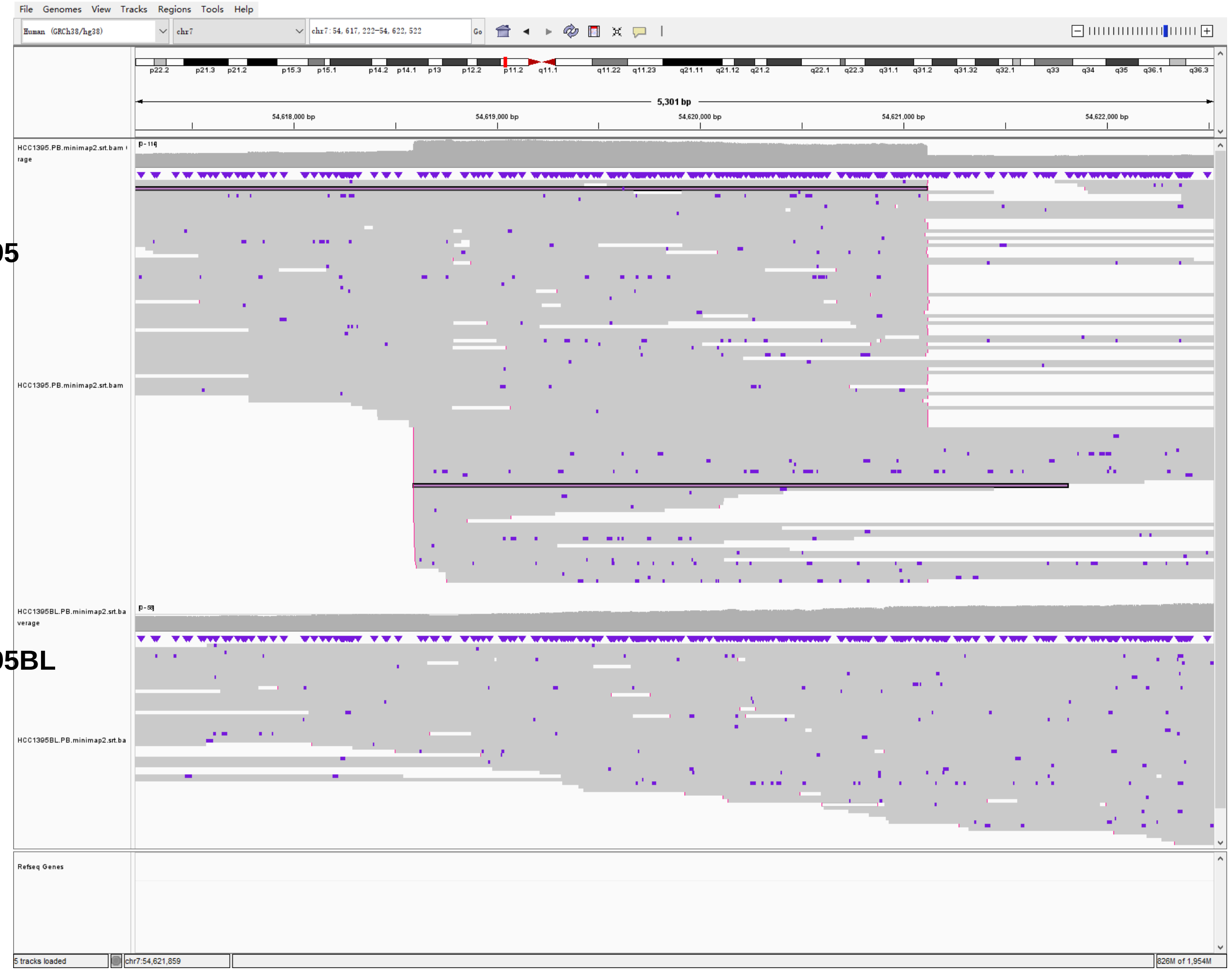

Read Dotplot evidence

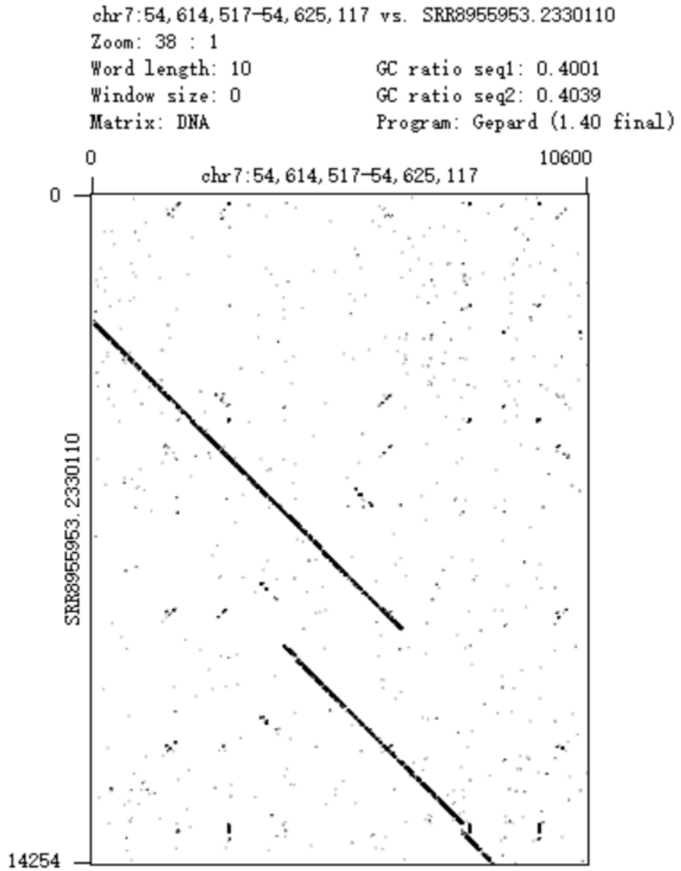

**Callset**		**Types**	**Details**
**Previous:**	two SSVs	a **DUP** and a **TRA**	**TRA**: chr7-54618592-chr10-37277919, **DUP**: 2715bp, chr7-54618433-54621260,
**SVision-pro:**	one CSV	**idDUP+tDUP**	**idDUP**:353bp, chr10-37277919-37278271, **tDUP**: 2526bp, chr7-54618592-54621117

chr11_65440714_chr11_65444882_-+, DUP

IGV evidence

HCC1395

HCC1395BL

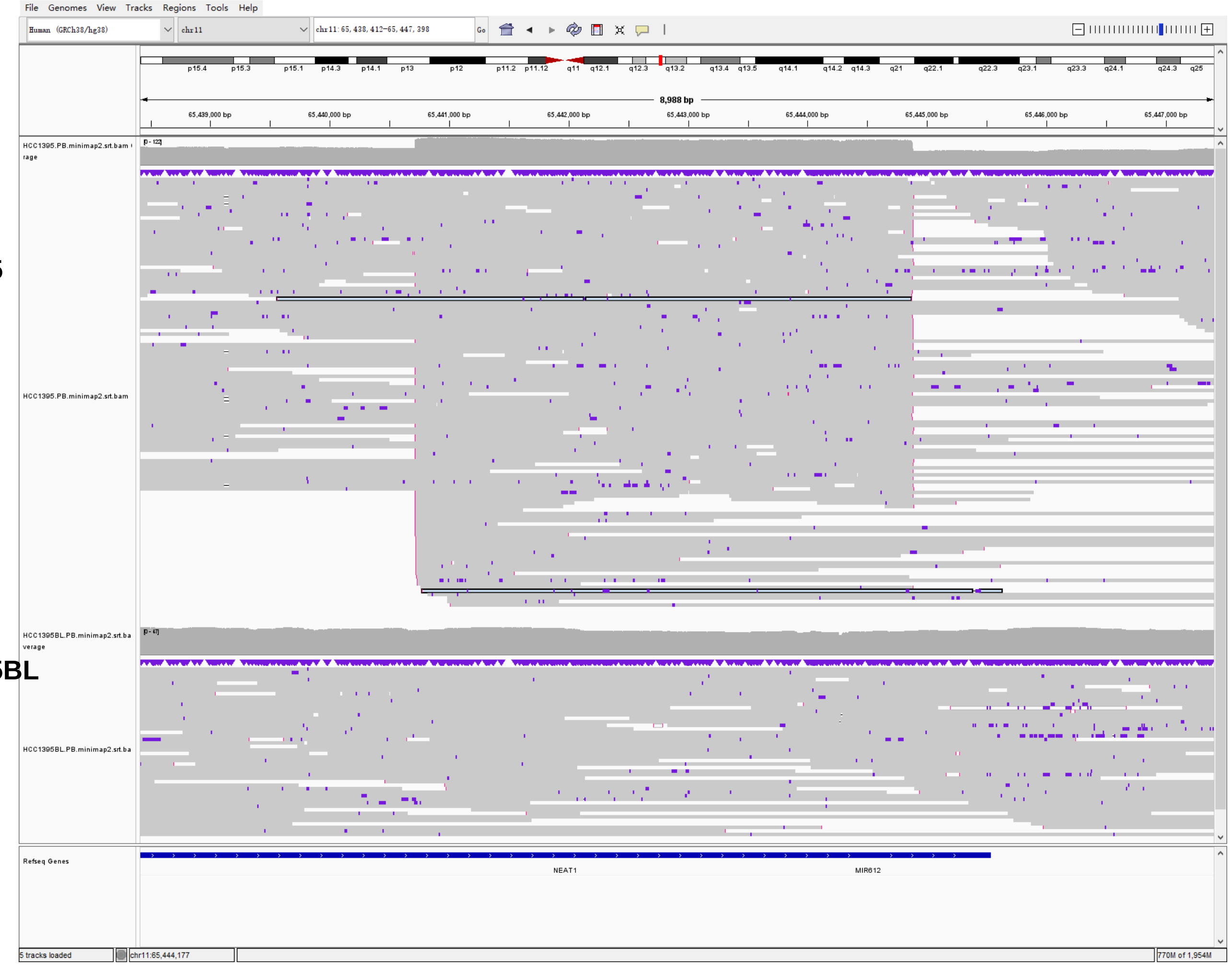

Read Dotplot evidence

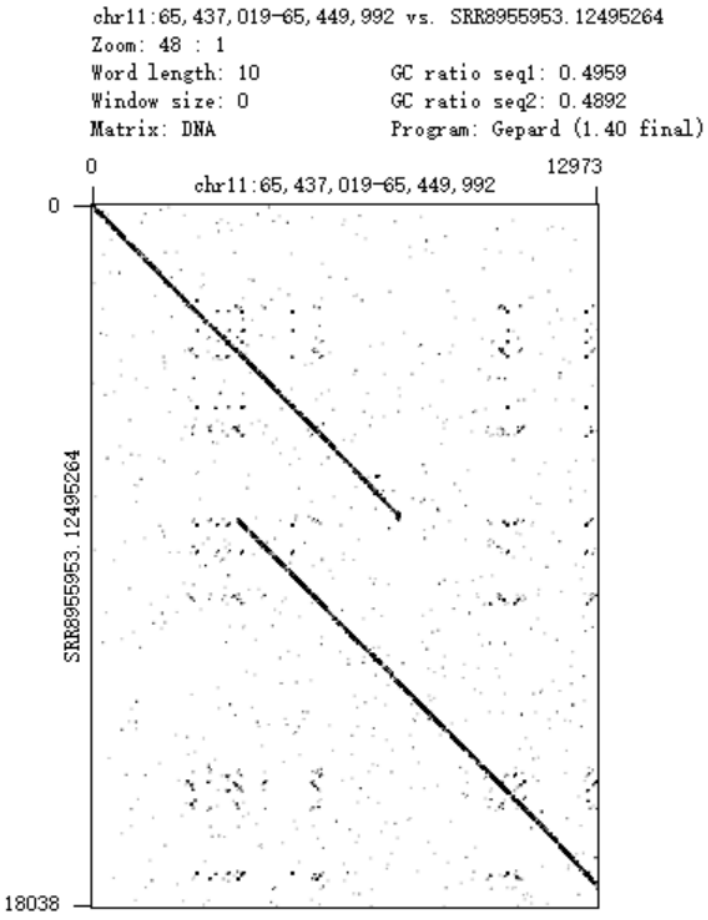

Callset		Types	Details
**Previous:**	one SSV	**DUP**	**DUP**: 4168bp, chr11-65440714-65440714
**SVision-pro:**	one CSV	**INS**+**tDUP**	**INS**: 80bp, chr11-65444884-65444884, **tDUP**: 4170bp, chr11-65440715-65444884

chr11_114917471_chr11_114930951_++, INV

IGV evidence

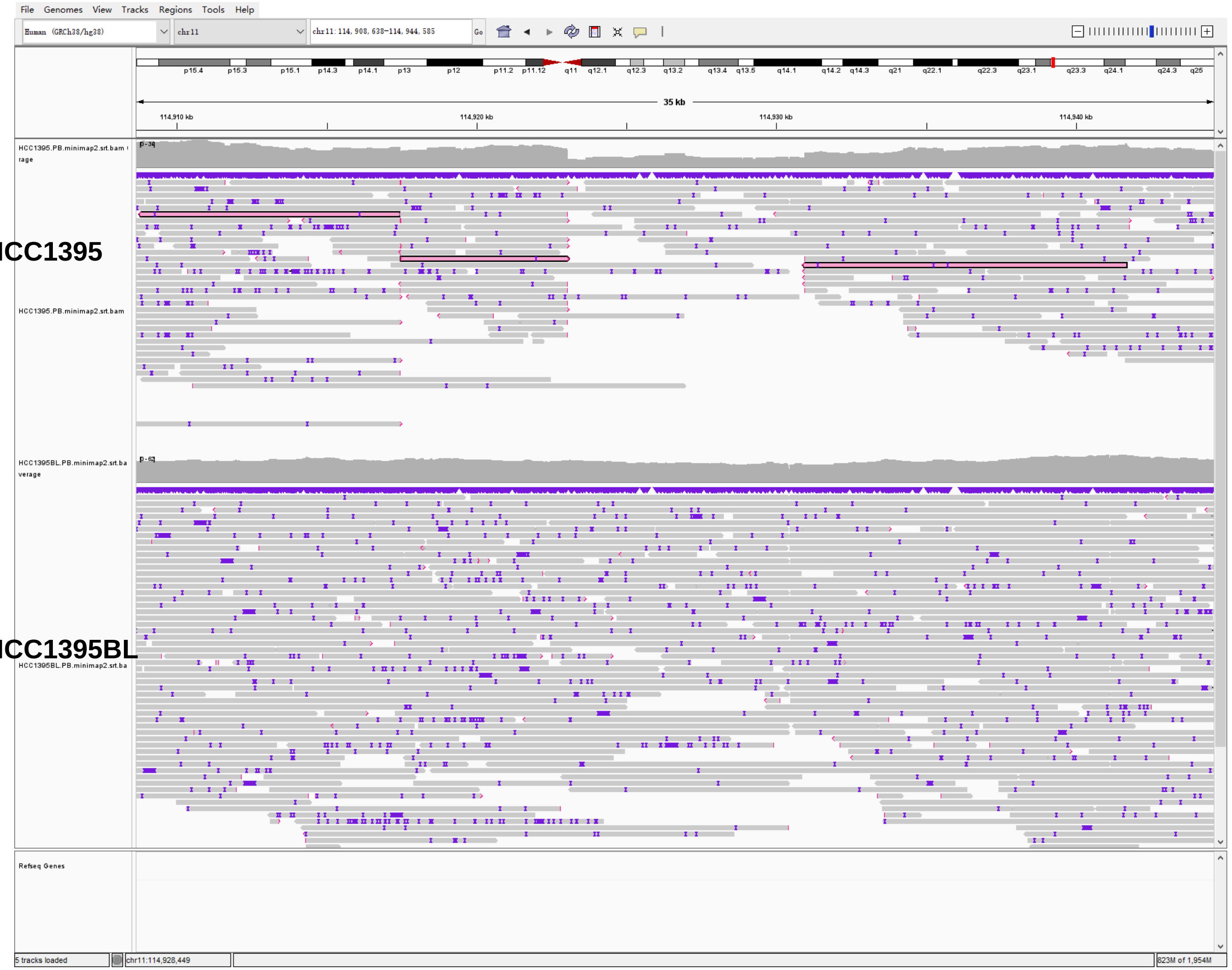

Read Dotplot evidence

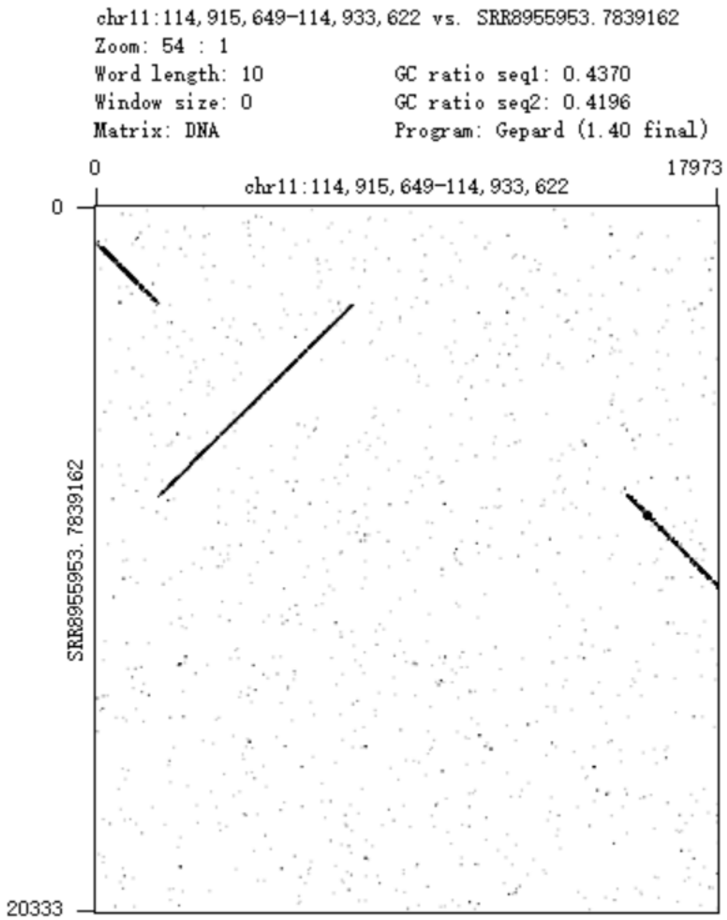

Callset		Types	Details
**Previous:**	one SSV	**INV**	**INV**_21903bp_ chr11_114917471_114930951
**SVision-pro:**	one CSV	**INV**+**DEL**	**INV**_5558bp_chr11_114917471_114923029_, **DEL**_7913bp_chr11_114923039_114930952

chr14_91643971_chr14_91654678_+-, DUP

IGV evidence

HCC1395

HCC1395BL

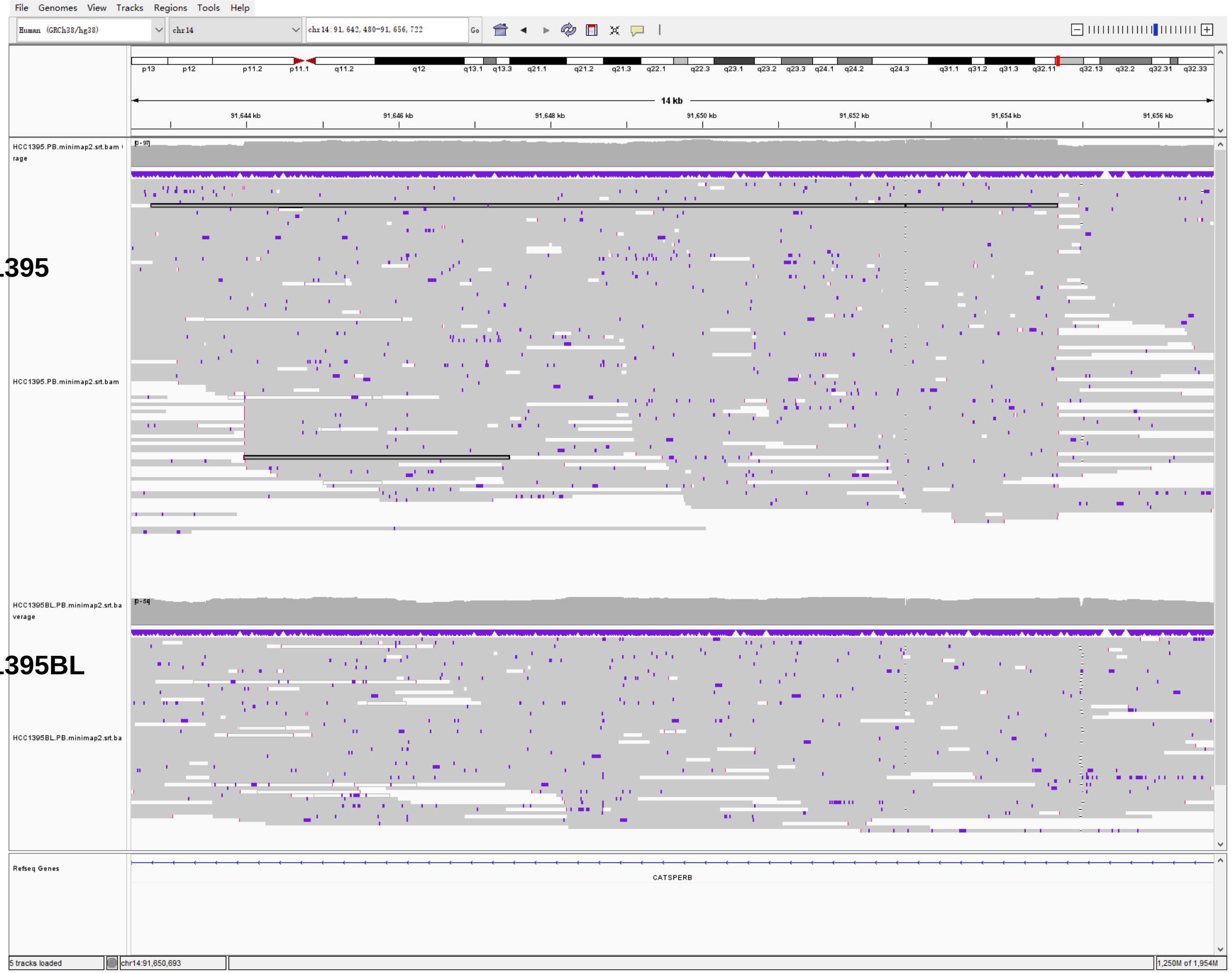

Read Dotplot evidence

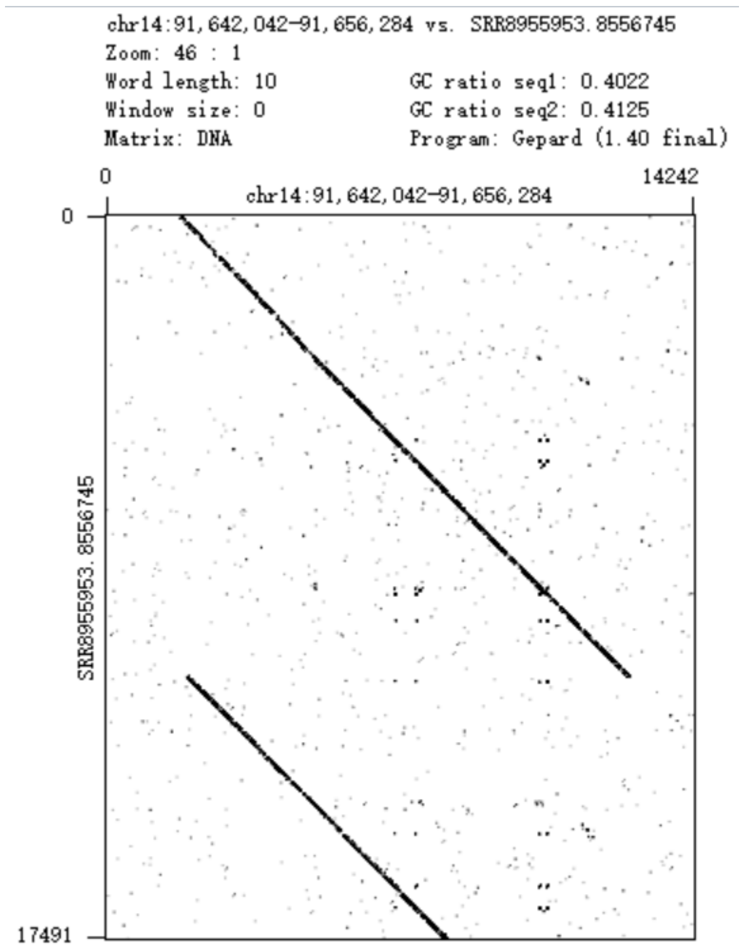

Callset		Types	Details
**Previous:**	one SSV	**DUP**	**DUP**: 21903bp, chr14-91643971-91654678
**SVision-pro:**	one CSV	**INS+tDUP**	**INS**: 67bp, chr14-91654641-91654641, **tDUP**: 10660bp, chr14-91643981-91654640
