# Supplementary material for: De novo and somatic structural variant discovery with SVision-pro
Source: Nat Biotechnol. 2024 Mar 22;43(2):181–5. doi: 10.1038/s41587-024-02190-7 (PMC11825360; doi:10.1038/s41587-024-02190-7)
Supplement: Supplementary file 2 — Reporting Summary [file 41587_2024_2190_MOESM2_ESM.pdf]

nature portfolio

Corresponding author(s): Kai Ye

Last updated by author(s): Feb 21, 2024

Reporting Summary

Nature Portfolio wishes to improve the reproducibility of the work that we publish. This form provides structure for consistency and transparency in reporting. For further information on Nature Portfolio policies, see our Editorial Policies and the Editorial Policy Checklist.

Statistics

For all statistical analyses, confirm that the following items are present in the figure legend, table legend, main text, or Methods section.

n/a	Confirmed	
☐	☒	The exact sample size (*n*) for each experimental group/condition, given as a discrete number and unit of measurement
☒	☐	A statement on whether measurements were taken from distinct samples or whether the same sample was measured repeatedly
☒	☐	The statistical test(s) used AND whether they are one- or two-sided<br>*Only common tests should be described solely by name; describe more complex techniques in the Methods section.*
☒	☐	A description of all covariates tested
☒	☐	A description of any assumptions or corrections, such as tests of normality and adjustment for multiple comparisons
☐	☒	A full description of the statistical parameters including central tendency (e.g. means) or other basic estimates (e.g. regression coefficient) AND variation (e.g. standard deviation) or associated estimates of uncertainty (e.g. confidence intervals)
☒	☐	For null hypothesis testing, the test statistic (e.g. *F*, *t*, *r*) with confidence intervals, effect sizes, degrees of freedom and *P* value noted<br>*Give P values as exact values whenever suitable.*
☒	☐	For Bayesian analysis, information on the choice of priors and Markov chain Monte Carlo settings
☒	☐	For hierarchical and complex designs, identification of the appropriate level for tests and full reporting of outcomes
☒	☐	Estimates of effect sizes (e.g. Cohen's *d*, Pearson's *r*), indicating how they were calculated

Our web collection on statistics for biologists contains articles on many of the points above.

Software and code

Policy information about availability of computer code

Data collection	wget (GNU v1.14) was used to download all published datasets from their official ftps.
Data analysis	Minimap2 (v2.20-r1061) and NGMLR (v0.2.7) were used for long read alignment.<br>SVision-pro (v1.6, https://github.com/songbowang125/SVision-pro.git), SVision (v1.3.9), Sniffles2 (v2.0.7), cuteSV (v2.0.2), pbsv (v2.9.0), debreak (v1.0.2), SVDSS (v1.0.5) and nanomonsv (v0.5.0) were used for structural variant calling.<br>Jasmine (v1.1.5) and SURVIVOR (v1.0.7) were used for callset merging.<br>Truvari (v3.5.0) and BEDtools (v2.30.0) were used for performance evaluation.<br>IGV (v2.16.2) and Gepard (v1.4.0) were used to create dotplot for manual inspection.<br>Vapor (version default) was used to perform computational validation<br>SVision-pro (v1.6) is available at GitHub (https://github.com/songbowang125/SVision-pro.git)<br>The scripts for model training, custom performance valuation and simulated data generation are available at GitHub (https://github.com/songbowang125/SVision-pro-Utils.git).

For manuscripts utilizing custom algorithms or software that are central to the research but not yet described in published literature, software must be made available to editors and reviewers. We strongly encourage code deposition in a community repository (e.g. GitHub). See the Nature Portfolio guidelines for submitting code & software for further information.

Data

Policy information about availability of data

All manuscripts must include a data availability statement. This statement should provide the following information, where applicable:

- Accession codes, unique identifiers, or web links for publicly available datasets
- A description of any restrictions on data availability
- For clinical datasets or third party data, please ensure that the statement adheres to our policy

The human reference genome GRCh37: http://ftp-trace.ncbi.nih.gov/1000genomes/ftp/technical/reference/phase2_reference_assembly_sequence/hs37d5.fa.gz
The human reference genome GRCh38: http://ftp.1000genomes.ebi.ac.uk/vol1/ftp/technical/reference/GRCh38_reference_genome/
NA19238 HiFi: http://ftp.1000genomes.ebi.ac.uk/vol1/ftp/data_collections/HGSVC2/working/20191205_YRI_PacBio_NA19238_HIFI/
NA19239 HiFi: http://ftp.1000genomes.ebi.ac.uk/vol1/ftp/data_collections/HGSVC2/working/20191205_YRI_PacBio_NA19239_HIFI/
NA19240 HiFi: http://ftp.1000genomes.ebi.ac.uk/vol1/ftp/data_collections/HGSVC2/working/20191005_YRI_PacBio_NA19240_HiFi/
HG00512 HiFi: http://ftp.1000genomes.ebi.ac.uk/vol1/ftp/data_collections/HGSVC2/working/20191031_CHS_PacBio_HG00512_HiFi/
HG00513 HiFi: http://ftp.1000genomes.ebi.ac.uk/vol1/ftp/data_collections/HGSVC2/working/20191031_CHS_PacBio_HG00513_HiFi/
HG00514 HiFi: http://ftp.1000genomes.ebi.ac.uk/vol1/ftp/data_collections/HGSVC2/working/20200731_CHS_PacBio_HG00514_HiFi_reseq/
HG00731 HiFi: http://ftp.1000genomes.ebi.ac.uk/vol1/ftp/data_collections/HGSVC2/working/20190925_PUR_PacBio_HiFi/
HG00732 HiFi: http://ftp.1000genomes.ebi.ac.uk/vol1/ftp/data_collections/HGSVC2/working/20190925_PUR_PacBio_HiFi/
HG00733 HiFi: http://ftp.1000genomes.ebi.ac.uk/vol1/ftp/data_collections/HGSVC2/working/20190925_PUR_PacBio_HiFi/
HG002 HiFi: https://ftp-trace.ncbi.nlm.nih.gov/giab/ftp/data/AshkenazimTrio/HG002_NA24385_son/PacBio_CCS_15kb_20kb_chemistry2/
HG003 HiFi: https://ftp-trace.ncbi.nlm.nih.gov/giab/ftp/data/AshkenazimTrio/HG003_NA24149_father/PacBio_CCS_15kb_20kb_chemistry2/
HG004 HiFi: https://ftp-trace.ncbi.nlm.nih.gov/giab/ftp/data/AshkenazimTrio/HG004_NA24143_mother/PacBio_CCS_15kb_20kb_chemistry2/
HG005 HiFi: https://ftp-trace.ncbi.nlm.nih.gov/giab/ftp/data/ChineseTrio/HG005_NA24631_son/PacBio_CCS_15kb_20kb_chemistry2/
HG006 HiFi: https://ftp-trace.ncbi.nlm.nih.gov/giab/ftp/data/ChineseTrio/HG006_NA24694-huCA017E_father/PacBio_CCS_15kb_20kb_chemistry2
HG007 HiFi: https://ftp-trace.ncbi.nlm.nih.gov/giab/ftp/data/ChineseTrio/HG007_NA24695-hu38168_mother/PacBio_CCS_15kb_20kb_chemistry2
Chinese Quartet LCL5 HiFi: https://chinese-quartet.org/#/data/download/quartet-genomics
Chinese Quartet LCL6 HiFi: https://chinese-quartet.org/#/data/download/quartet-genomics
Chinese Quartet LCL7 HiFi: https://chinese-quartet.org/#/data/download/quartet-genomics
Chinese Quartet LCL8 HiFi: https://chinese-quartet.org/#/data/download/quartet-genomics
HG002 ONT: https://ftp-trace.ncbi.nlm.nih.gov/giab/ftp/data/AshkenazimTrio/HG002_NA24385_son/UCSC_Ultralong_OxfordNanopore_Promethion/
HG003 ONT: https://ftp-trace.ncbi.nlm.nih.gov/giab/ftp/data/AshkenazimTrio/HG003_NA24149_father/UCSC_Ultralong_OxfordNanopore_Promethion/
HG004 ONT: https://ftp-trace.ncbi.nlm.nih.gov/giab/ftp/data/AshkenazimTrio/HG004_NA24143_mother/UCSC_Ultralong_OxfordNanopore_Promethion/
HG005 ONT: https://ftp-trace.ncbi.nlm.nih.gov/giab/ftp/data/ChineseTrio/HG005_NA24631_son/UCSC_Ultralong_OxfordNanopore_Promethion/
HG006 ONT: https://ftp-trace.ncbi.nlm.nih.gov/giab/ftp/data/ChineseTrio/HG006_NA24694-huCA017E_father/UCSC_Ultralong_OxfordNanopore_Promethion/
HG007 ONT: https://ftp-trace.ncbi.nlm.nih.gov/giab/ftp/data/ChineseTrio/HG007_NA24695-hu38168_mother/UCSC_Ultralong_OxfordNanopore_Promethion/
Chinese Quartet LCL5 ONT: https://chinese-quartet.org/#/data/download/quartet-genomics
Chinese Quartet LCL6 ONT: https://chinese-quartet.org/#/data/download/quartet-genomics
Chinese Quartet LCL7 ONT: https://chinese-quartet.org/#/data/download/quartet-genomics
Chinese Quartet LCL8 ONT: https://chinese-quartet.org/#/data/download/quartet-genomics
HCC1395 CCS: https://downloads.pacbcloud.com/public/revio/2023Q2/HCC1395/HCC1395/
HCC1395 ONT: https://www.ncbi.nlm.nih.gov/sra?term=SRP162370
HCC1395 CLR: https://www.ncbi.nlm.nih.gov/sra?term=SRP162370
HCC1395BL CCS: https://downloads.pacbcloud.com/public/revio/2023Q2/HCC1395/HCC1395-BL/
HCC1395BL ONT: https://www.ncbi.nlm.nih.gov/sra?term=SRP162370
HCC1395BL CLR: https://www.ncbi.nlm.nih.gov/sra?term=SRP162370
HG002 SV callset: https://ftp-trace.ncbi.nlm.nih.gov/giab/ftp/data/AshkenazimTrio/analysis/NIST_SVs_Integration_v0.6/HG002_SVs_Tier1_v0.6.vcf.gz
HCC1395 SV callset: https://static-content.springer.com/esm/art%3A10.1186%2Fs13059-022-02816-6/MediaObjects/13059_2022_2816_MOESM4_ESM.xlsx

Human research participants

Policy information about studies involving human research participants and Sex and Gender in Research.

Reporting on sex and gender	Not relevant to our study
Population characteristics	Not relevant to our study
Recruitment	Not relevant to our study
Ethics oversight	Not relevant to our study

Note that full information on the approval of the study protocol must also be provided in the manuscript.

Field-specific reporting

Please select the one below that is the best fit for your research. If you are not sure, read the appropriate sections before making your selection.

☒ Life sciences ☐ Behavioural & social sciences ☐ Ecological, evolutionary & environmental sciences

Life sciences study design

All studies must disclose on these points even when the disclosure is negative.

Sample size	For de novo structural variant analysis, we collected 19 already-published samples from six family datasets, including Ashkenazim Trio, Chinese Trio, YRI Trio, CHS Trio, PUR Trio and Chinese Quartet.<br>For somatic structural variant analysis, we collected one already-published normal-tumor paired cell line, including HCC1395 and HCC1395BL.<br>These sample sizes were chosen based on the accessibility of already-published long-read-sequencing data. These sample sizes were sufficient for performance benchmarking of SVision-pro and other callers due to they possessed ground-truth callsets.
Data exclusions	No data were excluded in this study
Replication	Replication was not relevant to our study. This study used deterministic algorithms without statistical analysis, and this study aims to demonstrate SVision-pro and its application to de novo and somatic structural variant detection with long-read sequencing data.
Randomization	Randomization was not relevant to our study. SVision-pro is a deterministic method. and all analysis in this study was done with preexisting data sources.
Blinding	Blinding was not relevant to our study. We used publicly available data, no data acquisition or statistical analysis was involved. Besides, in this study, all softwares are deterministic and do not take advantages from knowing the origin of the input data.

Reporting for specific materials, systems and methods

We require information from authors about some types of materials, experimental systems and methods used in many studies. Here, indicate whether each material, system or method listed is relevant to your study. If you are not sure if a list item applies to your research, read the appropriate section before selecting a response.

Materials & experimental systems

n/a	Involved in the study	
☒	☐	Antibodies
☒	☐	Eukaryotic cell lines
☒	☐	Palaeontology and archaeology
☒	☐	Animals and other organisms
☒	☐	Clinical data
☒	☐	Dual use research of concern

Methods

n/a	Involved in the study	
☒	☐	ChIP-seq
☒	☐	Flow cytometry
☒	☐	MRI-based neuroimaging
